# A Sustainable Visible Light‐Mediated Synthesis of Benzoxazole 2‐Carboxylates/Carboxamides

**DOI:** 10.1002/chem.202502901

**Published:** 2025-11-02

**Authors:** Anna‐Dimitra D. Gerogiannopoulou, Olga G. Mountanea, Maria A. Theodoropoulou, Christoforos G. Kokotos, George Kokotos

**Affiliations:** ^1^ Laboratory of Organic Chemistry Department of Chemistry National and Kapodistrian University of Athens Panepistimiopolis Athens 15771 Greece

**Keywords:** benzoxazoles, cyclization, photochemistry, photoredox catalysis

## Abstract

The development of novel methods for the synthesis of substituted benzoxazoles is of wide interest, since such heterocyclic compounds exhibit various bioactivities. We report herein an efficient photochemical protocol for the oxidative cyclization of glycine derivatives to produce 2‐substituted benzoxazoles bearing ester or amide moieties. Cheap and commercially available 1,8‐dihydroxyanthraquinone serves as the organocatalyst in the presence of a nonprecious salt (CuI), while Kessil LED 427 nm is the source of irradiation. An extended substrate scope proves the generality of the method, which operates under mild conditions and in line with the principles of green chemistry. Direct infusion‐high resolution mass spectrometry studies supported the proposal of the reaction mechanism. Furthermore, ease derivatization of selected benzoxazole 2‐carboxylates/carboxamides demonstrates their utility as versatile intermediates for the synthesis of a wide range of compounds, thereby highlighting the value and broader applicability of the new photochemical protocol.

## Graphical abstract

An efficient photochemical protocol for the synthesis of 2‐ester or 2‐amide benzoxazoles was developed, utilizing 1,8‐dihydroxyanthraquinone and CuI as the photocatalytic system under LED 427 nm irradiation. Furthermore, 2‐ester/amide benzoxazoles serve as intermediates for the synthesis of potentially valuable compounds.

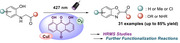

## 1 Introduction

**Figure 1:**
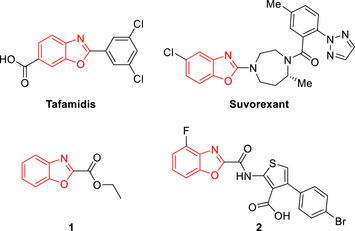
Selected FDA‐approved benzoxazole‐based drugs and bioactive benzoxazole 2‐carboxylates/carboxamides.

The benzoxazole moiety is among the most exploited heterocyclic motifs, since a variety of natural products, as well as bioactive compounds, bear this scaffold.^[^ ^1^, ^2^, ^3^, ^4^ ^]^ Various benzoxazole derivatives have been isolated from fungi, plants, or marine sources,^[^ ^4^ ^]^ while benzoxazoles also present interest as agrochemicals, exhibiting, for example, activity as pesticides.^[^ ^5^ ^]^ A plethora of benzoxazole‐based compounds have been found to exhibit various bioactivities, and some of them are approved drugs in clinical practice.^[^ ^1^, ^2^, ^3^, ^4^ ^]^ Examples of approved drugs bearing the benzoxazole core are tafamidis (Figure 1), a first‐in‐class medication for the treatment of transthyretin amyloidosis,^[^ ^6^ ^]^ or suvorexant (Figure 1), an orexin antagonist medication, which is used for the treatment of insomnia.^[^ ^7^ ^]^ Compounds **1** and **2** (Figure 1) are a benzoxazole 2‐carboxylate and a benzoxazole 2‐carboxamide, which exhibit antiallergic activity^[^ ^8^ ^]^ and CRAC channel inhibitory activity,^[^ ^9^ ^]^ respectively.

**Scheme 1:**
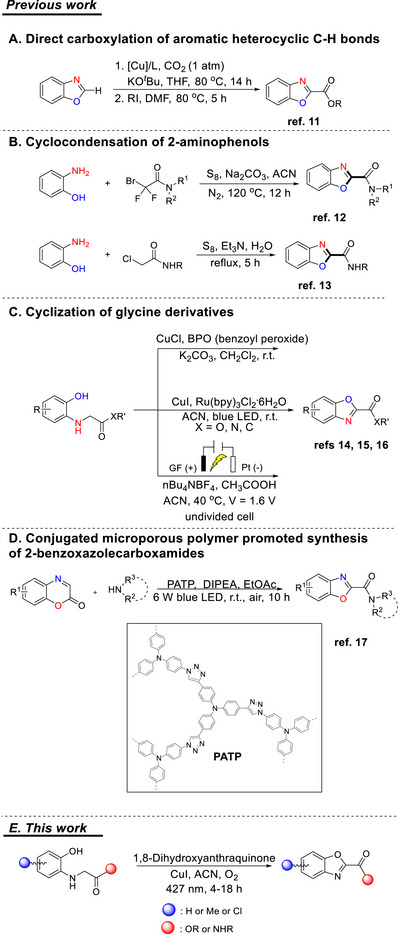
Methods for the synthesis of benzoxazole 2‐carboxylates/carboxamides.

The substitution of the benzoxazole core at the 2‐position considerably affects the biological and pharmacological properties of the benzoxazole products, and thus, a plethora of synthetic methods have been developed for the construction of 2‐aryl or 2‐alkyl benzoxazole derivatives.^[^ ^10^, ^11^ ^]^ However, only a few methods for the formation of benzoxazole 2‐carboxylates or 2‐carboxamides have been reported. In 2010, the direct carboxylation of benzoxazole with carbon dioxide via copper catalysis was demonstrated (Scheme 1, **A**),^[^ ^12^ ^]^ while later on, 2‐aminophenols were proven valuable starting materials for the construction of the benzoxazole ring (Scheme 1, **B**).^[^ ^13^, ^14^ ^]^ Their condensation with bromodifluoro compounds afforded benzoxazole 2‐carboxylates/carboxamides via S_8_‐triggered cleavage of three carbon − halogen bonds that also produced large amounts of acid waste.^[^ ^13^ ^]^ In addition, benzothiazole‐2‐carboxamides have been prepared by the cyclocondensation of 2‐chloracetamides with 2‐aminophenols in the presence of elemental sulfur under reflux in water.^[^ ^14^ ^]^ In 2021, Zhou et al. reported the cyclization of glycine derivatives, first through copper‐catalyzed oxidative C–H/O–H cross‐coupling using benzoyl peroxide^[^ ^15^ ^]^ and then through the oxidative cyclization by visible‐light photoredox catalysis using Ru(bpy)_3_Cl_2_·6H_2_O^[^ ^16^ ^]^ (Scheme 1, **C**). One year later, an electrochemical oxidation/cyclization of glycine derivatives was reported through intramolecular Shono‐type oxidative coupling (Scheme 1, **C**).^[^ ^17^ ^]^ Most recently, visible light has been introduced to synthesize benzoxazole 2‐carboxylates/carboxamides, starting from benzoxazinones and amines in the presence of a conjugated microporous polymer (PATP) (Scheme 1, **D**).^[^ ^18^ ^]^

In light of our long‐standing interest in photochemical transformations,^[^ ^19^, ^20^, ^21^, ^22^, ^23^, ^24^, ^25^, ^26^, ^27^ ^]^ and considering the underexplored bioactivity of benzoxazole 2‐carboxylates/carboxamides, we aimed at developing a general method for the synthesis of such heterocyclic derivatives. An alternative economical and sustainable method requires avoiding massive excess of oxidants and precious metal catalysts and harnessing visible light. Thus, we envisioned a visible light‐mediated procedure, employing an organocatalyst in the absence of a precious metal. Admittedly, the use of an organocatalyst represents a particularly attractive alternative, considering the relatively high cost associated with the previously used Ru(bpy)_3_Cl_2_·6H_2_O.^[^ ^16^ ^]^ Herein, we report an efficient protocol for the oxidative cyclization of glycine esters or amides using 1,8‐dihydroxyanthraquinone as the photocatalyst, in the presence of CuI, under LED 427 nm irradiation (Scheme 1, **E**).

## 2 Results and Discussion

Ethyl 2‐((2‐hydroxyphenyl)amino)acetate (**3a**) was selected as the model substrate for the cyclization reaction, since its successful employment would lead directly to the synthesis of **1**, which, as mentioned in the introduction, presents interesting biological properties.^[^ ^8^ ^]^ Diverse organocatalysts and Kessil LED lamps, covering wavelengths from the UVA to the visible light region, were studied. In all cases, copper (I) iodide (CuI) was used as the additive and acetonitrile (ACN) as the solvent. The results are summarized in Table 1. Anthraquinone‐based catalysts, as well as thioxanthone‐based catalysts, exhibited high or moderate catalytic efficiency (Table 1, entries 1 and 3–6), whereas 1,8‐dihydroxyanthraquinone proved to be the most effective (Table 1, entry 4), affording the desired product in 78% isolated yield. In contrast, significantly lower catalytic efficiencies were observed with benzophenone or acetophenone derivatives (Table 1, entries 7–9). Acenaphthenequinone facilitated high conversion of the starting material, though the isolated product yield remained moderate at 63% (Table 1, entry 10). Phenylglyoxylic acid was also ineffective (Table 1, entry 11). Notably, when anthraquinone was employed in the absence of CuI, the reaction yield decreased (Table 1, entries 1 vs. 2), highlighting the essential role of the dual catalytic system in this transformation.

**Table 1:** Organocatalyst screening.

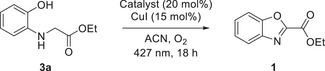		
Entry	Organocatalyst	Yield (%)^[^ ^a^ ^]^
1	Anthraquinone (**4a**) 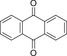	93 (72)
2^[^ ^b^ ^]^	Anthraquinone (**4a**)	58 (37)
3	Anthraquinone sulfonic acid sodium salt hydrate (**4b**) 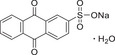	44 (36)
**4**	**1,8‐Dihydroxyanthraquinone (4c)** 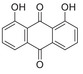	**89 (78)**
5	Thioxanthen‐9‐one (**4d**) 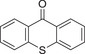	68 (47)
6	2,4‐Diethyl‐9*H*‐thioxanthen‐9‐one (**4e**) 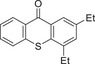	91 (68)
7	2,2‐Dimethoxy‐2‐phenylacetophenone (**4f**) 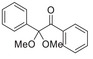	14 (18)
8	Benzophenone (**4** **g**) 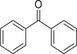	38 (28)
9	Acetophenone (**4** **h**) 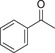	31 (23)
10	Acenaphthenequinone (**4i**) 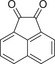	83 (63)
11	Phenylglyoxylic acid (**4j**) 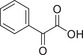	27

Reaction conditions: Ethyl 2‐((2‐hydroxyphenyl)amino)acetate (**3a**) (39 mg, 0.20 mmol, 1.00 equiv.), catalyst (0.04 mmol, 0.20 equiv.), and CuI (5.7 mg, 0.03 mmol, 0.15 equiv.) in ACN (2 mL), under air and LED (Kessil PR160L, 427 nm) irradiation for 18 hours at r.t.

^[a]^ Yield determined by ^1^H‐NMR. Yield of **1** after purification by column chromatography in parenthesis.

^[b]^ The reaction was performed without CuI.

Among the light sources utilized, LED 427 nm afforded the highest yield, with similarly high yields observed under irradiation at nearby wavelengths, including 440, 456, or 467 nm (for details, see Table S2, Supporting Information).^[^ ^28^ ^]^ A subsequent study focused on determining the most suitable copper salt.^[^ ^28^ ^]^ Although a number of copper salts were employed, CuI led to the highest yield (78% isolated yield) under 427 nm LED irradiation. Other Cu(I) halides, such as CuCl or CuBr, also delivered relatively high yields, though still inferior to CuI. All other copper‐based compounds delivered inferior or no results, with Cu_2_O, Cu(OAc)_2_, and CuOAc affording only moderate to low yields, while all tested Cu(II) salts were completely inactive under the reaction conditions (for details, see Table S3, Supporting Information).^[^ ^28^ ^]^ Further optimization studies were carried out to explore the role of the solvent (for details, see Table S4, Supporting Information).^[^ ^28^ ^]^ Acetonitrile was found to afford the highest yield, whereas the use of less polar solvents, such as *N,N*‐dimethylformamide, ethyl acetate, or toluene, led to moderate to low conversions. Increasing the concentration of ACN resulted in a significant drop in the yield. In contrast, increasing the solvent volume had minimal impact on the outcome, as similar yields were obtained. Highly polar solvents, such as water, methanol, or dimethylsulfoxide were found to be incompatible with the existing protocol. Moreover, chlorinated solvents, such as CHCl_3_ or CH_2_Cl_2_, proved to be unsuitable under these conditions.

Additional experiments were conducted to validate the reaction conditions and to explore the optimal stoichiometry of the reagents (Table 2). The reaction does not proceed efficiently in the absence of 1,8‐dihydroxyanthraquinone, copper(I) iodide, light irradiation, or under an argon atmosphere, indicating that the presence of oxygen is essential (Table 2, entries 1–5), with slightly improved results observed under open‐air conditions compared to an oxygen atmosphere.^[^ ^28^ ^]^ A reduced organocatalyst loading resulted in a 10% decrease in the yield (from the optimal 78% to 68%, Table 2, entries 1 vs. 6), while decreasing the amount of CuI from 15 mol% to 10 mol% afforded the same yield (Table 2, entries 1 vs. 7). However, further reduction of the copper salt loading led to significantly lower yields (Table 2, entries 8 and 9). Final experiments revealed that 18 hours yielded the desired product most efficiently, as shorter durations of 6 and 9 hours produced significantly lower yields (Table 2, entries 10 and 11).

**Table 2:** Study of reaction parameters ‐ catalyst loading and additive loading screening.

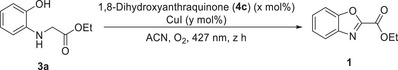				
Entry	Catalyst Loading (mol%)	Additive Loading (mol%)	Reaction Time (h)	Yield (%)^[^ ^a^ ^]^
1	20	15	18	89 (78)
2	‐	15	18	28
3	20	‐	18	34
4^[^ ^b^ ^]^	20	15	18	7
5^[^ ^c^ ^]^	20	15	18	22
6	15	15	18	84 (68)
**7**	**20**	**10**	**18**	**85 (78)**
8	20	5	18	82 (58)
9	20	2.5	18	81 (48)
10	20	10	6	66 (52)
11	20	10	9	82 (65)

Reaction conditions: **3a** (39 mg, 0.20 mmol, 1.00 equiv.), 1,8‐dihydroxyanthraquinone (**4c**) (x mol%) and CuI (y mol%) in ACN (2 mL), under air and LED (Kessil PR160L, 427 nm) irradiation for z h at r.t.

^[a]^ Yield determined by ^1^H‐NMR. Yield of **1** after purification by column chromatography in parenthesis.

^[b]^ The reaction was performed under dark.

^[c]^ The reaction was performed under argon.

To investigate the substrate scope of this new photochemical protocol, we initially synthesized a series of glycine derivatives (for details, see Supporting Information).^[^ ^28^ ^]^ 2‐Bromoacetates and 2‐bromoacetamides, required as starting materials, were synthesized via coupling of 2‐bromoacetic acid with a variety of alcohols and via acylation of primary amines with 2‐bromoacetyl chloride, respectively. Subsequently, the starting materials **3a**‐**3aa** were obtained through the reaction of 2‐aminophenol and its derivatives with bromoacetates and bromoacetamides in the presence of potassium fluoride.^[^ ^28^ ^]^ Alternatively, glycine‐derived amide substrates **3ab‐af** were synthesized in moderate to low yields via hydrolysis of the *tert*‐butyl ester **3** **h** to the corresponding acid, followed by coupling with the appropriate amine.

**Scheme 2:**
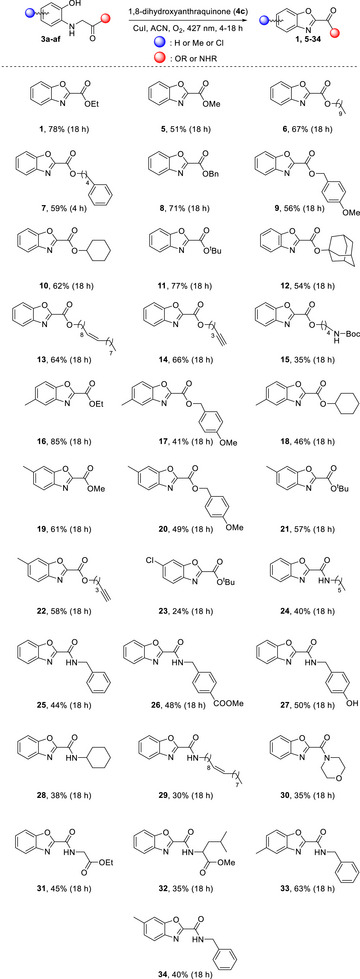
Substrate scope for the photochemical cyclization protocol.

Having established the optimum reaction conditions, we proceeded to the application of the protocol to a wide variety of substrates, and the results are summarized in Scheme 2. Notably, esters were found to perform superiorly in terms of yield compared to amide counterparts, suggesting a higher reactivity and cyclization propensity of ester substrates under photocatalytic conditions. We explored various aliphatic ester substrates, and benzoxazole 2‐carboxylates **1** and **5–7** were isolated in moderate to high yields, ranging from 51% to 78%. Ethyl ester **1** was isolated in 78% yield, while methyl ester **5** in 51% yield. A substrate with a longer decanoyl chain led to **6** in 67% yield, while a substrate featuring a phenyl ring at the terminal position of the alkyl chain furnished benzoxazole **7** in 59% yield. Additionally, benzyl ester derivative **8** was obtained in 71% yield. To probe the electronic influence of substituted benzyl esters, a *p*‐methoxybenzyl (PMB) derivative was evaluated, yielding the corresponding product **9** in 56%, slightly lower than the parent benzyl ester. Furthermore, a cyclohexyl‐substituted ester afforded product **10** in 62% yield, showing that even esters carrying bulkier aliphatic groups are tolerated, albeit with a slight reduction in efficiency relative to linear alkyl esters. In the same vein, *tert*‐butyl ester proved to be among the most reactive substrates, delivering the corresponding benzoxazole **11** in an excellent yield (77%). Conversely, when the ester derivatives contained bulkier groups, such as the adamantyl moiety, or more sensitive substituents, such as the Boc (*tert*‐butoxycarbonyl) group, a reduction in yield was observed, affording compounds **12** and **15** in lower yields (54% and 35%, respectively). Esters bearing double or triple bonds reacted efficiently, producing compounds **13** and **14** in 64% and 66% yields, respectively. The electronic nature of substituents on the aromatic ring of the glycine derivative was also systematically varied to probe their effect on reaction efficiency. Specifically, the incorporation of electron‐donating groups (EDGs), such as methyl substituents, enhanced the yield substantially in some cases. For instance, introducing a methyl group on the benzene ring resulted in an increased yield (85%) for compound **16**, compared to the unsubstituted counterpart **1**. A similar trend was observed with the methyl‐substituted compound **19**, which was obtained in a higher yield (61%) compared to compound **5**. This trend is consistent with the hypothesis that EDGs increase the nucleophilicity of the phenol moiety and stabilize radical intermediates, thereby facilitating more efficient cyclization. Additionally, we extended the scope of the reaction to include a variety of ester derivatives, such as the methyl‐substituted methoxybenzyl esters **17** and **20**, cyclohexyl ester **18**, *tert*‐butyl ester **21**, and the corresponding alkyne analogue **22**. In all these cases, the reaction proceeded smoothly and delivered comparable yields ranging from 41% to 58%, which were not significantly different from those obtained with the nonsubstituted compounds. Replacing the methyl group on the benzene ring with a chlorine atom resulted in the formation of compound **23**, albeit in a reduced yield (24%).

Subsequent efforts focused on the amide counterparts, where a general decrease in yield was noted. Application of the protocol to the hexyl amide derivative afforded compound **24** in 40% yield, while benzamide **25** was obtained in a slightly higher yield (44%). To investigate the influence of electron‐withdrawing groups (EWGs), structural modifications were introduced on the aromatic ring. Incorporation of a methoxycarbonyl substituent (–COOCH_3_) resulted in a modest improvement, yielding compound **26** in 48%. Although the enhancement was limited, it suggests a potential stabilizing effect of EWGs on reactive intermediates. Further substitution with a hydroxyl group led to the formation of compound **27** in a slightly improved yield (50%). In contrast, benzamides bearing bulkier or more complex substituents, namely cyclohexyl (**28**), oleyl (**29**), or morpholine (**30**) moieties, were isolated in lower yields (38%, 30%, and 35%, respectively). The synthetic protocol was further extended to the synthesis of benzoxazole 2‐carboxamides **31** and **32**, carrying a glycine or a leucine residue, respectively. These transformations afforded the desired products in moderate yields (45% for **31** and 35% for **32**). A methyl group was once again introduced, leading to the formation of benzyl amides **33** and **34**. Interestingly, these modifications resulted in significantly improved yields (63% and 40%, respectively). This observation is consistent with a broader trend identified in the study, wherein alkyl substituents, particularly when positioned *para* to the oxygen atom of the benzoxazole or benzamide core, appear to enhance reactivity and improve product yields.

**Scheme 3:**
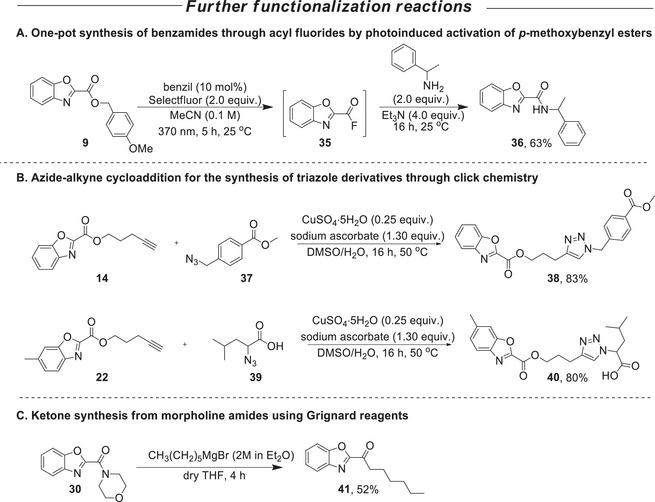
Functionalization reactions involving benzoxazole derivatives.

Finally, we aimed at incorporating tandem transformations into selected benzoxazole 2‐carboxylates/carboxamides produced by our synthetic photochemical protocol, thereby enabling the synthesis of structurally novel and potentially valuable compounds. Furthermore, the application of diverse reaction conditions to our benzoxazole 2‐carboxylates/carboxamides underscores their utility as versatile intermediates for the synthesis of a wide range of compounds, thereby further highlighting the value and broader applicability of our photochemical protocol (Scheme 3). First, we selected compound **9** for a metal‐free, photoinduced activation of *p*‐methoxybenzyl ester, employing Selectfluor and benzil, to generate acyl fluoride intermediates (Scheme 3, **A**). Following the conditions reported by Lee and Maruoka,^[^ ^29^ ^]^ and taking into account the challenges associated with isolating acyl fluorides, we evaluated the chemical yield based on the formation of the corresponding amide **36** (Scheme 3, **A**). This was achieved via in situ treatment of compound **35** with 1‐phenylethylamine and triethylamine, resulting in a 63% yield of the amide derivative **36**. In the subsequent reactions, we established a pathway for the synthesis of hybrid compounds incorporating a triazole ring (Scheme 3, **B**). Specifically, benzoxazole 2‐carboxylates **14** and **22** were subjected to click reaction conditions with azides **37** and **39**, respectively, following the conditions described by Suzuki and coworkers^[^ ^30^ ^]^ to afford compounds **38** and **40**. These results confirm that benzoxazole derivatives are compatible with such conditions, thereby facilitating access to triazole‐containing structures, which are frequently associated with biologically active and medicinally relevant compounds.^[^ ^31^ ^]^ Finally, benzoxazole 2‐carboxamide **30**, bearing a morpholine amide moiety, was treated with hexylmagnesium bromide, resulting in the formation of ketone **41** (Scheme 3, **C**). This transformation is consistent with the known reactivity of morpholine amides toward Grignard reagents,^[^ ^32^, ^33^, ^34^, ^35^ ^]^ and leads to 2‐substituted benzoxazole ketones.

**Scheme 4:**
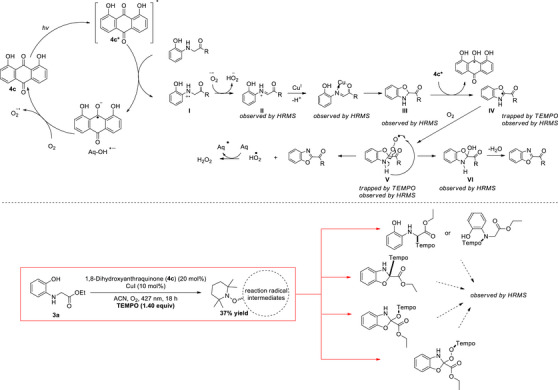
Proposed mechanism for the photochemical cyclization of glycine derivatives to benzoxazole 2‐carboxylates/carboxamides and radical trapping experiments.

A plausible mechanism for the photochemical cyclization of glycine derivatives using 1,8‐dihydroxyanthraquinone (**4c**) (DHAQ) and Cu(I) involves a cooperative catalytic system induced by visible light and molecular oxygen (Scheme 4). Under light irradiation, DHAQ is excited to its reactive photoexcited state (DHAQ*) (4c*), which initiates a single‐electron transfer (SET) with the glycine derivative, generating radical cation **I** and the reduced form of DHAQ (Aq‐OH^•–^). Aq‐OH^•–^ can react with O_2_, leading to O_2_^•–^ and regeneration of **4c**. Radical cation **I** is then oxidized by superoxide (O_2_^•–^), to produce imine intermediate **II**. In the presence of Cu(I), **II** is further activated—possibly forming an electrophilic iminium intermediate—which undergoes intramolecular nucleophilic attack by the *ortho*‐hydroxy group of the aromatic ring, resulting in cyclization to **III**. Following this, interaction between cyclized intermediate **III** and 4c* can generate the new radical species **IV** via a hydrogen atom transfer (HAT) event. Reaction of **IV** with molecular oxygen forms peroxy radical **V**, which facilitates oxidative aromatization to yield the final benzoxazole product and a hydroperoxyl radical (HO_2_^•^). The latter can potentially be converted to hydrogen peroxide in the presence of the catalyst. Intermediate **V** can also collapse to hydroxylated compound **VI**, which upon dehydration may likewise yield the final product. To further support the proposed reaction mechanism, we conducted Direct Infusion‐High Resolution Mass Spectrometry (DI‐HRMS) analysis,^[^ ^36^, ^37^, ^38^, ^39^, ^40^, ^41^, ^42^, ^43^ ^]^ which confirmed the formation of key intermediates, including the imine species, copper‐bound complexes, and both peroxy and hydroxy intermediates.^[^ ^28^ ^]^ To further investigate the involvement of radical intermediates, we carried out a key control experiment using **3a** in the presence of TEMPO, a well‐known radical scavenger. HRMS revealed the formation of TEMPO‐trapped adducts, indicating the presence of transient radical species during the reaction. Additionally, the reaction yield dropped significantly to 37%, supporting the notion that radical quenching by TEMPO inhibits the reaction pathway, thereby confirming the radical nature of the transformation.

## 3 Conclusion

In conclusion, we have developed an efficient photochemical protocol for the synthesis of benzoxazole 2‐carboxylates/carboxamides. A dual system, consisting of the organocatalyst 1,8‐dihydroxyanthraquinone and CuI as an additive, efficiently catalyzes the cyclization of glycine derivatives under irradiation with Kessil LED 427 nm. The method is mild and in line with sustainability, employing a cheap commercially available organocatalyst and a copper salt, avoiding any precious metal. DI‐HRMS analysis provided experimental evidence about the intermediates involved in the catalytic mechanism. Furthermore, we demonstrate that benzoxazole 2‐carboxylates/carboxamides may serve as intermediates for the synthesis of structurally novel and potentially valuable compounds.

## Supporting Information

The authors have cited additional references within the Supporting Information.^[^ ^44^, ^45^, ^46^, ^47^, ^48^, ^49^, ^50^, ^51^, ^52^, ^53^, ^54^, ^55^, ^56^, ^57^ ^]^

## Conflict of Interest

The authors declare no conflict of interest.

## Supporting information



Supporting Information

## Data Availability

The data that support the findings of this study are available in the supplementary material of this article.
