# Supplementary material for: A Sustainable Visible Light‐Mediated Synthesis of Benzoxazole 2‐Carboxylates/Carboxamides
Source: Chemistry. 2025 Nov 2;31(68):e02901. doi: 10.1002/chem.202502901 (PMC12679339; doi:10.1002/chem.202502901)
Supplement: Supplementary file 1 — Supporting Information [file CHEM-31-e02901-s001.pdf]

Chemistry - A European Journal

Supporting Information

A Sustainable Visible Light-Mediated Synthesis of Benzoxazole 2-Carboxylates/carboxamides

Anna-Dimitra D. Gerogiannopoulou,[a,‡] Olga G. Mountanea,[a,‡] Maria A. Theodoropoulou,[a] Christoforos G. Kokotos[a*] and George Kokotos[a*]

[a] Laboratory of Organic Chemistry, Department of Chemistry, National and Kapodistrian University of Athens, Panepistimiopolis, Athens 15771, Greece

	**Page**
**General Remarks**	**S3**
**Optimization of the Reaction Conditions**	**S4**
**General Procedure for the Photochemical Synthesis of Benzoxazoles**	**S12**
**Syntheses of Bromoacetates, Bromoacetamides and Carbamates**	**S25**
**General Procedure for the Synthesis of Glycine Derivatives Using Potassium Fluoride**	**S29**
**General Procedure for the Synthesis of Glycine Derivatives Using Coupling Reagents**	**S40**
**Further Functionalization of Benzoxazole Derivatives**	**S43**
**Direct Infusion-High Resolution Mass Spectrometry (DI-HRMS) Mechanistic Studies**	**S47**
**References**	**S59**
**NMR Traces**	**S60**

General Remarks

Chromatographic purification of products was accomplished using forced-flow chromatography on Merck® Kieselgel 60 230-400 mesh. Thin-layer chromatography (TLC) was performed on aluminum backed silica plates (0.2 mm, 60 F_{254}). Visualization of the developed chromatogram was performed by fluorescence quenching using phosphomolybdic acid. Melting points were determined on a Buchi® 530 hot stage apparatus and are uncorrected. Mass spectra (ESI) were recorded on a Finningan® Surveyor MSQ LC-MS spectrometer. HRMS spectra were recorded on Bruker® Maxis Impact QTOF spectrometer. ^{1}H NMR and ^{13}C NMR spectra were recorded on an Avance III HD Bruker 400 MHz (400 MHz and 100 MHz, respectively) or a Bruker Avance NEO 500 MHz (500 MHz and 125 MHz, respectively) system and are internally referenced to residual solvent signals. Data for ^{1}H NMR are reported as follows: chemical shift (*δ* ppm), integration, multiplicity (s = singlet, d = doublet, t = triplet, q = quartet, quin = quintet, m = multiplet, br s = broad signal, br m = broad multiplet), coupling constant and assignment. Data for ^{13}C-NMR are reported in terms of chemical shift (δ ppm). For all experiments, the intensity of the Kessil lamps was controlled in the maximum level with power consumption: 370 nm (max 43W), 370nm 2nd Gen (max 44W), 390 nm (max 52W), 427 nm & 440 nm (max 45W), 456 nm (max 50W), 467 nm (max 44W) and 525 nm (max 44W).

Optimization of the Reaction Conditions

Catalyst Screening

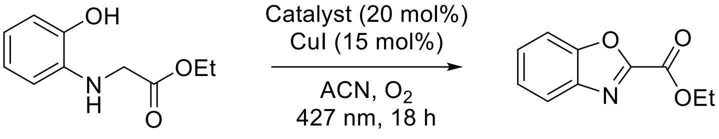

Entry	Catalyst	Yield (%)[a]
1	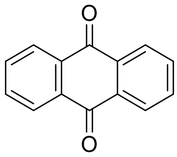 Anthraquinone (**4a**)	93 (72)
2[b]	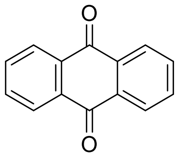 Anthraquinone (**4a**)	58 (37)
3	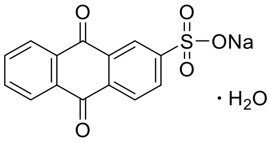 Anthraquinone sulfonic acid sodium salt hydrate (**4b**)	44 (36)
4	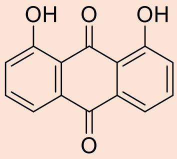 1,8-Dihydroxyanthraquinone (**4c**)	89 (78)
5	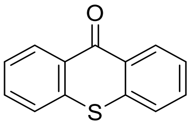 Thioxanthen-9-one (**4d**)	68 (47)
6	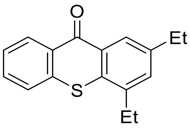 2,4-Diethyl-9*H*-thioxanthen-9-one (**4e**)	91 (68)

**7**	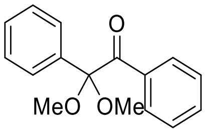 2,2-Dimethoxy-2-phenylacetophenone (**4f**)	14 (18)
**8**	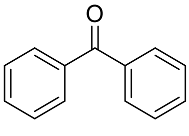 Benzophenone (**4g**)	38 (28)
**9**	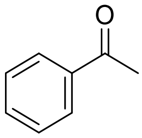 Acetophenone (**4h**)	31 (23)
**10**	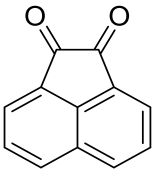 Acenaphthenequinone (**4i**)	83 (63)
**11**	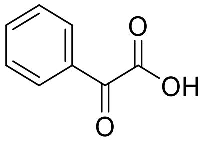 Phenylglyoxylic acid (**4j**)	27

Table S1: Reaction conditions: Ethyl 2-((2-hydroxyphenyl)amino)acetate (**3a**) (39 mg, 0.20 mmol, 1.00 equiv.), catalyst (0.04 mmol, 0.20 equiv.) and CuI (5.7 mg, 0.03 mmol, 0.15 equiv.) in ACN (2 mL), under air and LED (Kessil PR160L, 427 nm) irradiation for 18 h at r.t.. [a] Yield determined by ^{1}H-NMR. Yield of **1** after purification by column chromatography in parenthesis. [b] The reaction was performed without CuI.

Light Source Screening

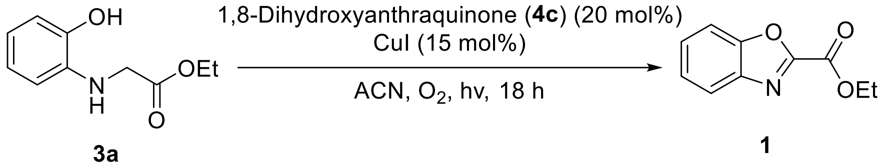

Entry	Light Source	Yield (%)[a]
**1**	370	27
**2**	370 (2nd Gen)	39
**3**	390	45
**4**	427	89 (78)
**5**	440	79 (66)
**6**	456	89 (77)
**7**	467	87 (60)
**8**	525	13
**9**	CFL	5

Table S2: Reaction conditions: Ethyl 2-((2-hydroxyphenyl)amino)acetate (**3a**) (39 mg, 0.20 mmol, 1.00 equiv.), 1,8-dihydroxyanthraquinone (**4c**) (9.6 mg, 0.04 mmol, 0.20 equiv.) and CuI (5.7 mg, 0.03 mmol, 0.15 equiv.) in ACN (2 mL), under air and irradiation for 18 h at r.t.. [a] Yield determined by ^{1}H-NMR. Yield of **1** after purification by column chromatography in parenthesis.

Additive Screening

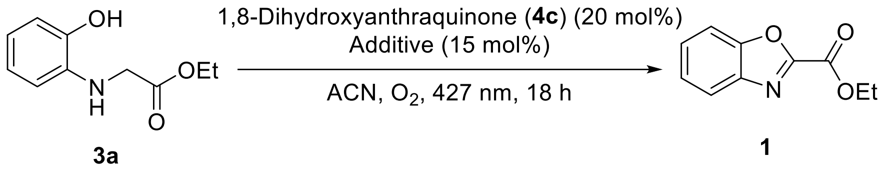

Entry	Additive	Yield (%)[a]
**1**	CuI	89 (78)
**2**	CuBr	72 (67)
**3**	CuCl	76 (63)
**4**	Cu_2O	69 (37)
**5**	CuOAc	41 (27)
**6**	$Cu(OAc)_2$	71 (21)
**7**	$CuBr_2$	-
**8**	$CuCl_2$	-
**9**	$Cu(TFA)_2$•xH_2O	-

Table S3: Reaction conditions: Ethyl 2-((2-hydroxyphenyl)amino)acetate (**3a**) (39 mg, 0.20 mmol, 1.00 equiv.), 1,8-dihydroxyanthraquinone (**4c**) (9.6 mg, 0.04 mmol, 0.20 equiv.) and additive (0.03 mmol, 0.15 equiv.) in ACN (2 mL), under air and LED (Kessil PR160L, 427 nm) irradiation for 18 h at r.t.. [a] Yield determined by ^{1}H-NMR. Yield of **1** after purification by column chromatography in parenthesis.

Solvent Screening

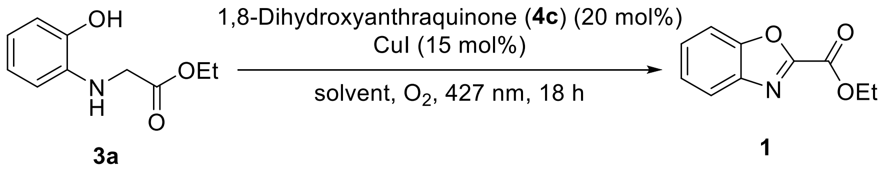

Entry	Solvent	Yield (%)[a]
1	ACN	89 (78)
2[b]	ACN	53 (37)
3[c]	ACN	85 (76)
4	DMF	100 (60)
5	Toluene	76 (37)
6	EtOAc	76 (39)
7	DMSO	-
8	H_2O	-
9	MeOH	-
10	$CHCl_3$	-
11	CH_2Cl_2	-

Table S4: Reaction conditions: Ethyl 2-((2-hydroxyphenyl)amino)acetate (**3a**) (39 mg, 0.20 mmol, 1.00 equiv.), 1,8-dihydroxyanthraquinone (**4c**) (9.6 mg, 0.04 mmol, 0.20 equiv.) and CuI (5.7 mg, 0.03 mmol, 0.15 equiv.) in solvent (2 mL), under air and LED (Kessil PR160L, 427 nm) irradiation for 18 h at r.t.. [a] Yield determined by ^{1}H-NMR. Yield of **1** after purification by column chromatography in parenthesis. [b] Solvent concentration 0.2 M. [c] Solvent concentration 0.05 M.

Study of Reaction Parameters

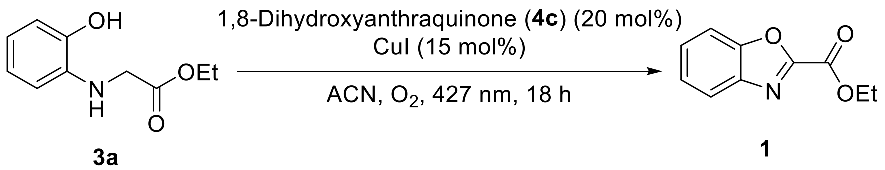

Entry	Catalyst Loading (mol%)	Additive Loading (mol%)	Yield (%)[a]
**1**	20	15	89
**2**	-	15	28
**3**	20	-	34
**4**[b]	20	15	7
**5**[c]	20	15	22

Table S5: Reaction conditions: Ethyl 2-((2-hydroxyphenyl)amino)acetate (**3a**) (39 mg, 0.20 mmol, 1.00 equiv.), 1,8-dihydroxyanthraquinone (**4c**) (9.6 mg, 0.04 mmol, 0.20 equiv.) and CuI (5.7 mg, 0.03 mmol, 0.15 equiv.) in ACN (2 mL), under air and LED (Kessil PR160L, 427 nm) irradiation for 18 h at r.t.. [a] Yield determined by ^{1}H-NMR. [b] The reaction was performed under dark. [c] The reaction was performed under argon.

Catalyst Loading and Additive Loading Screening

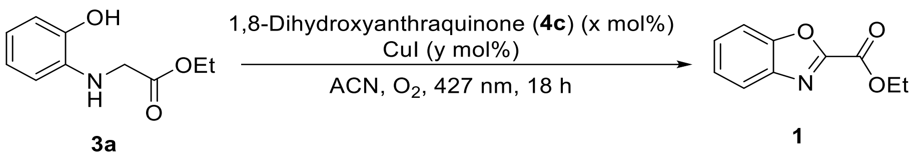

Entry	Catalyst Loading (mol%)	Additive Loading (mol%)	Yield (%)[a]
**1**	15	15	84 (68)
**2**	20	15	89 (78)
**3**	20	10	85 (78)
**4**[b]	20	10	78 (64)
**5**	20	5	82 (58)
**6**	20	2.5	81 (48)

Table S6: Reaction conditions: Ethyl 2-((2-hydroxyphenyl)amino)acetate (**3a**) (39 mg, 0.20 mmol, 1.00 equiv.), 1,8-dihydroxyanthraquinone (**4c**) (x mol%) and CuI (y mol%) in ACN (2 mL), under air and LED (Kessil PR160L, 427 nm) irradiation for 18 h at r.t.. [a] Yield determined by ^{1}H-NMR. Yield of **1** after purification by column chromatography in parenthesis. [b] The reaction was performed under an oxygen atmosphere.

Time Reaction Screening

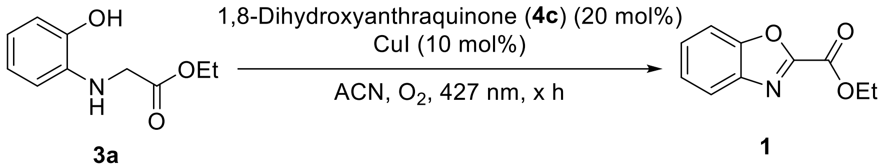

**Entry**	**Time Reaction (h)**	**Yield (%)**[a]
**1**	6	66 (52)
**2**	9	82 (65)
**3**	18	85 (78)

Table S7: Reaction conditions: Ethyl 2-((2-hydroxyphenyl)amino)acetate (**3a**) (39 mg, 0.20 mmol, 1.00 equiv.), 1,8-dihydroxyanthraquinone (**4c**) (9.6 mg, 0.04 mmol, 0.20 equiv.) and CuI (3.8 mg, 0.02 mmol, 0.10 equiv.) in ACN (2 mL), under air and LED (Kessil PR160L, 427 nm) irradiation for x h at r.t.. [a] Yield determined by ^{1}H-NMR. Yield of **1** after purification by column chromatography in parenthesis.

General Procedure for the Photochemical Synthesis of Benzoxazoles

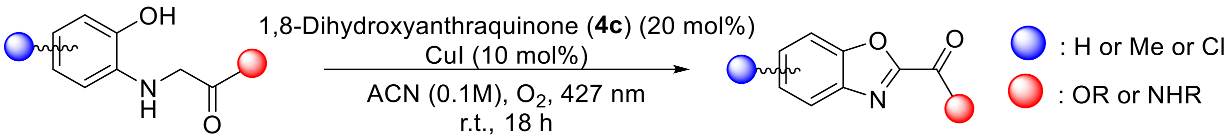

To a solution of the glycine derivative (0.20 mmol, 1.00 equiv.) in acetonitrile (2 mL), CuI (3.8 mg, 0.02 mmol, 0.10 equiv.) and 1,8-dihydroxyanthraquinone (**4c**) (9.6 mg, 0.04 mmol, 0.20 equiv.) were added. The reaction mixture was stirred at room temperature for 18 h (unless otherwise noted) under air and LED (Kessil PR160L, 427 nm) irradiation. Upon completion of the reaction, the reaction mixture was concentrated under reduced pressure and the resulting residue was purified by flash column chromatography (Pet. Ether:EtOAc 80:20 to 60:40).

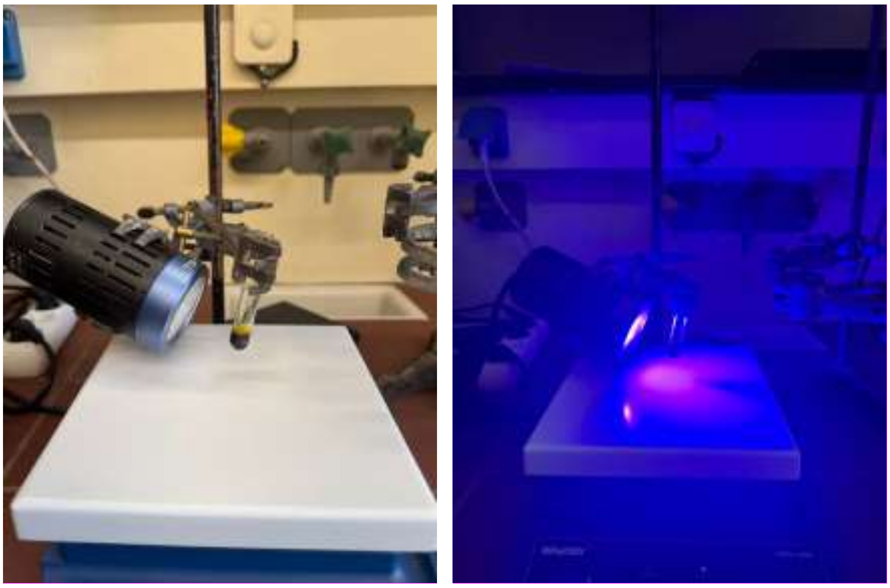

Figure S1. A: Reaction mixture setup, **B**: Reaction mixture during irradiation. The reaction tube was placed 4 cm away from the lamp.

Ethyl benzo[*d*]oxazole-2-carboxylate (1)[1]

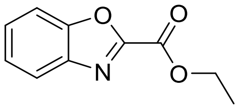

Yellow solid; Yield: **78%** (18 h); **m.p.** 93-95 °C (lit. m.p.: 93.2-95.1 °C); **^{1}H NMR (400 MHz, CDCl$_3$)** δ 7.88 (1H, d, *J* = 7.9 Hz, ArH), 7.65 (1H, d, *J* = 7.9 Hz, ArH), 7.51 (1H, t, *J* = 7.9 Hz, ArH), 7.44 (1H, t, *J* = 7.9 Hz, ArH), 4.54 (2H, q, *J* = 6.7 Hz, OCH_2), 1.48 (3H, t, *J* = 6.7 Hz, CH_3); **^{13}C NMR (100 MHz, CDCl$_3$)** δ 156.6, 152.9, 151.0, 140.6, 128.2, 125.9, 122.2, 111.8, 63.4, 14.3; **HRMS (ESI$^+$):** *m/z* calcd for $C_{10}H_{10}NO_3^+$: 192.0655; $[M+H]^+$ found: 192.0655.

Methyl benzo[*d*]oxazole-2-carboxylate (5)[2]

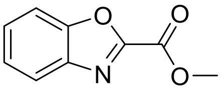

White solid; Yield: **51%** (18 h); **m.p.** 90-92 °C (lit. m.p.: 95.2-97.2 °C); **^{1}H NMR (400 MHz, CDCl$_3$)** δ 7.88 (1H, d, *J* = 8.0 Hz, ArH), 7.65 (1H, d, *J* = 8.0 Hz, ArH), 7.52 (1H, t, *J* = 8.0 Hz, ArH), 7.45 (1H, t, *J* = 8.0 Hz, ArH), 4.08 (3H, s, OCH_3); **^{13}C NMR (100 MHz, CDCl$_3$)** δ 157.0, 152.6, 151.0, 140.6, 128.4, 125.9, 122.3, 111.9, 53.8; **HRMS (ESI$^+$):** *m/z* calcd for $C_9H_8NO_3^+$: 178.0499; $[M+H]^+$ found: 178.0496.

Decyl benzo[*d*]oxazole-2-carboxylate (6)

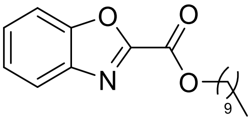

Yellow solid; Yield: **67%** (18 h); **m.p.** 37-38 °C; **^{1}H NMR (400 MHz, CDCl$_3$)** δ 7.89 (1H, d, *J* = 8.0 Hz, ArH), 7.65 (1H, d, *J* = 8.0 Hz, ArH), 7.51 (1H, t, *J* = 8.0 Hz, ArH), 7.44 (1H, t, *J* = 8.0 Hz, ArH), 4.48 (2H, t, *J* = 6.8 Hz, OCH_2), 1.85 (2H, quin, *J* = 6.8 Hz, CH_2), 1.49-1.40 (2H, m, CH_2), 1.38-1.21 (12H, m, 6 x CH_2), 0.86 (3H, t, *J* = 6.2 Hz, CH_3); **^{13}C NMR (100 MHz, CDCl$_3$)** δ 156.6, 152.8, 150.9, 140.5, 128.1, 125.7, 122.1, 111.7, 67.3, 31.8, 29.5, 29.4, 29.2, 29.2, 28.5, 25.7, 22.6, 14.0; **HRMS (ESI$^+$):** *m/z* calcd for $C_{18}H_{25}NNaO_3^+$: 326.1727; $[M+Na]^+$ found: 326.1728.

4-Phenylbutyl benzo[*d*]oxazole-2-carboxylate (7)

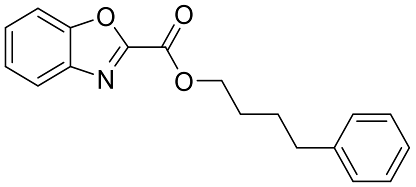

Yellowish low melting ponting solid; Yield: **59%** (4 h); **^{1}H NMR (400 MHz, CDCl$_3$)** δ 7.90 (1H, d, J = 8.0 Hz, ArH), 7.66 (1H, d, J = 8.0 Hz, ArH), 7.53 (1H, t, J = 8.0 Hz, ArH), 7.45 (1H, t, J = 8.0 Hz, ArH), 7.30-7.26 (2H, m, ArH), 7.22-7.16 (3H, m, ArH), 4.51 (2H, t, J = 6.6 Hz, OCH$_2$), 2.70 (2H, t, J = 7.5 Hz, CH$_2$Ph), 1.94-1.86 (2H, m, CH$_2$), 1.85-1.77 (2H, m, CH$_2$); **^{13}C NMR (100 MHz, CDCl$_3$)** δ 156.7, 152.9, 151.1, 141.9, 140.7, 128.6, 128.5, 128.3, 126.1, 125.9, 122.3, 111.9, 67.2, 35.5, 28.2, 27.7; **HRMS (ESI$^+$):** *m/z* calcd for $C_{18}H_{18}NO_3^+$: 296.1281; [M+H]$^+$ found: 296.1281.

Benzyl benzo[*d*]oxazole-2-carboxylate (8)[2]

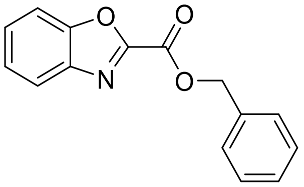

Orange solid; Yield: **71%** (18 h); **m.p.** 86-88 °C (lit. m.p.: 91.1-93.7 °C); **^{1}H NMR (400 MHz, CDCl$_3$)** δ 7.89 (1H, d, J = 8.0 Hz, ArH), 7.65 (1H, d, J = 8.0 Hz, ArH), 7.55-7.49 (3H, m, ArH), 7.47-7.35 (4H, m, ArH), 5.51 (2H, s, OCH$_2$); **^{13}C NMR (100 MHz, CDCl$_3$)** δ 156.5, 152.8, 151.1, 140.7, 134.6, 129.1, 129.1, 128.9, 128.4, 125.9, 122.3, 111.9, 68.8; **HRMS (ESI$^+$):** *m/z* calcd for $C_{15}H_{11}NNaO_3^+$: 276.0631; [M+Na]$^+$ found: 276.0631.

4-Methoxybenzyl benzo[*d*]oxazole-2-carboxylate (9)

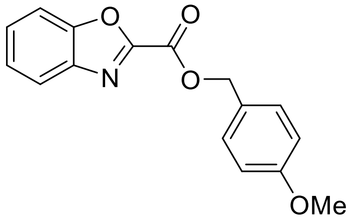

Yellowish solid; Yield: **56%** (18 h); **m.p.** 154-156 °C; **^{1}H NMR (400 MHz, CDCl$_3$)** δ 7.86 (1H, d, J = 8.0 Hz, ArH), 7.62 (1H, d, J = 8.0 Hz, ArH), 7.54-7.39 (4H, m, ArH), 6.91 (2H, d,

J = 8.0 Hz, ArH), 5.44 (2H, m, OCH_2), 3.80 (3H, s, OCH_3); **^{13}C NMR (100 MHz, $CDCl_3$)** *δ* 160.1, 156.4, 152.7, 150.8, 140.5, 130.9, 128.1, 126.5, 125.7, 122.1, 114.1, 111.7, 68.5, 55.3; **HRMS (ESI$^+$):** *m/z* calcd for $C_{16}H_{13}NNaO_4^+$: 306.0737; $[M+Na]^+$ found: 306.0736.

Cyclohexyl benzo[*d*]oxazole-2-carboxylate (10)

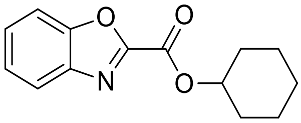

Yellow solid; Yield: **62%** (18 h); **m.p.** 70-72 ^{o}C; **^{1}H NMR (400 MHz, $CDCl_3$)** *δ* 7.89 (1H, d, *J* = 8.0 Hz, ArH), 7.65 (1H, d, *J* = 8.0 Hz, ArH), 7.50 (1H, t, *J* = 8.0 Hz, ArH), 7.43 (1H, t, *J* = 8.0 Hz, ArH), 5.18-5.10 (1H, m, OCH), 2.11-2.02 (2H, m, 2 x C*H*H), 1.89-1.80 (2H, m, 2 x C*H*H), 1.72-1.58 (3H, m, 3 x C*H*H), 1.49-1.37 (2H, m, 2 x C*H*H), 1.35-1.26 (1H, m, C*H*H); **^{13}C NMR (100 MHz, $CDCl_3$)** *δ* 156.0, 153.1, 150.8, 140.6, 127.9, 125.6, 122.1, 111.7, 76.4, 31.5, 25.1, 23.9; **HRMS (ESI$^+$):** *m/z* calcd for $C_{14}H_{15}NNaO_3^+$: 268.0944; $[M+Na]^+$ found: 268.0946.

tert-Butyl benzo[*d*]oxazole-2-carboxylate (11)[2]

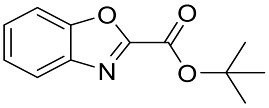

Yellow low melting point solid; Yield: **77%** (18 h); **^{1}H NMR (400 MHz, $CDCl_3$)** *δ* 7.87 (1H, d, *J* = 8.0 Hz, ArH), 7.63 (1H, d, *J* = 8.0 Hz, ArH), 7.49 (1H, t, *J* = 8.0 Hz, ArH), 7.42 (1H, t, *J* = 8.0 Hz, ArH), 1.68 (9H, s, 3 x CH_3); **^{13}C NMR (100 MHz, $CDCl_3$)** *δ* 155.7, 153.8, 150.9, 140.7, 127.9, 125.7, 122.2, 111.8, 85.3, 28.1; **HRMS (ESI$^+$):** *m/z* calcd for $C_{12}H_{13}NNaO_3^+$: 242.0788; $[M+Na]^+$ found: 242.0787.

Adamantan-1-yl benzo[*d*]oxazole-2-carboxylate (12)

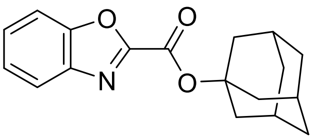

Yellow solid; Yield: **54%** (18 h); **m.p.** 92-94 °C; **^{1}H NMR (400 MHz, CDCl$_3$)** δ 7.88 (1H, d, J = 8.0 Hz, ArH), 7.63 (1H, d, J = 8.0 Hz, ArH), 7.49 (1H, t, J = 8.0 Hz, ArH), 7.42 (1H, t, J = 8.0 Hz, ArH), 2.36-2.32 (6H, m, 6 x C*H*H), 2.29-2.24 (3H, m, 3 x CH), 1.78-1.67 (6H, m, 6 x C*H*H); **^{13}C NMR (100 MHz, CDCl$_3$)** δ 155.1, 153.7, 150.8, 140.6, 127.7, 125.5, 122.0, 111.6, 85.3, 41.2, 36.0, 31.0; **HRMS (ESI$^+$):** *m/z* calcd for $C_{18}H_{19}NNaO_3^+$: 320.1257; $[M+Na]^+$ found: 320.1258.

(*Z*)-Octadec-9-en-1-yl benzo[*d*]oxazole-2-carboxylate (13)

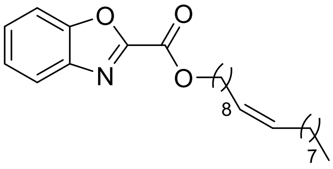

Yellow oil; Yield: **64%** (18 h); **^{1}H NMR (400 MHz, CDCl$_3$)** δ 7.89 (1H, d, J = 8.0 Hz, ArH), 7.65 (1H, d, J = 8.0 Hz, ArH), 7.51 (1H, t, J = 8.0 Hz, ArH), 7.44 (1H, t, J = 8.0 Hz, ArH), 5.47-5.22 (2H, m, 2 x =CH), 4.48 (2H, t, J = 6.7 Hz, OCH_2), 2.08-1.90 (4H, m, 2 x CH_2), 1.89-1.80 (2H, m, CH_2), 1.49-1.41 (2H, m, CH_2), 1.39-1.21 (20H, m, 10 x CH_2), 0.86 (3H, t, J = 6.1 Hz, CH_3); **^{13}C NMR (100 MHz, CDCl$_3$)** δ 156.6, 152.8, 150.9, 140.5, 130.0, 129.7, 128.1, 125.7, 122.1, 111.7, 67.3, 32.6, 31.9, 29.7, 29.7, 29.7, 29.6, 29.5, 29.3, 29.3, 29.1, 28.5, 27.2, 27.1, 25.7, 22.6, 14.1; **HRMS (ESI$^+$):** *m/z* calcd for $C_{26}H_{39}NNaO_3^+$: 436.2822; $[M+Na]^+$ found: 436.2822.

Pent-4-yn-1-yl benzo[*d*]oxazole-2-carboxylate (14)

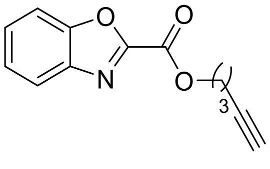

Yellowish solid; Yield: **66%** (18 h); **m.p.** 56-58 °C; **^{1}H NMR (400 MHz, CDCl$_3$)** δ 7.89 (1H, d, J = 8.0 Hz, ArH), 7.65 (1H, d, J = 8.0 Hz, ArH), 7.52 (1H, t, J = 8.0 Hz, ArH), 7.45 (1H, t,

J = 8.0 Hz, ArH), 4.60 (2H, t, *J* = 6.2 Hz, OCH_2), 2.45-2.38 (2H, m, CH_2), 2.08 (2H, quin, *J* = 6.2 Hz, CH_2), 1.99 (1H, s, ≡CH); **^{13}C NMR (100 MHz, $CDCl_3$)** δ 156.5, 152.6, 150.9, 140.5, 128.2, 125.8, 122.1, 111.7, 82.4, 69.5, 65.5, 27.3, 15.2; **HRMS (ESI$^+$):** *m/z* calcd for $C_{13}H_{11}NNaO_3^+$: 252.0631; $[M+Na]^+$ found: 252.0631.

4-((*tert*-Butoxycarbonyl)amino)butyl benzo[*d*]oxazole-2-carboxylate (15)

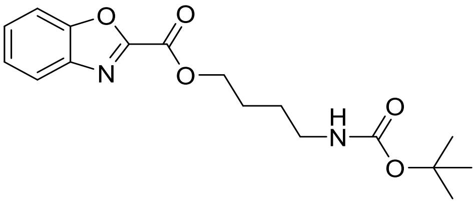

Yellow solid; Yield: **35%** (18 h); **m.p.** 90-92 ^{o}C; **^{1}H NMR (400 MHz, $CDCl_3$)** δ 7.89 (1H, d, *J* = 8.0 Hz, ArH), 7.66 (1H, d, *J* = 8.0 Hz, ArH), 7.52 (1H, t, *J* = 8.0 Hz, ArH), 7.45 (1H, t, *J* = 8.0 Hz, ArH), 4.60 (1H, br s, NH), 4.50 (2H, t, *J* = 7.2 Hz, OCH_2), 3.20 (2H, t, *J* = 7.2 Hz, NCH_2), 1.89 (2H, quin, *J* = 7.2 Hz, CH_2), 1.67 (2H, quin, *J* = 7.2 Hz, CH_2), 1.43 (9H, s, 3 x CH_3); **^{13}C NMR (100 MHz, $CDCl_3$)** δ 156.6, 156.1, 152.8, 151.1, 140.7, 128.3, 125.9, 122.3, 111.9, 79.4, 66.9, 40.3, 28.5, 26.8, 26.0; **HRMS (ESI$^+$):** *m/z* calcd for $C_{17}H_{23}N_2O_5^+$: 335.1601; $[M+H]^+$ found: 335.1601.

Ethyl 5-methylbenzo[*d*]oxazole-2-carboxylate (16)[3]

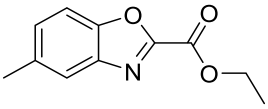

Yellow solid; Yield: **85%** (18 h); **m.p.** 92-93 ^{o}C (lit. m.p.: 97.4-98.6 ^{o}C); **^{1}H NMR (400 MHz, $CDCl_3$)** δ 7.63 (1H, s, ArH), 7.50 (1H, d, *J* = 8.2 Hz, ArH), 7.30 (1H, d, *J* = 8.2 Hz, ArH), 4.53 (2H, q, *J* = 6.7 Hz, OCH_2), 2.47 (3H, s, CH_3), 1.47 (3H, t, *J* = 6.7 Hz, CH_3); **^{13}C NMR (100 MHz, $CDCl_3$)** δ 156.7, 152.9, 149.3, 140.8, 135.9, 129.7, 121.8, 111.2, 63.3, 21.6, 14.3; **HRMS (ESI$^+$):** *m/z* calcd for $C_{11}H_{11}NNaO_3^+$: 228.0631; $[M+Na]^+$ found: 228.0631.

4-Methoxybenzyl 5-methylbenzo[*d*]oxazole-2-carboxylate (17)

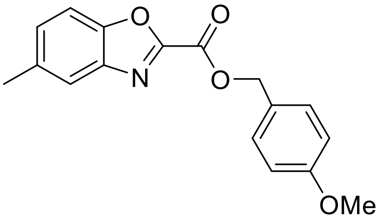

Yellowish solid; Yield: **41%** (18 h); **m.p.** 108-110 °C; **^{1}H NMR (400 MHz, CDCl$_3$)** δ 7.63 (1H, s, ArH), 7.50 (1H, d, J = 8.0 Hz, ArH), 7.46 (2H, d, J = 8.0 Hz, ArH), 7.32 (1H, d, J = 8.0 Hz, ArH), 6.91 (2H, d, J = 8.0 Hz, ArH), 5.44 (2H, m, OCH$_2$), 3.81 (3H, s, OCH$_3$), 2.49 (3H, s, CH$_3$); **^{13}C NMR (100 MHz, CDCl$_3$)** δ 160.1, 156.5, 152.7, 149.1, 140.7, 135.8, 131.0, 129.6, 126.6, 121.6, 114.1, 111.1, 68.5, 55.3, 21.5; **HRMS (ESI$^+$):** *m/z* calcd for $C_{17}H_{15}NNaO_4^+$: 320.0893; [M+Na]$^+$ found: 320.0885.

Cyclohexyl 5-methylbenzo[*d*]oxazole-2-carboxylate (18)

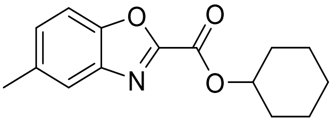

Yellow solid; Yield: **46%** (18 h); **m.p.** 109-110 °C; **^{1}H NMR (400 MHz, CDCl$_3$)** δ 7.65 (1H, s, ArH), 7.52 (1H, d, J = 8.0 Hz, ArH), 7.31 (1H, d, J = 8.0 Hz, ArH), 5.18-5.08 (1H, m, OCH), 2.49 (3H, s, CH$_3$), 2.11-2.02 (2H, m, 2 x C*H*H), 1.90-1.79 (2H, m, 2 x C*H*H), 1.72-1.58 (3H, m, 3 x C*H*H), 1.49-1.37 (2H, m, 2 x C*H*H), 1.34-1.22 (1H, m, C*H*H); **^{13}C NMR (100 MHz, CDCl$_3$)** δ 156.1, 153.1, 149.1, 140.7, 135.7, 129.4, 121.6, 111.0, 76.4, 31.5, 25.1, 23.9, 21.5; **HRMS (ESI$^+$):** *m/z* calcd for $C_{15}H_{17}NNaO_3^+$: 282.1101; [M+Na]$^+$ found: 282.1096.

Methyl 6-methylbenzo[*d*]oxazole-2-carboxylate (19)[4a]

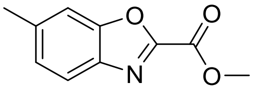

Yellow solid; Yield: **61%** (18 h); **m.p.** 113-115 °C (lit. m.p.: 111-112 °C)[4b]; **^{1}H NMR (400 MHz, CDCl$_3$)** δ 7.76 (1H, d, J = 8.0 Hz, ArH), 7.46 (1H, s, ArH), 7.29 (1H, d, J = 8.0 Hz, ArH), 4.10 (3H, s, OCH$_3$), 2.55 (3H, s, CH$_3$); **^{13}C NMR (100 MHz, CDCl$_3$)** δ 157.0, 152.1,

151.3, 139.2, 138.4, 127.4, 121.4, 111.6, 53.5, 22.0; **HRMS (ESI$^+$):** *m/z* calcd for $C_{10}H_9NNaO_3^+$: 214.0475; [M+Na]$^+$ found: 214.0477.

4-Methoxybenzyl 6-methylbenzo[*d*]oxazole-2-carboxylate (20)

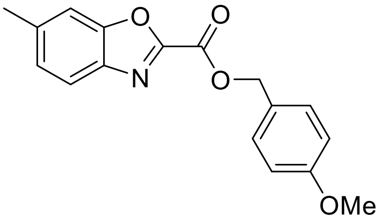

Yellowish solid; Yield: **49%** (18 h); **m.p.** 123-124 °C; **^{1}H NMR (400 MHz, CDCl$_3$)** δ 7.73 (1H, d, J = 8.0 Hz, ArH), 7.51-7.38 (3H, m, ArH), 7.25 (1H, d, J = 8.0 Hz, ArH), 6.91 (2H, d, J = 8.0 Hz, ArH), 5.44 (2H, m, OCH_2), 3.81 (3H, s, OCH_3), 2.52 (3H, s, CH_3); **^{13}C NMR (100 MHz, CDCl$_3$)** δ 160.1, 156.5, 152.2, 151.2, 139.1, 138.4, 131.0, 127.3, 126.6, 121.4, 114.0, 111.5, 68.4, 55.3, 22.0; **HRMS (ESI$^+$):** *m/z* calcd for $C_{17}H_{15}NNaO_4^+$: 320.0893; [M+Na]$^+$ found: 320.0889.

tert-Butyl 6-methylbenzo[*d*]oxazole-2-carboxylate (21)

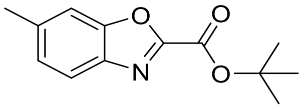

Yellow oil; Yield: **57%** (18 h); **^{1}H NMR (400 MHz, CDCl$_3$)** δ 7.73 (1H, d, J = 8.4 Hz, ArH), 7.41 (1H, s, ArH), 7.23 (1H, d, J = 8.4 Hz, ArH), 2.51 (3H, s, CH_3), 1.67 (9H, s, 3 x CH_3); **^{13}C NMR (100 MHz, CDCl$_3$)** δ 155.7, 153.2, 151.2, 138.7, 138.5, 127.1, 121.3, 111.5, 84.9, 28.0, 22.0; **HRMS (ESI$^+$):** *m/z* calcd for $C_{13}H_{15}NNaO_3^+$: 256.0944; [M+Na]$^+$ found: 256.0944.

Pent-4-yn-1-yl 6-methylbenzo[*d*]oxazole-2-carboxylate (22)

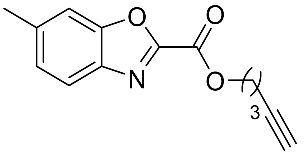

Yellowish solid; Yield: **58%** (18 h); **m.p.** 68-70 °C; **^{1}H NMR (500 MHz, CDCl$_3$)** δ 7.73 (1H, d, J = 8.0 Hz, ArH), 7.43 (1H, s, ArH), 7.25-7.23 (1H, m, ArH), 4.57 (2H, t, J = 6.7 Hz, OCH$_2$), 2.51 (3H, s, CH$_3$), 2.40 (2H, td, J = 6.7 and 2.7 Hz, CH$_2$), 2.06 (2H, quin, J = 6.7 Hz, CH$_2$), 1.97 (1H, t, J = 2.7 Hz, ≡CH); **^{13}C NMR (125 MHz, CDCl$_3$)** δ 156.6, 152.1, 151.2, 139.2, 138.4, 127.4, 121.4, 111.6, 82.5, 69.4, 65.5, 27.3, 22.0, 15.2; **HRMS (ESI$^+$):** *m/z* calcd for $C_{14}H_{13}NNaO_3^+$: 266.0788; [M+H]$^+$ found: 266.0793.

***tert*-Butyl 6-chlorobenzo[*d*]oxazole-2-carboxylate (23)**

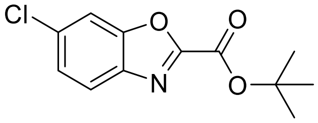

Orange oil; Yield: **24%** (18 h); **^{1}H NMR (400 MHz, CDCl$_3$)** δ 7.79 (1H, d, J = 8.6 Hz, ArH), 7.65 (1H, s, ArH), 7.41 (1H, d, J = 8.6 Hz, ArH), 1.68 (9H, s, 3 x CH$_3$); **^{13}C NMR (100 MHz, CDCl$_3$)** δ 155.1, 154.2, 150.9, 139.3, 133.7, 126.6, 122.6, 112.2, 85.5, 28.0; **HRMS (ESI$^+$):** *m/z* calcd for $C_{12}H_{13}ClNO_3^+$: 254.0578; [M+H]$^+$ found: 254.0577.

***N*-Hexyl benzo[*d*]oxazole-2-carboxamide (24)**

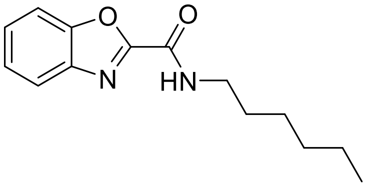

Brown oil; Yield: **40%** (18 h); **^{1}H NMR (400 MHz, CDCl$_3$)** δ 7.78 (1H, d, J = 8.1 Hz, ArH), 7.65 (1H, d, J = 8.1 Hz, ArH), 7.49-7.40 (2H, m, ArH), 7.30 (1H, br s, NH), 3.50 (2H, q, J = 6.8 Hz, NCH$_2$), 1.65 (2H, quin, J = 7.4 Hz, CH$_2$), 1.42-1.28 (6H, m, 3 x CH$_2$), 0.89 (3H, t, J = 6.0 Hz, CH$_3$); **^{13}C NMR (100 MHz, CDCl$_3$)** δ 155.8, 151.3, 140.3, 127.4, 125.6, 121.3, 112.0, 40.1, 31.5, 29.5, 26.7, 22.7, 14.1; **HRMS (ESI$^+$):** *m/z* calcd for $C_{14}H_{18}N_2NaO_2^+$: 269.1260; [M+Na]$^+$ found: 269.1260.

N-Benzyl benzo[d]oxazole-2-carboxamide (25)[1]

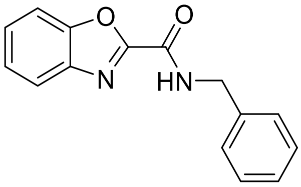

Yellow solid; Yield: **44%** (18 h); **m.p.** 89-91 °C (lit. m.p.: 91.7-93.8 °C); **^{1}H NMR (400 MHz, CDCl$_3$)** δ 7.76 (1H, d, J = 7.9 Hz, ArH), 7.69-7.58 (2H, m, ArH and NH), 7.50-7.29 (7H, m, ArH), 4.69 (2H, d, J = 6.0 Hz, NCH$_2$); **^{13}C NMR (100 MHz, CDCl$_3$)** δ 155.7, 155.5, 151.3, 140.2, 137.1, 129.0, 128.1, 128.1, 127.5, 125.7, 121.3, 112.0, 44.0; **HRMS (ESI$^+$):** *m/z* calcd for $C_{15}H_{12}N_2NaO_2^+$: 275.0791; [M+Na]$^+$ found: 275.0792.

Methyl 4-((benzo[d]oxazole-2-carboxamido)methyl)benzoate (26)

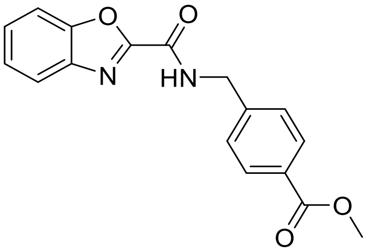

Yellow solid; Yield: **48%** (18 h); **m.p.** 110-112 °C; **^{1}H NMR (400 MHz, CDCl$_3$)** δ 8.01 (2H, d, J = 8.0 Hz, ArH), 7.80-7.22 (2H, m, ArH), 7.65 (1H, d, J = 8.0 Hz, ArH), 7.52-7.39 (4H, m, ArH, and NH), 4.75 (2H, s, NCH$_2$), 3.90 (3H, s, OCH$_3$); **^{13}C NMR (100 MHz, CDCl$_3$)** δ 166.8, 155.9, 155.3, 151.3, 142.3, 140.2, 130.3, 127.8, 127.7, 125.8, 121.4, 112.0, 52.3, 43.5; **HRMS (ESI$^+$):** *m/z* calcd for $C_{17}H_{14}N_2NaO_4^+$: 333.0846; [M+Na]$^+$ found: 333.0846.

N-(4-Hydroxybenzyl)benzo[d]oxazole-2-carboxamide (27)

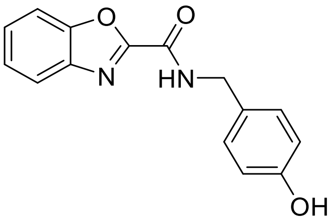

Brown solid; Yield: **50%** (18 h); **m.p.** 126-128 °C; **^{1}H NMR (400 MHz, CD$_3$OD)** δ 7.83 (1H, d, J = 5.8 Hz, ArH), 7.72 (1H, d, J = 5.8 Hz, ArH), 7.57-7.51 (1H, m, ArH), 7.50-7.44

(1H, m, ArH), 7.22 (2H, d, *J* = 5.8 Hz, ArH), 6.76 (2H, d, *J* = 5.8 Hz, ArH), 4.51 (2H, s, NCH_2Ph); **^{13}C NMR (100 MHz, CD_3OD)** δ 158.0, 157.5, 157.0, 152.2, 141.7, 130.3, 130.1, 128.7, 126.7, 122.4, 116.3, 112.6, 43.9; **HRMS (ESI$^+$):** *m/z* calcd for $C_{15}H_{12}N_2NaO_3^+$: 291.0740; $[M+Na]^+$ found: 291.0736.

N-Cyclohexylbenzo[d]oxazole-2-carboxamide (28)[5a]

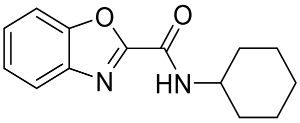

Red solid; Yield: **38%** (18 h); **m.p.** 120-121 °C (lit. m.p.: 127-129 °C)[5b]; **^{1}H NMR (400 MHz, $CDCl_3$)** δ 7.78 (1H, d, *J* = 8.0 Hz, ArH), 7.66 (1H, d, *J* = 8.0 Hz, ArH), 7.50-7.39 (2H, m, ArH), 7.17 (1H, s, NH), 4.08-3.95 (1H, m, NCH), 2.10-2.01 (2H, m, 2 x C*H*H), 1.83-1.75 (2H, m, 2 x C*H*H), 1.70-1.63 (1H, m, C*H*H), 1.50-1.18 (5H, m, 5 x C*H*H); **^{13}C NMR (100 MHz, $CDCl_3$)** δ 155.8, 154.7, 151.1, 140.2, 127.2, 125.5, 121.1, 111.9, 48.9, 32.9, 25.4, 24.7; **HRMS (ESI$^+$):** *m/z* calcd for $C_{14}H_{16}N_2NaO_2^+$: 267.1104; $[M+Na]^+$ found: 267.1103.

(Z)-N-(Octadec-9-en-1-yl)benzo[d]oxazole-2-carboxamide (29)

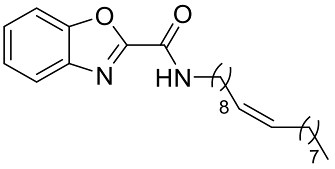

Yellow oil; Yield: **30%** (18 h); **^{1}H NMR (400 MHz, $CDCl_3$)** δ 7.79 (1H, d, *J* = 8.0 Hz, ArH), 7.66 (1H, d, *J* = 8.0 Hz, ArH), 7.48 (1H, t, *J* = 8.0 Hz, ArH), 7.43 (1H, t, *J* = 8.0 Hz, ArH), 7.28 (1H, s, NH), 5.46-5.29 (2H, m, 2 x =CH), 3.51 (2H, q, *J* = 6.6 Hz, NCH_2), 2.14-1.88 (4H, m, 2 x CH_2), 1.70-1.62 (2H, m, CH_2), 1.44-1.22 (22H, m, 11 x CH_2), 0.88 (3H, t, *J* = 5.8 Hz, CH_3); **^{13}C NMR (100 MHz, $CDCl_3$)** δ 155.6, 151.2, 140.2, 131.0, 130.0, 129.8, 127.3, 125.5, 121.1, 111.9, 39.9, 31.9, 29.7, 29.7, 29.7, 29.7, 29.5, 29.4, 29.3, 29.2, 29.2, 27.2, 27.2, 26.9, 22.7, 14.1; **HRMS (ESI$^+$):** *m/z* calcd for $C_{26}H_{40}N_2NaO_2^+$: 435.2982; $[M+Na]^+$ found: 435.2982.

Benzo[*d*]oxazol-2-yl(morpholino)methanone (30)[6]

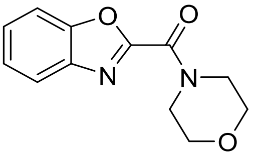

Brown solid; Yield: **35%** (18 h); **m.p.** 98-99 °C (lit. m.p.: 101-103 °C)[5b]; **^{1}H NMR (400 MHz, $CDCl_3$)** δ 7.80 (1H, d, J = 7.5 Hz, ArH), 7.65 (1H, d, J = 7.5 Hz, ArH), 7.48 (1H, t, J = 7.5 Hz, ArH), 7.42 (1H, t, J = 7.5 Hz, ArH), 4.30-4.23 (2H, m, CH_2), 3.90-3.77 (6H, m, 3 x CH_2); **^{13}C NMR (100 MHz, $CDCl_3$)** δ 156.0, 154.6, 149.9, 140.1, 127.3, 125.3, 121.3, 111.6, 67.1, 66.7, 47.5, 43.3; **HRMS (ESI$^+$):** *m/z* calcd for $C_{12}H_{12}N_2NaO_3^+$: 255.0740; [M+Na]$^+$ found: 255.0740.

Ethyl (benzo[*d*]oxazole-2-carbonyl)glycinate (31)[2]

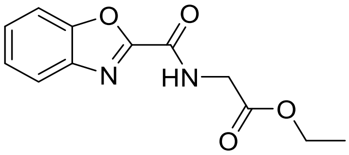

Yellowish solid; Yield: **45%** (18 h); **m.p.** 109-111 °C (lit. m.p.: 109.9-110.4 °C); **^{1}H NMR (400 MHz, $CDCl_3$)** δ 7.82 (1H, d, J = 7.5 Hz, ArH), 7.75 (1H, s, NH), 7.66 (1H, d, J = 7.5 Hz, ArH), 7.49 (1H, t, J = 7.5 Hz, ArH), 7.44 (1H, t, J = 7.5 Hz, ArH), 4.32-4.24 (4H, m, OCH_2 and NCH_2), 1.32 (3H, t, J = 6.6 Hz, CH_3); **^{13}C NMR (100 MHz, $CDCl_3$)** δ 168.8, 155.7, 154.8, 151.1, 140.2, 127.5, 125.6, 121.5, 111.8, 61.9, 41.5, 14.1; **HRMS (ESI$^+$):** *m/z* calcd for $C_{12}H_{12}N_2NaO_4^+$: 271.0689; [M+Na]$^+$ found: 271.0688.

Methyl (benzo[*d*]oxazole-2-carbonyl)leucinate (32)[1]

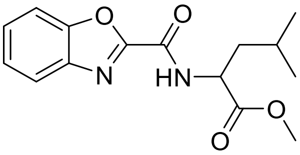

Brown oil; Yield: **35%** (18 h); **^{1}H NMR (400 MHz, $CDCl_3$)** δ 7.81 (1H, d, J = 7.9 Hz, ArH), 7.68-7.57 (2H, m, ArH and NH), 7.52-7.40 (2H, m, ArH), 4.91-4.83 (1H, m, NCH), 3.78 (3H, s, OCH_3), 1.83-1.70 (3H, m, CH and CH_2), 0.99 (6H, t, J = 6.2 Hz, 2 x CH_3); **^{13}C NMR (100 MHz, $CDCl_3$) δ** 172.6, 155.5, 155.1, 151.3, 140.3, 127.7, 125.8, 121.5, 112.0, 52.7, 51.2,

41.7, 25.0, 23.0, 22.0; **HRMS (ESI$^+$):** *m/z* calcd for $C_{15}H_{18}N_2NaO_4^+$: 313.1159; $[M+Na]^+$ found: 313.1159.

N-Benzyl 5-methylbenzo[d]oxazole-2-carboxamide (33)

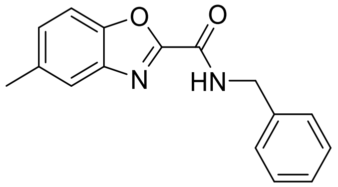

Yellow solid; Yield: **63%** (18 h); **m.p.** 109-111 °C; **^{1}H NMR (400 MHz, CDCl$_3$)** δ 7.66 (1H, br s, NH), 7.54-7.48 (2H, m, ArH), 7.38-7.25 (6H, m, ArH), 4.68 (2H, d, J = 5.7 Hz, NCH_2), 2.47 (3H, s, CH_3); **^{13}C NMR (100 MHz, CDCl$_3$)** δ 155.8, 155.6, 149.5, 140.4, 137.1, 135.7, 129.0, 128.8, 128.1, 128.0, 120.9, 111.3, 44.0, 21.6; **HRMS (ESI$^+$):** *m/z* calcd for $C_{16}H_{14}N_2NaO_2^+$: 289.0947; $[M+Na]^+$ found: 289.0948.

N-Benzyl-6-methylbenzo[d]oxazole-2-carboxamide (34)

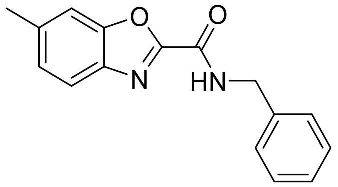

Orange solid; Yield: **40%** (18 h); **m.p.** 112-114 °C; **^{1}H NMR (400 MHz, CDCl$_3$)** δ 7.64 (1H, d, J = 8.0 Hz, ArH), 7.60 (1H, s, NH), 7.47 (1H, s, ArH), 7.44-7.30 (5H, m, ArH), 7.29-7.24 (1H, m, ArH), 4.71 (2H, d, J = 5.8 Hz, NCH_2Ph), 2.54 (3H, s, CH_3); **^{13}C NMR (100 MHz, CDCl$_3$)** δ 155.7, 154.9, 151.4, 138.3, 137.9, 136.9, 128.9, 128.0, 127.9, 127.0, 120.5, 111.8, 43.8, 21.9; **HRMS (ESI$^+$):** *m/z* calcd for $C_{16}H_{14}N_2NaO_2^+$: 289.0947; $[M+Na]^+$ found: 289.0951.

Syntheses of Bromoacetates, Bromoacetamides and Carbamates

General Procedure for the Synthesis of 2-Bromoacetates. To a stirring solution of the corresponding alcohol (1.00 mmol, 1.00 equiv.) in dry CH_2Cl_2 (5 mL), 2-bromoacetic acid (167 mg, 1.20 mmol, 1.20 equiv.), *N,N'*-dicyclohexylcarbodiimide (DCC) (248 mg, 1.20 mmol, 1.20 equiv.) and 4-dimethylaminopyridine (4-DMAP) (12 mg, 0.10 mmol, 0.10 equiv.) were added consecutively at 0 ^{o}C. The reaction mixture was left under stirring at r.t. for 18 h. The solvent was evaporated *in vacuo* and the residue was purified by flash column chromatography (Pet. Ether:EtOAc 80:20-70:30).

Decyl 2-bromoacetate (S1a)[7]

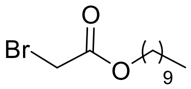

Colorless oil; Yield: **82%**; **^{1}H NMR (400 MHz, CDCl$_3$)** δ 4.18-4.12 (2H, m, OCH_2), 3.81 (2H, s, $BrCH_2$), 1.68-1.60 (2H, m, CH_2), 1.37-1.21 (14H, m, 7 x CH_2), 0.86 (3H, t, *J* = 5.8 Hz, CH_3); **^{13}C NMR (100 MHz, CDCl$_3$)** δ 167.2, 66.4, 31.8, 29.4, 29.4, 29.2, 29.1, 28.4, 25.8, 25.7, 22.6, 14.0; **HRMS (ESI$^+$):** *m/z* calcd for $C_{12}H_{23}BrNaO_2^+$: 301.0774; $[M+Na]^+$ found: 301.0774.

4-Phenylbutyl 2-bromoacetate (S1b)[8]

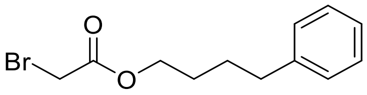

Off-white low melting point solid; Yield: **95%**; **^{1}H NMR (400 MHz, CDCl$_3$)** δ 7.38-7.30 (2H, m, ArH), 7.28-7.20 (3H, m, ArH), 4.23 (2H, t, *J* = 5.5 Hz, OCH_2), 3.85 (2H, s, $BrCH_2$), 2.70 (2H, t, *J* = 5.5 Hz, CH_2Ph), 1.79-1.72 (4H, m, 2 x CH_2); **^{13}C NMR (100 MHz, CDCl$_3$)** δ 167.1, 141.7, 128.3, 125.8, 66.0, 35.2, 27.9, 27.4, 25.9; **HRMS (ESI$^+$):** *m/z* calcd for $C_{12}H_{15}BrNaO_2^+$: 293.0148; $[M+Na]^+$ found: 293.0149.

4-Methoxybenzyl 2-bromoacetate (S1c)[9]

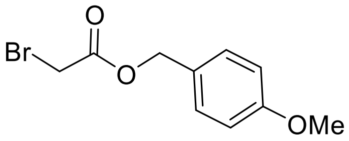

Colorless oil; Yield: **92%**; **^{1}H NMR (400 MHz, CDCl$_3$)** δ 7.30 (2H, d, *J* = 8.0 Hz, ArH), 6.88 (2H, d, *J* = 8.0 Hz, ArH), 5.12 (2H, s, OCH_2), 3.82 (2H, s, $BrCH_2$), 3.78 (3H, s, OCH_3); **^{13}C NMR (100 MHz, CDCl$_3$)** δ 166.9, 159.7, 130.2, 126.9, 113.8, 67.7, 55.1, 25.9; **HRMS (ESI$^+$):** *m/z* calcd for $C_{10}H_{11}BrNaO_3^+$: 280.9784; $[M+Na]^+$ found: 280.9783.

Cyclohexyl 2-bromoacetate (S1d)[10]

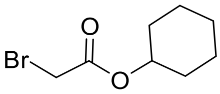

Colorless oil; Yield: **71%**; **^{1}H NMR (400 MHz, CDCl$_3$)** δ 4.85-4.74 (1H, m, OCH), 3.79 (2H, s, $BrCH_2$), 1.87-1.79 (2H, m, 2 x C*H*H), 1.76-1.67 (2H, m, 2 x C*H*H), 1.55-1.22 (6H, m, 6 x C*H*H); **^{13}C NMR (100 MHz, CDCl$_3$)** δ 166.6, 74.7, 31.1, 26.4, 25.1, 23.4; **HRMS (ESI$^+$):** *m/z* calcd for $C_8H_{13}BrNaO_2^+$: 242.9991; $[M+Na]^+$ found: 242.9986.

Adamantan-1-yl 2-bromoacetate (S1e)[11]

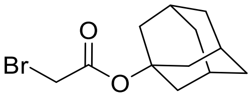

Off-white low melting point solid; Yield: **58%**; **^{1}H NMR (400 MHz, CDCl$_3$)** δ 3.73 (2H, s, $BrCH_2$), 2.20-2.15 (3H, m, 3 x CH), 2.13-2.09 (6H, m, 3 x CH_2), 1.68-1.63 (6H, m, 3 x CH_2); **^{13}C NMR (100 MHz, CDCl$_3$)** δ 165.8, 82.9, 41.0, 36.0, 30.8, 27.8; **HRMS (ESI$^+$):** *m/z* calcd for $C_{12}H_{17}BrNaO_2^+$: 295.0304; $[M+Na]^+$ found: 295.0304.

(*Z*)-Octadec-9-en-1-yl 2-bromoacetate (S1f)[12]

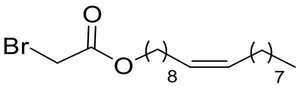

Colorless oil; Yield: **93%**; **^{1}H NMR (400 MHz, CDCl$_3$)** δ 5.46-5.23 (2H, m, 2 x =CH), 4.16 (2H, t, *J* = 6.6 Hz, OCH$_2$), 3.82 (2H, s, BrCH$_2$), 2.13-1.88 (4H, m, 2 x CH$_2$), 1.70-1.59 (2H, m, CH$_2$), 1.39-1.23 (22H, m, 11 x CH$_2$), 0.87 (3H, t, *J* = 5.9 Hz, CH$_3$); **^{13}C NMR (100 MHz, CDCl$_3$)** δ 167.3, 130.0, 129.7, 66.4, 32.6, 31.9, 29.7, 29.7, 29.6, 29.5, 29.3, 29.3, 29.1, 29.1, 28.4, 27.2, 27.1, 25.9, 25.7, 22.6, 14.1; **HRMS (ESI$^+$):** *m/z* calcd for $C_{20}H_{37}BrNaO_2^+$: 411.1869; [M+Na]$^+$ found: 411.1868.

Pent-4-yn-1-yl 2-bromoacetate (S1g)

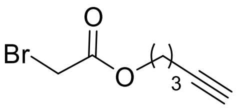

Colorless oil; Yield: **86%**; **^{1}H NMR (400 MHz, CDCl$_3$)** δ 4.28 (2H, t, *J* = 6.8 Hz, OCH$_2$), 3.82 (2H, s, BrCH$_2$), 2.30 (2H, t, *J* = 6.8 Hz, CH$_2$), 1.97 (1H, s, ≡CH), 1.88 (2H, quin, *J* = 6.8 Hz, CH$_2$); **^{13}C NMR (100 MHz, CDCl$_3$)** δ 167.1, 82.6, 69.2, 64.6, 27.2, 25.7, 15.0; **HRMS (ESI$^+$):** *m/z* calcd for $C_7H_9BrNaO_2^+$: 226.9678; [M+Na]$^+$ found: 226.9675.

4-((*tert*-Butoxycarbonyl)amino)butyl 2-bromoacetate (S1h)

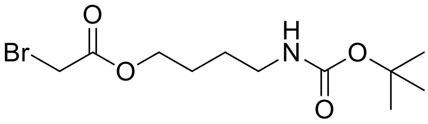

Colorless oil; Yield: **79%**; **^{1}H NMR (400 MHz, CDCl$_3$)** δ 4.56 (1H, br s, NH), 4.16 (2H, t, *J* = 6.7 Hz, OCH$_2$), 3.80 (2H, s, BrCH$_2$), 3.12 (2H, t, *J* = 6.7 Hz, 2H, NCH$_2$), 1.67 (2H, quin, *J* = 6.7 Hz, CH$_2$), 1.54 (2H, quin, *J* = 6.7 Hz, CH$_2$), 1.41 (9H, s, 3 x CH$_3$); **^{13}C NMR (100 MHz, CDCl$_3$)** δ 167.3, 156.1, 79.4, 66.0, 40.3, 28.5, 26.7, 25.9, 25.8; HRMS (ESI$^+$): *m/z* calcd for $C_{11}H_{21}BrNO_4^+$: 310.0648; [M+H]$^+$, found: 310.0644.

General Procedure for the Synthesis of 2-Bromoacetamides. To a flame-dried round-bottom flask under an argon atmosphere, a solution of 2-bromoacetyl chloride (0.09 mL, 1.10 mmol, 1.10 equiv.) in dry THF (1.3 mL) was added. The mixture was cooled at 0 °C and the amine (1.00 mmol, 1.00 equiv.) was added dropwise, followed by 4-DMAP (61 mg, 0.50 mmol, 0.50 equiv.). The reaction mixture was then allowed to warm to room temperature and stirred for 18 h. Upon reaction completion, H_2O (3 mL) was added and the organic layer was extracted with CH_2Cl_2 (2 x 10 mL). The organic layer was separated and washed with saturated aqueous $NaHCO_3$ (2 x 10 mL), brine (10 mL) and, then, dried over Na_2SO_4. The solvent was evaporated under reduced pressure. The synthesized 2-bromoacetamides were used directly to the next step without further purification.

Synthesis of *tert*-Butyl (4-hydroxybutyl)carbamate (S1i)[13]

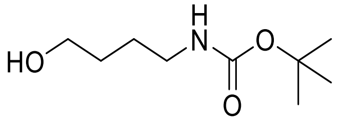

To a dry round bottom flask, a solution of 4-aminobutan-1-ol (0.09 mL, 1.00 mmol, 1.00 equiv.) in MeOH (2 mL) was added and the mixture was left under stirring at 0 ^oC for 10 min. After the dropwise addition of Et_3N (0.20 mL, 1.40 mmol, 1.40 equiv.) and Boc_2O (240 mg, 1.10 mmol, 1.10 equiv.), the reaction mixture was left under stirring at 0 ^oC for 30 min and then at r.t. for 18 h. The solvent was evaporated under reduced pressure, the residue was diluted in EtOAc (15 mL) and washed consecutively with H_2O (10 mL), aqueous $NaHCO_3$ 5% (10 mL), brine (10 mL). The organic layer was separated and dried over Na_2SO_4. The solvent was evaporated under reduced pressure and the residue was purified by flash column chromatography (Pet. Ether:EtOAc 80:20). Colorless oil; Yield: **83%**; **^{1}H NMR (400 MHz, $CDCl_3$)** δ 4.75 (1H, br s, NH), 3.63-3.56 (2H, m, OCH_2), 3.41 (1H, br s, OH), 3.13-3.04 (2H, m, NCH_2), 1.58-1.48 (4H, m, 2 x CH_2), 1.39 (9H, s, 3 x CH_3); **^{13}C NMR (100 MHz, $CDCl_3$)** δ 156.4, 79.3, 62.2, 40.5, 29.7, 28.5, 26.6; MS (ESI) *m/z* 212 $[M+Na]^+$.

General Procedure for the Synthesis of Glycine Derivatives Using Potassium Fluoride

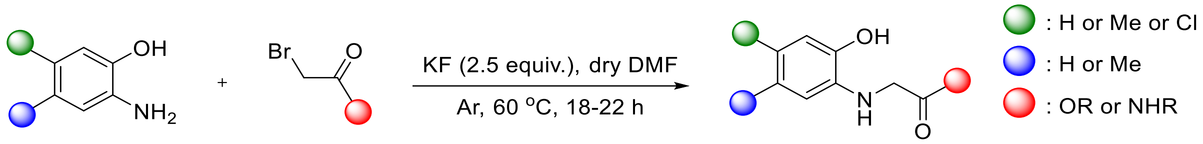

To a flame-dried round-bottom flask, 2-aminophenol or the corresponding substituted derivative (1.00 mmol, 1.00 equiv.) and potassium fluoride (145 mg, 2.50 mmol, 2.50 equiv.) were added. The flask was sealed, degassed and purged with argon before adding dry DMF (5 mL) and the appropriate bromoacetate or bromoacetamide (1.00 mmol, 1.00 equiv.). The reaction mixture was stirred at 60 °C for 18 h under an Ar atmosphere. After reaction completion, the solvent was removed under reduced pressure and the residue was diluted with EtOAc (10 mL), then washed with saturated aqueous $NaHCO_3$ (2 x 10 mL) followed by brine (2 x 10 mL). The organic phase was dried over Na_2SO_4, filtered and concentrated under reduced pressure and the residue was purified by flash column chromatography (Pet. Ether:EtOAc 80:20-50:50).

Ethyl (2-hydroxyphenyl)glycinate (3a)[14]

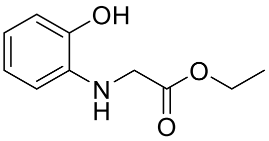

Orange solid; Yield: **83%**; **m.p.** 88-91 °C (lit. m.p.: 90-94 °C); **^{1}H NMR (400 MHz, $CDCl_3$)** δ 6.83 (1H, t, J = 7.8 Hz, ArH), 6.72 (1H, d, J = 7.8 Hz, ArH), 6.66 (1H, t, J = 7.8 Hz, ArH), 6.58 (1H, d, J = 7.8 Hz, ArH), 5.14 (2H, br s, NH and OH), 4.26 (2H, q, J = 7.2 Hz, OCH_2), 3.93 (2H, s, NCH_2), 1.30 (3H, t, J = 7.2 Hz, CH_3); **^{13}C NMR (100 MHz, $CDCl_3$)** δ 172.3, 144.6, 136.1, 121.5, 119.1, 115.0, 113.0, 61.6, 46.6, 14.3; **HRMS (ESI$^+$):** m/z calcd for $C_{10}H_{13}NNaO_3^+$: 218.0788; $[M+Na]^+$ found: 218.0788.

Methyl (2-hydroxyphenyl)glycinate (3b)[1]

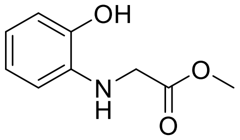

Red low melting ponting solid; Yield: **82%**; **^{1}H NMR (400 MHz, $CDCl_3$)** δ 6.81 (1H, t, J = 7.7 Hz, ArH), 6.70 (1H, d, J = 7.7 Hz, ArH), 6.63 (1H, t, J = 7.7 Hz, ArH), 6.55 (1H, d, J = 7.7 Hz, ArH), 5.31 (2H, br s, NH and OH), 3.95 (2H, s, NCH_2), 3.77 (3H, s, OCH_3); **^{13}C NMR (100 MHz, $CDCl_3$)** δ 172.7, 144.3, 136.1, 121.2, 118.8, 114.9, 112.2, 52.4, 46.1; **HRMS (ESI$^+$):** m/z calcd for $C_9H_{12}NO_3^+$: 182.0812; $[M+H]^+$ found: 182.0809.

Decyl 2-((2-hydroxyphenyl)amino)acetate (**3c**)

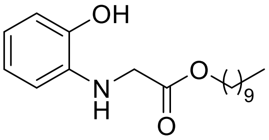

Red solid; Yield: **86%**; **m.p.:** 64-67 ^{o}C; **^{1}H NMR (400 MHz, $CDCl_3$)** δ 6.81 (1H, t, J = 7.8 Hz, ArH), 6.70 (1H, d, J = 7.8 Hz, ArH), 6.63 (1H, t, J = 7.8 Hz, ArH), 6.56 (1H, d, J = 7.8 Hz, ArH), 5.57 (2H, br s, NH and OH), 4.19 (2H, t, J = 6.2 Hz, OCH_2), 3.94 (2H, s, NCH_2), 1.74-1.61 (2H, m, CH_2), 1.38-1.23 (14H, m, 7 x CH_2), 0.91 (3H, t, J = 6.4 Hz, CH_3); **^{13}C NMR (100 MHz, $CDCl_3$)** δ 172.3, 144.3, 136.0, 121.1, 118.7, 114.8, 112.2, 65.6, 46.2, 31.8, 29.5, 29.4, 29.2, 29.2, 28.5, 25.8, 22.6, 14.0; **HRMS (ESI$^+$):** m/z calcd for $C_{18}H_{29}NNaO_3^+$: 330.2040; $[M+Na]^+$ found: 330.2038.

4-Phenylbutyl (2-hydroxyphenyl)glycinate (3d)

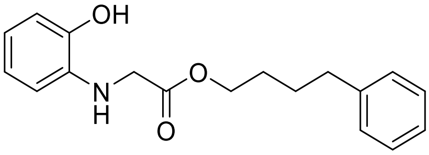

Off-white low melting ponting solid; Yield: **67%**; **^{1}H NMR (400 MHz, $CDCl_3$)** δ 7.34-7.28 (2H, m, ArH), 7.25-7.16 (3H, m, ArH), 6.83 (1H, t, J = 7.8 Hz, ArH), 6.72 (1H, d, J = 7.8 Hz, ArH), 6.66 (1H, t, J = 7.8 Hz, ArH), 6.58 (1H, d, J = 7.8 Hz, ArH), 4.99 (2H, br s, NH and OH), 4.22 (2H, t, J = 5.8 Hz, OCH_2), 3.94 (2H, s, NCH_2), 2.65 (2H, t, J = 6.1 Hz, CH_2Ar),

1.74-1.67 (4H, m, 2 x CH_2); **^{13}C NMR (100 MHz, $CDCl_3$)** δ 172.3, 144.5, 142.0, 136.0, 128.5, 128.5, 126.0, 121.4, 119.1, 115.0, 112.8, 65.5, 46.5, 35.5, 28.2, 27.7; **HRMS (ESI$^+$):** *m/z* calcd for $C_{18}H_{21}NNaO_3^+$: 322.1414 [M+Na]$^+$, found: 322.1413.

Benzyl (2-hydroxyphenyl)glycinate (3e)[1]

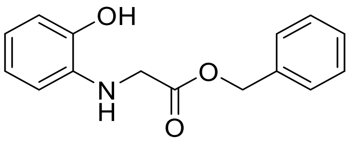

Red low melting ponting solid; Yield: **59%**; **^{1}H NMR (400 MHz, $CDCl_3$)** δ 7.46-7.31 (5H, m, ArH), 6.83 (1H, t, J = 7.8 Hz, ArH), 6.74-6.64 (2H, m, ArH), 6.57 (1H, d, J = 7.8 Hz, ArH), 5.32 (1H, br s, NH), 5.24 (2H, s, OCH_2), 5.13 (1H, br s, OH), 4.00 (2H, s, NCH_2); **^{13}C NMR (100 MHz, $CDCl_3$)** δ 172.2, 144.4, 136.0, 135.3, 128.7, 128.6, 128.5, 121.3, 119.0, 115.0, 112.5, 67.3, 46.4; **HRMS (ESI$^+$):** *m/z* calcd for $C_{15}H_{15}NNaO_3^+$: 280.0944; [M+Na]$^+$ found: 280.0944.

4-Methoxybenzyl (2-hydroxyphenyl)glycinate (3f)

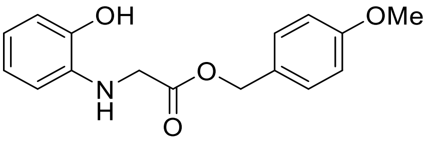

Brown oil; Yield: **62%**; **^{1}H NMR (400 MHz, $CDCl_3$)** δ 7.31 (2H, d, J = 8.0 Hz, ArH), 6.91 (2H, d, J = 8.0 Hz, ArH), 6.81 (1H, t, J = 8.0 Hz, ArH), 6.73-6.62 (2H, m, ArH), 6.55 (1H, d, J = 8.0 Hz, ArH), 5.40 (2H, br s, NH and OH), 5.17 (2H, s, OCH_2), 3.96 (2H, s, NCH_2), 3.82 (3H, s, OCH_3); **^{13}C NMR (100 MHz, $CDCl_3$)** δ 172.0, 159.7, 144.3, 135.9, 130.2, 127.4, 121.2, 118.8, 114.8, 114.0, 112.4, 67.0, 55.2, 46.3; **HRMS (ESI$^+$):** *m/z* calcd for $C_{16}H_{17}NNaO_4^+$: 310.1050; [M+Na]$^+$ found: 310.1050.

Cyclohexyl 2-((2-hydroxyphenyl)amino)acetate (3g)

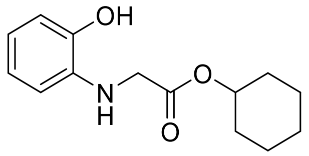

Brownish solid; Yield: **81%**; **m.p.:** 88-90 °C; **^{1}H NMR (400 MHz, CDCl$_3$)** δ 6.81 (1H, t, *J* = 7.8 Hz, ArH), 6.70 (1H, d, *J* = 7.8 Hz, ArH), 6.63 (1H, t, *J* = 7.8 Hz, ArH), 6.56 (1H, d, *J* = 7.8 Hz, ArH), 6.38 (1H, br s, OH), 4.95-4.85 (1H, m, OCH), 4.64 (1H, br s, NH), 3.93 (2H, s, NCH_2), 1.95-1.81 (2H, m, 2 x C*H*H), 1.79-1.67 (2H, m, 2 x C*H*H), 1.59-1.25 (6H, m, 6 x C*H*H); **^{13}C NMR (100 MHz, CDCl$_3$)** δ 171.7, 144.4, 136.1, 121.0, 118.7, 114.7, 112.3, 74.1, 46.6, 31.4, 25.2, 23.5; **HRMS (ESI$^+$):** *m/z* calcd for $C_{14}H_{19}NNaO_3^+$: 272.1257; $[M+Na]^+$ found: 272.1258.

***tert*-Butyl (2-hydroxyphenyl)glycinate (3h)[15]**

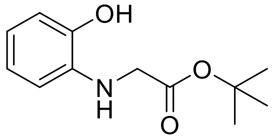

Yellow solid; Yield: **78%**; **m.p.** 82-84 °C; **^{1}H NMR (400 MHz, CDCl$_3$)** δ 6.81 (1H, t, *J* = 7.8 Hz, ArH), 6.69 (1H, d, *J* = 7.8 Hz, ArH), 6.62 (1H, t, *J* = 7.8 Hz, ArH), 6.55 (1H, d, *J* = 7.8 Hz, ArH), 5.61 (2H, br s, NH and OH), 3.85 (2H, s, NCH_2), 1.51 (9H, s, 3 x CH_3); **^{13}C NMR (100 MHz, CDCl$_3$)** δ 171.7, 144.6, 136.3, 121.1, 118.7, 114.8, 112.4, 82.4, 47.1, 28.2; **HRMS (ESI$^+$):** *m/z* calcd for $C_{12}H_{18}NO_3^+$: 224.1281; $[M+H]^+$ found: 224.1281.

Adamantan-1-yl 2-((2-hydroxyphenyl)amino)acetate (3i)

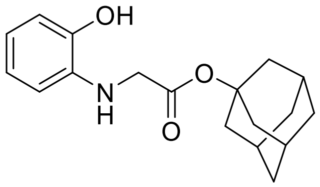

Brownish solid; Yield: **63%**; **m.p.** 102-104 °C; **^{1}H NMR (400 MHz, CDCl$_3$)** δ 6.81 (1H, t, *J* = 7.7 Hz, ArH), 6.71 (1H, d, *J* = 7.7 Hz, ArH), 6.63 (1H, t, *J* = 7.7 Hz, ArH), 6.56 (1H, d, *J* = 7.7 Hz, ArH), 5.43 (2H, br s, NH and OH), 3.83 (2H, s, NCH_2), 2.20-2.13 (9H, m, 3 x CH_2 and 3 x CH), 1.70-1.64 (6H, m, 3 x CH_2); **^{13}C NMR (100 MHz, CDCl$_3$)** δ 171.1, 144.5,

136.2, 121.1, 118.6, 114.7, 112.6, 82.3, 47.2, 41.3, 36.0, 30.8; **HRMS (ESI$^+$):** *m/z* calcd for $C_{18}H_{23}NNaO_3^+$: 324.1570; [M+H]$^+$ found: 324.1572.

(*Z*)-Octadec-9-en-1-yl 2-((2-hydroxyphenyl)amino)acetate (**3j**)

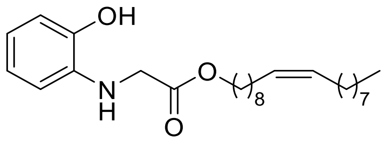

Yellowish oil; Yield: **80%**; **^{1}H NMR (400 MHz, CDCl$_3$)** δ 6.82 (1H, t, *J* = 7.8 Hz, ArH), 6.72 (1H, d, *J* = 7.8 Hz, ArH), 6.65 (1H, t, *J* = 7.8 Hz, ArH), 6.58 (1H, d, *J* = 7.8 Hz, ArH), 5.84 (1H, br s, OH), 5.42-5.33 (2H, m, 2 x =CH), 4.69 (1H, br s, NH), 4.19 (2H, t, *J* = 7.7 Hz, OCH_2), 3.94 (2H, s, NCH_2), 2.06-1.96 (4H, m, 2 x CH_2), 1.69-1.61 (2H, m, CH_2), 1.38-1.25 (22H, m, 11 x CH_2), 0.89 (3H, t, *J* = 6.4 Hz, CH_3); **^{13}C NMR (100 MHz, CDCl$_3$)** δ 172.2, 144.4, 136.0, 130.0, 129.8, 121.3, 118.9, 114.8, 112.7, 65.6, 46.4, 32.6, 31.9, 29.7, 29.7, 29.7, 29.6, 29.5, 29.4, 29.3, 29.2, 28.5, 27.2, 27.2, 25.8, 22.6, 14.1; **HRMS (ESI$^+$):** *m/z* calcd for $C_{26}H_{43}NNaO_3^+$: 440.3135; [M+Na]$^+$ found: 440.3135.

Pent-4-yn-1-yl 2-((2-hydroxyphenyl)amino)acetate (**3k**)

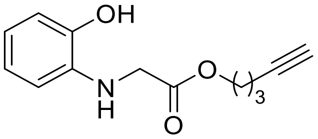

Brown solid; Yield: **87%**; **m.p.** 80-82 °C; **^{1}H NMR (400 MHz, CDCl$_3$)** δ 6.82 (1H, t, *J* = 7.8 Hz, ArH), 6.71 (1H, d, *J* = 7.8 Hz, ArH), 6.64 (1H, t, *J* = 7.8 Hz, ArH), 6.56 (1H, d, *J* = 7.8 Hz, ArH), 5.36 (2H, br s, NH and OH), 4.30 (2H, t, *J* = 6.2 Hz, OCH_2), 3.95 (2H, s, NCH_2), 2.27 (2H, t, *J* = 7.0 Hz, CH_2), 1.99 (1H, s, ≡CH), 1.88 (2H, quin, *J* = 8.8 Hz, CH_2); **^{13}C NMR (100 MHz, CDCl$_3$)** δ 172.1, 144.3, 135.9, 121.2, 118.8, 114.8, 112.4, 82.8, 69.2, 63.9, 46.3, 27.3, 15.0; **HRMS (ESI$^+$):** *m/z* calcd for $C_{13}H_{15}NNaO_3^+$: 256.0944; [M+Na]$^+$ found: 256.0945.

4-((*tert*-Butoxycarbonyl)amino)butyl (2-hydroxyphenyl)glycinate (3l)

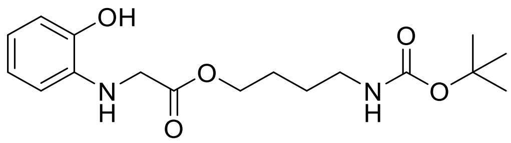

Yellowish low melting ponting solid; Yield: **70%**; **^{1}H NMR (400 MHz, CDCl$_3$)** δ 6.81-6.74 (2H, m, ArH), 6.62 (1H, t, J = 7.7 Hz, ArH), 6.54 (1H, d, J = 7.7 Hz, ArH), 5.28 (2H, br s, NH and OH), 4.70 (1H, br s, NH), 4.12 (2H, t, J = 6.2 Hz, OCH$_2$), 3.93 (2H, s, NCH$_2$), 3.12-2.98 (2H, m, NCH$_2$), 1.62-1.53 (2H, m, CH$_2$), 1.50-1.41 (11H, m, CH$_2$ and 3 x CH$_3$); **^{13}C NMR (100 MHz, CDCl$_3$)** δ 171.9, 156.6, 144.6, 136.4, 121.0, 118.6, 114.9, 112.2, 79.8, 64.9, 46.6, 40.3, 28.5, 26.7, 25.9; **HRMS (ESI$^+$):** m/z calcd for $C_{17}H_{26}N_2NaO_5^+$: 361.1734; [M+Na]$^+$ found: 361.1739.

Ethyl (2-hydroxy-5-methylphenyl)glycinate (3m)[14]

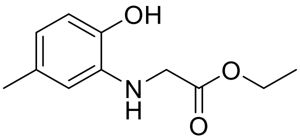

Yellow solid; Yield: **69%**; **m.p.** 101-103 °C (lit. m.p.: 114-118 °C); **^{1}H NMR (400 MHz, CDCl$_3$)** δ 6.62 (1H, d, J = 8.0 Hz, ArH), 6.45 (1H, d, J = 8.0 Hz, ArH), 6.39 (1H, s, ArH), 5.02 (2H, br s, NH and OH), 4.25 (2H, q, J = 7.1 Hz, OCH$_2$), 3.92 (2H, s, NCH$_2$), 2.24 (3H, s, CH$_3$), 1.30 (3H, t, J = 7.1 Hz, CH$_3$); **^{13}C NMR (100 MHz, CDCl$_3$)** δ 172.3, 142.2, 136.0, 130.8, 119.2, 114.9, 113.7, 61.6, 46.6, 21.1, 14.3; **HRMS (ESI$^+$):** m/z calcd for $C_{11}H_{15}NNaO_3^+$: 280.0944; [M+Na]$^+$ found: 232.0945.

4-Methoxybenzyl 2-((2-hydroxy-5-methylphenyl)amino)acetate (**3n**)

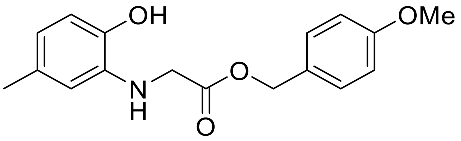

Brownish solid; Yield: **89%**; **m.p.:** 86-88 °C; **^{1}H NMR (400 MHz, CDCl$_3$)** δ 7.30 (2H, d, J = 8.0 Hz, ArH), 6.89 (2H, d, J = 8.0 Hz, ArH), 6.63 (1H, d, J = 8.0 Hz, ArH), 6.45 (1H, d, J = 8.0 Hz, ArH), 6.36 (1H, s, ArH), 5.16 (2H, s, OCH$_2$), 4.67 (2H, br s, OH and NH), 3.94 (2H, s, NCH$_2$), 3.82 (3H, s, OCH$_3$), 2.21 (3H, s, CH$_3$); **^{13}C NMR (100 MHz, CDCl$_3$)** δ 171.9,

159.7, 142.1, 135.7, 130.7, 130.2, 127.5, 119.2, 114.8, 114.0, 113.7, 66.9, 55.3, 46.6, 21.0; **HRMS (ESI$^+$):** *m/z* calcd for $C_{17}H_{19}NNaO_4^+$: 324.1206; $[M+Na]^+$ found: 324.1207.

Cyclohexyl (2-hydroxy-5-methylphenyl)glycinate (3o)

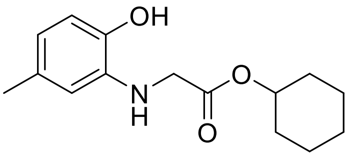

Brownish solid; Yield: **59%**; **m.p.:** 79-81 °C; **^{1}H NMR (400 MHz, CDCl$_3$)** δ 6.61 (1H, d, *J* = 6.0 Hz, ArH), 6.44 (1H, d, *J* = 6.0 Hz, ArH), 6.38 (1H, s, ArH), 5.16 (2H, br s, NH and OH), 4.93-4.84 (1H, m, OCH), 3.91 (2H, s, NCH$_2$), 2.23 (3H, s, CH$_3$), 1.91-1.83 (2H, m, 2 x C*H*H), 1.78-1.68 (2H, m, 2 x C*H*H), 1.59-1.24 (6H, m, 6 x C*H*H); **^{13}C NMR (100 MHz, CDCl$_3$)** δ 171.6, 142.1, 135.8, 130.4, 118.9, 114.7, 113.4, 74.0, 46.7, 31.4, 25.2, 23.5, 21.0; **HRMS (ESI$^+$):** *m/z* calcd for $C_{15}H_{21}NNaO_3^+$: 286.1414; $[M+Na]^+$ found: 286.1413.

Methyl 2-((2-hydroxy-4-methylphenyl)amino)acetate (3p)

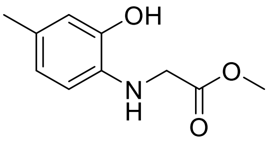

Red-brown solid; Yield: **42%**; **^{1}H NMR (400 MHz, CDCl$_3$)** δ 6.62 (1H, d, *J* = 8.1 Hz, ArH), 6.52-6.47 (2H, m, ArH), 5.37 (2H, br s, NH and OH), 3.92 (2H, s, NCH$_2$), 3.78 (3H, s, OCH$_3$), 2.18 (3H, s, CH$_3$); **^{13}C NMR (100 MHz, CDCl$_3$)** δ 172.8, 144.8, 133.2, 129.2, 121.2, 115.9, 113.3, 52.3, 46.7, 20.5; **HRMS (ESI$^+$):** *m/z* calcd for $C_{10}H_{13}NNaO_3^+$: 218.0788; $[M+Na]^+$ found: 218.0789.

4-Methoxybenzyl 2-((2-hydroxy-4-methylphenyl)amino)acetate (3q)

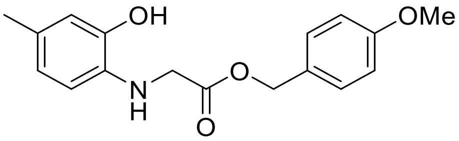

Brown solid; Yield: **75%**; **m.p.:** 98-100 °C; **^{1}H NMR (400 MHz, CDCl$_3$)** δ 7.29 (2H, d, *J* = 8.0 Hz, ArH), 6.89 (2H, d, *J* = 8.0 Hz, ArH), 6.61 (1H, d, *J* = 8.0 Hz, ArH), 6.56 (1H, s,

ArH), 6.52 (1H, d, *J* = 8.0 Hz, ArH), 5.14 (2H, s, OCH_2), 4.62 (2H, br s, OH and NH), 3.91 (2H, s, NCH_2), 3.82 (3H, s, OCH_3), 2.20 (3H, s, CH_3); **^{13}C NMR (100 MHz, $CDCl_3$)** δ 172.2, 159.8, 145.2, 133.1, 130.3, 129.7, 127.4, 121.4, 115.9, 114.4, 114.0, 66.9, 55.3, 47.4, 20.6; **HRMS (ESI$^+$):** *m/z* calcd for $C_{17}H_{20}NO_4^+$: 302.1387; $[M+H]^+$ found: 302.1388.

tert-Butyl 2-((2-hydroxy-4-methylphenyl)amino)acetate (**3r**)

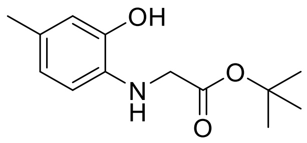

Brown solid; Yield: **73%**; **m.p.:** 84-86 °C; **^{1}H NMR (400 MHz, $CDCl_3$)** δ 6.60 (1H, d, *J* = 8.0 Hz, ArH), 6.54-6.43 (2H, m, ArH), 5.61 (2H, br s, NH and OH), 3.83 (2H, s, NCH_2), 2.17 (3H, s, CH_3), 1.51 (9H, s, 3 x CH_3); **^{13}C NMR (100 MHz, $CDCl_3$)** δ 171.7, 144.8, 133.4, 128.6, 120.9, 115.8, 112.9, 82.1, 47.4, 27.9, 20.5; **HRMS (ESI$^+$):** *m/z* calcd for $C_{13}H_{20}NO_3^+$: 238.1438; $[M+H]^+$ found: 238.1438.

Pent-4-yn-1-yl 2-((2-hydroxy-4-methylphenyl)amino)acetate (**3s**)

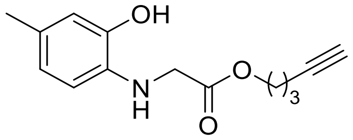

Brown oil; Yield: **77%**; **^{1}H NMR (400 MHz, $CDCl_3$)** δ 6.62 (1H, d, *J* = 8.0 Hz, ArH), 6.57-6.48 (2H, m, ArH), 4.88 (2H, br s, NH and OH), 4.28 (2H, t, *J* = 5.8 Hz, OCH_2), 3.91 (2H, s, NCH_2), 2.30-2.24 (2H, m, CH_2), 2.19 (3H, s, CH_3), 1.98 (1H, s, ≡CH), 1.87 (2H, quin, *J* = 7.9 Hz, CH_2); **^{13}C NMR (100 MHz, $CDCl_3$)** δ 172.2, 145.0, 133.0, 129.5, 121.3, 115.9, 113.9, 82.8, 69.2, 63.8, 47.1, 27.3, 20.6, 15.0; **HRMS (ESI$^+$):** *m/z* calcd for $C_{14}H_{18}NO_3^+$: 248.1281; $[M+H]^+$ found: 248.1281.

tert-Butyl 2-((4-chloro-2-hydroxyphenyl)amino)acetate (3t)

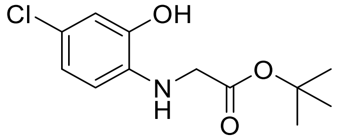

Brown solid; Yield: **46%**; **m.p.:** 72-74 °C; **^{1}H NMR (400 MHz, $CDCl_3$)** δ 7.34 (1H, br s, OH), 6.73 (1H, d, J = 8.5 Hz, ArH), 6.53 (1H, s, ArH), 6.34 (1H, d, J = 8.5 Hz, ArH), 4.58 (1H, br s, NH), 3.85 (2H, s, NCH_2), 1.52 (9H, s, 3 x CH_3); **^{13}C NMR (100 MHz, $CDCl_3$)** δ 172.2, 144.9, 134.6, 122.5, 120.3, 114.7, 111.6, 83.0, 46.4, 28.0; **HRMS (ESI$^+$):** *m/z* calcd for $C_{12}H_{16}ClNNaO_3^+$: 280.0711; $[M+Na]^+$ found: 280.0709.

N-Benzyl-2-((2-hydroxyphenyl)amino)acetamide (3u)[1]

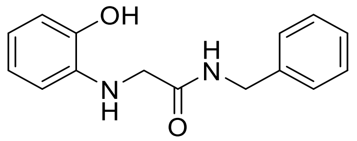

Yellow solid; Yield: **80%**; **m.p.:** 149-150 °C; **^{1}H NMR (400 MHz, CD_3OD)** δ 7.27-7.18 (5H, m, ArH), 6.69 (2H, t, J = 7.9 Hz, ArH), 6.57 (1H, t, J = 7.9 Hz, ArH), 6.43 (1H, d, J = 7.9 Hz, ArH), 4.39 (2H, s, NCH_2), 3.81 (2H, s, NCH_2); **^{13}C NMR (100 MHz, CD_3OD)** δ 174.2, 146.0, 139.8, 137.9, 129.4, 128.4, 128.1, 121.2, 119.2, 114.8, 112.0, 49.1, 43.8; **HRMS (ESI$^+$):** *m/z* calcd for $C_{15}H_{16}N_2NaO_2^+$: 279.1104; $[M+Na]^+$ found: 279.1105.

Methyl 4-((2-((2-hydroxyphenyl)amino)acetamido)methyl)benzoate (3v)

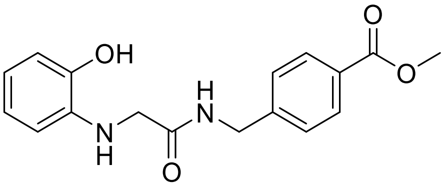

Brownish low melting ponting solid; Yield: **75%**; **^{1}H NMR (400 MHz, $CDCl_3$)** δ 7.87 (2H, d, J = 8.0 Hz, ArH), 7.55 (1H, br s, NH), 7.17 (2H, d, J = 8.0 Hz, ArH), 6.80-6.72 (2H, m, ArH), 6.62 (1H, t, J = 8.0 Hz, ArH), 6.44 (1H, d, J = 8.0 Hz, ArH), 4.43 (2H, d, J = 6.2 Hz, NCH_2), 3.86 (3H, s, OCH_3), 3.81 (2H, s, NCH_2); **^{13}C NMR (100 MHz, $CDCl_3$) δ** 172.4, 167.1, 144.1, 143.1, 136.0, 129.9, 129.1, 127.3, 120.9, 119.1, 114.5, 111.4, 52.3, 48.7, 42.8; **HRMS (ESI$^+$):** *m/z* calcd for $C_{17}H_{18}N_2NaO_3^+$: 337.1159; $[M+Na]^+$ found: 337.1159.

N-(4-Hydroxybenzyl)-2-((2-hydroxyphenyl)amino)acetamide (3w)

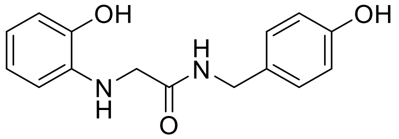

Brown solid; Yield: **19%**; **m.p.:** 76-78 °C; **^{1}H NMR (400 MHz, CD_3OD)** δ 7.07-7.01 (2H, m, ArH), 6.73-6.65 (4H, m, ArH), 6.61-6.54 (1H, m, ArH), 6.47-6.38 (1H, m, ArH), 4.29 (2H, s, NCH_2), 3.79 (2H, s, NCH_2); **^{13}C NMR (100 MHz, CD_3OD)** δ 173.9, 157.7, 146.0, 137.8, 130.5, 129.9, 121.2, 119.3, 116.2, 114.9, 112.2, 49.1, 43.5; **HRMS (ESI$^+$):** *m/z* calcd for $C_{15}H_{16}N_2NaO_3^+$: 259.1053; $[M+Na]^+$ found: 259.1053.

2-((2-Hydroxyphenyl)amino)-1-morpholinoethan-1-one (3y)

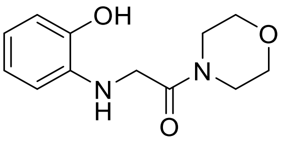

Brownish solid; Yield: **73%**; **m.p.:** 150-152 °C; **^{1}H NMR (400 MHz, $CDCl_3$)** δ 6.89-6.69 (4H, m, ArH), 5.74 (2H, br s, NH and OH), 3.97 (2H, s, NCH_2), 3.71-3.61 (6H, m, 3 x CH_2), 3.47-3.39 (2H, m, CH_2); **^{13}C NMR (100 MHz, $CDCl_3$)** δ 168.7, 146.3, 135.0, 120.9, 115.8, 115.6, 66.7, 66.3, 47.5, 44.9, 42.5; **HRMS (ESI$^+$):** *m/z* calcd for $C_{12}H_{17}N_2O_3^+$: 237.1234; $[M+H]^+$ found: 237.1232.

N-Benzyl-2-((2-hydroxy-5-methylphenyl)amino)acetamide (3z)

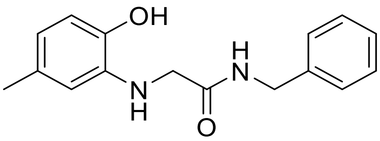

Yellow solid; Yield: **80%**; **m.p.:** 134-136 °C; **^{1}H NMR (400 MHz, CD_3OD)** δ 7.26-7.18 (5H, m, ArH), 6.58 (1H, d, J = 7.9 Hz, ArH), 6.37 (1H, d, J = 7.9 Hz, ArH), 6.23 (1H, s, ArH), 4.40 (2H, s, NCH_2), 3.79 (2H, s, NCH_2), 2.15 (3H, s, CH_3); **^{13}C NMR (100 MHz, CD_3OD)** δ 174.2, 143.8, 139.9, 137.4, 130.4, 129.5, 128.4, 128.1, 119.6, 114.8, 113.2, 49.2, 43.7, 21.2; **HRMS (ESI$^+$):** *m/z* calcd for $C_{16}H_{18}N_2NaO_2^+$: 293.1260; $[M+Na]^+$ found: 293.1260.

N-Benzyl-2-((2-hydroxy-4-methylphenyl)amino)acetamide (**3aa**)

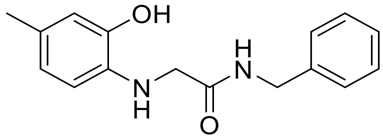

Orange solid; Yield: **93%**; **m.p.:** 98-100 °C; **^{1}H NMR (400 MHz, CDCl$_3$)** δ 7.38 (1H, s, OH), 7.32-7.24 (3H, m, ArH), 7.21 (2H, d, J = 7.8 Hz, ArH), 6.68-6.60 (2H, m, ArH), 6.45 (1H, d, J = 7.8 Hz, ArH), 4.48 (2H, d, J = 6.1 Hz, NCH$_2$), 3.83 (2H, s, NCH$_2$), 2.22 (3H, s, CH$_3$); **^{13}C NMR (100 MHz, CDCl$_3$)** δ 171.9, 143.9, 137.7, 133.6, 128.9, 128.6, 127.6, 127.4, 121.3, 115.6, 112.0, 49.3, 43.2, 20.5; **HRMS (ESI$^+$):** m/z calcd for $C_{16}H_{19}N_2O_2^+$: 271.1441; $[M+H]^+$ found: 271.1441.

General Procedure for the Synthesis of Glycine Derivatives Using Coupling Reagents

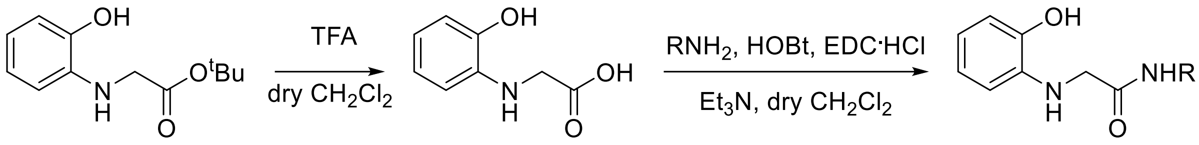

A solution of *tert*-butyl (2-hydroxyphenyl)glycinate (**3h**) (223 mg, 1.00 mmol, 1.00 equiv.) along with trifluoroacetic acid (TFA) (5 mL) in CH_2Cl_2 (10 mL) was stirred for 1 h at room temperature. The organic solvent was evaporated under reduced pressure and then CH_2Cl_2 (5 mL) was added and re-evaporated twice. The product was used in the next step without further purification. To a round bottom flask, product (2-hydroxyphenyl)glycine (167 mg, 1.00 mmol, 1.00 equiv.) and the corresponding amine (1.00 mmol, 1.00 equiv.) were dissolved in dry CH_2Cl_2 (10 mL). The solution was cooled at 0 °C and then, Et_3N (0.31 mL, 2.20 mmol, 2.20 equiv. in case of hydrochloride salt of the amine or 0.15 mL, 1.10 mmol, 1.10 equiv. in case of free amine) and, subsequently, *N*-(3-dimethylaminopropyl)-*N*-ethylcarbodiimide hydrochloride (EDCI•HCl) (211 mg, 1.10 mmol, 1.10 equiv.) and 1-hydroxybenzotriazole (HOBt) (135 mg, 1.00 mmol, 1.00 equiv.) were added and the reaction mixture was left for 1 h at 0 °C, then warmed up at r.t. and left under stirring for 18 h. Upon reaction completion, the solvent was evaporated *in vacuo* and the residue was diluted with EtOAc (15 mL). The organic layer was washed with H_2O (1 x 10 mL), aqueous HCl 1 N (1 x 10 mL), washed with saturated aqueous $NaHCO_3$ (1 x 10 mL) and brine (1 x 10 mL). The organic layer was separated and dried over Na_2SO_4. The solvent was evaporated *in vacuo* and the residue was purified by flash column chromatography (Pet. Ether:EtOAc 80:20-50:50).

_N_-Hexyl-2-((2-hydroxyphenyl)amino)acetamide (3ab)

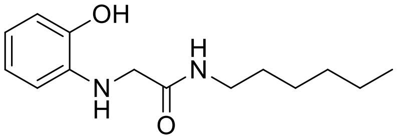

Yellow oil; Yield: **40%**; **^{1}H NMR (400 MHz, $CDCl_3$)** δ 8.62 (1H, br s, NH), 7.16 (1H, t, J = 6.2 Hz, NH), 6.89 (1H, d, J = 7.7 Hz, ArH), 6.78 (1H, t, J = 7.7 Hz, ArH), 6.68 (1H, t, J = 7.7 Hz, ArH), 6.46 (1H, d, J = 7.7 Hz, ArH), 4.77 (1H, br s, OH), 3.81 (2H, s, NCH_2), 3.28 (2H, q, J = 6.9 Hz, NCH_2), 1.46 (2H, quin, J = 6.9 Hz, CH_2), 1.29-1.20 (6H, m, 3 x CH_2), 0.85

(3H, t, *J* = 6.6 Hz, CH_3); **^{13}C NMR (100 MHz, $CDCl_3$)** *δ* 172.3, 144.4, 136.3, 120.8, 119.1, 114.5, 111.3, 49.0, 39.5, 31.4, 29.4, 26.5, 22.6, 14.0; **HRMS (ESI$^+$):** *m/z* calcd for $C_{14}H_{23}N_2O_2^+$: 251.1754 [M+H]$^+$, found: 251.1749.

N-Cyclohexyl-2-((2-hydroxyphenyl)amino)acetamide (3ac)

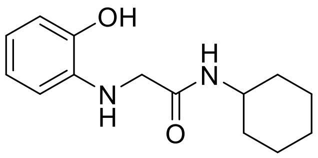

Orange solid; Yield: **24%**; **m.p.:** 155-156 °C; **^{1}H NMR (400 MHz, $CDCl_3$)** *δ* 7.95 (1H, br s, NH), 6.94 (1H, d, *J* = 8.1 Hz, NH), 6.85 (1H, d, *J* = 7.5 Hz, ArH), 6.79 (1H, t, *J* = 7.5 Hz, ArH), 6.68 (1H, t, *J* = 7.5 Hz, ArH), 6.47 (1H, d, *J* = 7.5 Hz, ArH), 4.76 (1H, br s, OH), 3.89-3.73 (3H, m, NCH_2 and NCH), 1.91-1.79 (2H, m, 2 x C*H*H), 1.71-1.53 (3H, m, 3 x C*H*H), 1.38-1.24 (2H, m, 2 x C*H*H), 1.16-1.04 (3H, m, 3 x C*H*H); **^{13}C NMR (100 MHz, $CDCl_3$)** *δ* 170.8, 144.0, 136.3, 120.9, 118.9, 114.4, 111.6, 49.2, 48.2, 32.8, 25.3, 24.7; **HRMS (ESI$^+$):** *m/z* calcd for $C_{14}H_{21}N_2O_2^+$: 249.1598; [M+H]$^+$ found: 249.1598.

(*Z*)-2-((2-Hydroxyphenyl)amino)-*N*-(octadec-9-en-1-yl)acetamide (3ad)

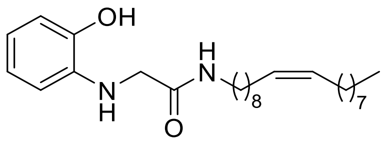

Brown oil; Yield: **33%**; **^{1}H NMR (400 MHz, $CDCl_3$)** *δ* 7.02-6.94 (1H, m, NH), 6.90 (1H, br s, NH), 6.81 (2H, t, *J* = 8.0 Hz, ArH), 6.68 (1H, t, *J* = 8.0 Hz, ArH), 6.49 (1H, d, *J* = 8.0 Hz, ArH), 5.42-5.29 (2H, m, 2 x =CH), 4.70 (1H, br s, OH), 3.80 (2H, s, NCH_2), 3.31-3.23 (2H, m, NCH_2), 2.06-1.93 (4H, m, 2 x CH_2), 1.50-1.42 (2H, m, CH_2), 1.36-1.19 (22H, m, 11 x CH_2), 0.88 (3H, t, *J* = 6.1 Hz, CH_3); **^{13}C NMR (100 MHz, $CDCl_3$)** *δ* 171.5, 143.9, 136.2, 129.9, 129.8, 121.1, 118.9, 114.4, 111.6, 49.0, 39.3, 32.6, 31.9, 29.7, 29.7, 29.7, 29.6, 29.5, 29.5, 29.4, 29.3, 29.2, 27.2, 27.2, 26.8, 22.7, 14.1; **HRMS (ESI$^+$):** *m/z* calcd for $C_{26}H_{44}N_2NaO_2^+$: 439.3295; [M+Na]$^+$ found: 439.3294.

Ethyl 2-(2-((2-hydroxyphenyl)amino)acetamido)acetate (3ae)

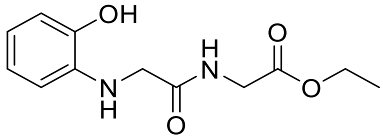

Yellowish solid; Yield: **17%**; **m.p.:** 82-84 °C; **^{1}H NMR (400 MHz, CDCl$_3$)** δ 7.41 (1H, s, OH), 6.84-6.76 (2H, m, ArH), 6.66 (1H, t, J = 7.8 Hz, ArH), 6.54 (1H, d, J = 7.8 Hz, ArH), 4.17 (2H, q, J = 6.7 Hz, OCH$_2$), 4.04 (2H, d, J = 5.7 Hz, NCH$_2$), 3.84 (2H, s, NCH$_2$), 1.25 (3H, t, J = 6.7 Hz, CH$_3$); **^{13}C NMR (100 MHz, CDCl$_3$)** δ 172.2, 170.0, 143.8, 136.1, 121.3, 119.0, 114.6, 111.9, 61.7, 48.7, 41.0, 14.0; **HRMS (ESI$^+$):** *m/z* calcd for $C_{12}H_{16}N_2NaO_4^+$: 275.1002; [M+Na]$^+$ found: 275.1001.

Methyl (2-hydroxyphenyl)glycylleucinate (3af)[1]

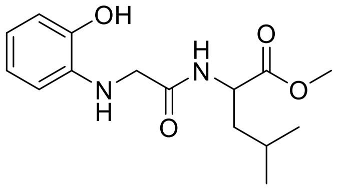

Yellow low melting ponting solid; Yield: **25%**; **^{1}H NMR (400 MHz, CDCl$_3$)** δ 7.33-7.25 (1H, m, NH), 6.87 (1H, br s, NH), 6.83-6.73 (2H, m, ArH), 6.65 (1H, t, J = 7.8 Hz, ArH), 6.50 (1H, d, J = 7.8 Hz, ArH), 5.09-4.36 (2H, br m, NCH and OH), 3.82 (2H, d, J = 5.3 Hz, NCH$_2$), 3.69 (3H, s, OCH$_3$), 1.65-1.46 (3H, m, CH$_2$ and CH), 0.88 (3H, d, J = 6.0 Hz, CH$_3$), 0.85 (3H, d, J = 6.0 Hz, CH$_3$); **^{13}C NMR (100 MHz, CDCl$_3$)** δ 173.5, 172.2, 144.1, 136.3, 121.1, 119.1, 114.6, 111.9, 52.5, 50.6, 49.0, 41.0, 24.9, 22.9, 21.7; **HRMS (ESI$^+$):** *m/z* calcd for $C_{15}H_{22}N_2NaO_4^+$: 317.1472; [M+Na]$^+$ found: 317.1474.

Further Functionalization of Benzoxazole Derivatives

Synthesis of *N*-(1-Phenylethyl)benzo[*d*]oxazole-2-carboxamide (36)[16]

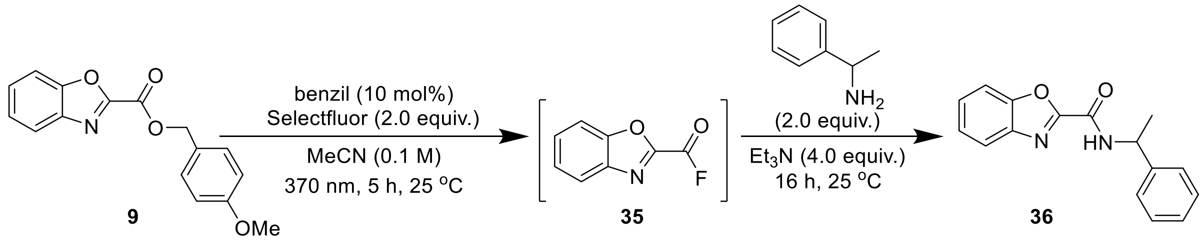

A test tube was charged with PMB-ester **9** (28 mg, 0.10 mmol, 1.00 equiv.), benzil (2 mg, 0.01 mmol, 0.1 equiv.) and Selectfluor (71 mg, 0.20 mmol, 2.00 equiv.) in anhydrous acetonitrile (1 mL). The tube was sealed with a septum and was degassed. Then, the tube was flushed with argon and the reaction mixture was stirred at 25 °C under 370 nm LED light irradiation for 5.5 h. Subsequently, the reaction mixture was cooled at 0 °C and sequentially treated with 1-phenylethylamine (26 μL, 0.20 mmol, 2.00 equiv.) and triethylamine (56 μL, 0.40 mmol, 4.00 equiv.). The reaction mixture was then stirred at 25 °C for an additional 16 h. The reaction mixture was quenched with water (3 mL), extracted with ethyl acetate (3 x 3 mL), dried over Na_2SO_4, and concentrated under reduced pressure. The resulting residue was purified by flash column chromatography on silica gel using a gradient of petroleum ether and ethyl acetate (80:20) as the eluent. White solid; Yield: **63%; m.p.:** 66-68 °C; **^{1}H NMR** (400 MHz, $CDCl_3$): δ 7.77 (1H, d, J = 8.0 Hz, ArH), 7.65 (1H, d, J = 8.0 Hz, ArH), 7.56-7.27 (8H, m, ArH and NH), 5.43-5.29 (1H, m, NCH), 1.66 (3H, d, J = 6.8 Hz, CH_3); **^{13}C NMR** (100 MHz, $CDCl_3$): δ 155.5, 154.7, 151.1, 142.0, 140.1, 128.8, 127.8, 127.3, 126.3, 125.5, 121.1, 111.8, 49.4, 21.6; **HRMS (ESI$^+$):** *m/z* calcd for $C_{16}H_{15}N_2O_2^+$: 267.1128; $[M+H]^+$ found: 267.1128.

General Procedure for the Click Reaction Between Terminal Alkyne Benzoxazole Derivatives and Azides

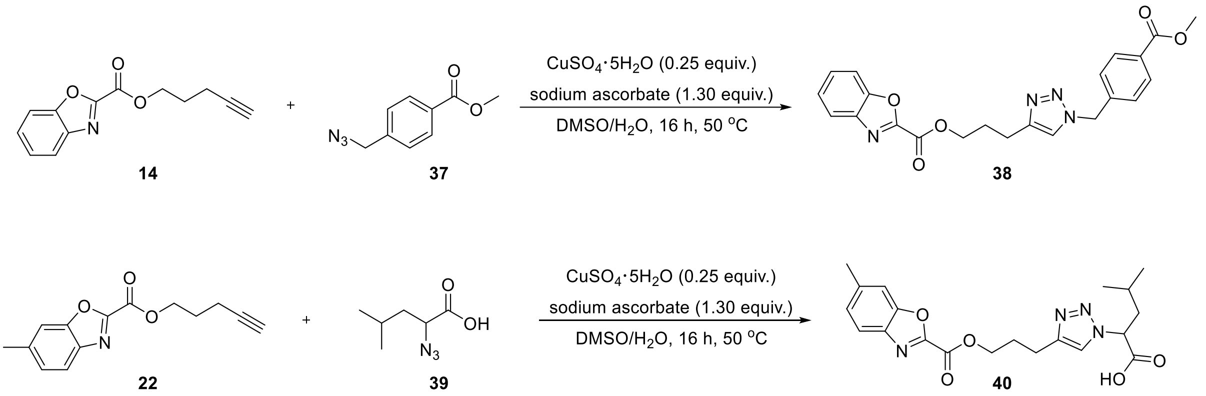

A mixture of alkyne **14** or **22** (0.06 mmol, 1.00 equiv.), the appropriate azide (0.19 mmol, 3.20 equiv.), $CuSO_4{\cdot}5H_2O$ (3.7 mg, 0.015 mmol, 0.25 equiv.) and sodium ascorbate (15.8 mg, 0.08 mmol, 1.30 equiv.) in DMSO (6 mL) and water (0.75 mL) was stirred for 18 h at 50 °C. Then, the reaction mixture was poured into brine (10 mL) and extracted with EtOAc (3 x 5 mL). The organic layer was separated, washed with water (5 mL) and brine (5 mL) and dried over Na_2SO_4. The organic layer was separated and dried over Na_2SO_4. The solvent was evaporated under reduced pressure and the residue was purified by flash column chromatography (Pet. Ether:EtOAc 20:80 in the first case or CH_2Cl_2:MeOH 90:10-80:20 in the second one).

3-(1-(4-(Methoxycarbonyl)benzyl)-1*H*-1,2,3-triazol-4-yl)propyl benzo[*d*]oxazole-2-carboxylate (38)

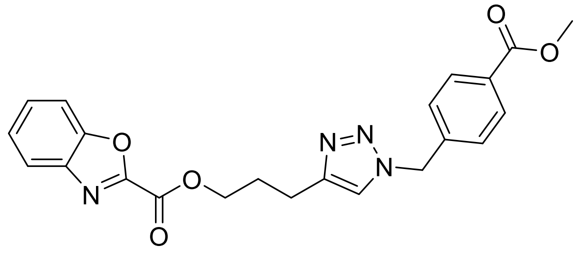

White solid; Yield: **83%**; **m.p.:** 109-110 °C; **^{1}H NMR (400 MHz, $CDCl_3$)** δ 8.01 (2H d, J = 8.0 Hz, ArH), 7.87 (1H, d, J = 8.0 Hz, ArH), 7.64 (1H, d, J = 8.0 Hz, ArH), 7.52 (1H, t, J = 8.0 Hz, ArH), 7.44 (1H, t, J = 8.0 Hz, ArH), 7.34 (1H, s, triazole-H), 7.29 (2H, d, J = 8.0 Hz, ArH), 5.55 (2H, s, CH_2Ph), 4.52 (2H, t, J = 6.8 Hz, OCH_2), 3.90 (3H, s, OCH_3), 2.91 (2H, t, J = 6.8 Hz, CH_2), 2.25 (2H, quin, J = 6.8 Hz, CH_2); **^{13}C NMR (100 MHz, $CDCl_3$)** δ 166.3, 156.5, 152.5, 150.8, 147.1, 140.5, 139.6, 130.5, 130.3, 128.2, 127.7, 125.8, 122.1, 121.2,

111.7, 66.1, 53.6, 52.2, 28.0, 22.0; **HRMS (ESI$^+$):** *m/z* calcd for $C_{22}H_{21}N_4O_5^+$: 421.1506; $[M+H]^+$ found: 421.1506.

4-Methyl-2-(4-(3-((6-methylbenzo[*d*]oxazole-2-carbonyl)oxy)propyl)-1*H*-1,2,3-triazol-1-yl)pentanoic acid (40)

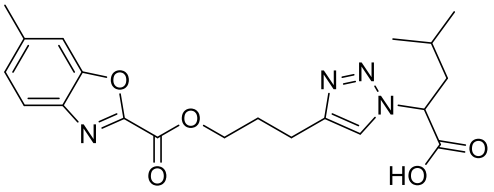

White solid; Yield: **80%**; **m.p.:** 114-116 °C; **^{1}H NMR (400 MHz, CD$_3$OD)** δ 7.83 (1H, s, triazole-H), 7.72 (1H, d, *J* = 8.0 Hz, ArH), 7.55 (1H, s, ArH), 7.34 (1H, d, *J* = 8.0 Hz, ArH), 5.16 (1H, dd, *J* = 9.4 and 6.8 Hz, NCH), 3.60 (2H, t, *J* = 6.8 Hz, OCH$_2$), 2.77 (2H, t, *J* = 6.8 Hz, CH$_2$), 2.53 (3H, s, CH$_3$), 2.09-2.00 (2H, m, CH$_2$), 1.89 (2H, quin, *J* = 6.8 Hz, CH$_2$), 1.30-1.21 (1H, m, C*H*(CH$_3$)$_2$), 0.94 (3H, d, *J* = 6.6 Hz, CH$_3$), 0.88 (3H, d, *J* = 6.6 Hz, CH$_3$); **^{13}C NMR (100 MHz, CD$_3$OD)** δ 176.5, 158.0, 154.0, 152.5, 148.2, 140.9, 139.3, 128.6, 122.7, 121.9, 112.6, 65.8, 62.0, 43.0, 33.3, 26.2, 23.2, 22.8, 21.9, 21.6; **HRMS (ESI$^+$):** *m/z* calcd for $C_{20}H_{24}N_4NaO_5^+$: 423.1639; $[M+Na]^+$ found: 423.1639.

Synthesis of 1-(Benzo[*d*]oxazol-2-yl)heptan-1-one (41)

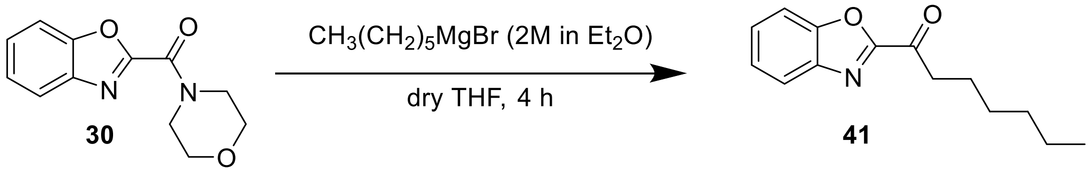

To a flame-dried flask containing benzo[*d*]oxazol-2-yl(morpholino)methanone **30** (10 mg, 0.04 mmol, 1.00 equiv.) dissolved in dry THF (4 mL) under an inert atmosphere, hexylmagnesium bromide (2 M in diethyl ether, 0.06 mL, 0.12 mmol, 3.00 equiv.) was added dropwise at 0 °C. The reaction mixture was then allowed to warm to room temperature and stirred for 4 hours. After reaction completion, a saturated aqueous solution of NH_4Cl (5 mL) was added. The aqueous phase was extracted with diethyl ether (3 x 5 mL), and the combined

organic layers were washed with brine (1 x 5 mL), dried over Na_2SO_4, filtered, and concentrated under reduced pressure. Purification of the crude product by flash column chromatography (Pet. Ether:EtOAc, 95:5-90:10) afforded the desired compound. Colorless oil; Yield: **52%**; **^{1}H NMR (400 MHz, $CDCl_3$)** δ 7.90 (1H d, J = 8.0 Hz, ArH), 7.66 (1H, d, J = 8.0 Hz, ArH), 7.53 (1H, t, J = 8.0 Hz, ArH), 7.46 (1H, t, J = 8.0 Hz, ArH), 3.22 (2H, t, J = 7.6 Hz, $COCH_2$), 1.81 (2H, quin, J = 7.6 Hz, CH_2), 1.46-1.29 (6H, m, 3 x CH_2), 0.89 (3H, t, J = 6.6 Hz, CH_3); **^{13}C NMR (100 MHz, $CDCl_3$)** δ 190.5, 157.2, 150.7, 140.5, 128.5, 125.7, 122.2, 111.9, 39.5, 31.5, 28.8, 23.8, 22.5, 14.0; **HRMS (ESI$^+$):** *m/z* calcd for $C_{14}H_{17}NNaO_2^+$: 254.1151; $[M+Na]^+$ found: 254.1151.

Direct Infusion-High Resolution Mass Spectrometry (DI-HRMS) Mechanistic Studies

Instrumentation

High Resolution Mass Spectra were recorded with a Q-TOF (Time of Flight Mass Spectrometer) Bruker Maxis Impact with electrospray ionization (ESI) source. N_2 was used as the collision gas and positive ionization mode was used for all MS experiments. The data acquisition was carried out with Data Analysis from Bruker Daltonics (version 4.1). Acetonitrile LC-MS gradient was obtained from Carlo Erba Reagents (Chaussée du Vexin, France). Source conditions: End plate offset 500V, Capillary 4500V, Nebulizer 0.4 bar, dry gas 4.0 L/min, dry temperature 180 ^{o}C and Quadrupole conditions: Ion energy 5 eV, Collision energy 10 eV, Transfer time 143 μs, Collision ion RF 3500 vpp, Pre pulse storage 1μs. The annotation of the intermediates was based on the exact mass high accuracy (mass error lower than 5 ppm) and in most cases on the isotopic distribution similarity (mSigma values lower than 50).

Study for the Identification of Intermediates Trapped by Radical Quencher TEMPO in the Photochemical Synthesis of Benzoxazoles

A solution of the glycine derivative **3a** (19.5 mg, 0.10 mmol, 1.00 equiv.) in acetonitrile (1 mL) was treated with CuI (1.9 mg, 0.01 mmol, 0.10 equiv.), 1,8-dihydroxyanthraquinone (**4c**) (4.8 mg, 0.02 mmol, 0.20 equiv.) and TEMPO (21.9 mg, 0.14 mmol, 1.40 equiv.). The reaction mixture was stirred at room temperature for 18 h under air and LED (Kessil PR160L, 427 nm) irradiation. After reaction completion, a sample of the reaction mixture (20 μL) was first diluted with 980 μL ACN and 100 μL of that sample were further diluted with 900 μL of ACN. Finally, 100 μL were injected for DI-HRMS analysis.

A suspect analysis approach was carried out, which enabled us to observe ions corresponding to intermediates formed by TEMPO-trapped radicals.

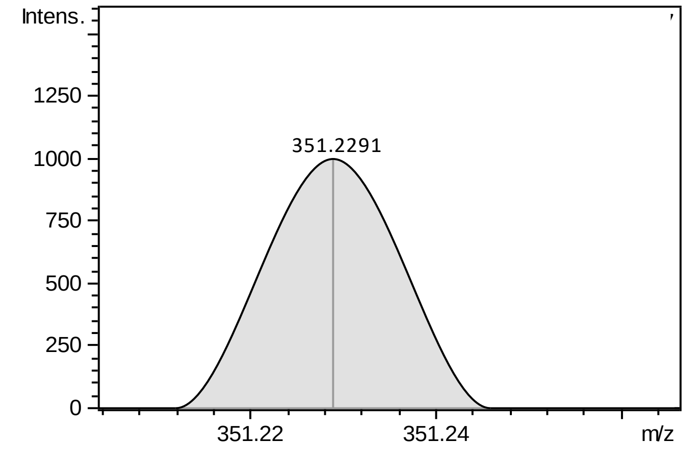

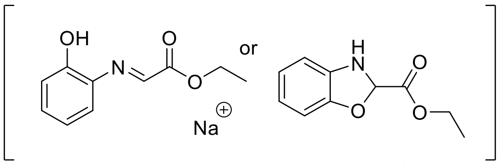

Chemical Formula: $C_{10}H_{11}NNaO_3^+$
Exact Mass: 216.0631

Meas. m/z	#	Ion Formula	m/z	err [ppm]	mSigma	# Sigma	Score	rdb	e⁻ Conf	N-Rule
351.2291	1	C19H31N2O4	351.2278	-3.6	18.0	1	100.00	5.5	even	ok

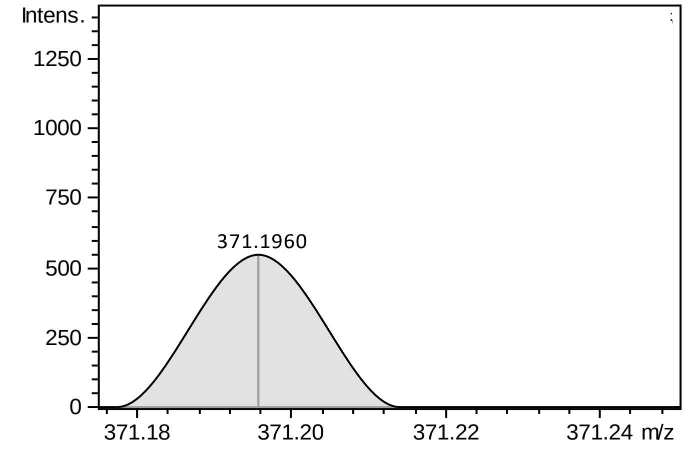

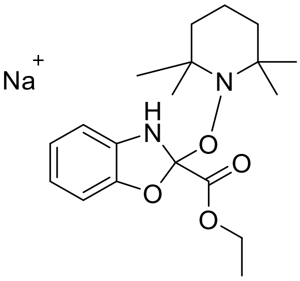

Chemical Formula: $C_{19}H_{28}N_2NaO_4^+$
Exact Mass: 371.1941

Meas. m/z	#	Ion Formula	m/z	err [ppm]	mSigma	# Sigma	Score	rdb	e⁻ Conf	N-Rule
371.1960	1	C19H28N2NaO4	371.1941	5.0	n.a.	1	-1.#J	6.5	even	ok

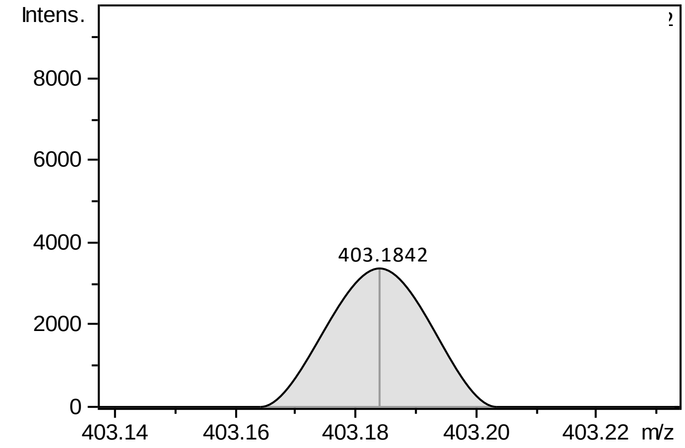

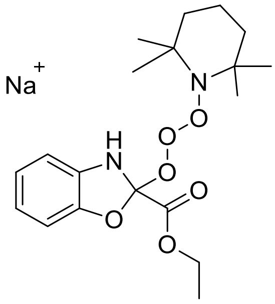

Chemical Formula: $C_{19}H_{28}N_2NaO_6^+$
Exact Mass: 403.1840

Meas. m/z	#	Ion Formula	m/z	err [ppm]	mSigma	# Sigma	Score	rdb	e⁻ Conf	N-Rule
403.1842	1	C19H28N2NaO6	403.1840	0.5	42.8	1	100.00	6.5	even	ok

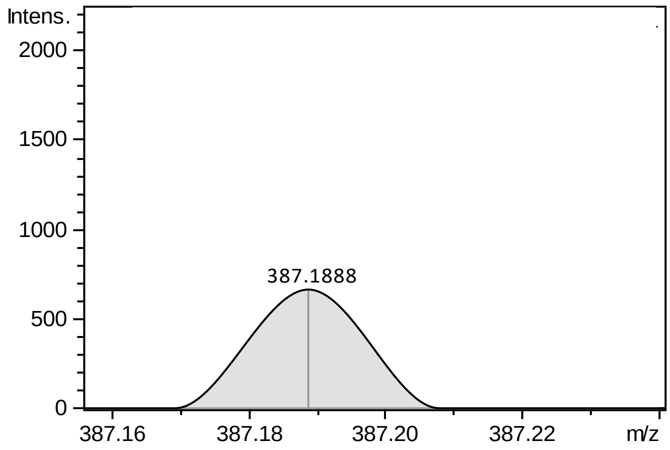

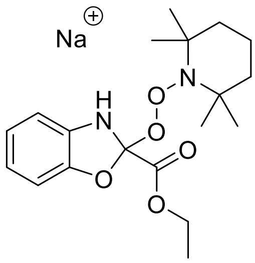

Chemical Formula: $C_{19}H_{28}N_2NaO_5^+$
Exact Mass: 387.1890

Meas. m/z	#	Ion Formula	m/z	err [ppm]	mSigma	# Sigma	Score	rdb	e⁻ Conf	N-Rule
387.1888	1	C19H28N2NaO5	387.1890	-0.6	n.a.	1	-1.#J	6.5	even	ok

Study of the Photochemical Synthesis of Benzoxazoles

A solution of the glycine derivative **3a** (19.5 mg, 0.10 mmol, 1.00 equiv.) in acetonitrile (1 mL) was treated with CuI (1.9 mg, 0.01 mmol, 0.10 equiv.) and 1,8-dihydroxyanthraquinone (**4c**) (4.8 mg, 0.02 mmol, 0.20 equiv.). The reaction mixture was stirred at room temperature under air and LED (Kessil PR160L, 427 nm) irradiation. At the specific time of study, a sample of the reaction mixture (10 μL) was first diluted with 990 μL ACN and 100 μL of that sample were further diluted with 900 μL of ACN. Finally, 100 μL were injected for analysis.

Following a suspect analysis approach, we observed ions, which can be attributed to benzoxazole **1**, as well as intermediates **I-VI**. Next, the High-Resolution Mass Spectra of ions observed in positive mode are presented for the following time points: (A) 30 min, (B) 1 h, (C) 2 h, (D) 3 h, and (E) 4 h.

Product and intermediates observed after 30 min irradiation:

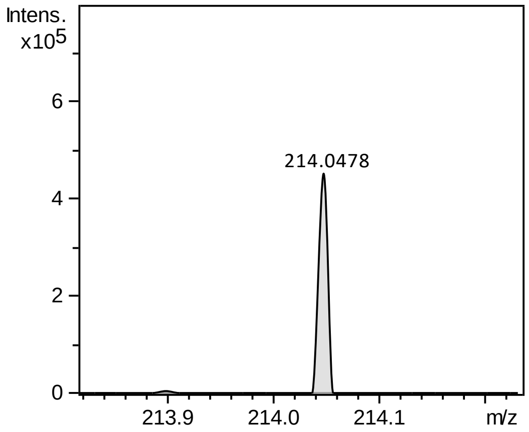

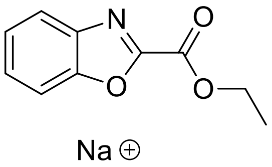

Chemical Formula: $C_{10}H_9NNaO_3^+$
Exact Mass: 214.0475

Meas. m/z	#	Ion Formula	m/z	err [ppm]	mSigma	# Sigma	Score	rdb	e⁻ Conf	N-Rule
214.0478	1	C10H9NNaO3	214.0475	-1.6	10.6	1	100.00	6.5	even	ok

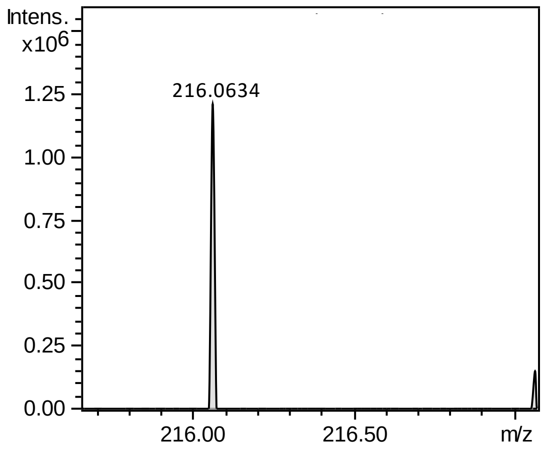

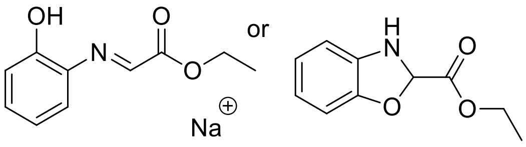

Chemical Formula: $C_{10}H_{11}NNaO_3^+$
Exact Mass: 216.0631

Meas. m/z	#	Ion Formula	m/z	err [ppm]	mSigma	# Sigma	Score	rdb	e$^-$ Conf	N-Rule
216.0634	1	C10H11NNaO3	216.0631	1.5	7.8	1	100.00	5.5	even	ok

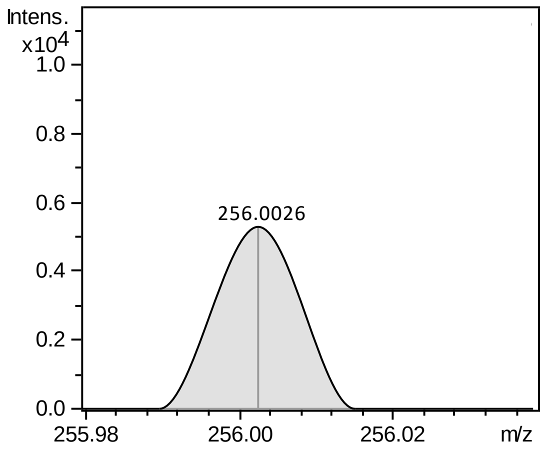

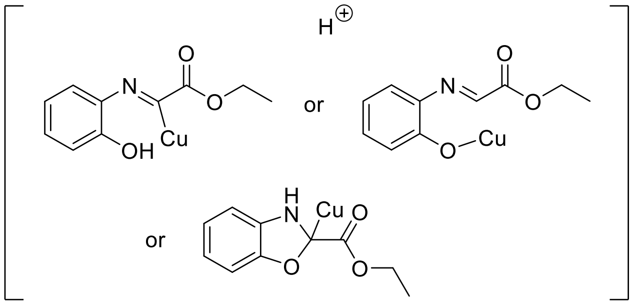

Chemical Formula: $C_{10}H_{11}CuNO_3^+$
Exact Mass: 256.0029

Meas. m/z	#	Ion Formula	m/z	err [ppm]	mSigma	# Sigma	Score	rdb	e$^-$ Conf	N-Rule
256.0026	1	C10H11CuNO3	256.0029	-1.3	206.3	1	100.00	5.5	even	ok

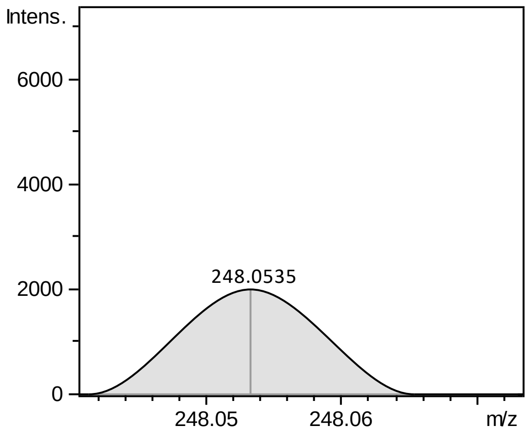

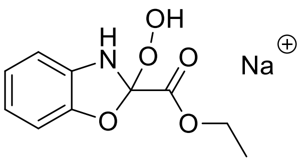

Chemical Formula: $C_{10}H_{11}NNaO_5^+$
Exact Mass: 248.0529

Meas. m/z	#	Ion Formula	m/z	err [ppm]	mSigma	# Sigma	Score	rdb	e$^-$ Conf	N-Rule
248.0535	1	C10H11NNaO5	248.0529	-2.2	16.9	1	100.00	5.5	even	ok

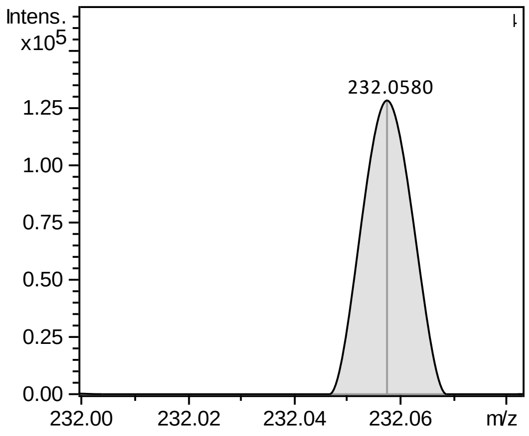

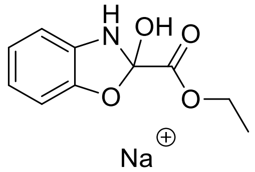

Chemical Formula: $C_{10}H_{11}NNaO_4^+$
Exact Mass: 232.0580

Meas. m/z	#	Ion Formula	m/z	err [ppm]	mSigma	# Sigma	Score	rdb	e⁻ Conf	N-Rule
232.0580	1	C10H11NNaO4	232.0580	-0.1	8.4	1	100.00	5.5	even	ok

Product and intermediates observed after 1 h irradiation:

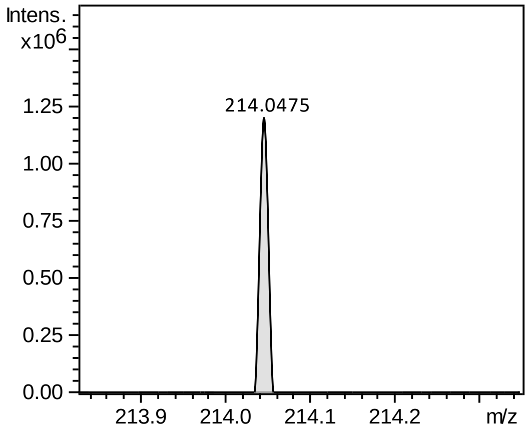

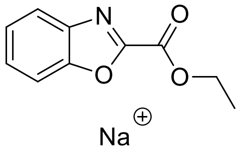

Chemical Formula: $C_{10}H_9NNaO_3^+$
Exact Mass: 214.0475

Meas. m/z	#	Ion Formula	m/z	err [ppm]	mSigma	# Sigma	Score	rdb	e⁻ Conf	N-Rule
214.0475	1	C10H9NNaO3	214.0475	0.1	11.7	1	100.00	6.5	even	ok

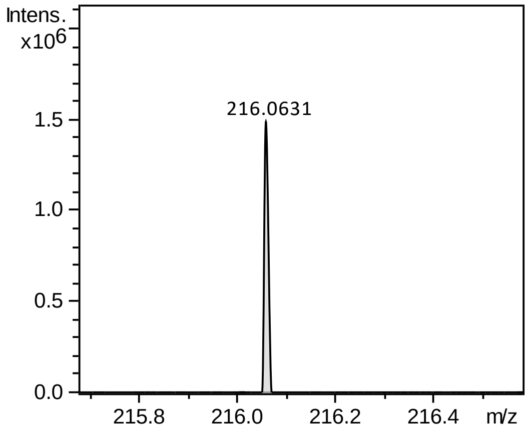

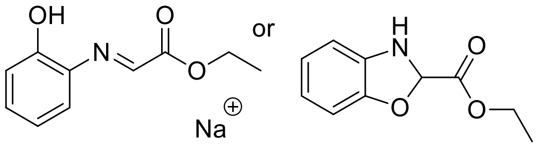

Chemical Formula: $C_{10}H_{11}NNaO_3^+$
Exact Mass: 216.0631

Meas. m/z	#	Ion Formula	m/z	err [ppm]	mSigma	# Sigma	Score	rdb	e⁻ Conf	N-Rule
216.0631	1	C10H11NNaO3	216.0631	-0.1	7.4	1	100.00	5.5	even	ok

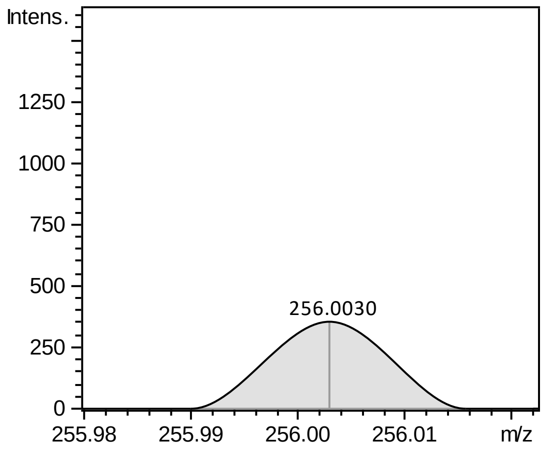

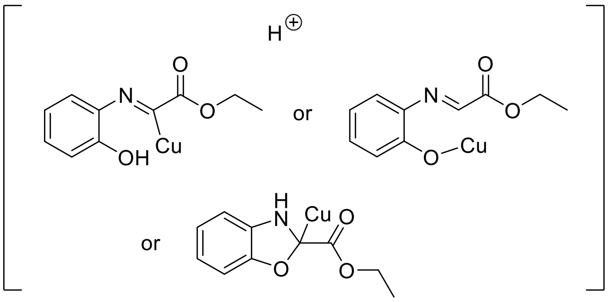

Chemical Formula: $C_{10}H_{11}CuNO_3^{+}$
Exact Mass: 256.0029

Meas. m/z	#	Ion Formula	m/z	err [ppm]	mSigma	# Sigma	Score	rdb	e⁻ Conf	N-Rule
256.0030	1	C10H11CuNO3	256.0029	0.2	n.a.	1	-1.#J	5.5	even	ok

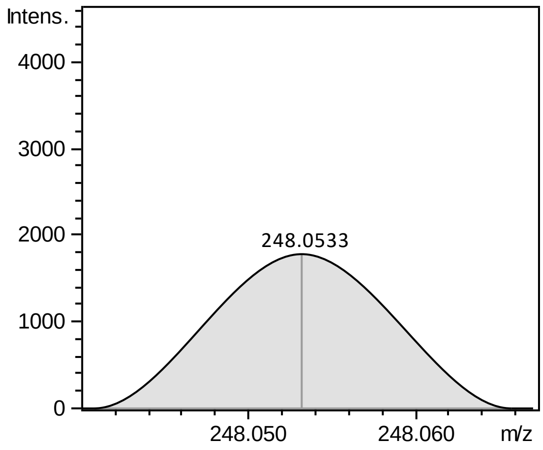

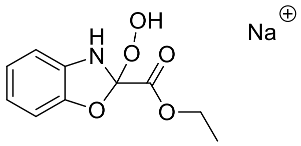

Chemical Formula: $C_{10}H_{11}NNaO_5^{+}$
Exact Mass: 248.0529

Meas. m/z	#	Ion Formula	m/z	err [ppm]	mSigma	# Sigma	Score	rdb	e⁻ Conf	N-Rule
248.0533	1	C10H11NNaO5	248.0529	1.5	14.8	1	100.00	5.5	even	ok

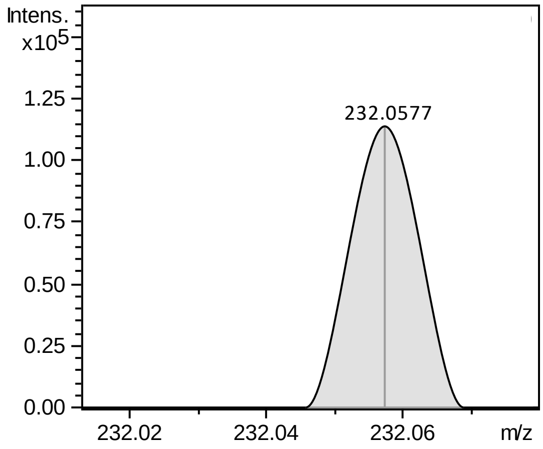

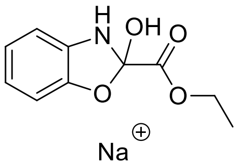

Chemical Formula: $C_{10}H_{11}NNaO_4^{+}$
Exact Mass: 232.0580

Meas. m/z	#	Ion Formula	m/z	err [ppm]	mSigma	# Sigma	Score	rdb	e⁻ Conf	N-Rule
232.0577	1	C10H11NNaO4	232.0580	1.3	11.1	1	100.00	5.5	even	ok

Product and intermediates observed after 2 h irradiation:

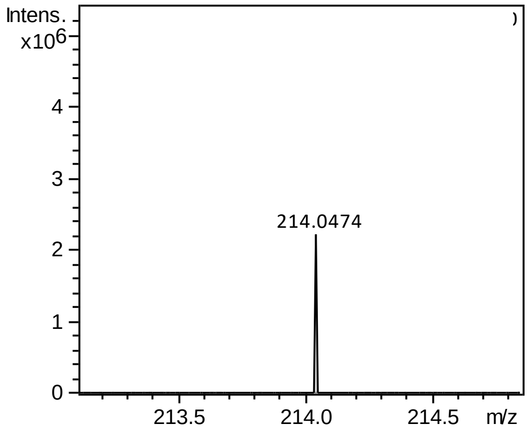

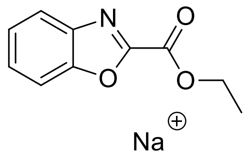

Chemical Formula: $C_{10}H_9NNaO_3^+$
Exact Mass: 214.0475

Meas. m/z	#	Ion Formula	m/z	err [ppm]	mSigma	# Sigma	Score	rdb	e⁻ Conf	N-Rule
214.0474	1	C10H9NNaO3	214.0475	0.4	10.9	1	100.00	6.5	even	ok

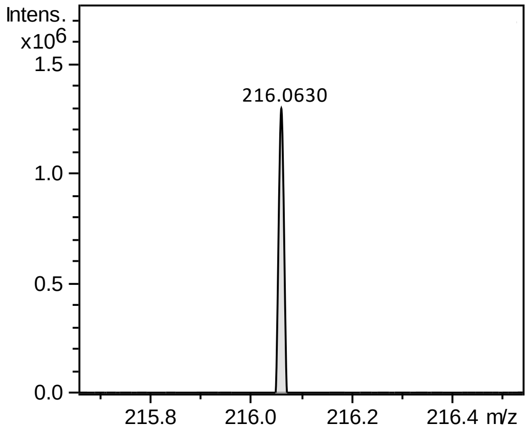

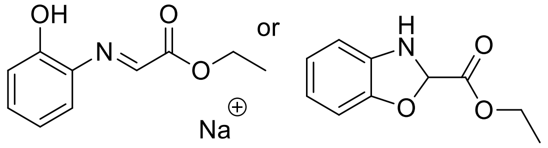

Chemical Formula: $C_{10}H_{11}NNaO_3^+$
Exact Mass: 216.0631

Meas. m/z	#	Ion Formula	m/z	err [ppm]	mSigma	# Sigma	Score	rdb	e⁻ Conf	N-Rule
216.0630	1	C10H11NNaO3	216.0631	-0.4	8.7	1	100.00	5.5	even	ok

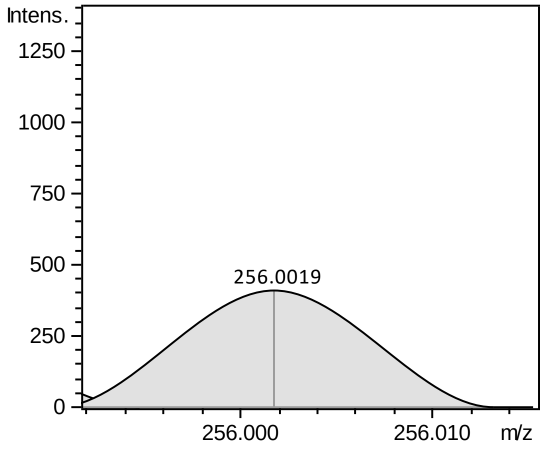

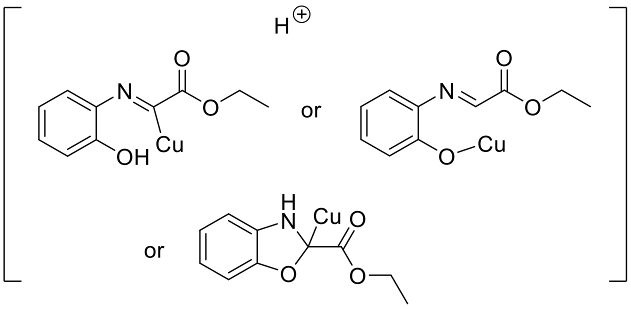

Chemical Formula: $C_{10}H_{11}CuNO_3^+$
Exact Mass: 256.0029

Meas. m/z	#	Ion Formula	m/z	err [ppm]	mSigma	# Sigma	Score	rdb	e⁻ Conf	N-Rule
256.0019	1	C10H11CuNO3	256.0029	4.2	n.a.	1	-1.#J	5.5	even	ok

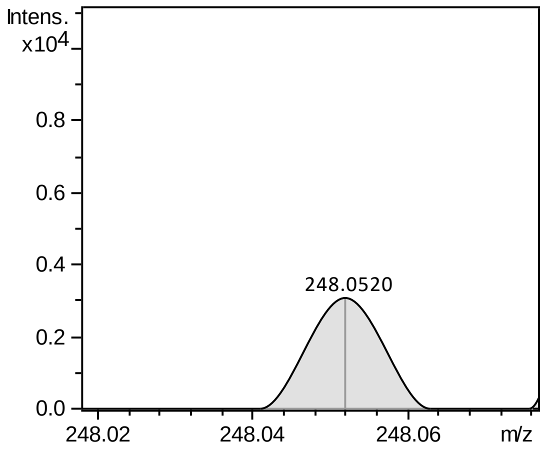

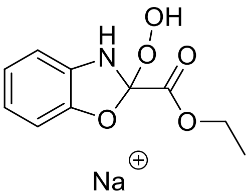

Chemical Formula: $C_{10}H_{11}NNaO_5^+$
Exact Mass: 248.0529

Meas. m/z	#	Ion Formula	m/z	err [ppm]	mSigma	# Sigma	Score	rdb	e⁻ Conf	N-Rule
248.0520	1	C10H11NNaO5	248.0529	3.6	17.4	1	100.00	5.5	even	ok

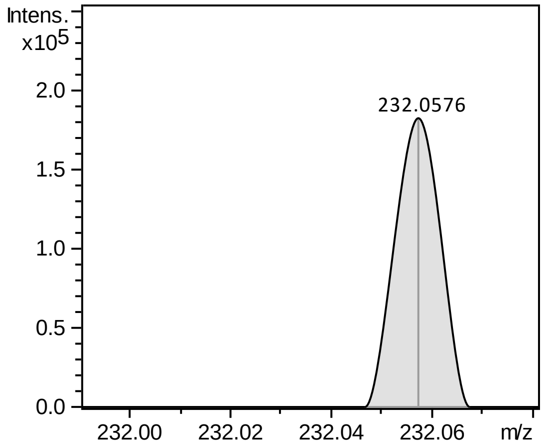

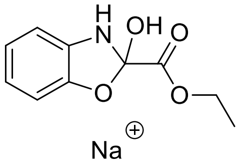

Chemical Formula: $C_{10}H_{11}NNaO_4^+$
Exact Mass: 232.0580

Meas. m/z	#	Ion Formula	m/z	err [ppm]	mSigma	# Sigma	Score	rdb	e⁻ Conf	N-Rule
232.0576	1	C10H11NNaO4	232.0580	-1.8	11.2	5	100.00	5.5	even	ok

Product and intermediates observed after 3 h irradiation:

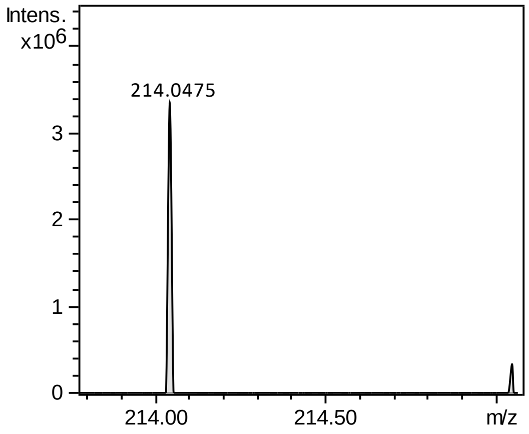

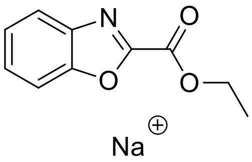

Chemical Formula: $C_{10}H_9NNaO_3^+$
Exact Mass: 214.0475

Meas. m/z	#	Ion Formula	m/z	err [ppm]	mSigma	# Sigma	Score	rdb	e⁻ Conf	N-Rule
214.0475	1	C10H9NNaO3	214.0475	-0.1	13.6	1	100.00	6.5	even	ok

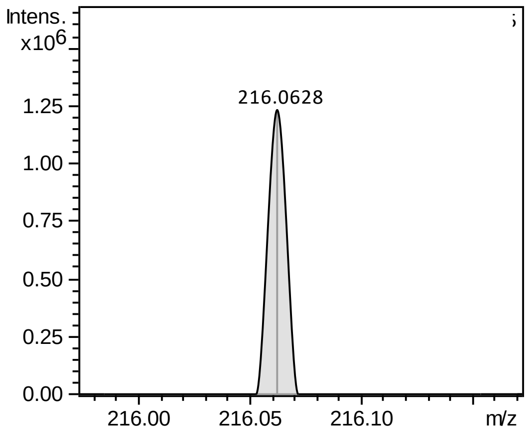

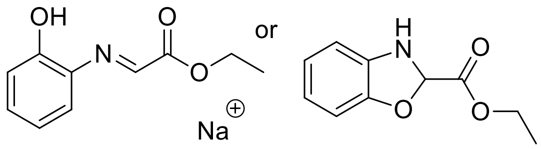

Chemical Formula: $C_{10}H_{11}NNaO_3^+$
Exact Mass: 216.0631

Meas. m/z	#	Ion Formula	m/z	err [ppm]	mSigma	# Sigma	Score	rdb	e$^-$ Conf	N-Rule
216.0628	1	C10H11NNaO3	216.0631	-1.5	7.1	1	100.00	5.5	even	ok

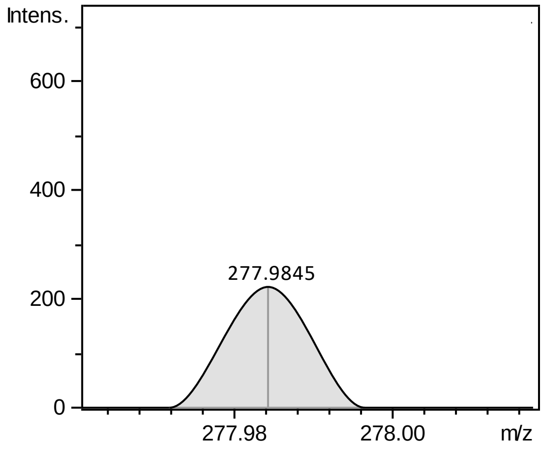

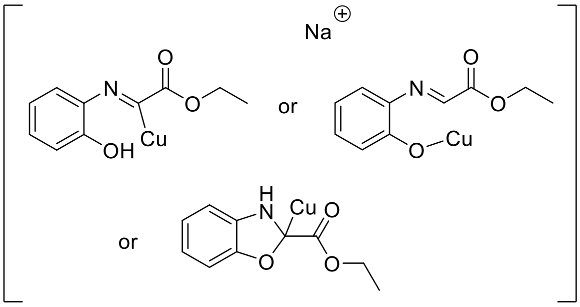

Chemical Formula: $C_{10}H_{10}CuNNaO_3^+$
Exact Mass: 277.9845

Meas. m/z	#	Ion Formula	m/z	err [ppm]	mSigma	# Sigma	Score	rdb	e$^-$ Conf	N-Rule
277.9845	1	C10H10CuNNaO3	277.9849	-1.4	n.a.	1	-1.#J	5.5	even	ok

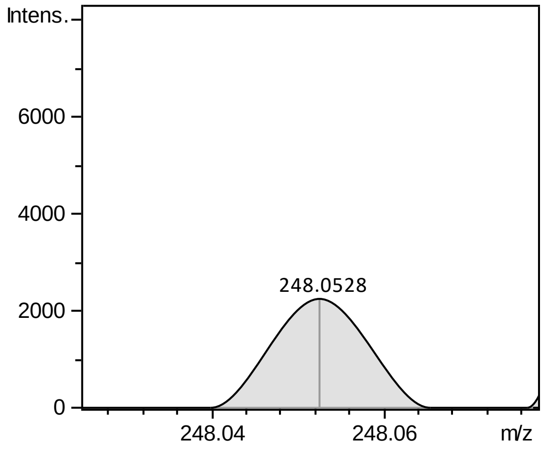

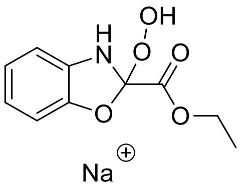

Chemical Formula: $C_{10}H_{11}NNaO_5^+$
Exact Mass: 248.0529

Meas. m/z	#	Ion Formula	m/z	err [ppm]	mSigma	# Sigma	Score	rdb	e$^-$ Conf	N-Rule
248.0528	1	C10H11NNaO5	248.0529	0.4	27.9	2	100.00	5.5	even	ok

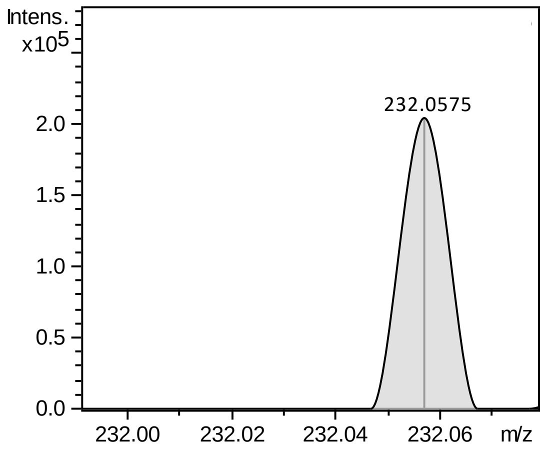

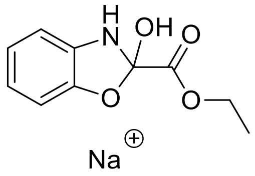

Chemical Formula: $C_{10}H_{11}NNaO_4^+$
Exact Mass: 232.0580

Meas. m/z	#	Ion Formula	m/z	err [ppm]	mSigma	# Sigma	Score	rdb	e⁻ Conf	N-Rule
232.0575	1	C10H11NNaO4	232.0580	-2.1	13.0	1	100.00	5.5	even	ok

Product and intermediates observed after 4 h irradiation:

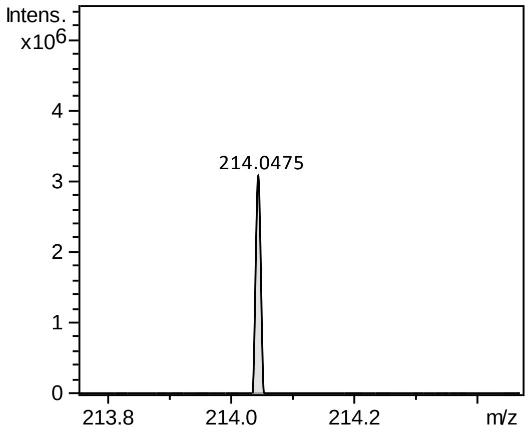

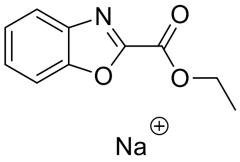

Chemical Formula: $C_{10}H_9NNaO_3^+$
Exact Mass: 214.0475

Meas. m/z	#	Ion Formula	m/z	err [ppm]	mSigma	# Sigma	Score	rdb	e⁻ Conf	N-Rule
214.0475	1	C10H9NNaO3	214.0475	0.2	11.3	1	100.00	6.5	even	ok

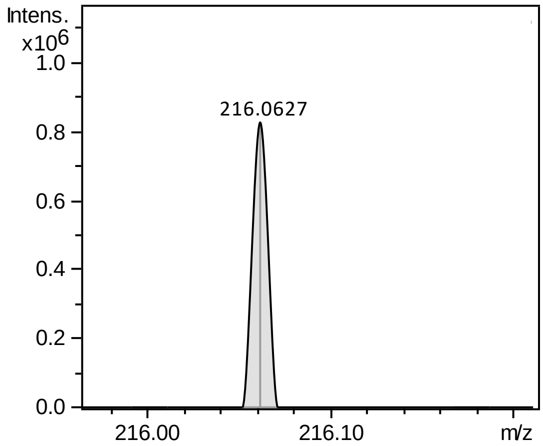

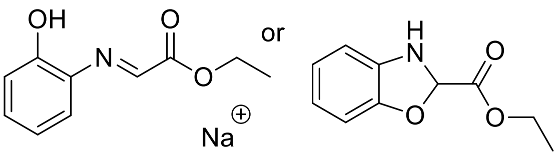

Chemical Formula: $C_{10}H_{11}NNaO_3^+$
Exact Mass: 216.0631

Meas. m/z	#	Ion Formula	m/z	err [ppm]	mSigma	# Sigma	Score	rdb	e⁻ Conf	N-Rule
216.0627	1	C10H11NNaO3	216.0631	2.0	7.1	1	100.00	5.5	even	ok

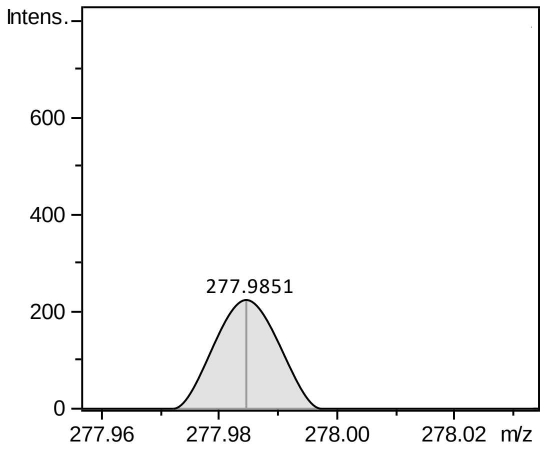

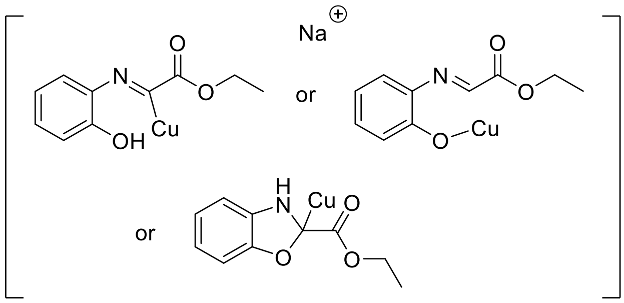

Chemical Formula: $C_{10}H_{10}CuNNaO_3^+$
Exact Mass: 277.9845

Meas. m/z	#	Ion Formula	m/z	err [ppm]	mSigma	# Sigma	Score	rdb	e¯ Conf	N-Rule
277.9851	1	C10H10CuNNaO3	277.9849	0.7	n.a.	1	-1.#J	5.5	even	ok

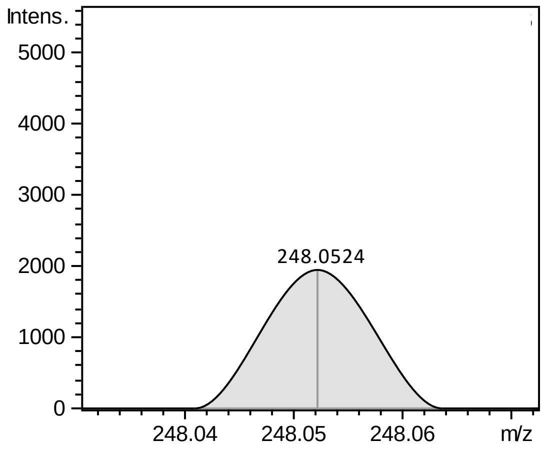

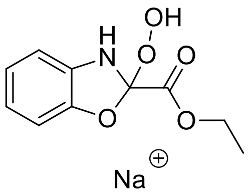

Chemical Formula: $C_{10}H_{11}NNaO_5^+$
Exact Mass: 248.0529

Meas. m/z	#	Ion Formula	m/z	err [ppm]	mSigma	# Sigma	Score	rdb	e¯ Conf	N-Rule
248.0524	1	C10H11NNaO5	248.0529	2.2	23.6	2	100.00	5.5	even	ok

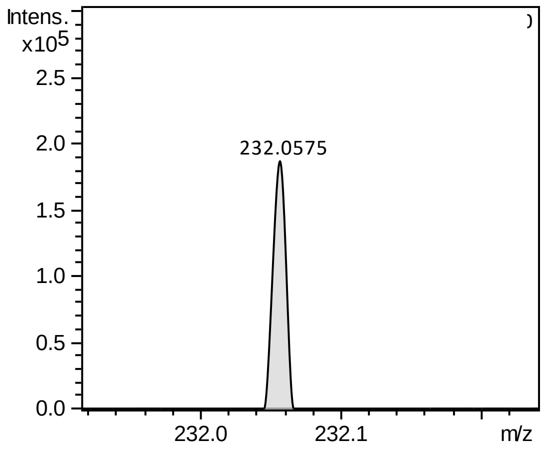

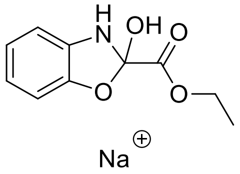

Chemical Formula: $C_{10}H_{11}NNaO_4^+$
Exact Mass: 232.0580

Meas. m/z	#	Ion Formula	m/z	err [ppm]	mSigma	# Sigma	Score	rdb	e¯ Conf	N-Rule
232.0575	1	C10H11NNaO4	232.0580	-2.3	16.0	1	100.00	5.5	even	ok

References

1. L. Liu, Z. Xu, T. Liu, C. Xu, W. Zhang, X. Hua, F. Ling, W. Zhong, *J. Org. Chem.* **2022**, *87*, 11379-11386.
2. Z.-Q. Zhu, S. Liu, Z.-Y. Hu, Z.-B. Xie, J. Tang, Z.-G. Le. *Adv. Synth. Catal.* **2021**, *363*, 2568-2572.
3. S. Liu, Z.-Q. Zhu, Z.-Y. Hu, J. Tang, E. Yuan, *Org. Biomol. Chem.* **2021**, *19*, 1616-1619.
4. a) J.-H. Chen, C.-H. Deng, S. Fang, J.-G. Ma, P. Cheng, *Green Chem.* **2018**, *20,* 989-996; b) N. Kawahara, T. Shimamori, T. Itoh, H. Ogura, *Heterocycles* **1986**, *24,* 2803-2807.
5. a) T. He, H. Li, P. Li, L. Wang, *Chem. Commun.* **2011**, *47*, 8946-8948; b) K. Dickoré, K. Sasse, K.-D. Bode, *Just. Lieb. Ann. Chem.* **1970**, *733,* 70-87.
6. Q. Xing, H. Lv, C. Xia, F. Li, *Chem. Commun.* **2017**, *53*, 6914-6917.
7. S. Morrissey, B. Pegot, D. Coleman, M. T. Garcia, D. Ferguson, B. Quilty, N. Gathergood, *Green Chem.* **2009**, *11*, 475-483.
8. C. Empel, S. Jana, Ł. W. Ciszewski, K. Zawada, C. Pei, D. Gryko, R. M. Koenigs, *Chem. Eur. J.* **2023**, 29, e202300214.
9. S.-K. Chen, W.-Q. Ma, Z.-B. Yan, F.-M. Zhang, S.-H. Wang, Y.-Q. Tu, X.-M. Zhang, J.-M. Tian, *J. Am. Chem. Soc.* **2018**, *140*, 10099-10103.
10. V. Steck, G. Sreenilayam, R. Fasan, *Synlett* **2020**, *31*, 224-229.
11. H.-S. Dang, M. R. J. Elsegood, K.-M. Kim, B. P. Roberts, *J. Chem. Soc., Perkin Trans. 1* **1999**, 2061-2068.
12. L. Deng, N. W. Boaz, (Eastman Chemical Company), US2012/0029198A1, 2012.
13. S.-H. Kwak, C. S. Cochrane, J. Cho, P. A. Dome, A. F. Ennis, J. H. Kim, P. Zhou, J. Hong, *ChemMedChem* **2023**, 18, e202300023.
14. N. Zidar, D. Kikelj, *Tetrahedron* **2008**, *64*, 5756-5761.
15. M. Shabpiray, A. Sharifi, M. Saeed Abaee, M. Mirzaei, N. Ghonouei, *Tetrahedron Lett.* **2023**, *126*, 154643.
16. Y. Zhang, R. Wang, C. Lu, C. Ma, F. Wang, G. Yang, Y. Zhang, J. Nie, *ACS Sustain. Chem. Eng.* **2024**, *12*, 16239-16248.

NMR Traces

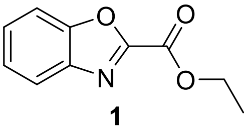

1

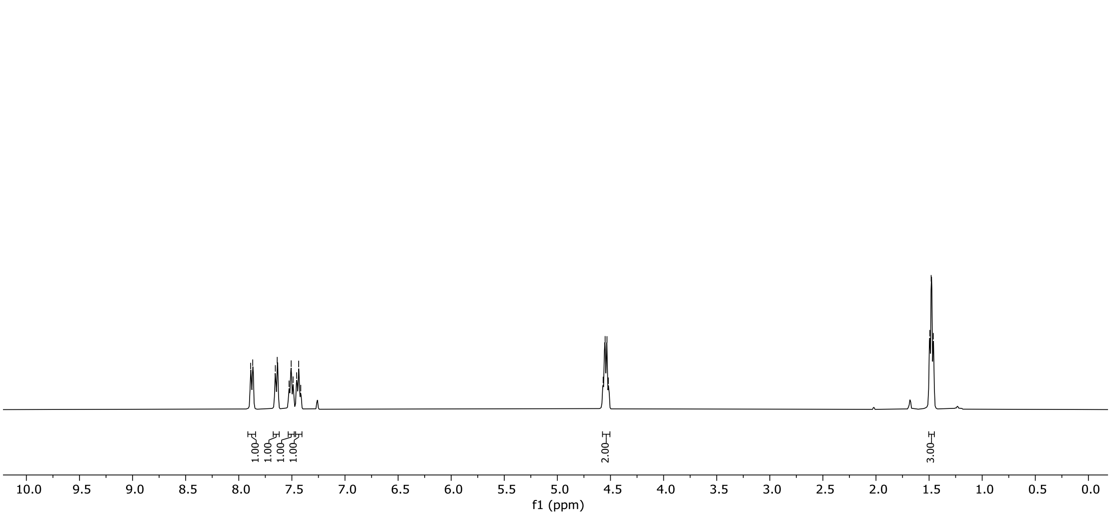
7.89 7.87 7.66 7.64 7.53 7.51 7.49 7.46 7.44 7.42
4.57 4.55 4.53 4.52
1.49 1.48 1.46
1.00 1.00 1.00 1.00
2.00
3.00
10.0 9.5 9.0 8.5 8.0 7.5 7.0 6.5 6.0 5.5 5.0 4.5 4.0 3.5 3.0 2.5 2.0 1.5 1.0 0.5 0.0
f1 (ppm)

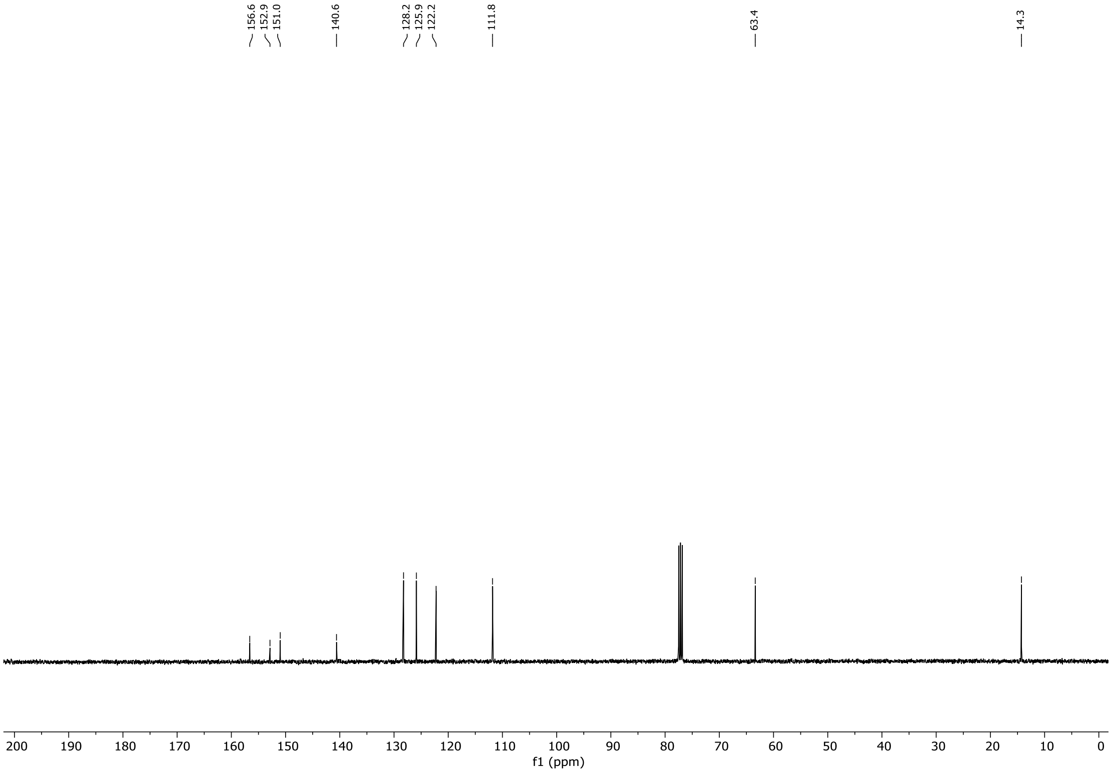
156.6 152.9 151.0
140.6
128.2 125.9 122.2
111.8
63.4
14.3
200 190 180 170 160 150 140 130 120 110 100 90 80 70 60 50 40 30 20 10 0
f1 (ppm)

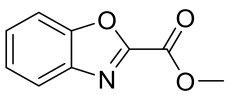

5

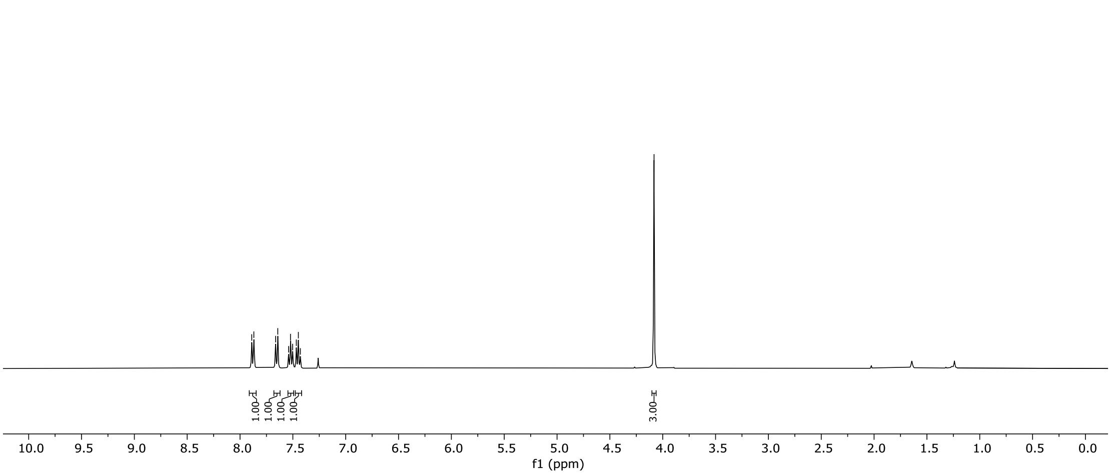
7.89
7.87
7.66
7.64
7.54
7.52
7.50
7.47
7.45
7.43
4.08
1.00
1.00
1.00
1.00
3.00
10.0 9.5 9.0 8.5 8.0 7.5 7.0 6.5 6.0 5.5 5.0 4.5 4.0 3.5 3.0 2.5 2.0 1.5 1.0 0.5 0.0
f1 (ppm)

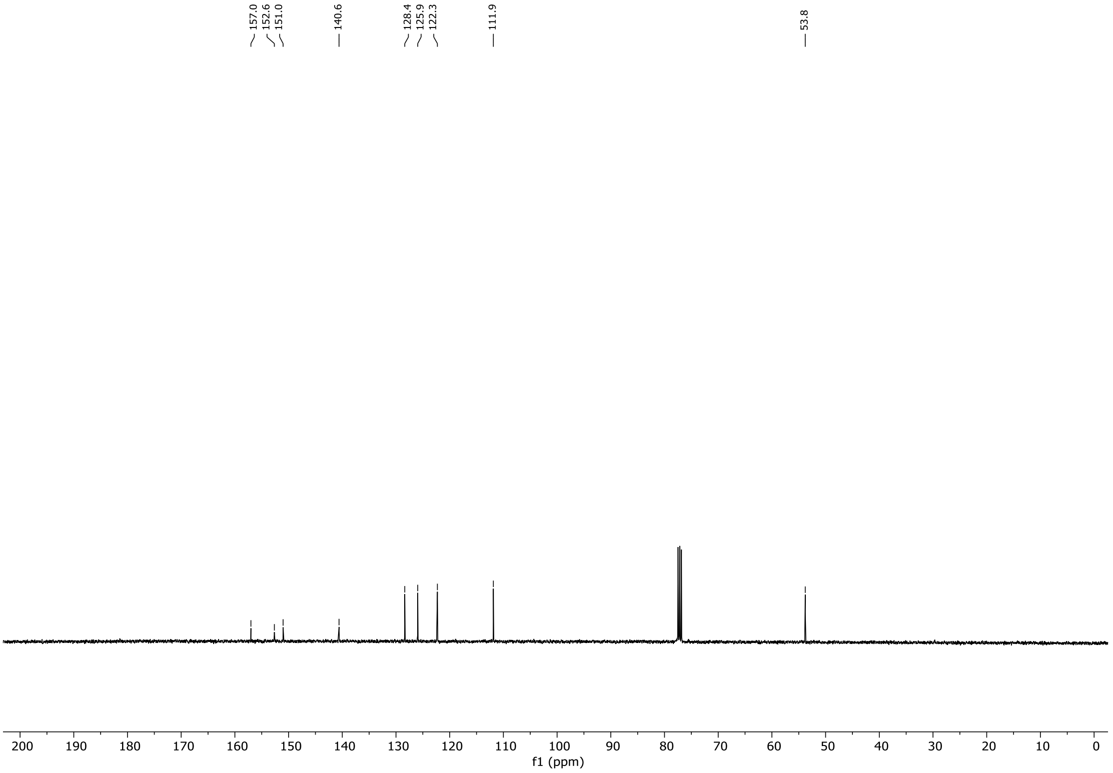
157.0
152.6
151.0
140.6
128.4
125.9
122.3
111.9
53.8
200 190 180 170 160 150 140 130 120 110 100 90 80 70 60 50 40 30 20 10 0
f1 (ppm)

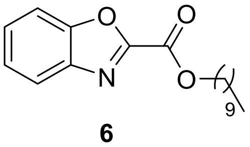
O
O
N
O
9

6

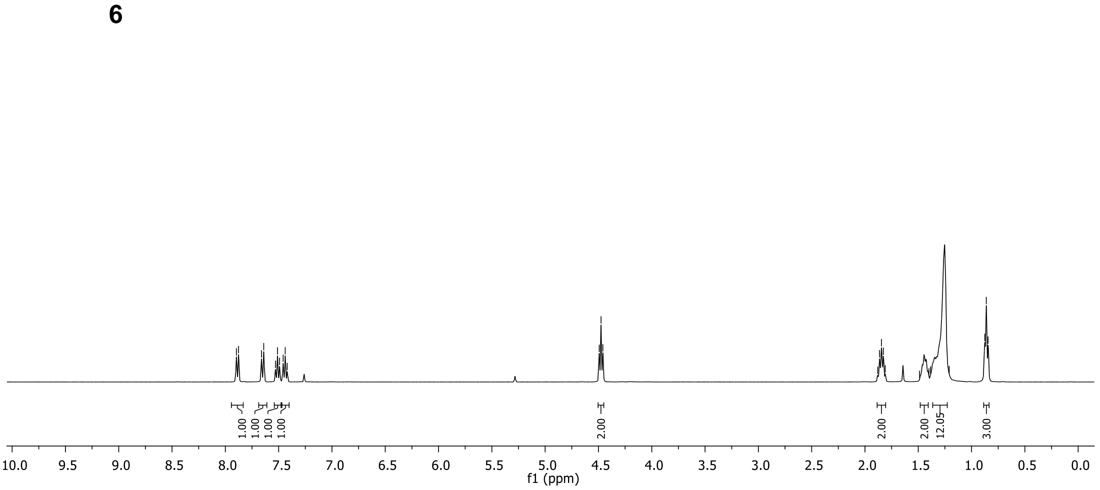
7.90
7.88
7.66
7.64
7.53
7.51
7.49
7.46
7.44
7.42
4.49
4.48
4.46
1.88
1.86
1.85
1.83
1.81
1.49
1.40
1.38
1.21
0.88
0.86
0.84
1.00
1.00
1.00
1.00
2.00
2.00
2.00
12.05
3.00
10.0 9.5 9.0 8.5 8.0 7.5 7.0 6.5 6.0 5.5 5.0 4.5 4.0 3.5 3.0 2.5 2.0 1.5 1.0 0.5 0.0
f1 (ppm)

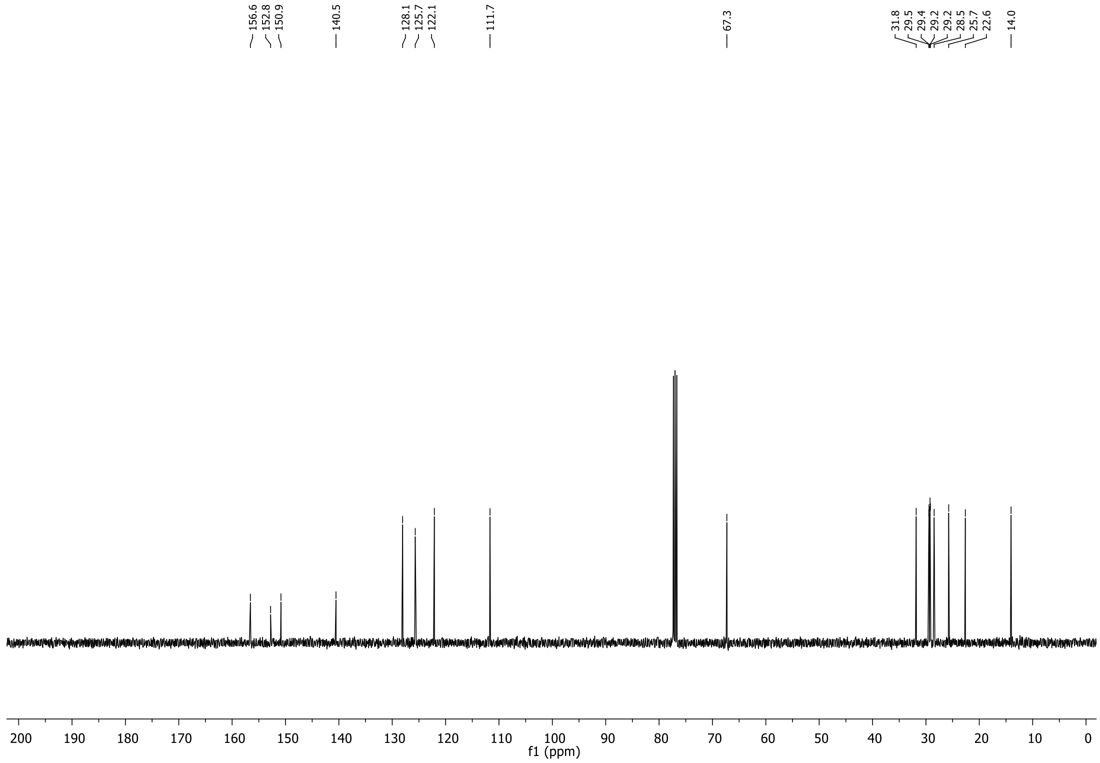
156.6
152.8
150.9
140.5
128.1
125.7
122.1
111.7
67.3
31.8
29.5
29.4
29.2
29.2
28.5
25.7
22.6
14.0
200 190 180 170 160 150 140 130 120 110 100 90 80 70 60 50 40 30 20 10 0
f1 (ppm)

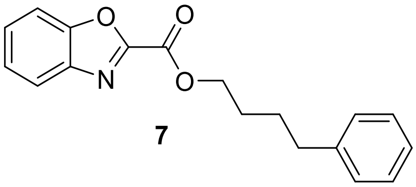
O
N
O
O

7

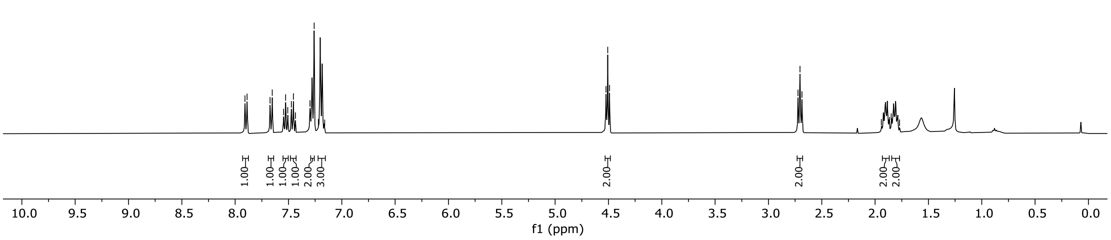
7.91
7.89
7.67
7.65
7.55
7.53
7.51
7.47
7.45
7.44
7.30
7.26
7.22
7.16
4.52
4.51
4.49
2.72
2.70
2.69
1.94
1.86
1.85
1.77
1.00
1.00
1.00
1.00
2.00
3.00
2.00
2.00
2.00
2.00
10.0
9.5
9.0
8.5
8.0
7.5
7.0
6.5
6.0
5.5
5.0
4.5
4.0
3.5
3.0
2.5
2.0
1.5
1.0
0.5
0.0
f1 (ppm)

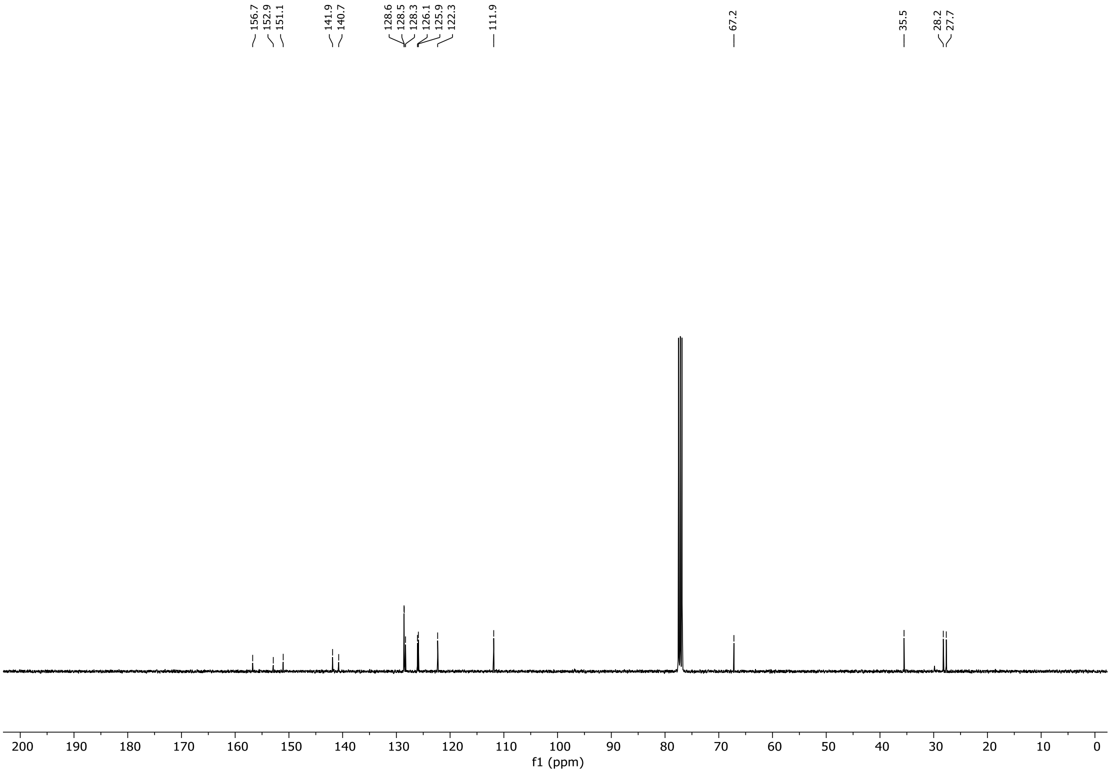
156.7
152.9
151.1
141.9
140.7
128.6
128.5
128.3
126.1
125.9
122.3
111.9
67.2
35.5
28.2
27.7
200
190
180
170
160
150
140
130
120
110
100
90
80
70
60
50
40
30
20
10
0
f1 (ppm)

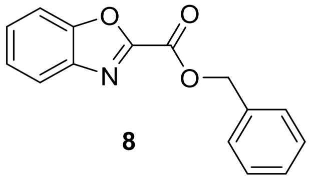

8

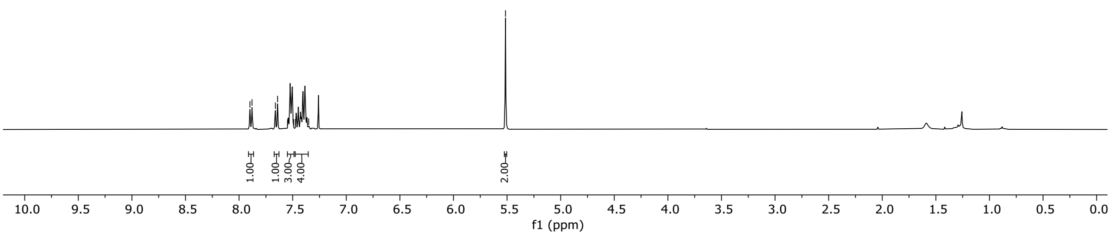
7.90
7.88
7.66
7.64
7.55
7.49
7.47
7.35
5.51
1.00
1.00
3.00
4.00
2.00
10.0 9.5 9.0 8.5 8.0 7.5 7.0 6.5 6.0 5.5 5.0 4.5 4.0 3.5 3.0 2.5 2.0 1.5 1.0 0.5 0.0
f1 (ppm)

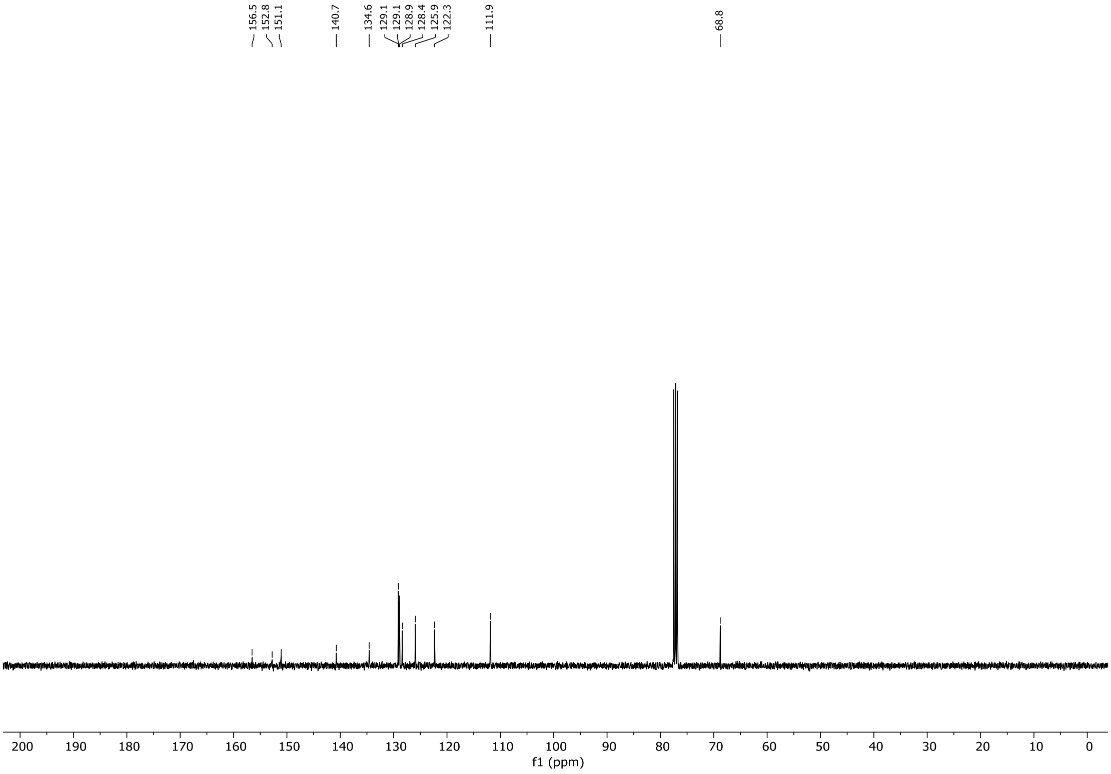
156.5
152.8
151.1
140.7
134.6
129.1
129.1
128.9
128.4
125.9
122.3
111.9
68.8
200 190 180 170 160 150 140 130 120 110 100 90 80 70 60 50 40 30 20 10 0
f1 (ppm)

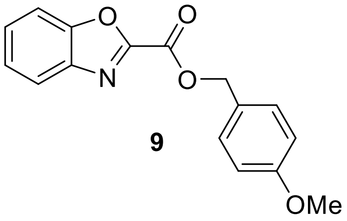
O
O
N
O
OMe

9

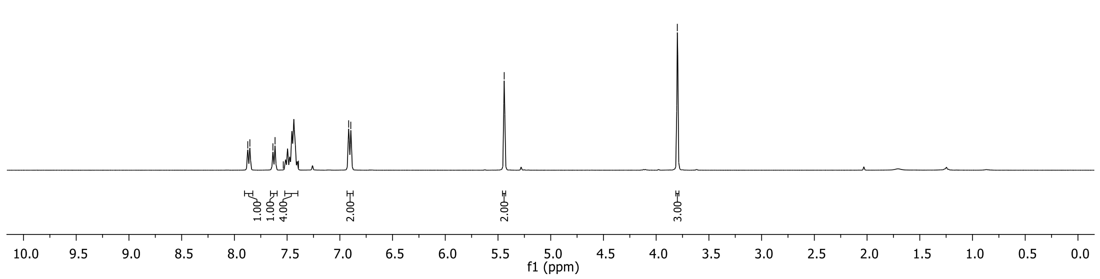
7.87
7.85
7.63
7.61
7.54
7.39
6.92
6.90
5.44
3.80
1.00
1.00
4.00
2.00
2.00
3.00
10.0
9.5
9.0
8.5
8.0
7.5
7.0
6.5
6.0
5.5
5.0
4.5
4.0
3.5
3.0
2.5
2.0
1.5
1.0
0.5
0.0
f1 (ppm)

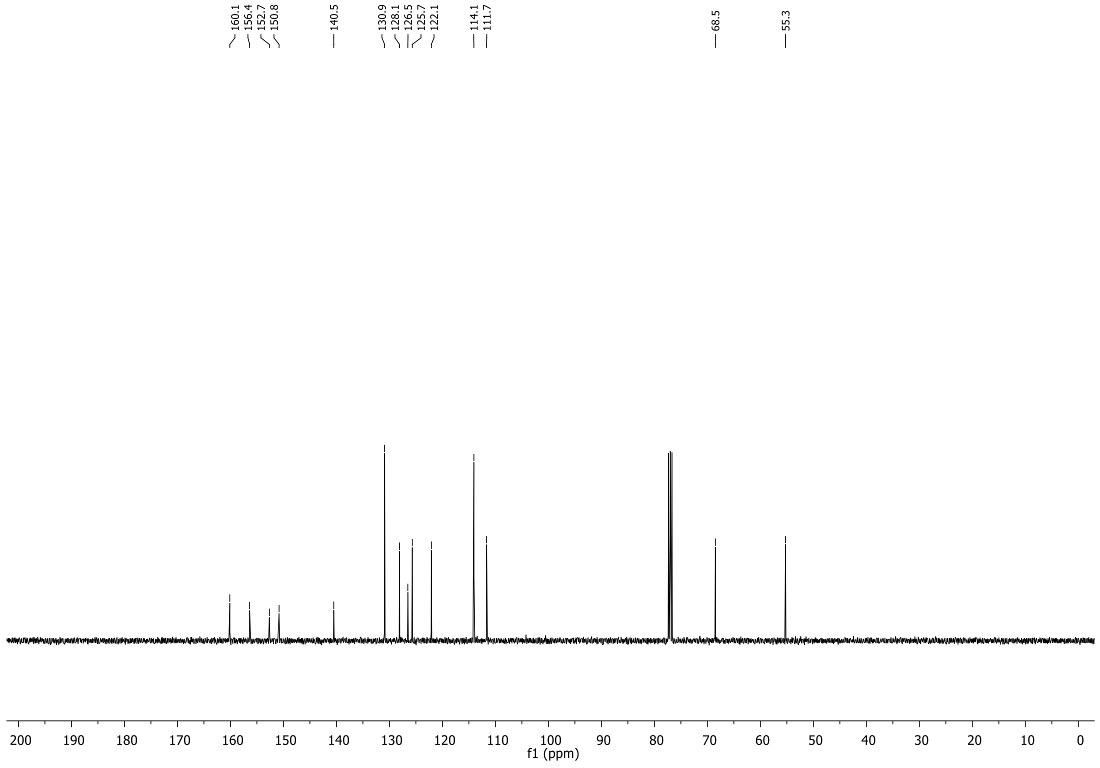
160.1
156.4
152.7
150.8
140.5
130.9
128.1
126.5
125.7
122.1
114.1
111.7
68.5
55.3
200
190
180
170
160
150
140
130
120
110
100
90
80
70
60
50
40
30
20
10
0
f1 (ppm)

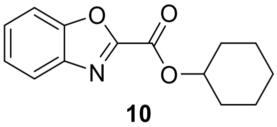

10

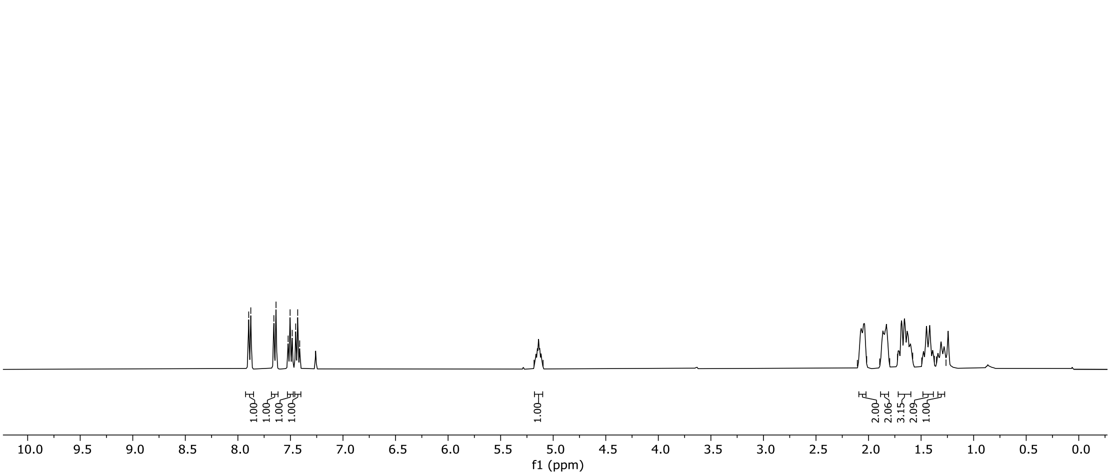
7.90
7.88
7.66
7.64
7.52
7.50
7.48
7.45
7.43
7.41
5.18
5.10
2.11
2.02
1.89
1.80
1.72
1.58
1.49
1.37
1.35
1.26
1.00
1.00
1.00
1.00
1.00
2.00
2.06
3.15
2.09
1.00
10.0
9.5
9.0
8.5
8.0
7.5
7.0
6.5
6.0
5.5
5.0
4.5
4.0
3.5
3.0
2.5
2.0
1.5
1.0
0.5
0.0
f1 (ppm)

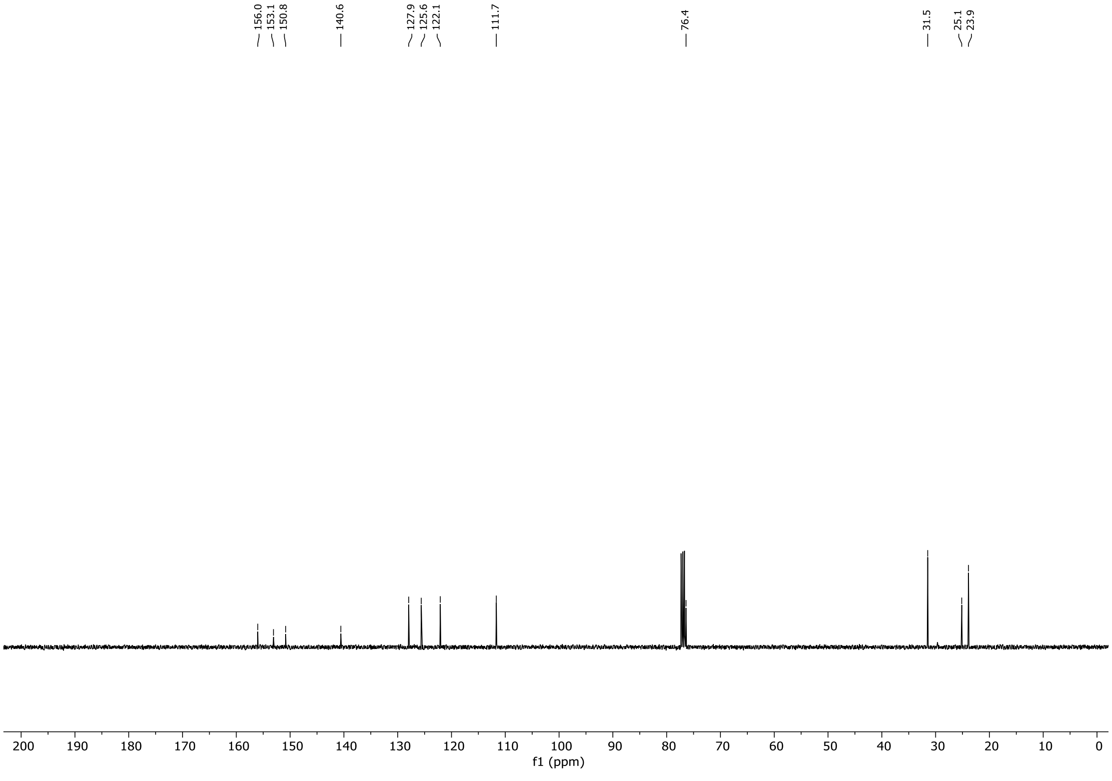
156.0
153.1
150.8
140.6
127.9
125.6
122.1
111.7
76.4
31.5
25.1
23.9
200
190
180
170
160
150
140
130
120
110
100
90
80
70
60
50
40
30
20
10
0
f1 (ppm)

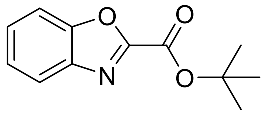
O
O
N
O

11

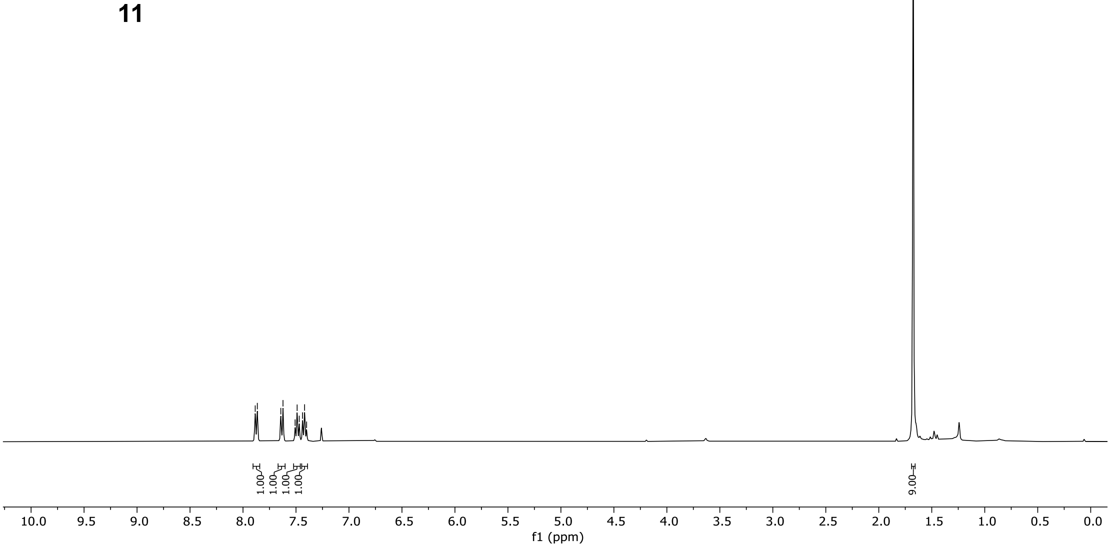
7.88
7.86
7.64
7.62
7.51
7.49
7.47
7.44
7.42
7.40
1.68
1.00
1.00
1.00
1.00
9.00
f1 (ppm)

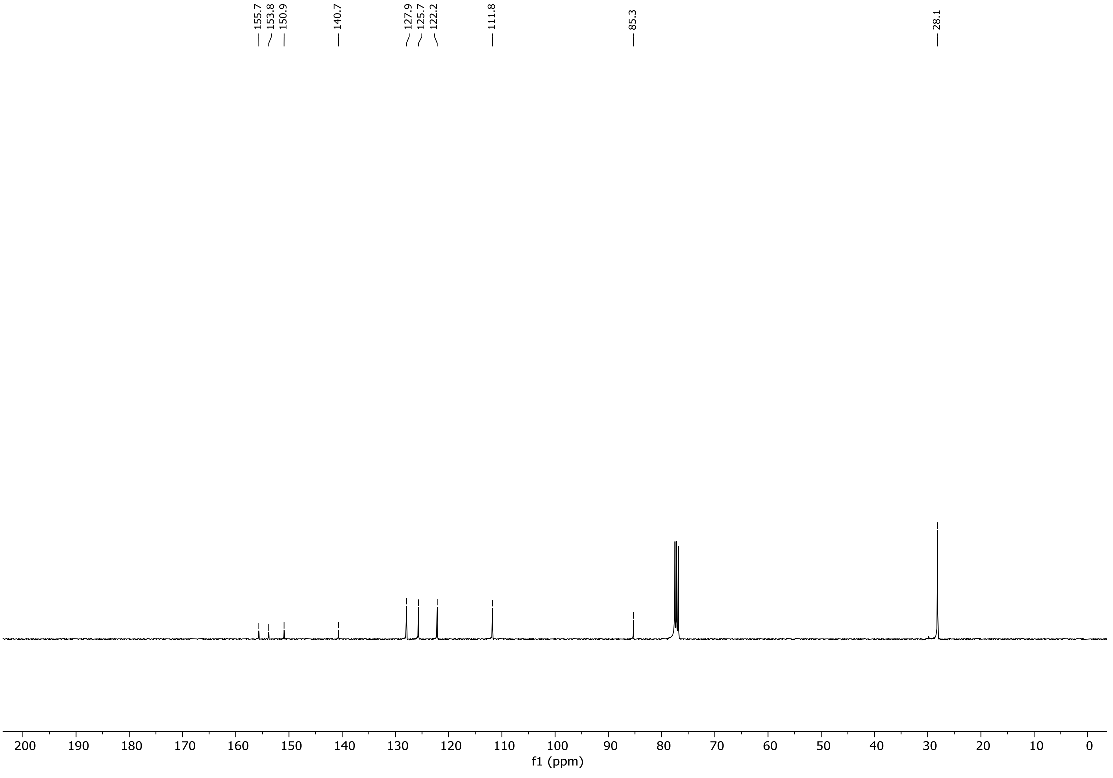
155.7
153.8
150.9
140.7
127.9
125.7
122.2
111.8
85.3
28.1
f1 (ppm)

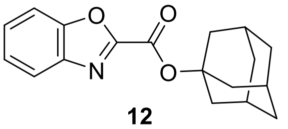
O
O
N
O

12

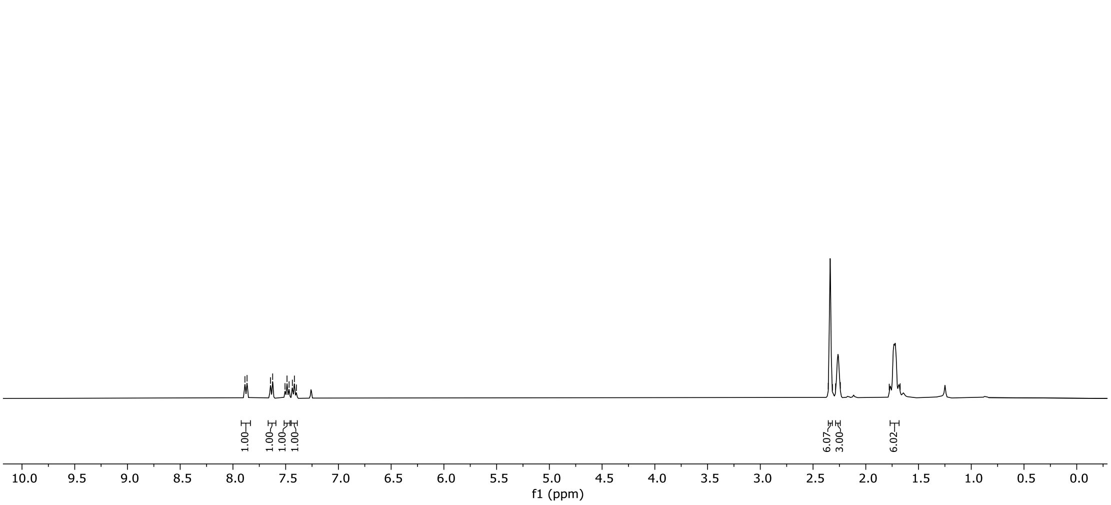
7.89
7.87
7.64
7.62
7.51
7.49
7.47
7.44
7.42
7.40
2.36
2.32
2.29
2.24
1.78
1.67
1.00
1.00
1.00
1.00
6.07
3.00
6.02
10.0 9.5 9.0 8.5 8.0 7.5 7.0 6.5 6.0 5.5 5.0 4.5 4.0 3.5 3.0 2.5 2.0 1.5 1.0 0.5 0.0
f1 (ppm)

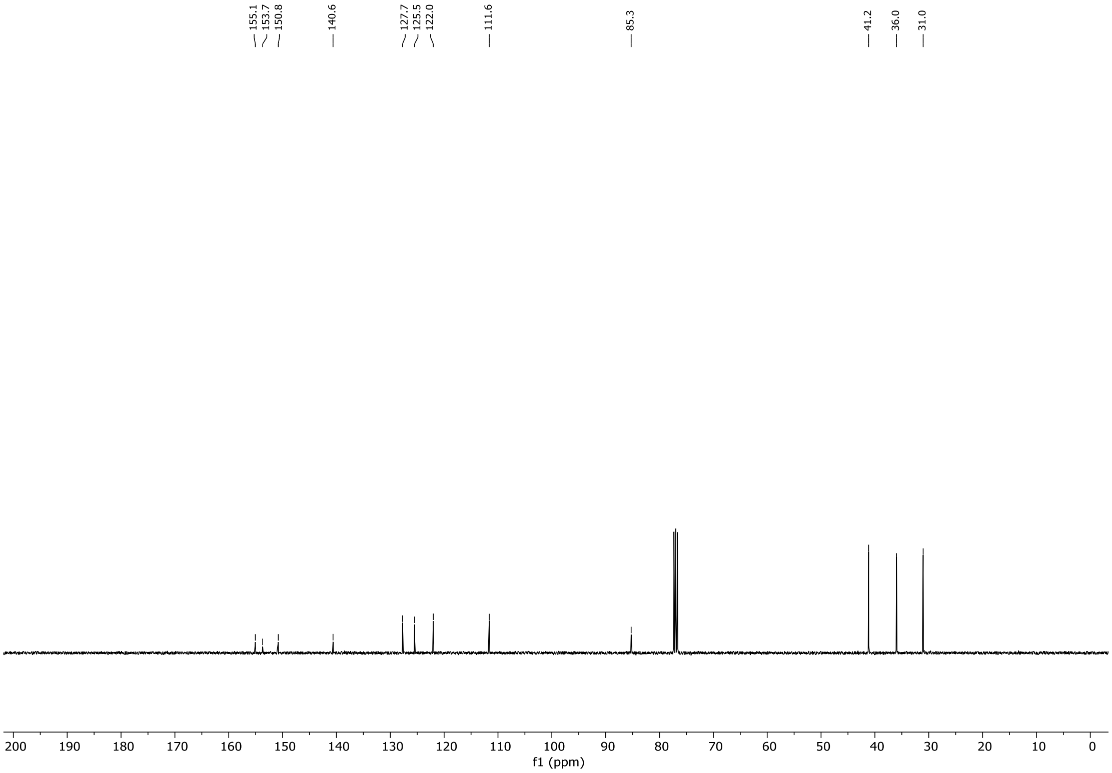
155.1
153.7
150.8
140.6
127.7
125.5
122.0
111.6
85.3
41.2
36.0
31.0
200 190 180 170 160 150 140 130 120 110 100 90 80 70 60 50 40 30 20 10 0
f1 (ppm)

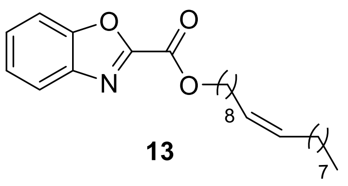

13

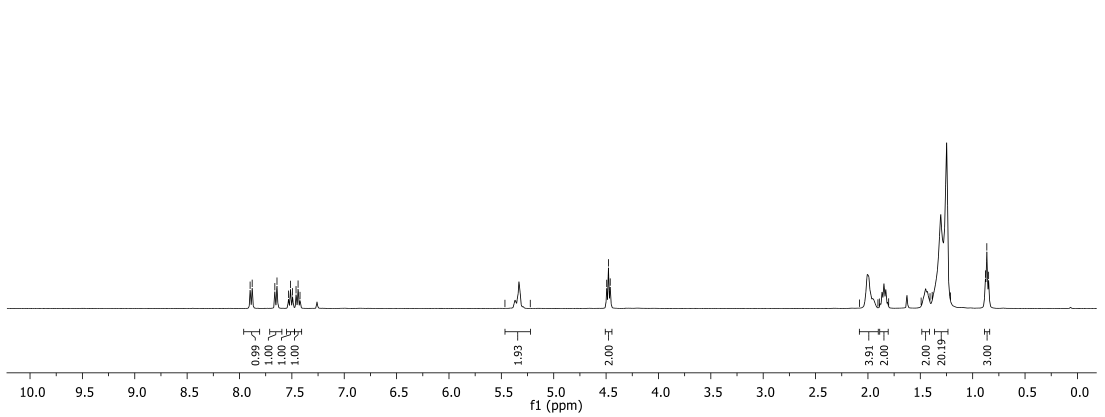

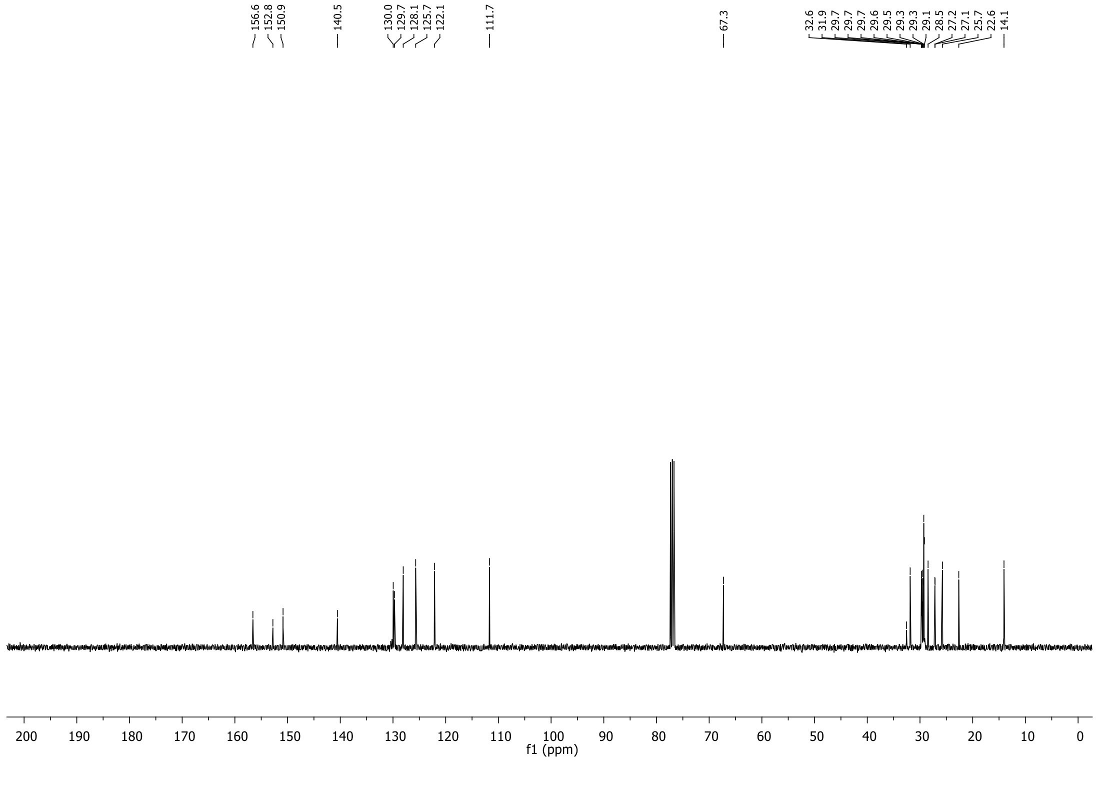

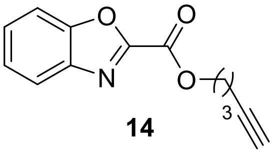

14

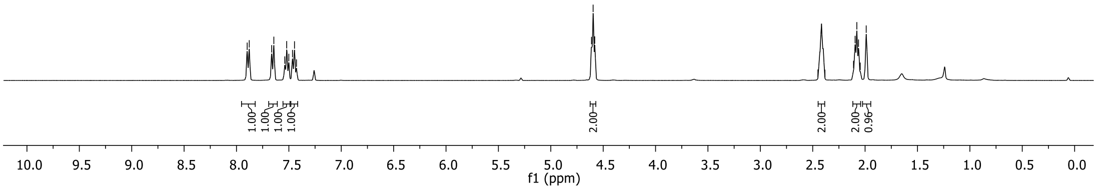

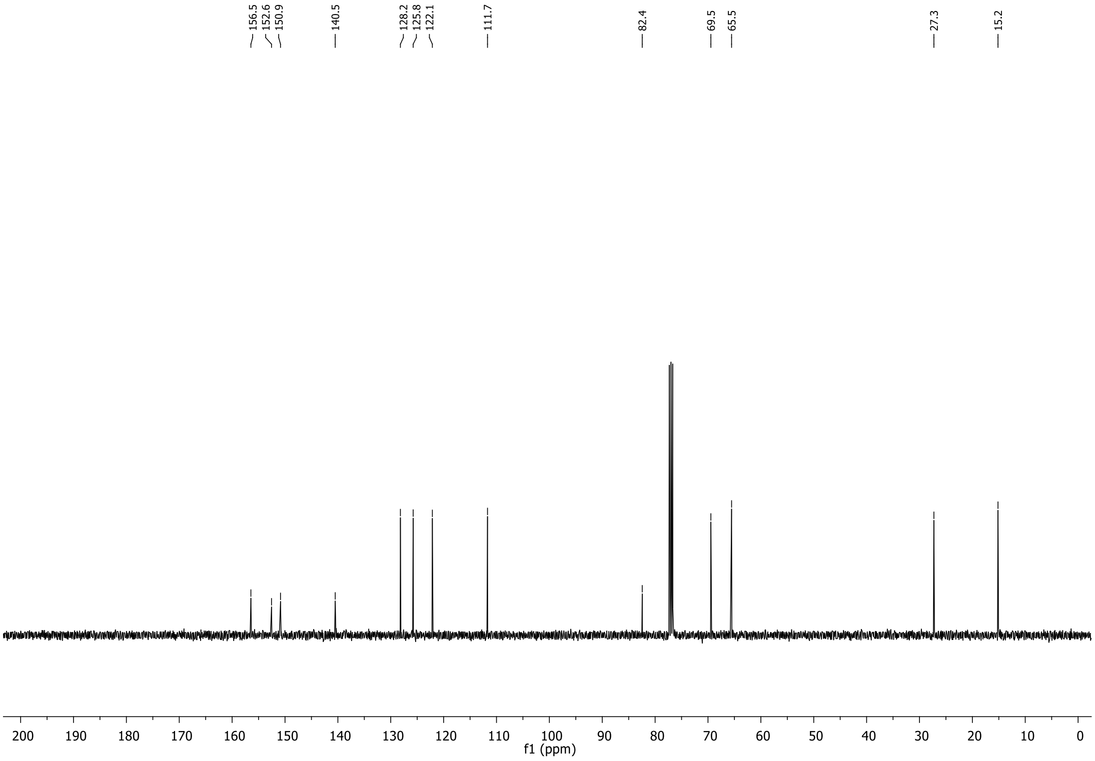

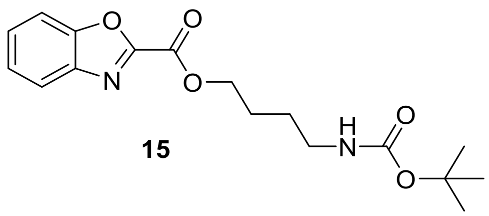
O
O
N
O
H
N
O
O

15

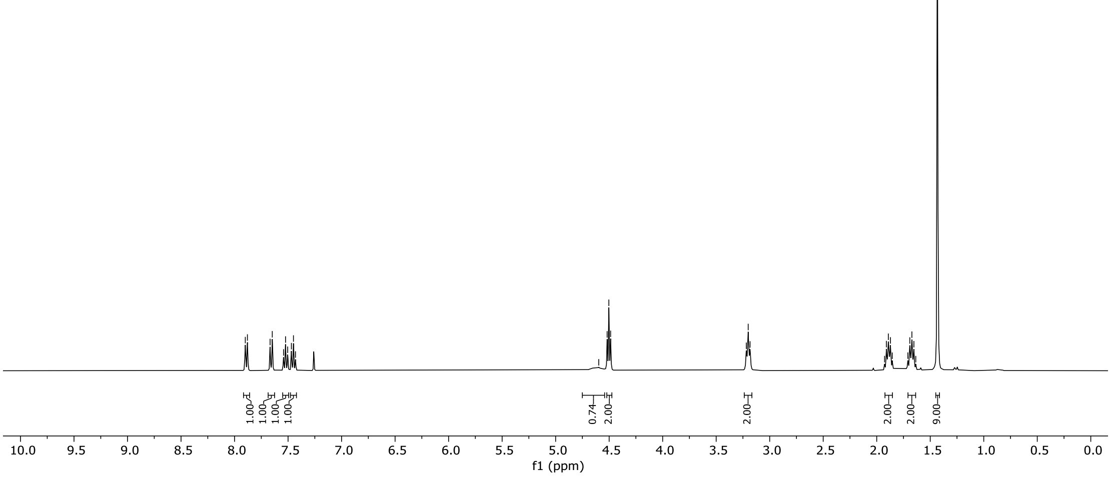
7.90
7.88
7.67
7.65
7.54
7.52
7.50
7.47
7.45
7.43
4.60
4.52
4.50
4.49
3.22
3.20
3.18
1.93
1.91
1.89
1.87
1.85
1.71
1.69
1.67
1.65
1.64
1.43
1.00
1.00
1.00
1.00
0.74
2.00
2.00
2.00
2.00
9.00
f1 (ppm)

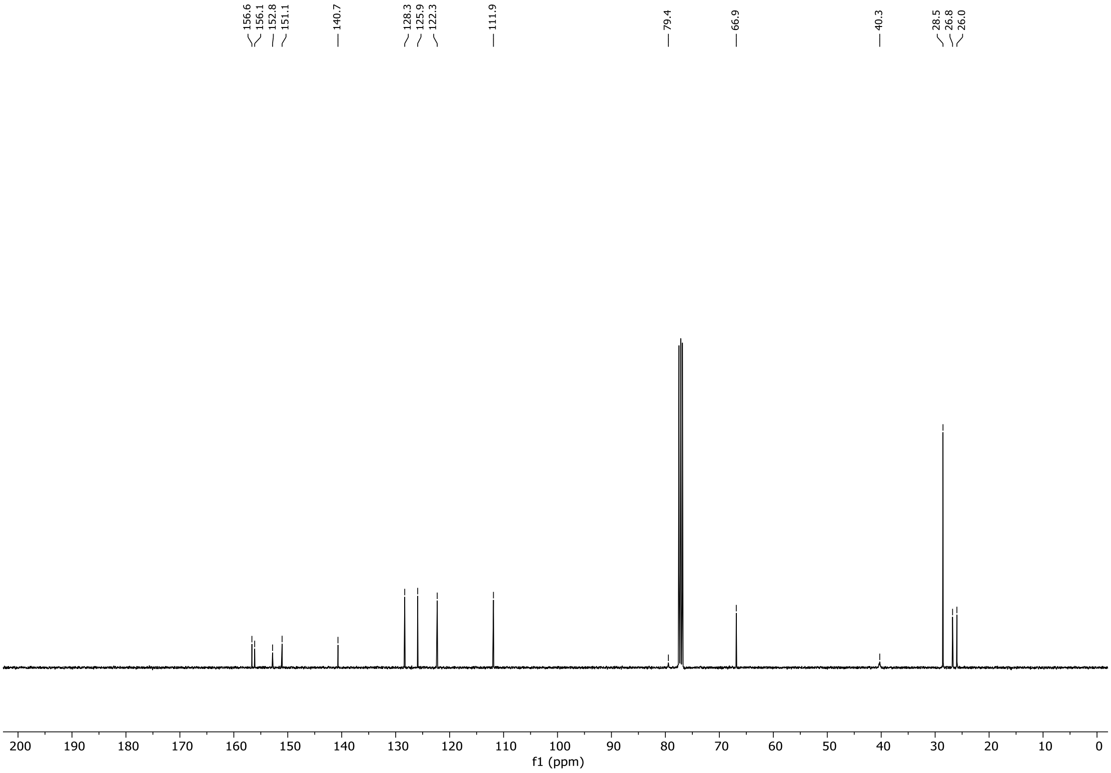
156.6
156.1
152.8
151.1
140.7
128.3
125.9
122.3
111.9
79.4
66.9
40.3
28.5
26.8
26.0
f1 (ppm)

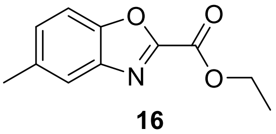
O
O
N
O

16

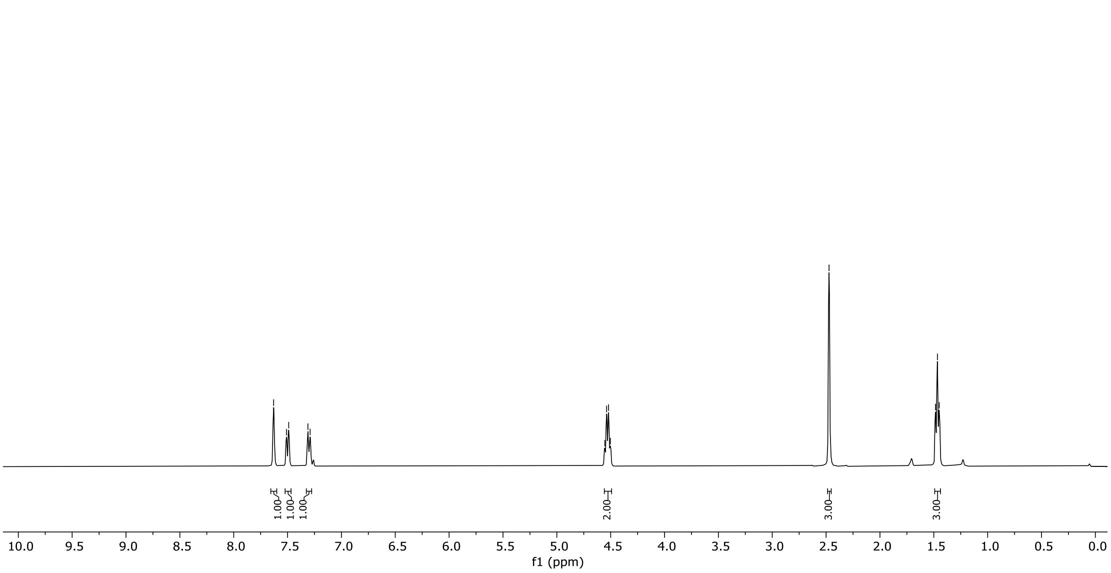
7.63
7.51
7.49
7.31
7.29
4.55
4.54
4.52
4.50
2.47
1.48
1.47
1.45
1.00
1.00
1.00
2.00
3.00
3.00
f1 (ppm)

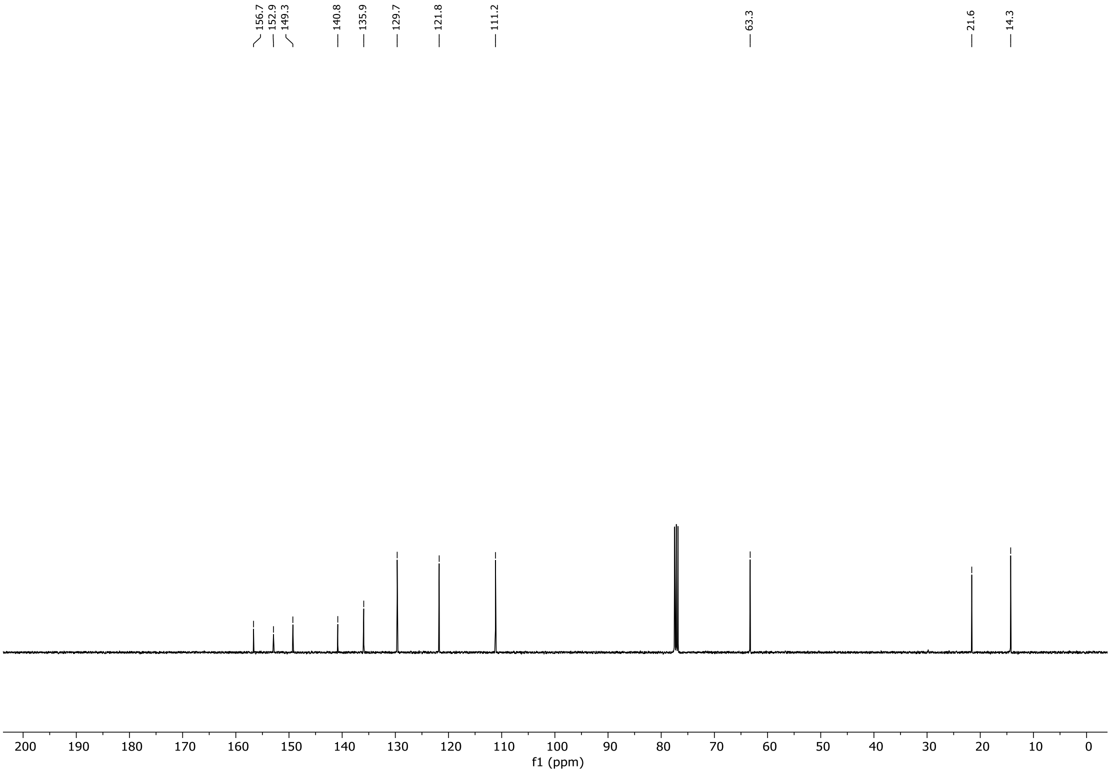
156.7
152.9
149.3
140.8
135.9
129.7
121.8
111.2
63.3
21.6
14.3
f1 (ppm)

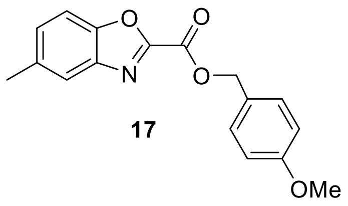

17

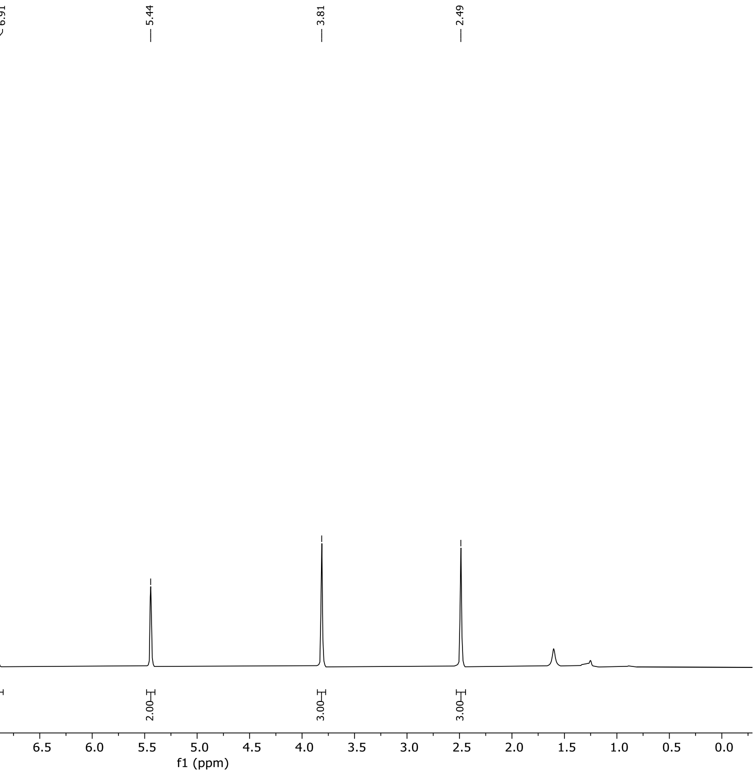

7.63
7.51
7.49
7.47
7.45
7.33
7.30
6.92
6.91
5.44
3.81
2.49
1.00
1.00
2.00
1.00
2.00
2.00
3.00
3.00
10.0 9.5 9.0 8.5 8.0 7.5 7.0 6.5 6.0 5.5 5.0 4.5 4.0 3.5 3.0 2.5 2.0 1.5 1.0 0.5 0.0
f1 (ppm)

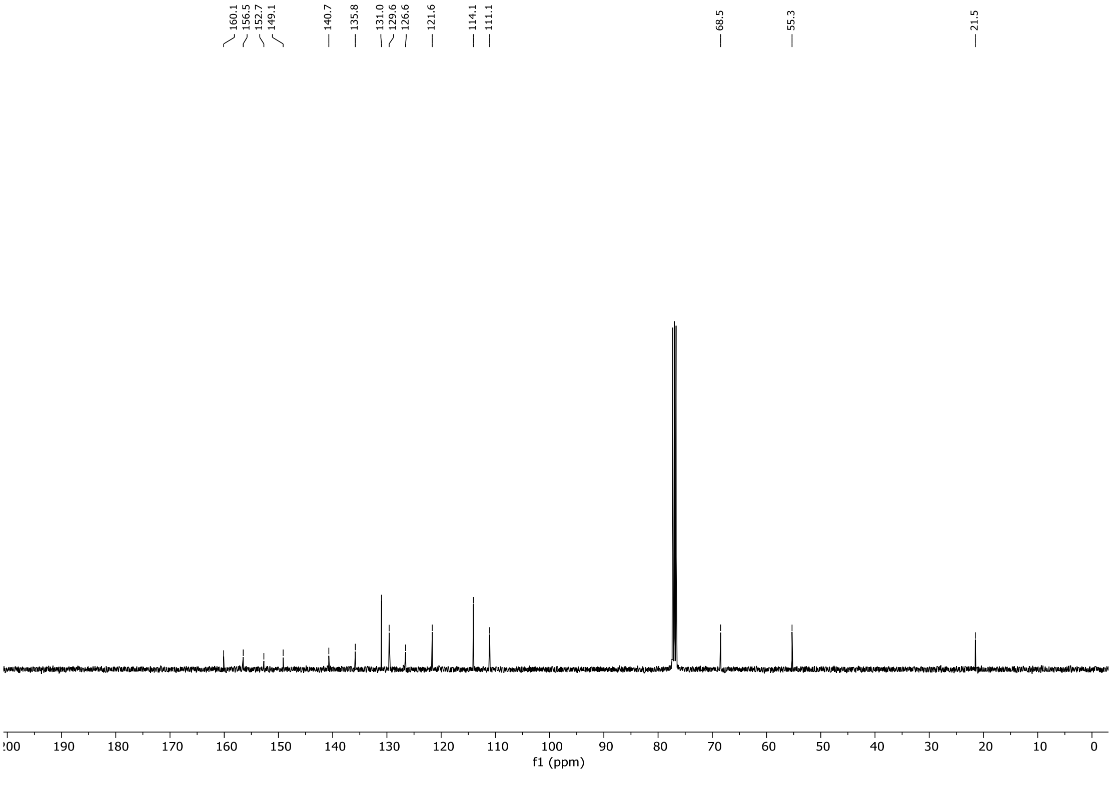

160.1
156.5
152.7
149.1
140.7
135.8
131.0
129.6
126.6
121.6
114.1
111.1
68.5
55.3
21.5
200 190 180 170 160 150 140 130 120 110 100 90 80 70 60 50 40 30 20 10 0
f1 (ppm)

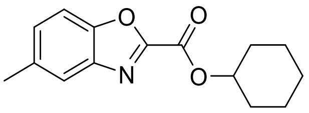
O
O
N
O

18

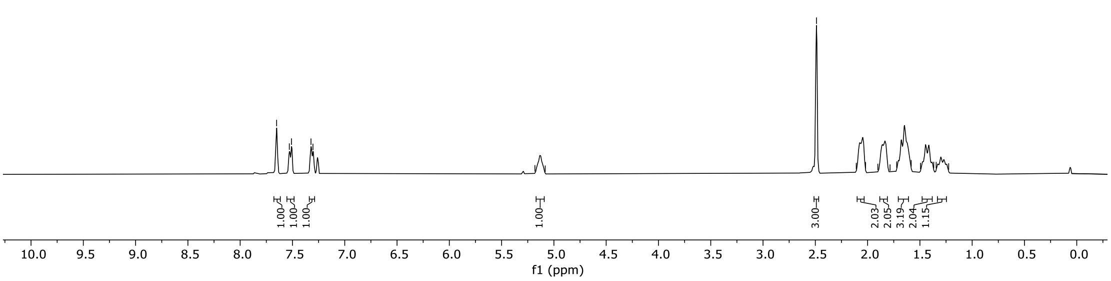
7.65
7.53
7.51
7.32
7.30
5.18
5.08
2.49
2.11
2.02
1.90
1.79
1.72
1.58
1.49
1.37
1.34
1.22
1.00
1.00
1.00
1.00
3.00
2.03
2.05
3.19
2.04
1.15
f1 (ppm)

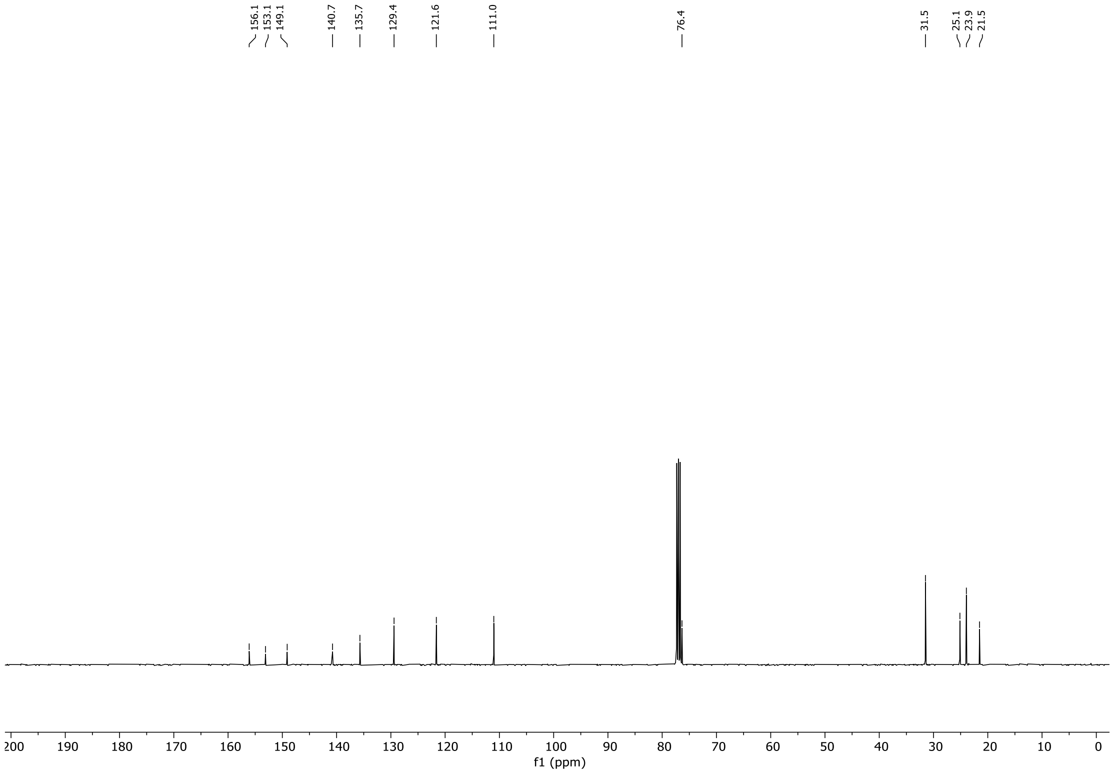
156.1
153.1
149.1
140.7
135.7
129.4
121.6
111.0
76.4
31.5
25.1
23.9
21.5
f1 (ppm)

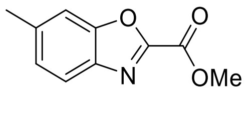
O
N
O
OMe

19

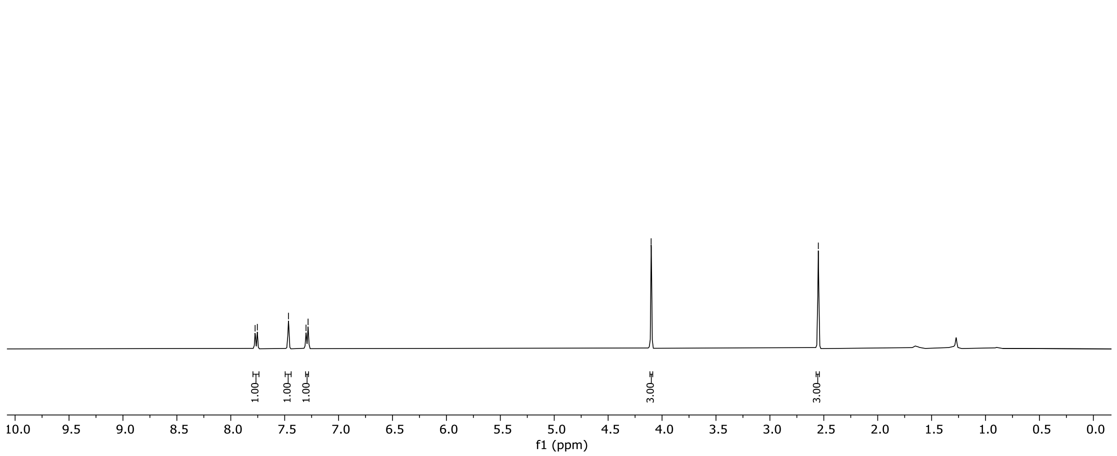
7.77
7.75
7.46
7.30
7.28
4.10
2.55
1.00
1.00
1.00
3.00
3.00
10.0 9.5 9.0 8.5 8.0 7.5 7.0 6.5 6.0 5.5 5.0 4.5 4.0 3.5 3.0 2.5 2.0 1.5 1.0 0.5 0.0
f1 (ppm)

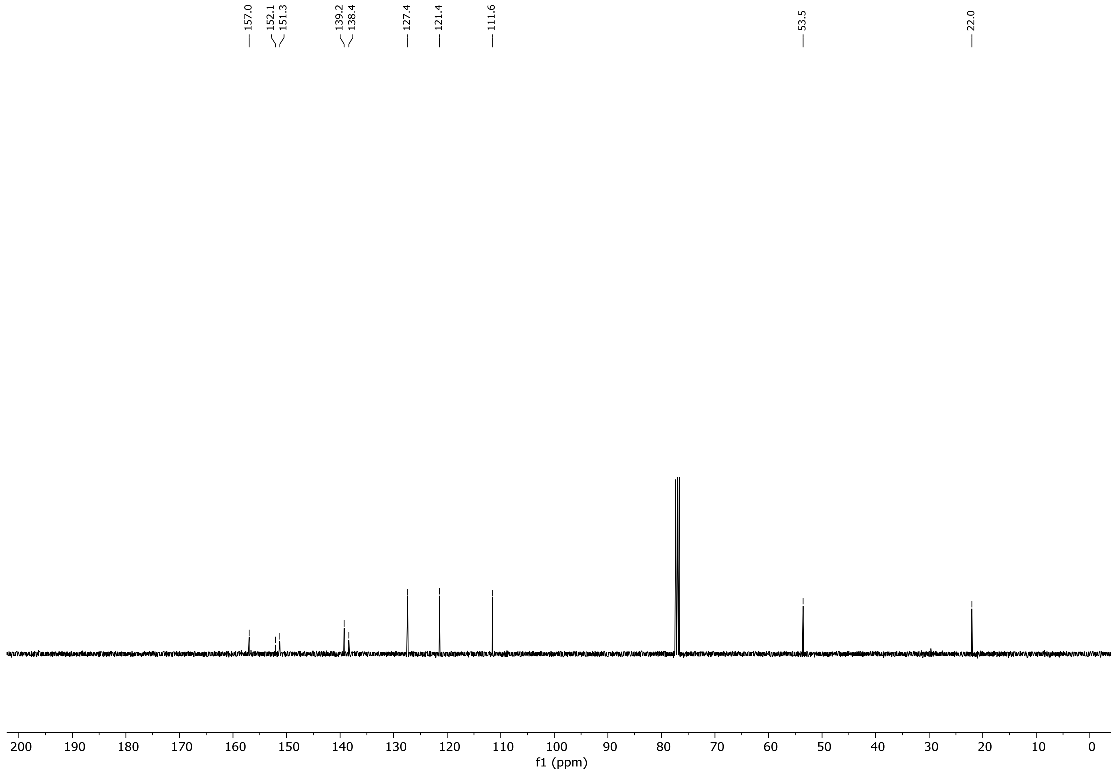
157.0
152.1
151.3
139.2
138.4
127.4
121.4
111.6
53.5
22.0
200 190 180 170 160 150 140 130 120 110 100 90 80 70 60 50 40 30 20 10 0
f1 (ppm)

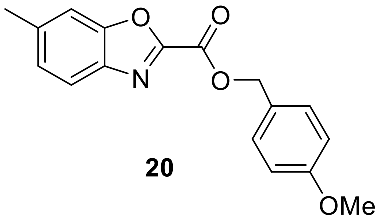
O
O
N
O
OMe

20

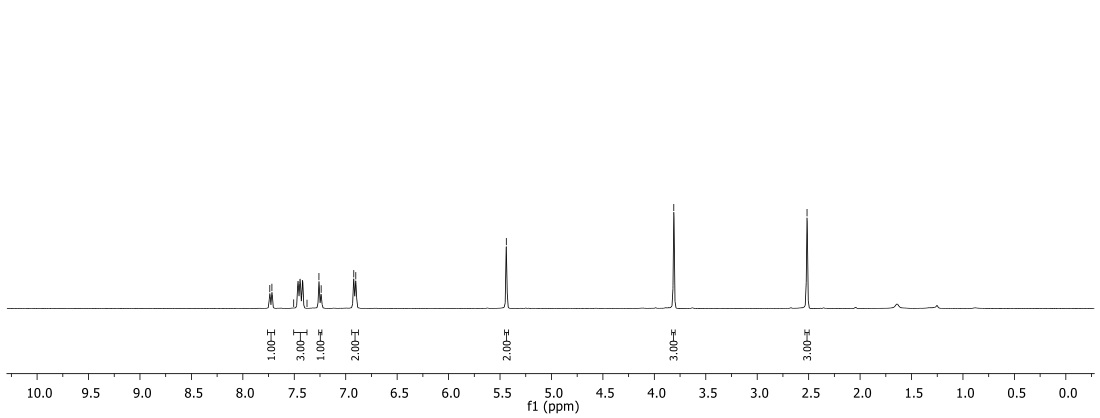
7.74
7.72
7.51
7.38
7.26
7.24
6.92
6.90
5.44
3.81
2.52
1.00
3.00
1.00
2.00
2.00
3.00
3.00
10.0 9.5 9.0 8.5 8.0 7.5 7.0 6.5 6.0 5.5 5.0 4.5 4.0 3.5 3.0 2.5 2.0 1.5 1.0 0.5 0.0
f1 (ppm)

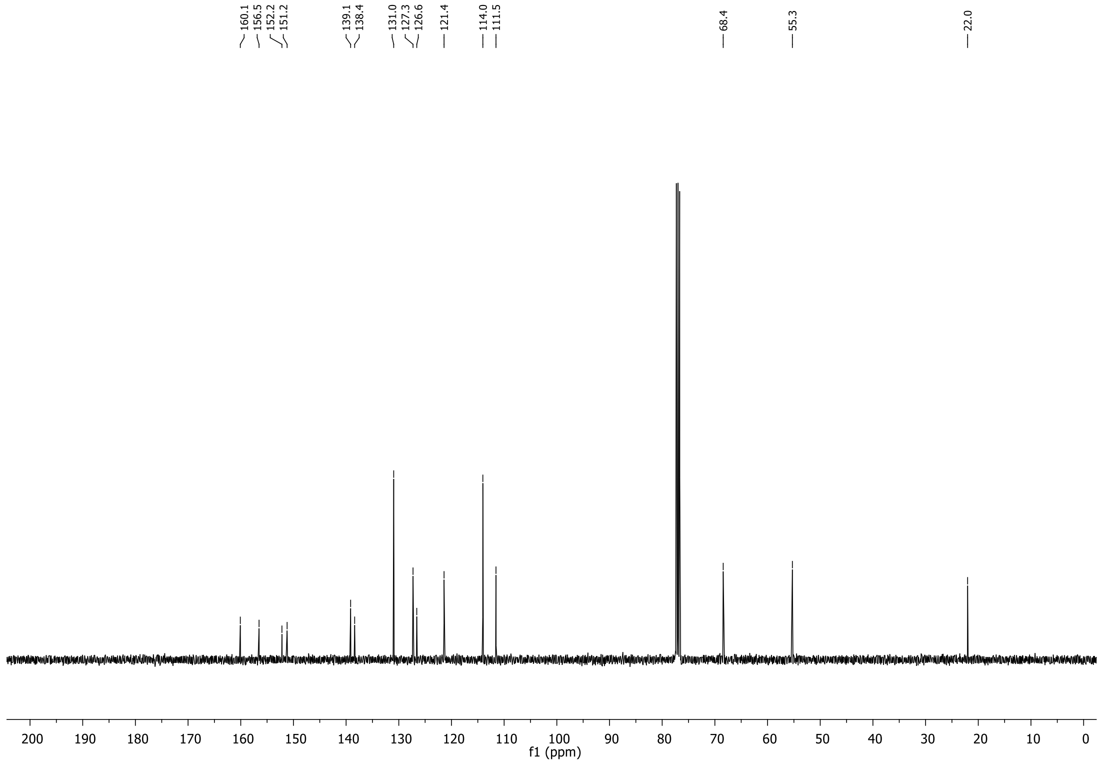
160.1
156.5
152.2
151.2
139.1
138.4
131.0
127.3
126.6
121.4
114.0
111.5
68.4
55.3
22.0
200 190 180 170 160 150 140 130 120 110 100 90 80 70 60 50 40 30 20 10 0
f1 (ppm)

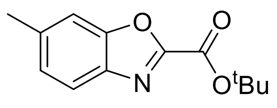
O
N
O
O^{t}Bu

21

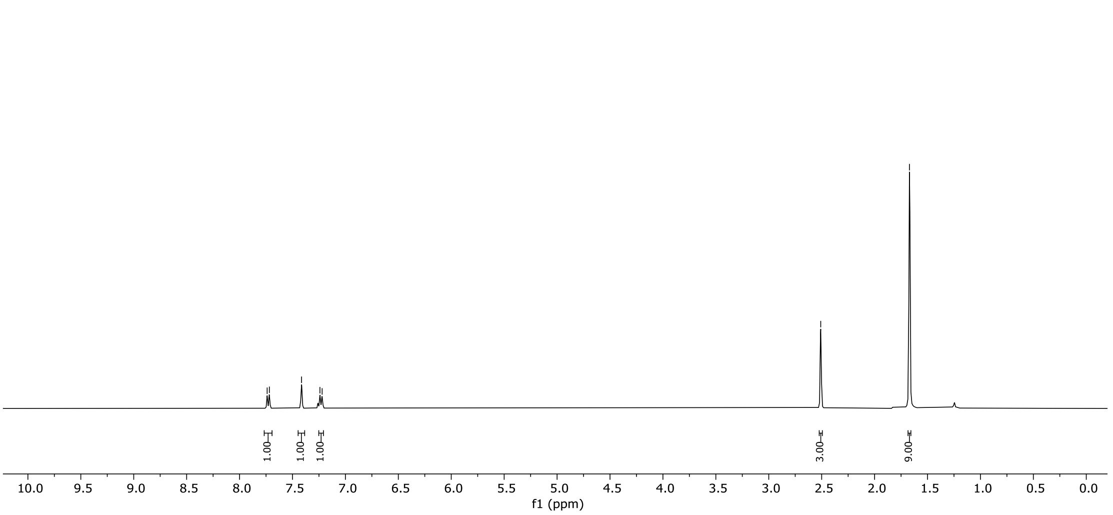
7.74
7.72
7.41
7.24
7.22
2.51
1.67
1.00
1.00
1.00
3.00
9.00
f1 (ppm)

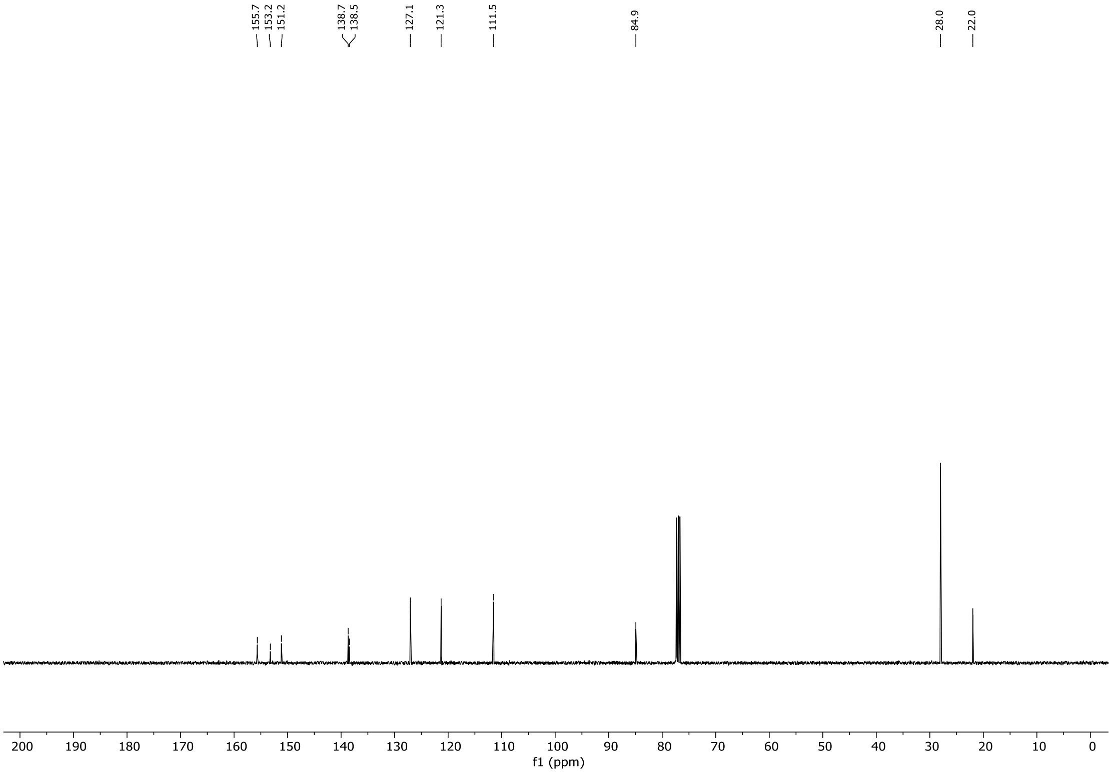
155.7
153.2
151.2
138.7
138.5
127.1
121.3
111.5
84.9
28.0
22.0
f1 (ppm)

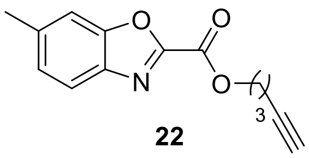
O
N
O
O
3

22

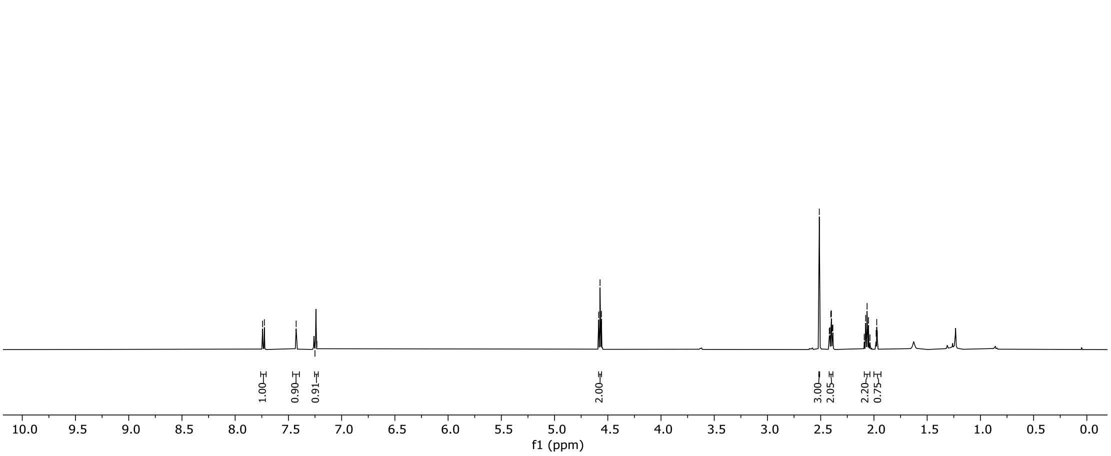
7.74
7.73
7.43
7.25
7.23
4.59
4.57
4.56
2.51
2.42
2.41
2.41
2.40
2.39
2.39
2.09
2.08
2.06
2.05
2.04
1.98
1.97
1.97
1.00
0.90
0.91
2.00
3.00
2.05
2.20
0.75
f1 (ppm)

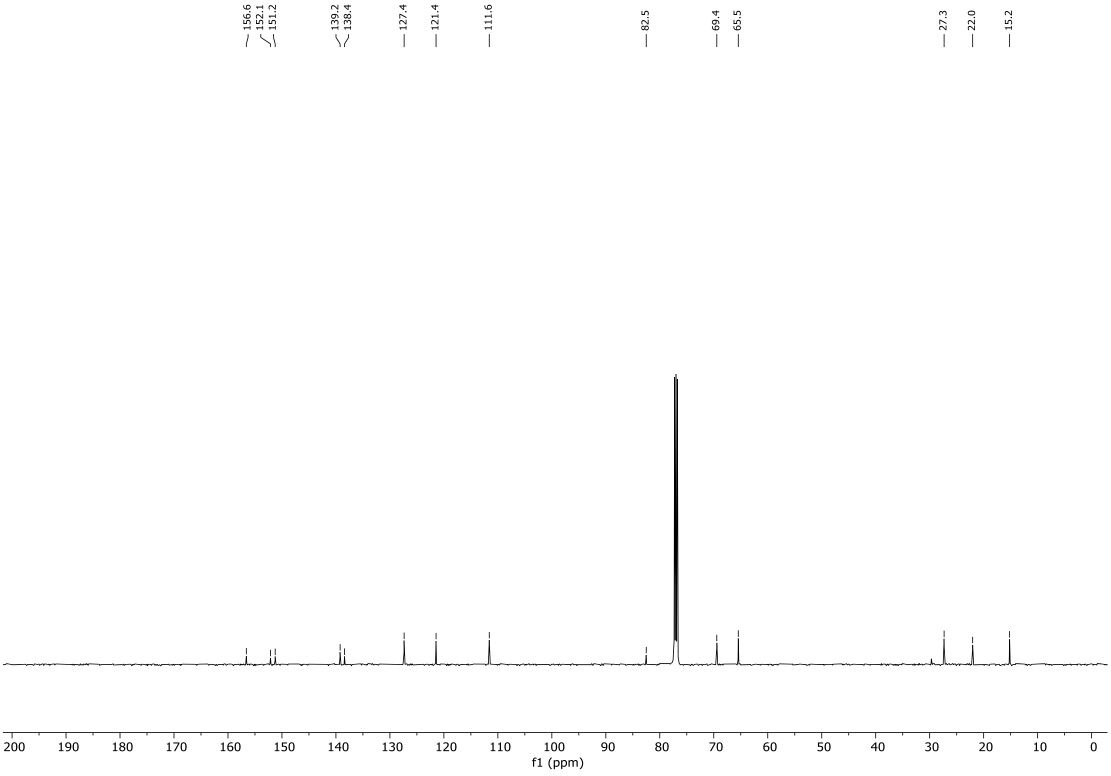
156.6
152.1
151.2
139.2
138.4
127.4
121.4
111.6
82.5
69.4
65.5
27.3
22.0
15.2
f1 (ppm)

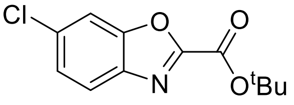

23

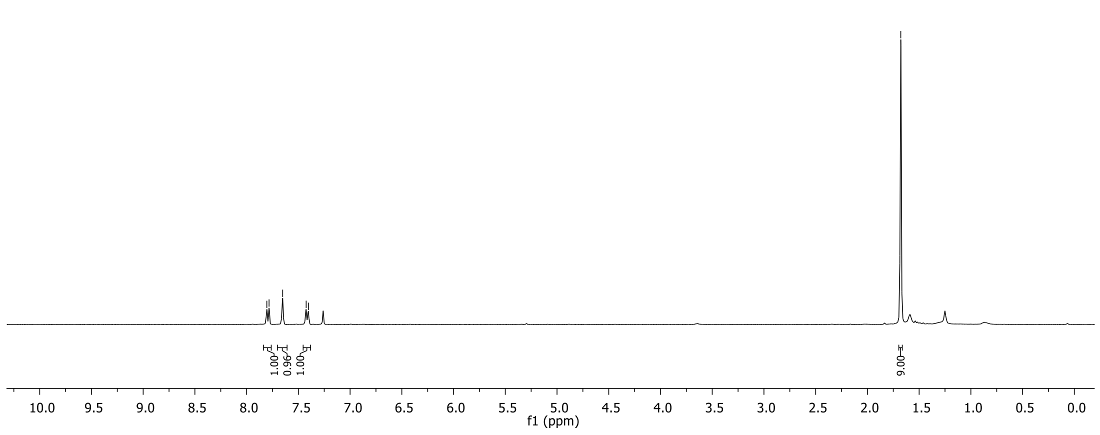
7.80
7.78
7.65
7.42
7.40
1.68
1.00
0.96
1.00
9.00
10.0 9.5 9.0 8.5 8.0 7.5 7.0 6.5 6.0 5.5 5.0 4.5 4.0 3.5 3.0 2.5 2.0 1.5 1.0 0.5 0.0
f1 (ppm)

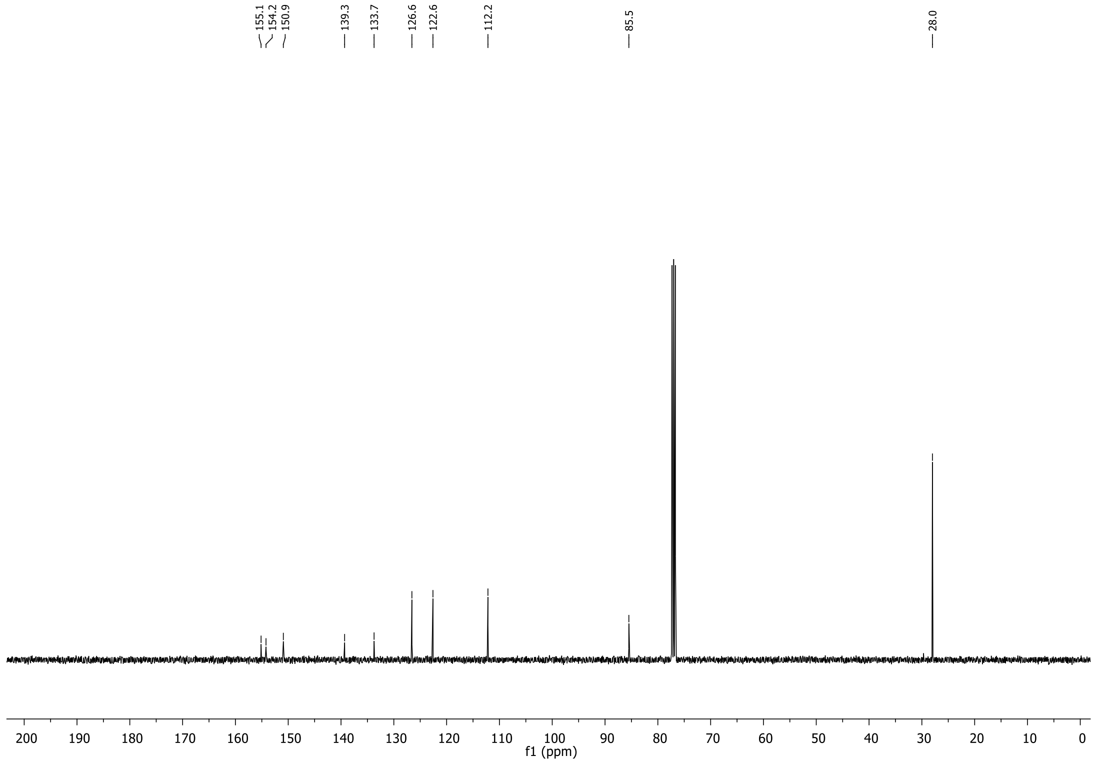
155.1
154.2
150.9
139.3
133.7
126.6
122.6
112.2
85.5
28.0
200 190 180 170 160 150 140 130 120 110 100 90 80 70 60 50 40 30 20 10 0
f1 (ppm)

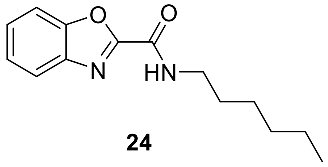
O
O
N
HN

24

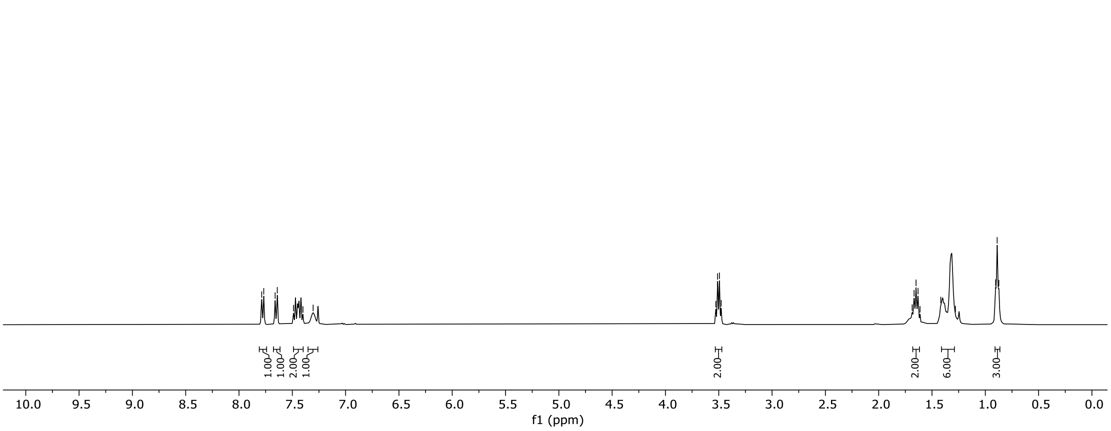
7.79
7.77
7.66
7.64
7.49
7.40
7.30
3.53
3.51
3.49
3.48
1.69
1.67
1.65
1.63
1.61
1.42
1.28
0.90
0.89
0.87
1.00
1.00
2.00
1.00
2.00
2.00
6.00
3.00
10.0 9.5 9.0 8.5 8.0 7.5 7.0 6.5 6.0 5.5 5.0 4.5 4.0 3.5 3.0 2.5 2.0 1.5 1.0 0.5 0.0
f1 (ppm)

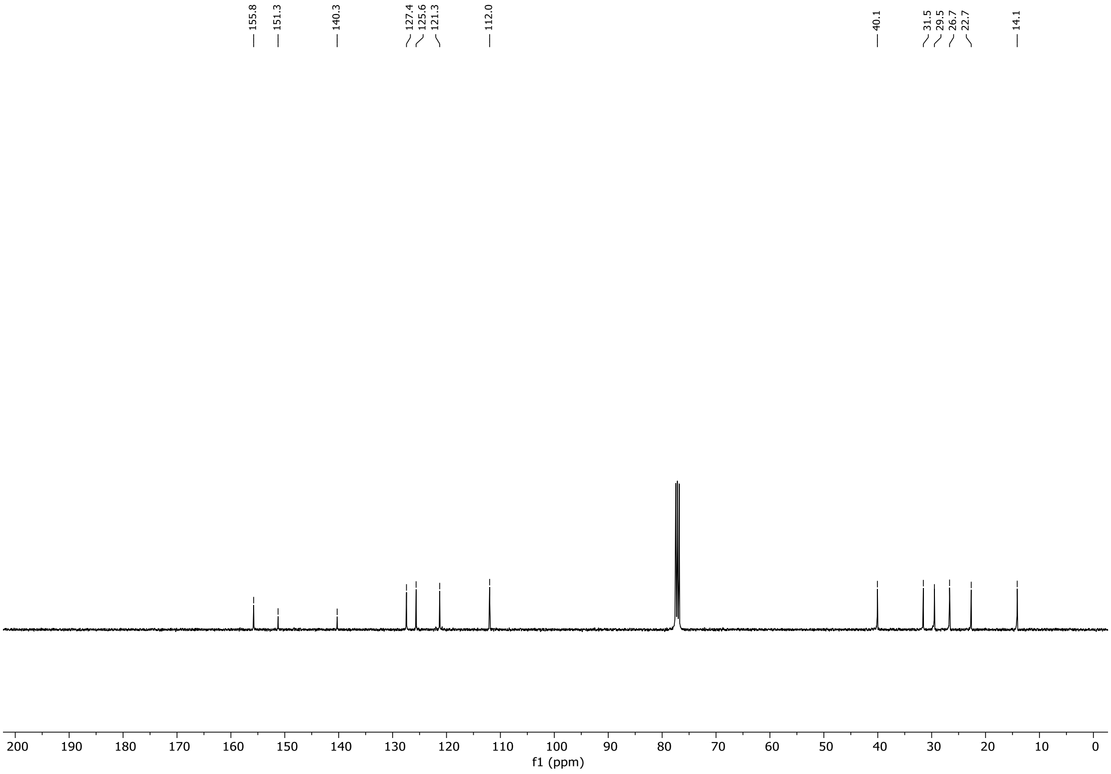
155.8
151.3
140.3
127.4
125.6
121.3
112.0
40.1
31.5
29.5
26.7
22.7
14.1
200 190 180 170 160 150 140 130 120 110 100 90 80 70 60 50 40 30 20 10 0
f1 (ppm)

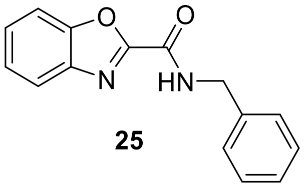
O
O
N
HN

25

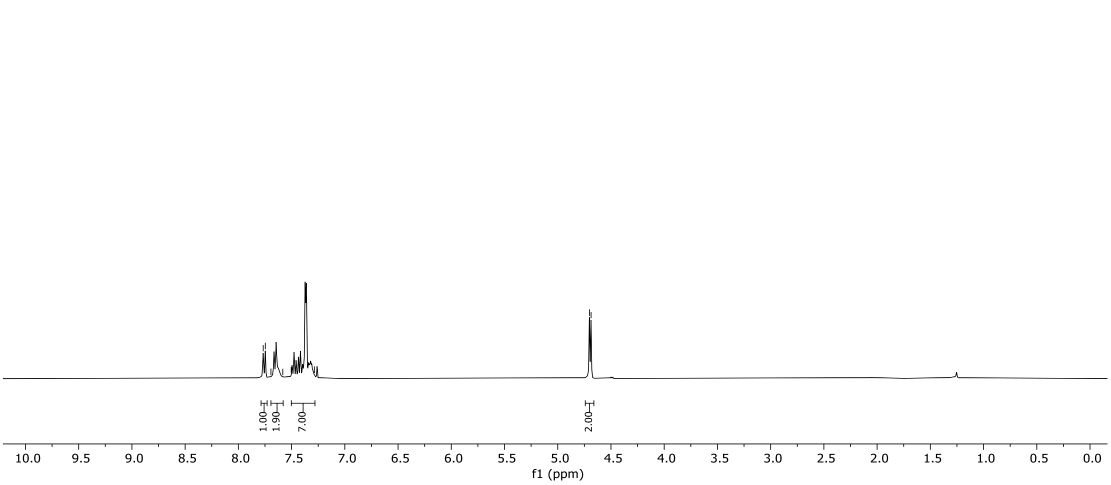
7.77
7.75
7.69
7.58
7.50
7.29
4.70
4.69
1.00
1.90
7.00
2.00
10.0 9.5 9.0 8.5 8.0 7.5 7.0 6.5 6.0 5.5 5.0 4.5 4.0 3.5 3.0 2.5 2.0 1.5 1.0 0.5 0.0
f1 (ppm)

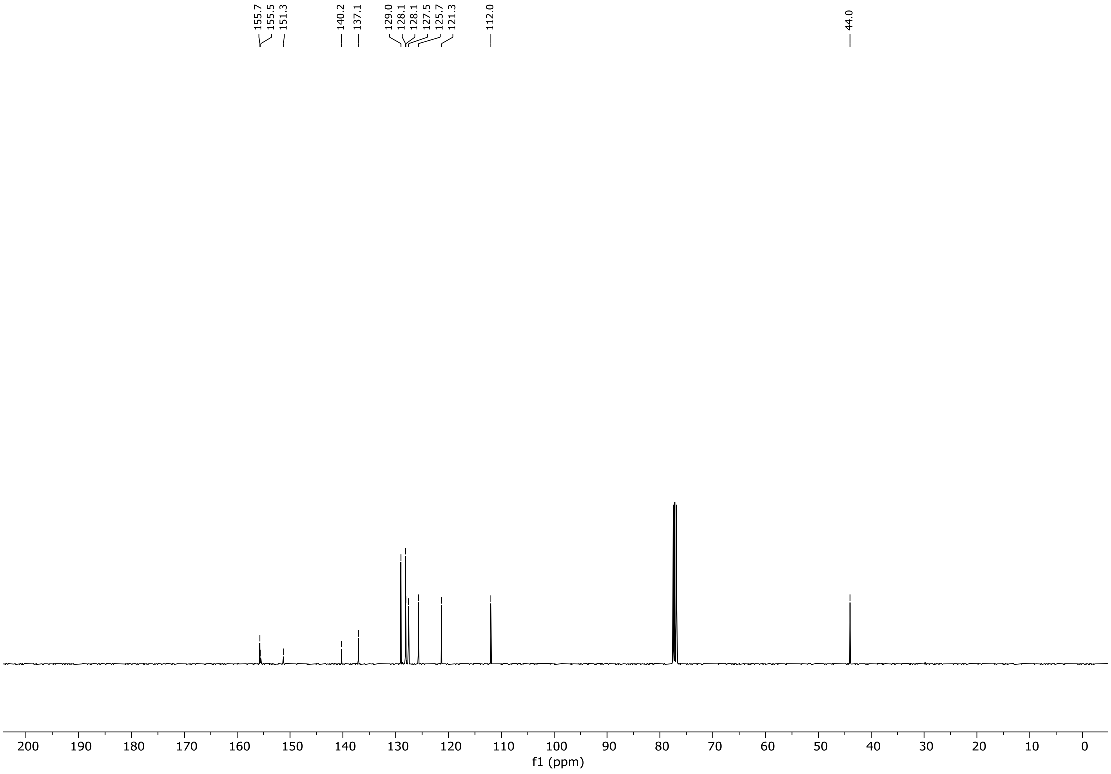
155.7
155.5
151.3
140.2
137.1
129.0
128.1
128.1
127.5
125.7
121.3
112.0
44.0
200 190 180 170 160 150 140 130 120 110 100 90 80 70 60 50 40 30 20 10 0
f1 (ppm)

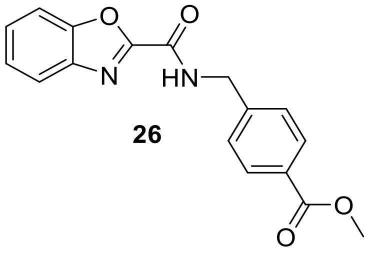

26

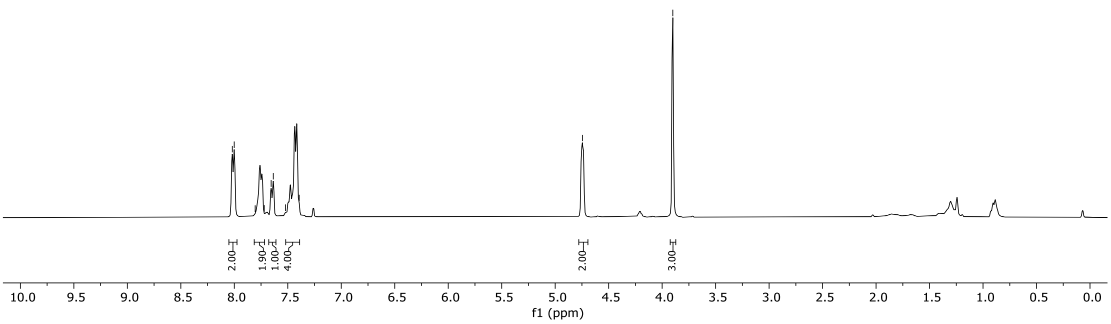
8.02
8.00
7.80
7.72
7.66
7.64
7.52
7.39
4.75
3.90
2.00
1.90
1.00
4.00
2.00
3.00
10.0 9.5 9.0 8.5 8.0 7.5 7.0 6.5 6.0 5.5 5.0 4.5 4.0 3.5 3.0 2.5 2.0 1.5 1.0 0.5 0.0
f1 (ppm)

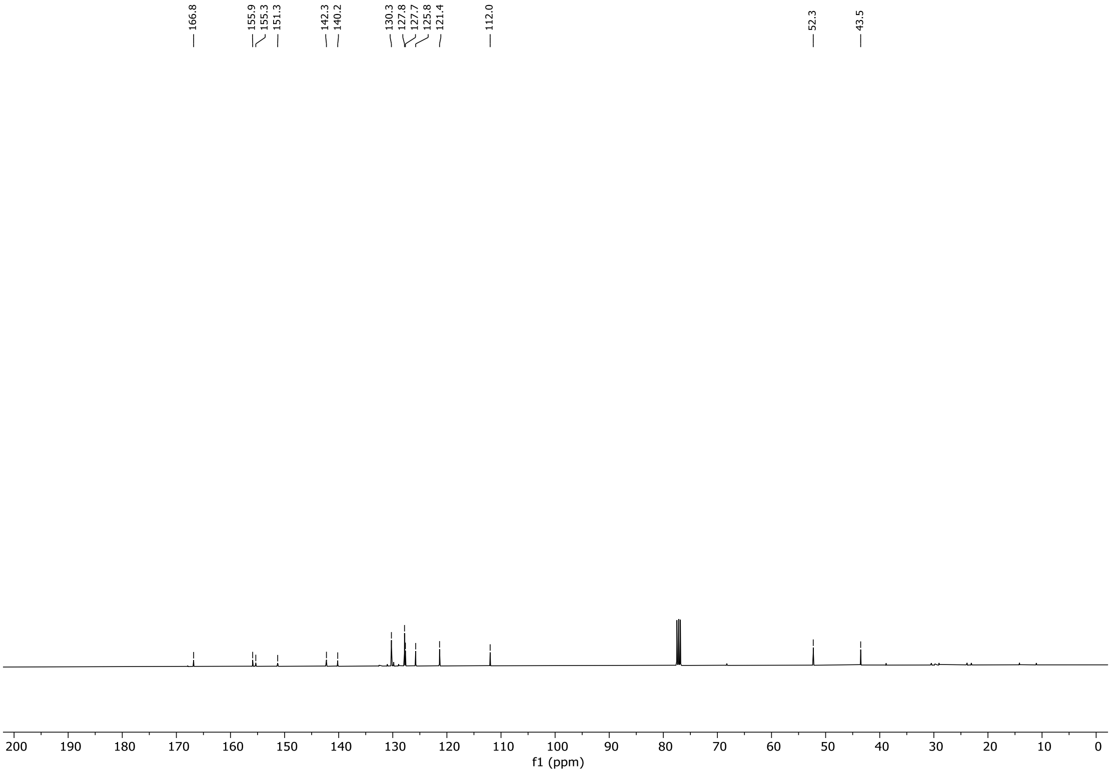
166.8
155.9
155.3
151.3
142.3
140.2
130.3
127.8
127.7
125.8
121.4
112.0
52.3
43.5
200 190 180 170 160 150 140 130 120 110 100 90 80 70 60 50 40 30 20 10 0
f1 (ppm)

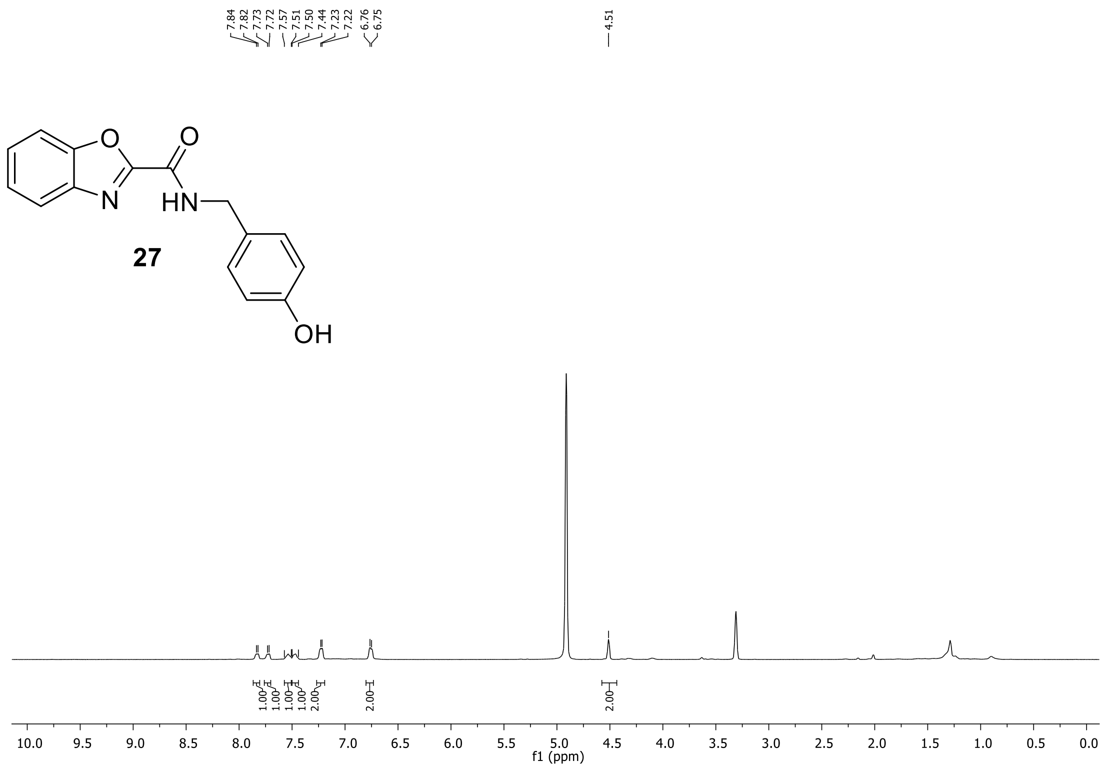

7.84
7.82
7.73
7.72
7.57
7.51
7.50
7.44
7.23
7.22
6.76
6.75
4.51
O
O
N
HN
OH
27
1.00
1.00
1.00
1.00
2.00
2.00
2.00
10.0 9.5 9.0 8.5 8.0 7.5 7.0 6.5 6.0 5.5 5.0 4.5 4.0 3.5 3.0 2.5 2.0 1.5 1.0 0.5 0.0
f1 (ppm)

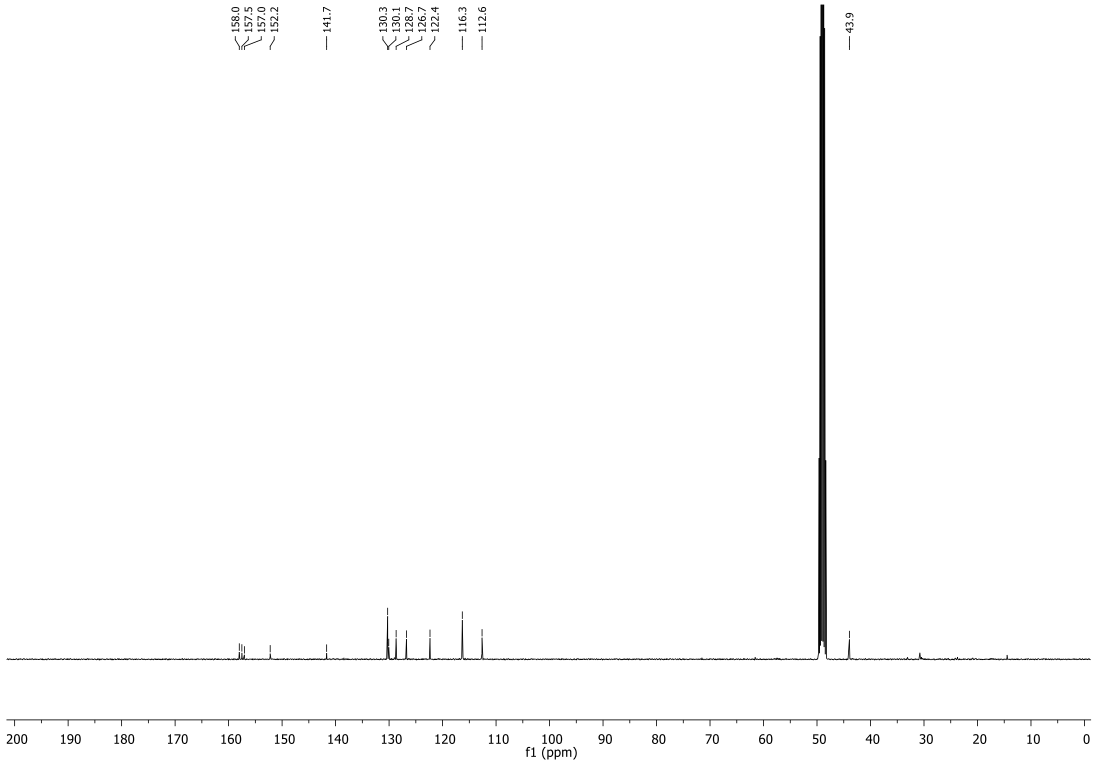

158.0
157.5
157.0
152.2
141.7
130.3
130.1
128.7
126.7
122.4
116.3
112.6
43.9
200 190 180 170 160 150 140 130 120 110 100 90 80 70 60 50 40 30 20 10 0
f1 (ppm)

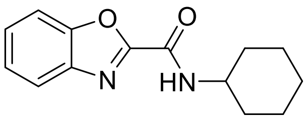
O
O
N
HN

28

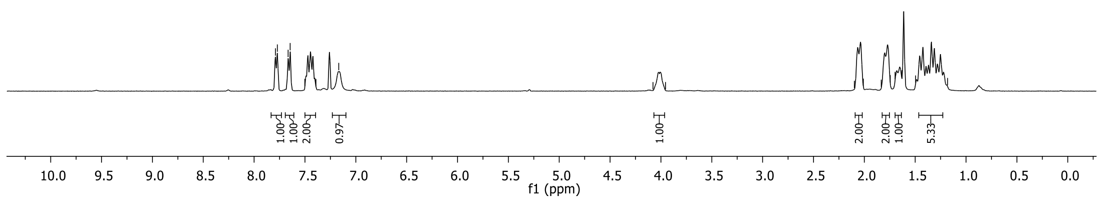
7.79
7.77
7.67
7.65
7.50
7.39
7.17
4.08
3.95
2.10
2.01
1.83
1.75
1.70
1.63
1.50
1.18
1.00
1.00
2.00
0.97
1.00
2.00
2.00
1.00
5.33
10.0 9.5 9.0 8.5 8.0 7.5 7.0 6.5 6.0 5.5 5.0 4.5 4.0 3.5 3.0 2.5 2.0 1.5 1.0 0.5 0.0
f1 (ppm)

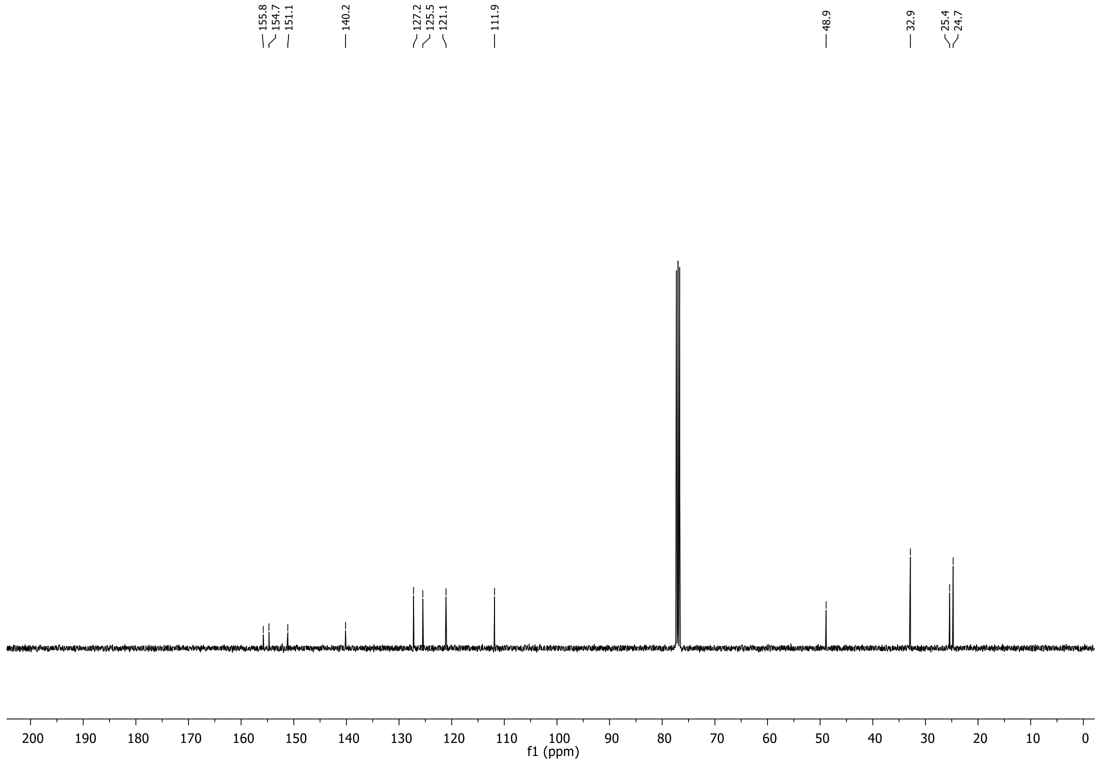
155.8
154.7
151.1
140.2
127.2
125.5
121.1
111.9
48.9
32.9
25.4
24.7
200 190 180 170 160 150 140 130 120 110 100 90 80 70 60 50 40 30 20 10 0
f1 (ppm)

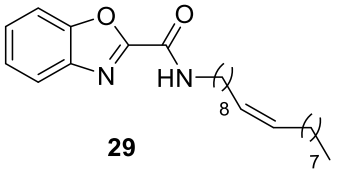
O
O
N
HN
8
7

29

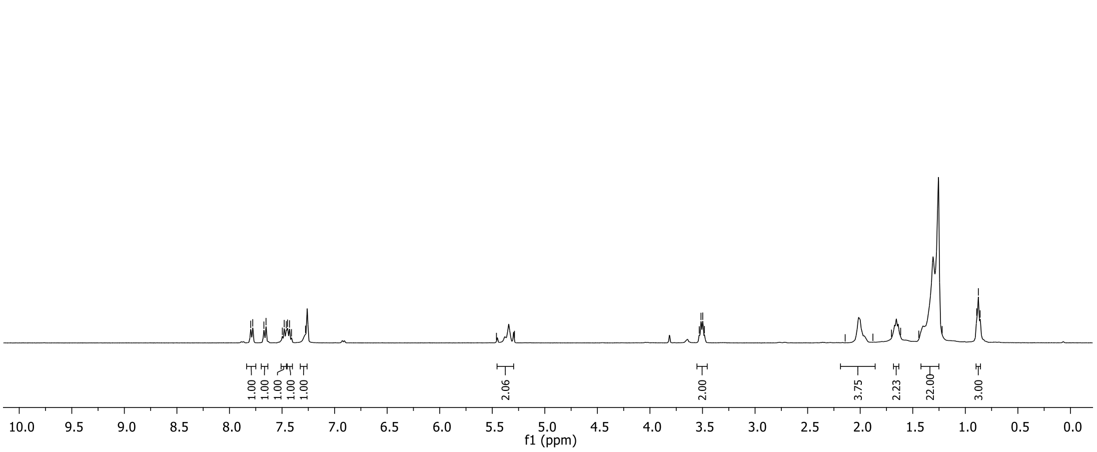
7.80
7.78
7.67
7.65
7.50
7.48
7.46
7.45
7.43
7.41
7.28
5.46
5.29
3.53
3.51
3.50
3.48
2.14
1.88
1.70
1.62
1.44
1.22
0.89
0.88
0.86
1.00
1.00
1.00
1.00
1.00
2.06
2.00
3.75
2.23
22.00
3.00
f1 (ppm)

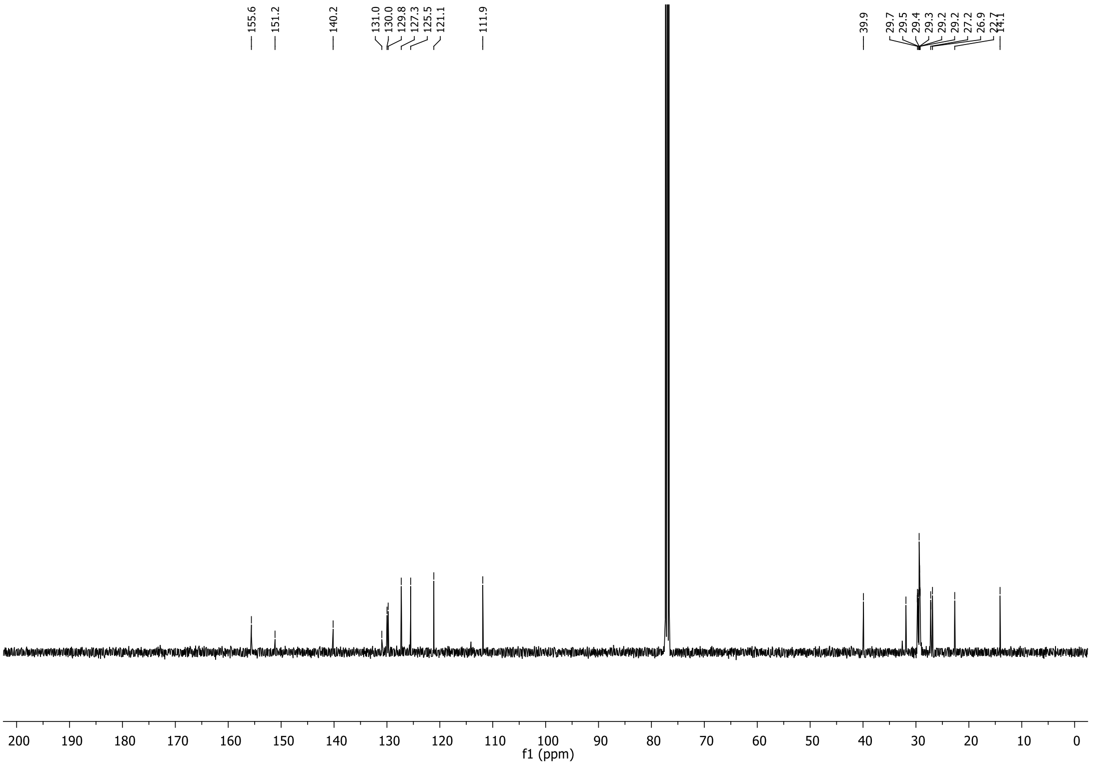
155.6
151.2
140.2
131.0
130.0
129.8
127.3
125.5
121.1
111.9
39.9
29.7
29.5
29.4
29.3
29.2
29.2
27.2
26.9
22.7
14.1
f1 (ppm)

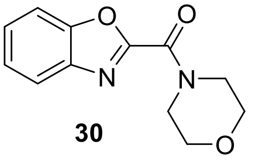

30

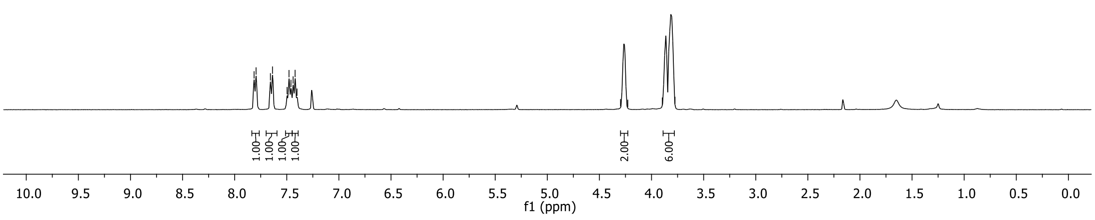
7.81
7.79
7.66
7.64
7.50
7.48
7.46
7.44
7.42
7.40
4.30
4.23
3.90
3.77
1.00
1.00
1.00
1.00
2.00
6.00
10.0 9.5 9.0 8.5 8.0 7.5 7.0 6.5 6.0 5.5 5.0 4.5 4.0 3.5 3.0 2.5 2.0 1.5 1.0 0.5 0.0
f1 (ppm)

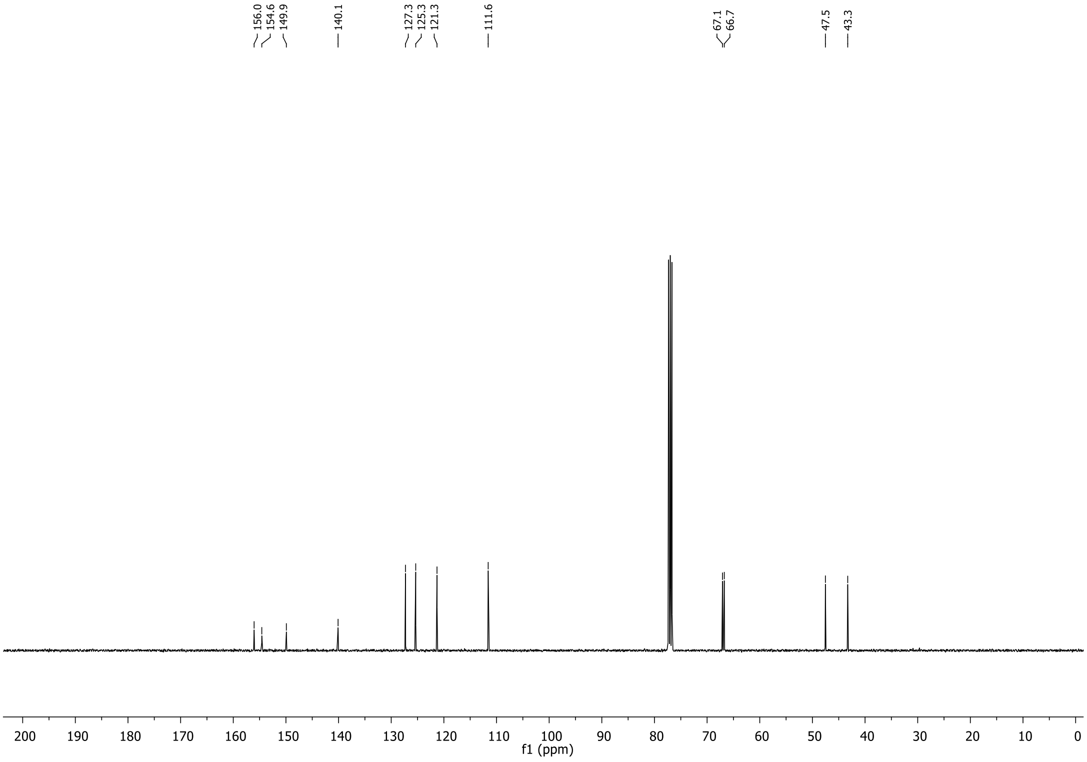
156.0
154.6
149.9
140.1
127.3
125.3
121.3
111.6
67.1
66.7
47.5
43.3
200 190 180 170 160 150 140 130 120 110 100 90 80 70 60 50 40 30 20 10 0
f1 (ppm)

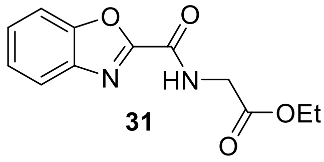
O
O
N
HN
OEt
O

31

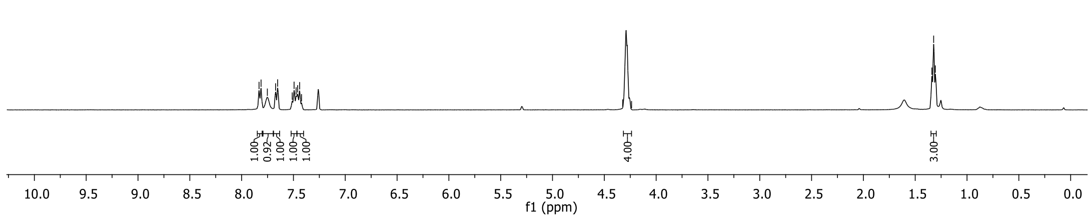
7.83
7.81
7.75
7.67
7.65
7.51
7.49
7.47
7.46
7.44
7.42
4.32
4.24
1.34
1.32
1.31
1.00
0.92
1.00
1.00
1.00
4.00
3.00
10.0 9.5 9.0 8.5 8.0 7.5 7.0 6.5 6.0 5.5 5.0 4.5 4.0 3.5 3.0 2.5 2.0 1.5 1.0 0.5 0.0
f1 (ppm)

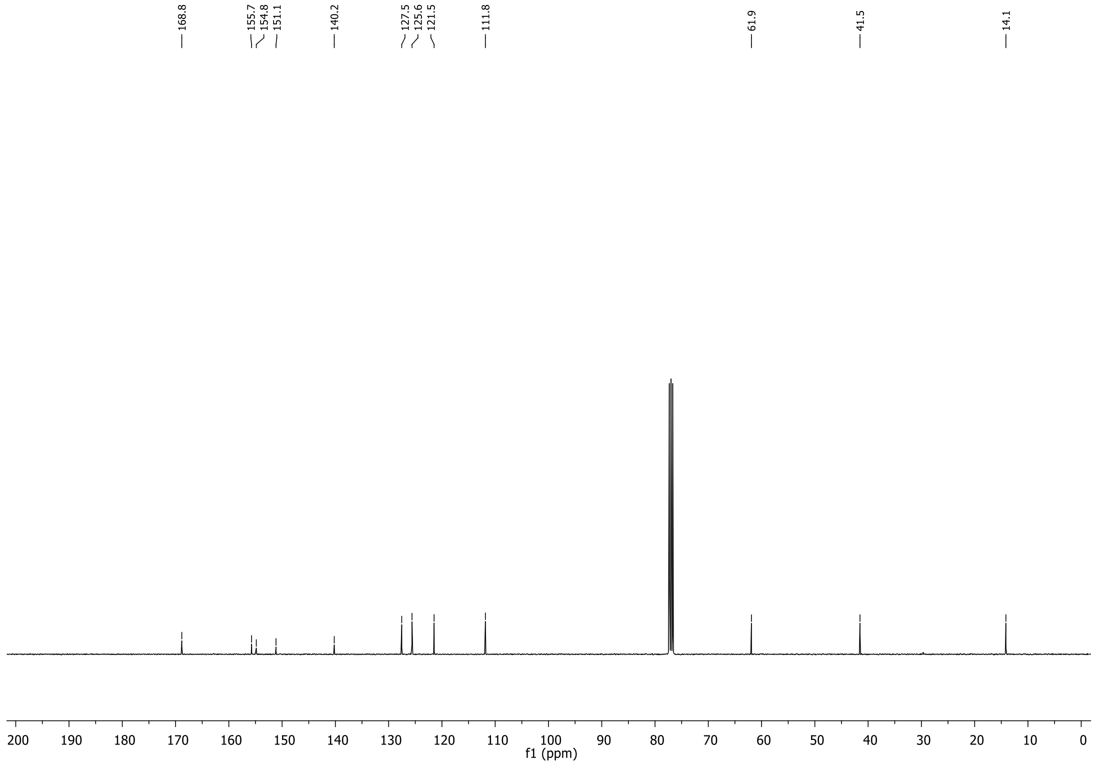
168.8
155.7
154.8
151.1
140.2
127.5
125.6
121.5
111.8
61.9
41.5
14.1
200 190 180 170 160 150 140 130 120 110 100 90 80 70 60 50 40 30 20 10 0
f1 (ppm)

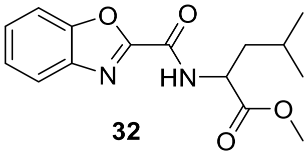
O
O
N
HN
O
O

32

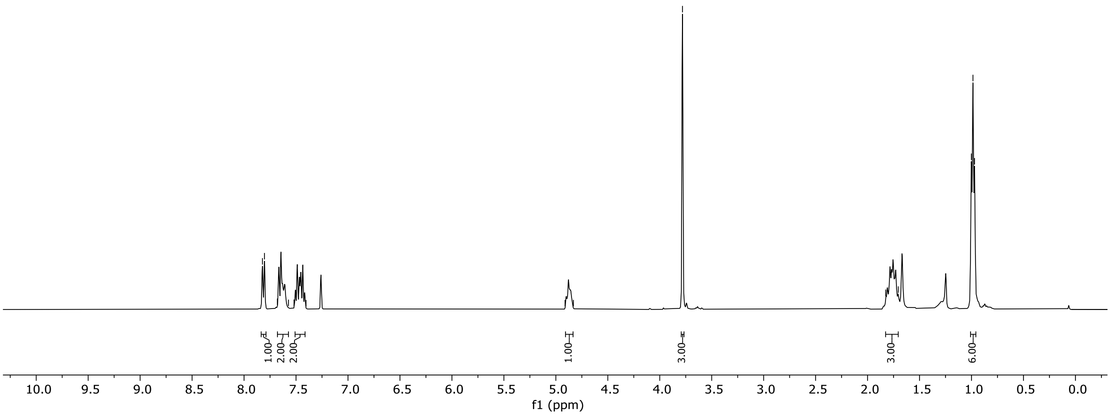
7.82
7.80
7.68
7.57
7.52
7.40
4.91
4.83
3.78
1.83
1.70
1.00
0.99
0.97
1.00
2.00
2.00
1.00
3.00
3.00
6.00
f1 (ppm)

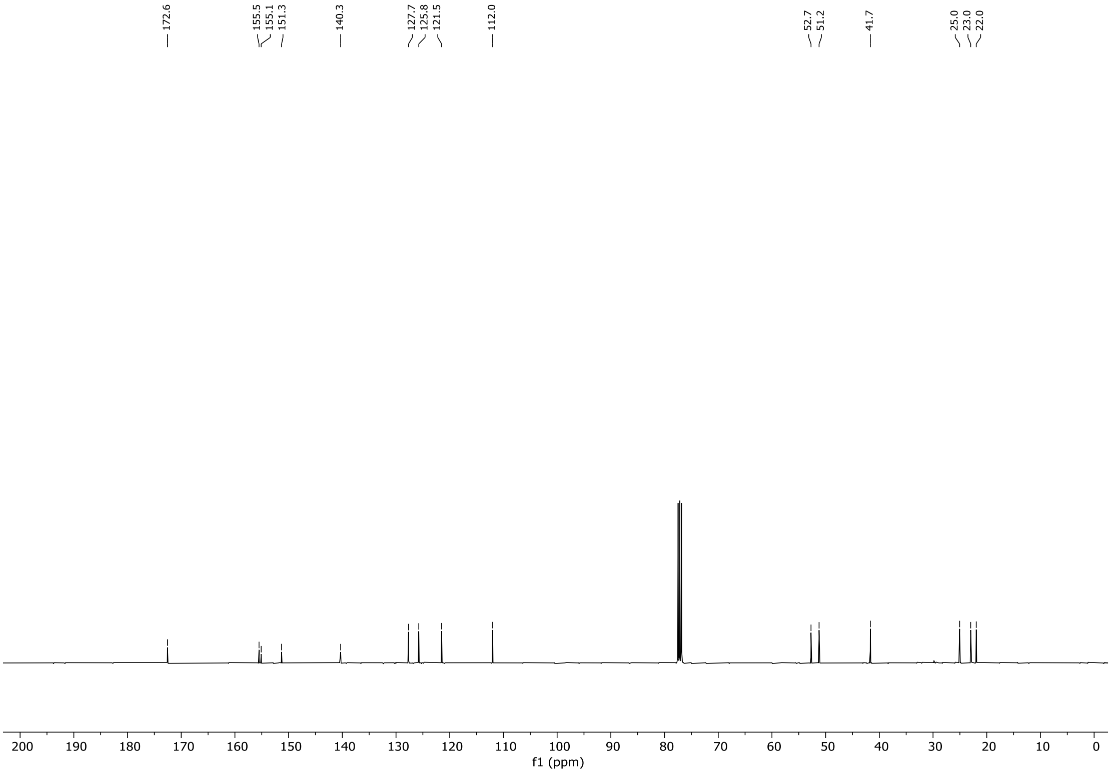
172.6
155.5
155.1
151.3
140.3
127.7
125.8
121.5
112.0
52.7
51.2
41.7
25.0
23.0
22.0
f1 (ppm)

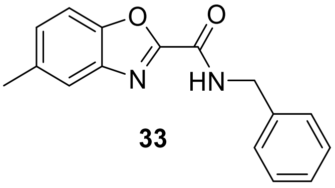
O
O
N
HN

33

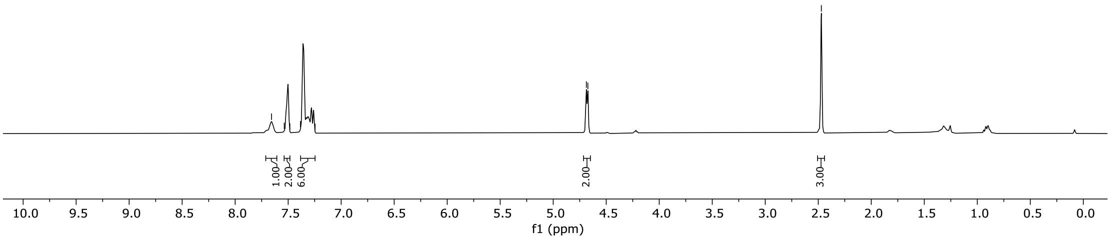
7.66
7.54
7.48
7.38
7.25
4.69
4.67
2.47
1.00
2.00
6.00
2.00
3.00
f1 (ppm)

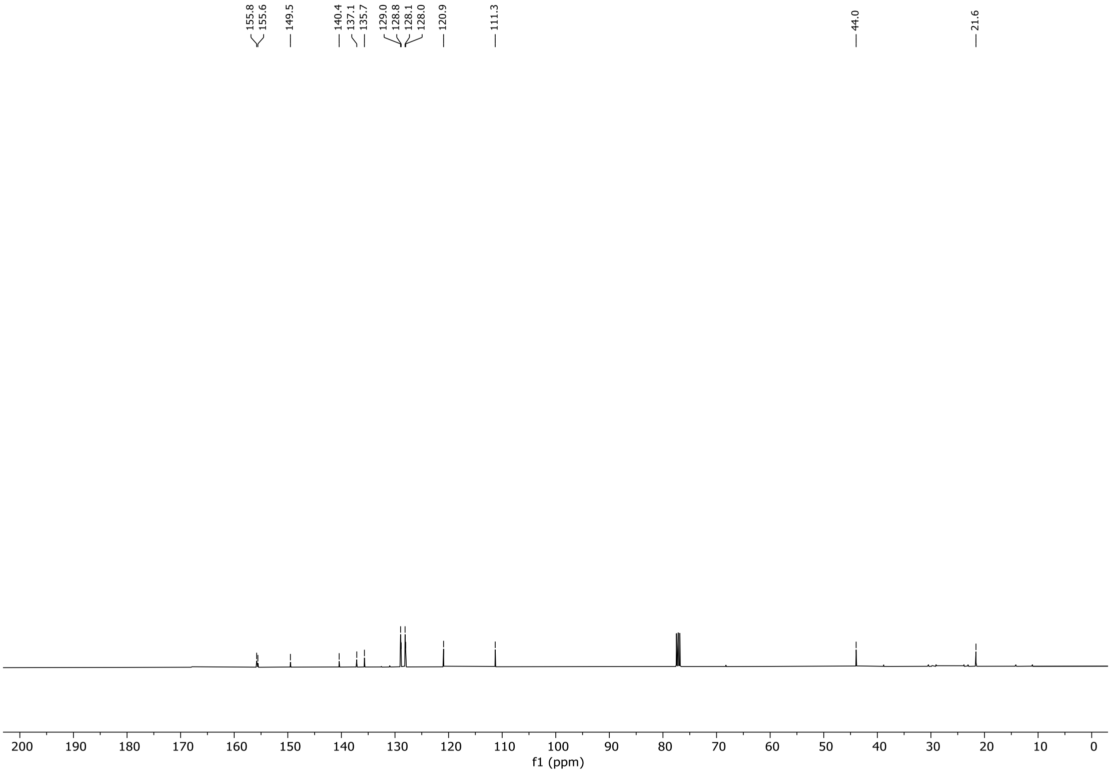
155.8
155.6
149.5
140.4
137.1
135.7
129.0
128.8
128.1
128.0
120.9
111.3
44.0
21.6
f1 (ppm)

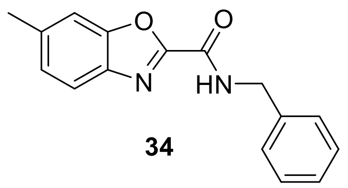
O
N
HN

34

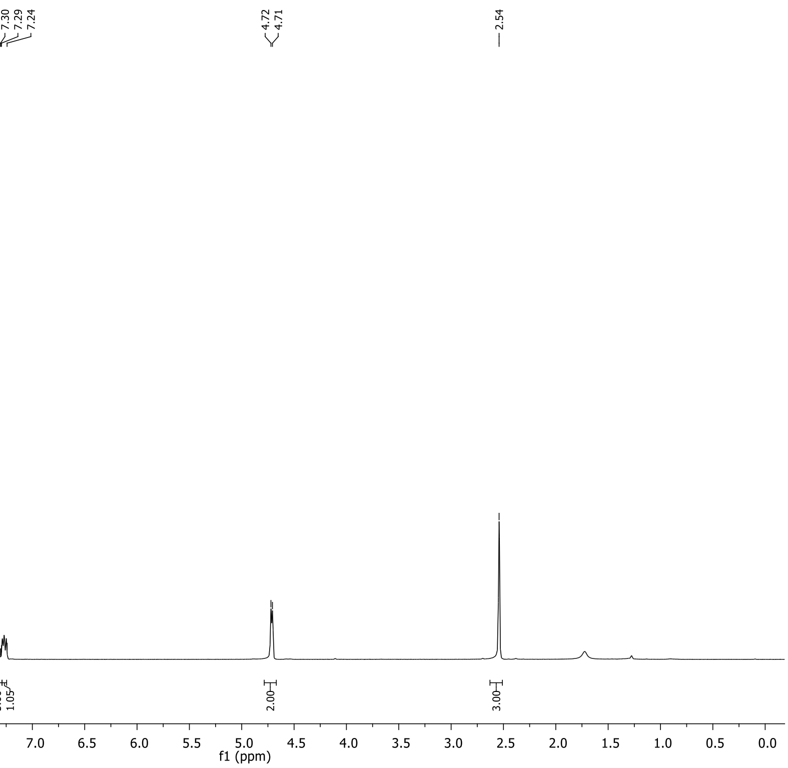
7.66
7.63
7.60
7.47
7.44
7.30
7.29
7.24
4.72
4.71
2.54
1.00
1.00
1.00
5.00
1.05
2.00
3.00
10.0 9.5 9.0 8.5 8.0 7.5 7.0 6.5 6.0 5.5 5.0 4.5 4.0 3.5 3.0 2.5 2.0 1.5 1.0 0.5 0.0
f1 (ppm)

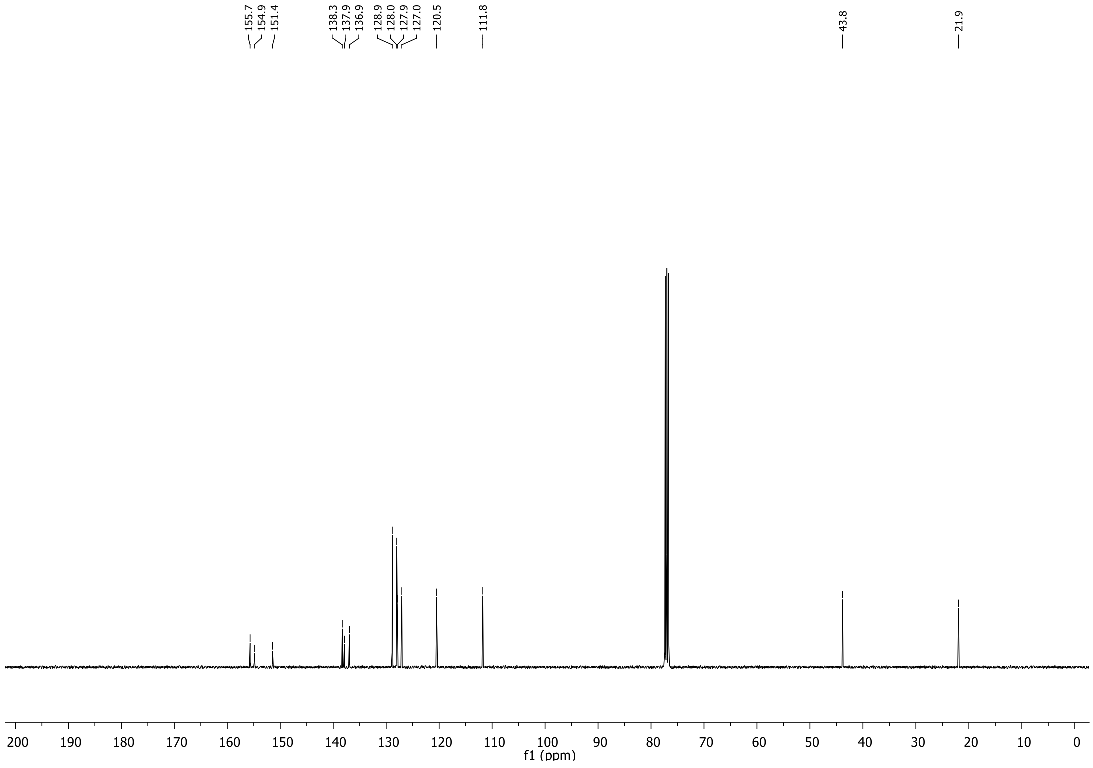
155.7
154.9
151.4
138.3
137.9
136.9
128.9
128.0
127.9
127.0
120.5
111.8
43.8
21.9
200 190 180 170 160 150 140 130 120 110 100 90 80 70 60 50 40 30 20 10 0
f1 (ppm)

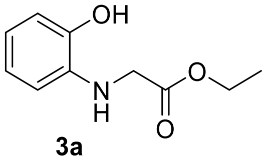
OH
N
H
O
O

3a

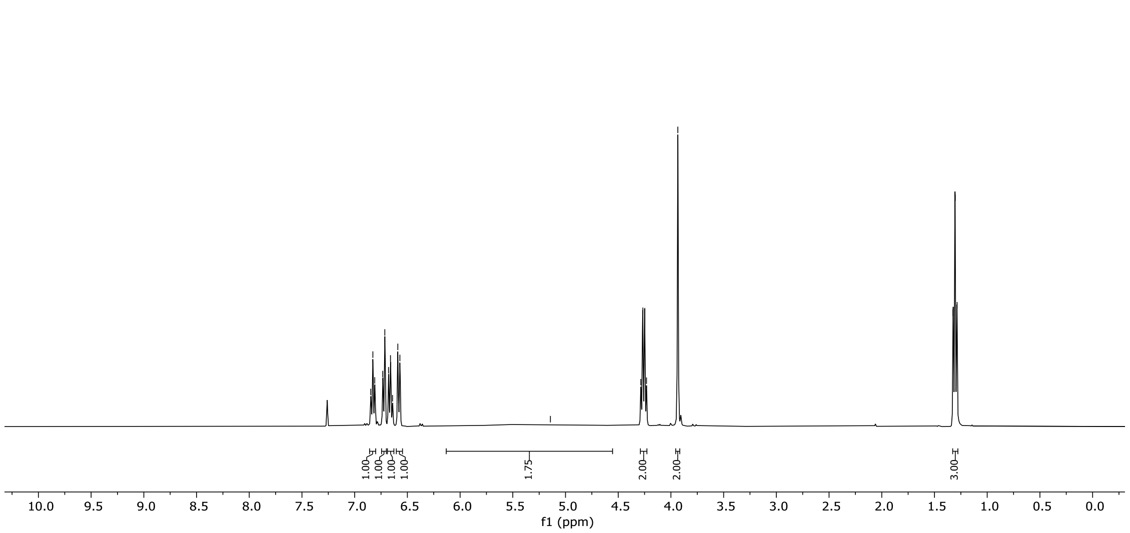
6.85
6.83
6.81
6.73
6.71
6.68
6.66
6.64
6.59
6.57
5.14
4.29
4.27
4.25
4.23
3.93
1.32
1.30
1.29
1.00
1.00
1.00
1.00
1.75
2.00
2.00
3.00
f1 (ppm)

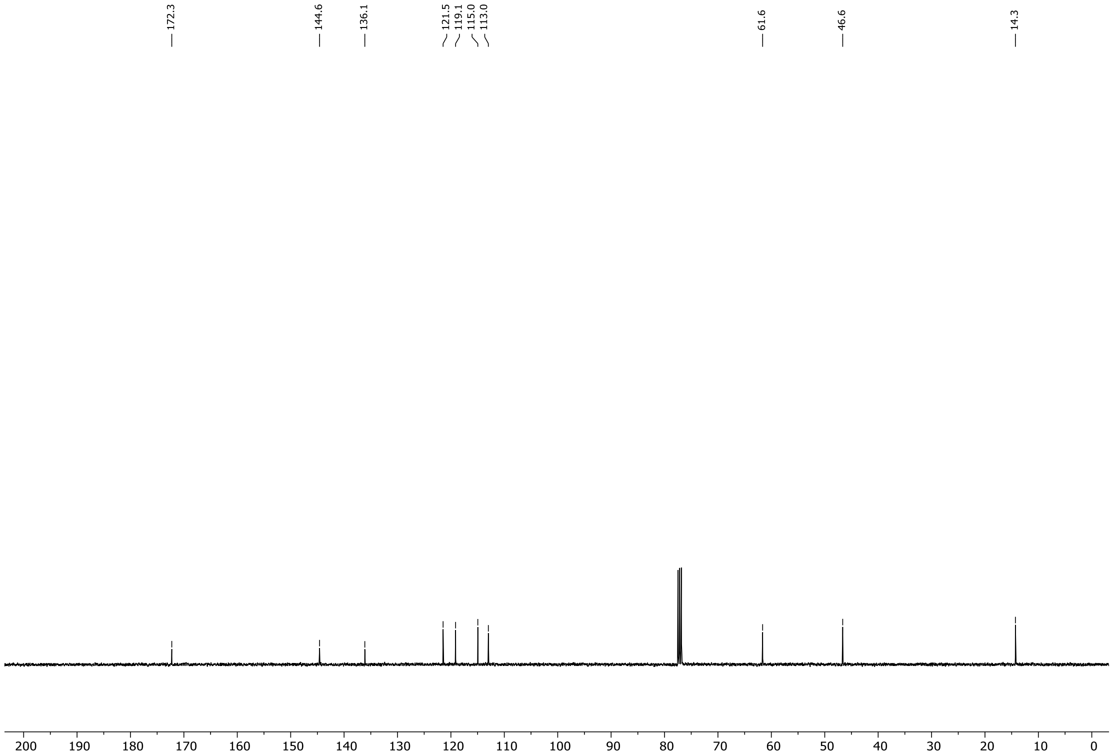
172.3
144.6
136.1
121.5
119.1
115.0
113.0
61.6
46.6
14.3
f1 (ppm)

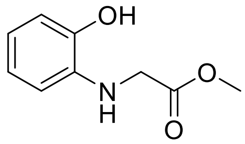
OH
N
H
O
O

3b

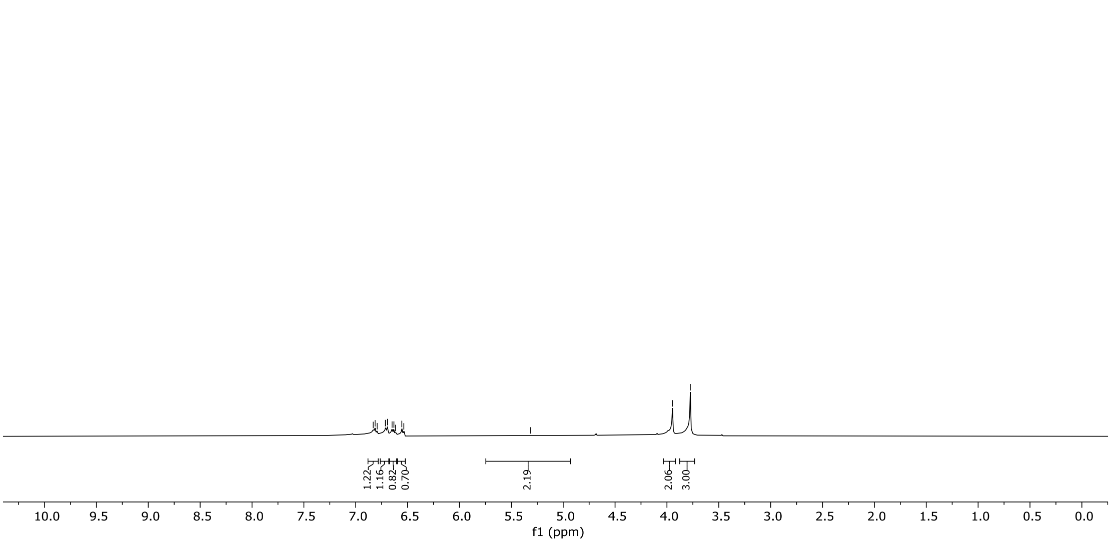
6.83
6.81
6.79
6.71
6.69
6.65
6.63
6.62
6.56
6.54
5.31
3.95
3.77
1.22
1.16
0.82
0.70
2.19
2.06
3.00
10.0 9.5 9.0 8.5 8.0 7.5 7.0 6.5 6.0 5.5 5.0 4.5 4.0 3.5 3.0 2.5 2.0 1.5 1.0 0.5 0.0
f1 (ppm)

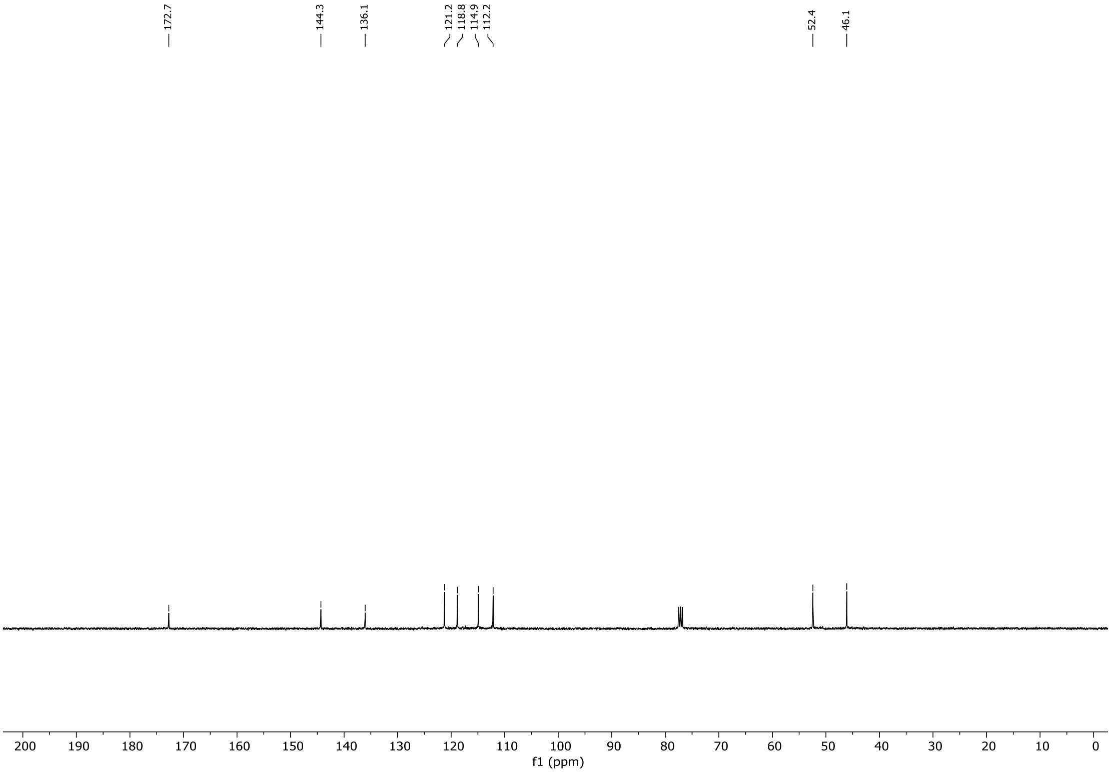
172.7
144.3
136.1
121.2
118.8
114.9
112.2
52.4
46.1
200 190 180 170 160 150 140 130 120 110 100 90 80 70 60 50 40 30 20 10 0
f1 (ppm)

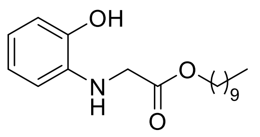
OH
N
H
O
O
9

3c

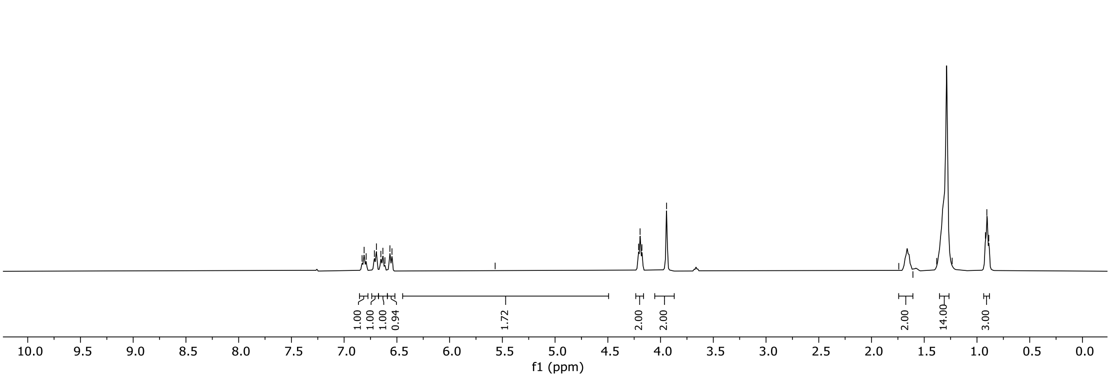
6.83
6.81
6.79
6.71
6.69
6.65
6.63
6.61
6.57
6.55
5.57
4.21
4.19
4.18
3.94
1.74
1.61
1.38
1.23
0.92
0.91
0.89
1.00
1.00
1.00
0.94
1.72
2.00
2.00
2.00
14.00
3.00
f1 (ppm)

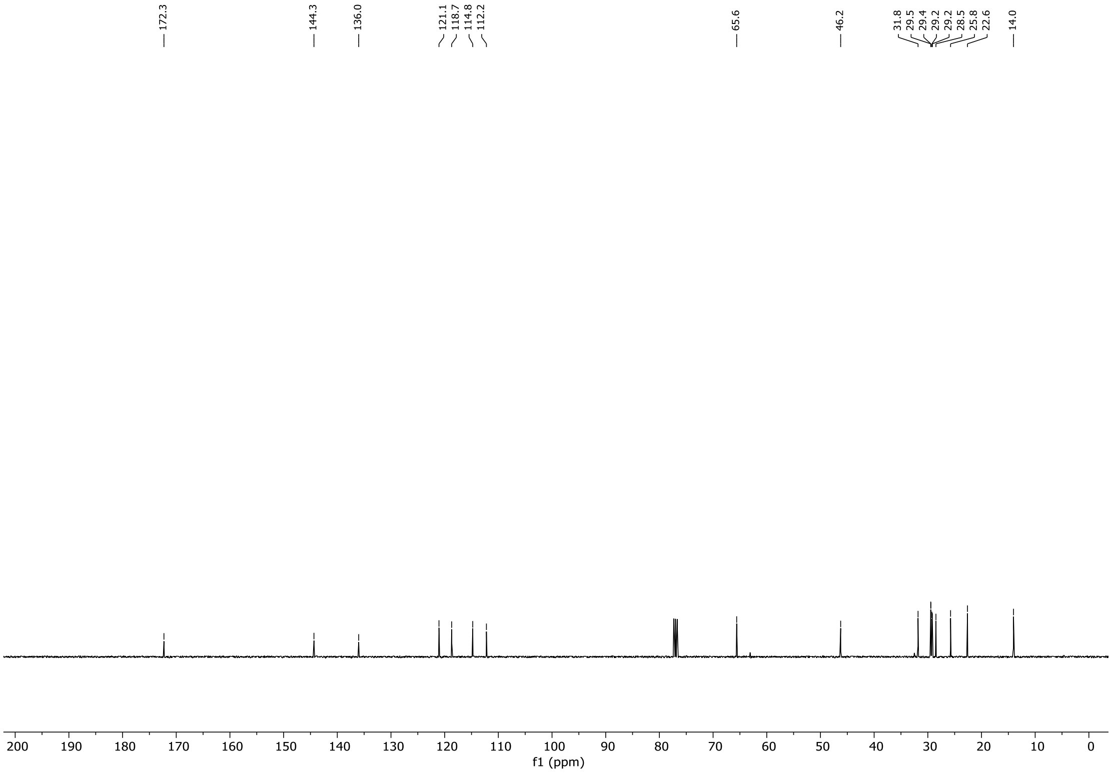
172.3
144.3
136.0
121.1
118.7
114.8
112.2
65.6
46.2
31.8
29.5
29.4
29.2
29.2
28.5
25.8
22.6
14.0
f1 (ppm)

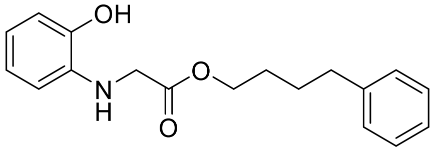
OH
N
H
O
O

3d

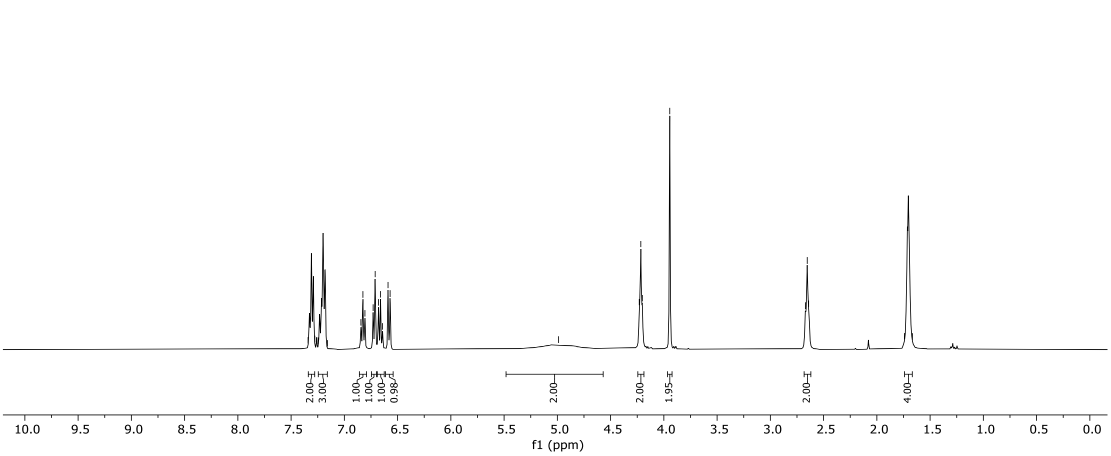
7.34
7.28
7.25
7.16
6.84
6.83
6.81
6.73
6.71
6.68
6.66
6.64
6.59
6.57
4.99
4.23
4.22
4.20
3.94
2.67
2.65
2.64
1.74
1.67
2.00
3.00
1.00
1.00
1.00
0.98
2.00
2.00
1.95
2.00
4.00
f1 (ppm)

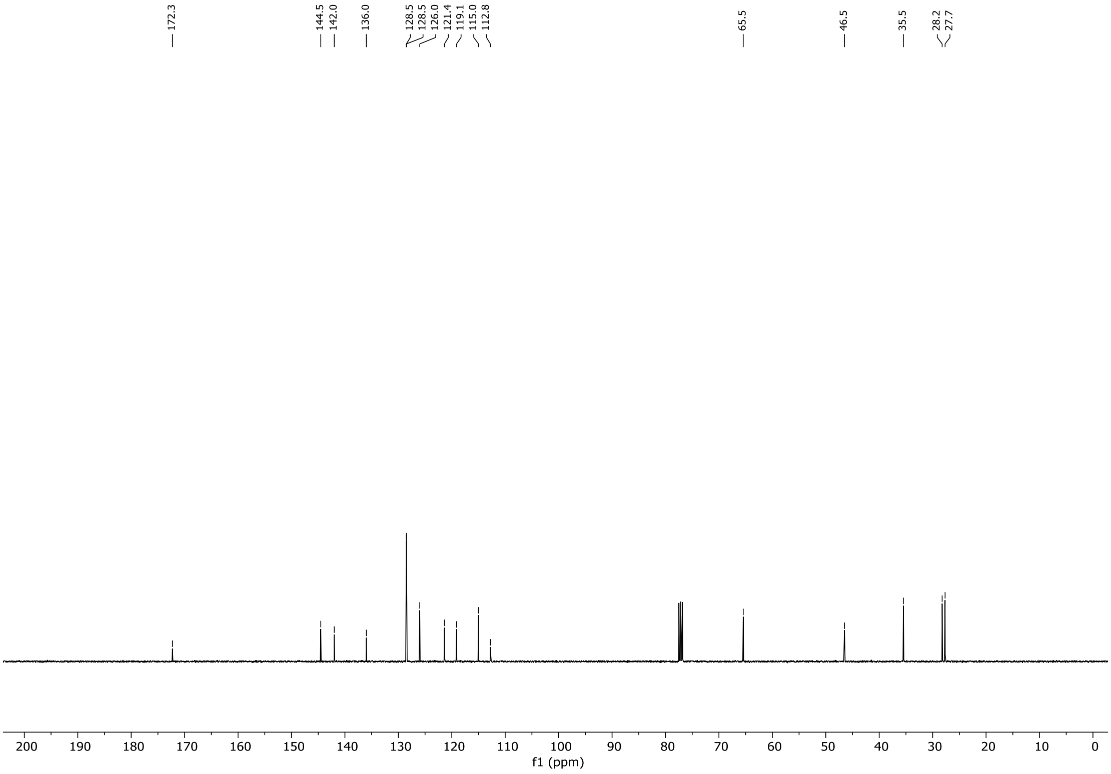
172.3
144.5
142.0
136.0
128.5
128.5
126.0
121.4
119.1
115.0
112.8
65.5
46.5
35.5
28.2
27.7
f1 (ppm)

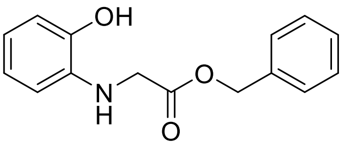

3e

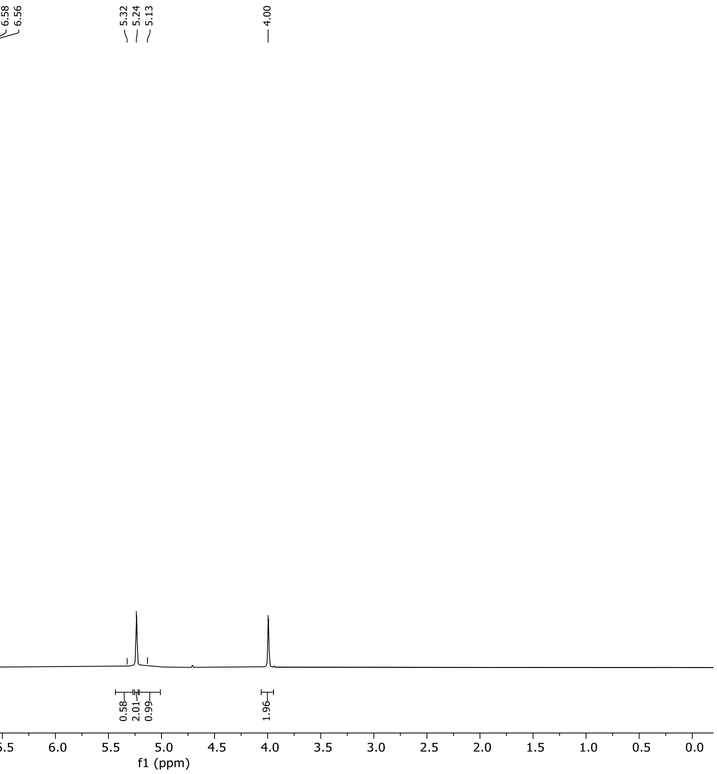
7.46
7.31
6.85
6.83
6.81
6.74
6.64
6.58
6.56
5.32
5.24
5.13
4.00
5.04
1.01
2.02
0.96
0.58
2.01
0.99
1.96
f1 (ppm)

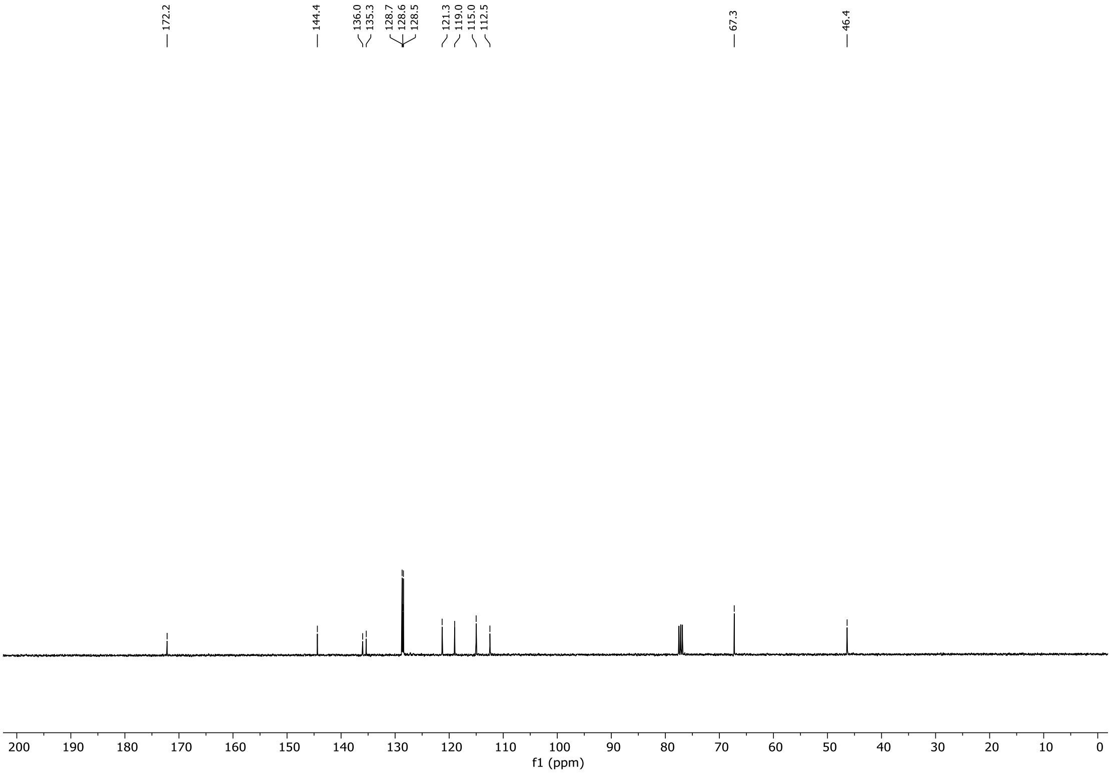
172.2
144.4
136.0
135.3
128.7
128.6
128.5
121.3
119.0
115.0
112.5
67.3
46.4
f1 (ppm)

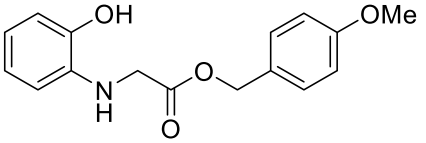
OH
OMe
N
H
O
O

3f

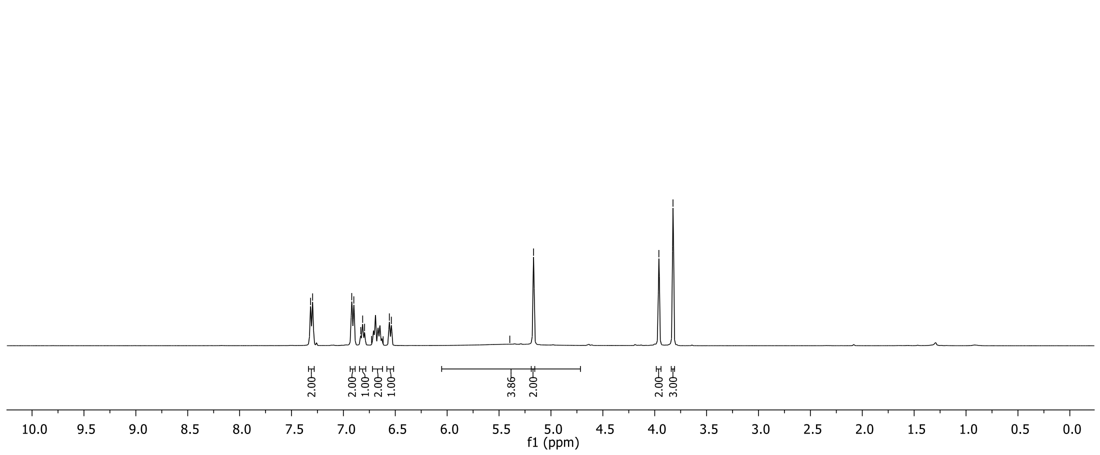
7.32
7.30
6.92
6.90
6.83
6.81
6.80
6.73
6.62
6.56
6.54
5.40
5.17
3.96
3.82
2.00
2.00
1.00
2.00
1.00
3.86
2.00
2.00
3.00
10.0 9.5 9.0 8.5 8.0 7.5 7.0 6.5 6.0 5.5 5.0 4.5 4.0 3.5 3.0 2.5 2.0 1.5 1.0 0.5 0.0
f1 (ppm)

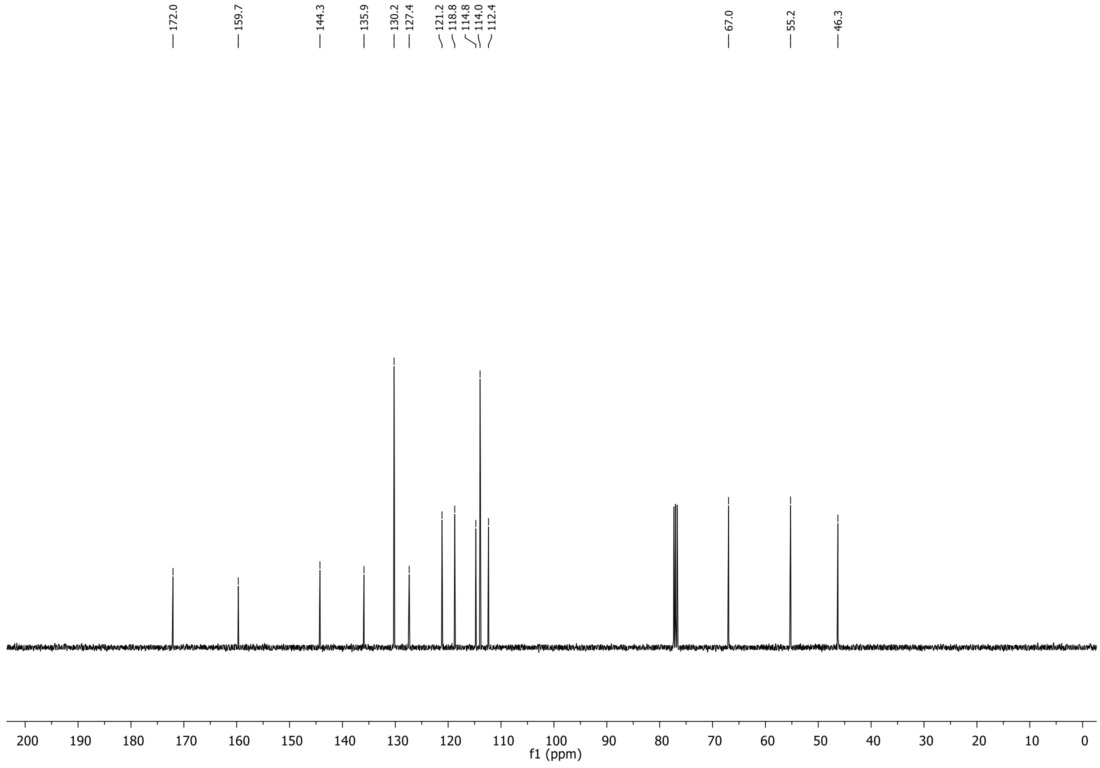
172.0
159.7
144.3
135.9
130.2
127.4
121.2
118.8
114.8
114.0
112.4
67.0
55.2
46.3
200 190 180 170 160 150 140 130 120 110 100 90 80 70 60 50 40 30 20 10 0
f1 (ppm)

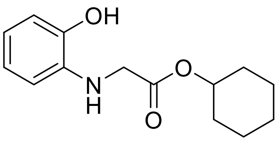
OH
N
H
O
O

3g

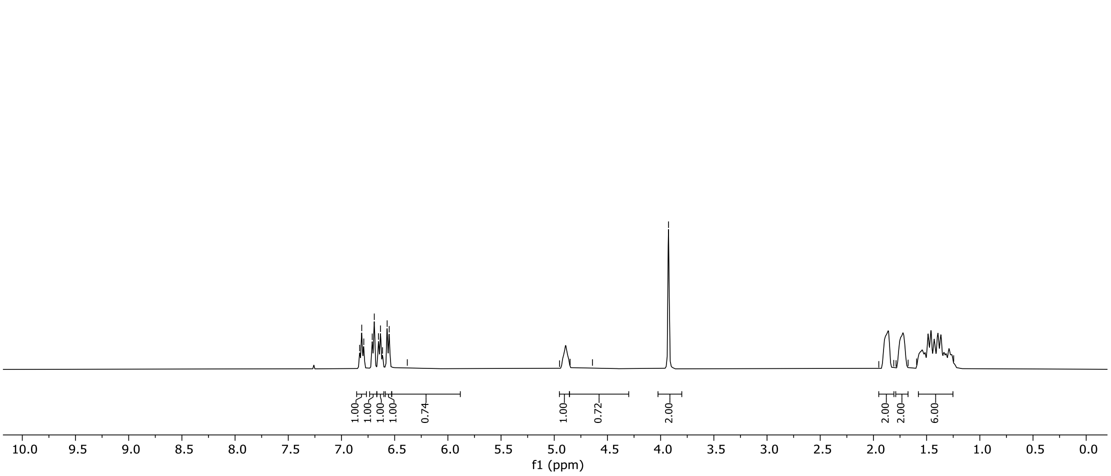
6.83
6.81
6.79
6.71
6.69
6.65
6.63
6.61
6.57
6.55
6.38
4.95
4.85
4.64
3.93
1.95
1.81
1.79
1.67
1.59
1.25
1.00
1.00
1.00
1.00
0.74
1.00
0.72
2.00
2.00
2.00
6.00
f1 (ppm)

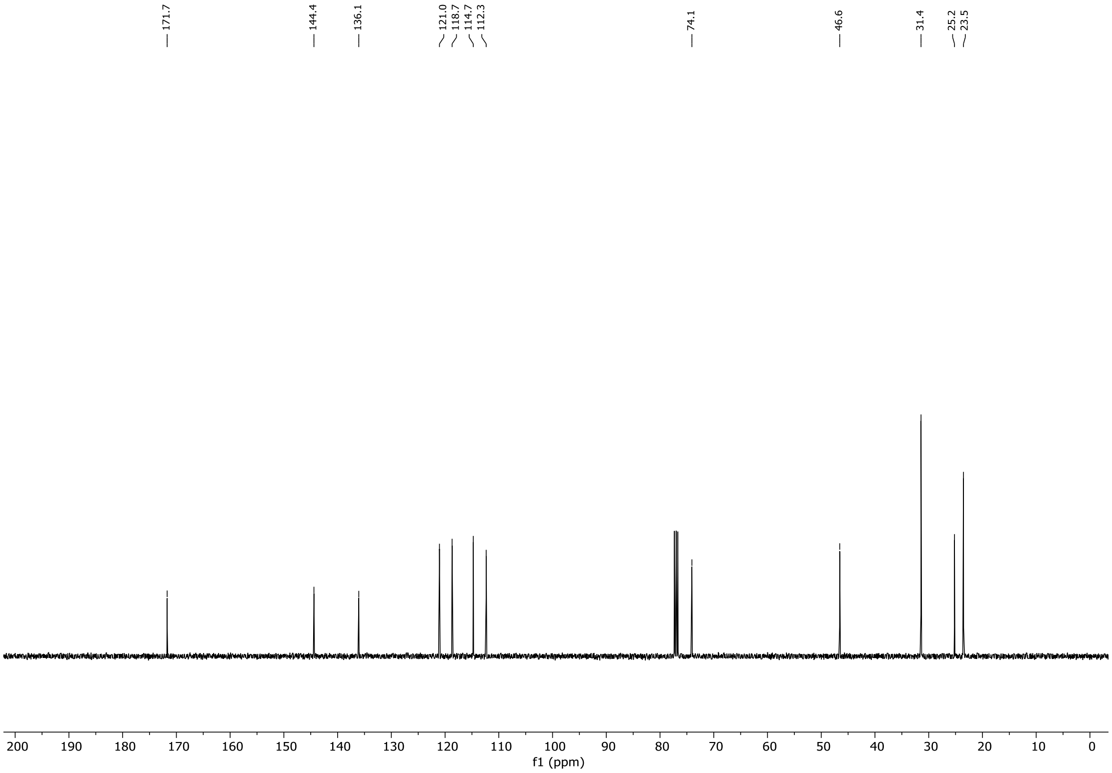
171.7
144.4
136.1
121.0
118.7
114.7
112.3
74.1
46.6
31.4
25.2
23.5
f1 (ppm)

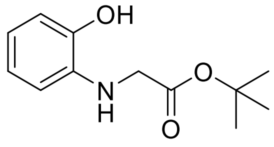

3h

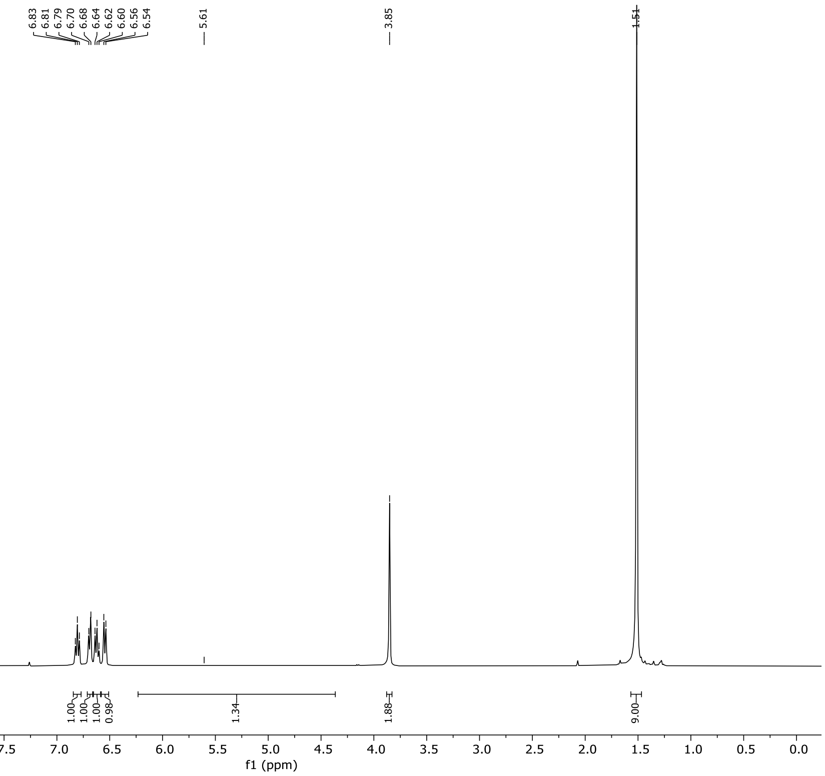

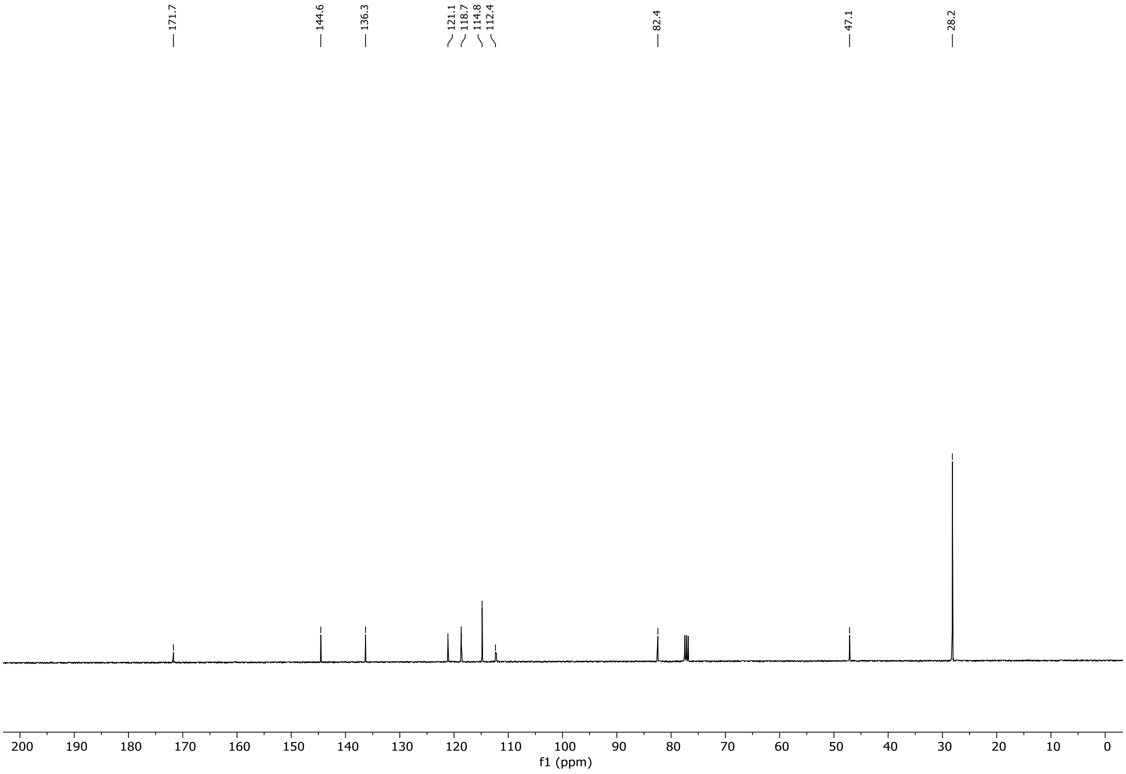

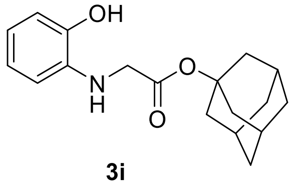

3i

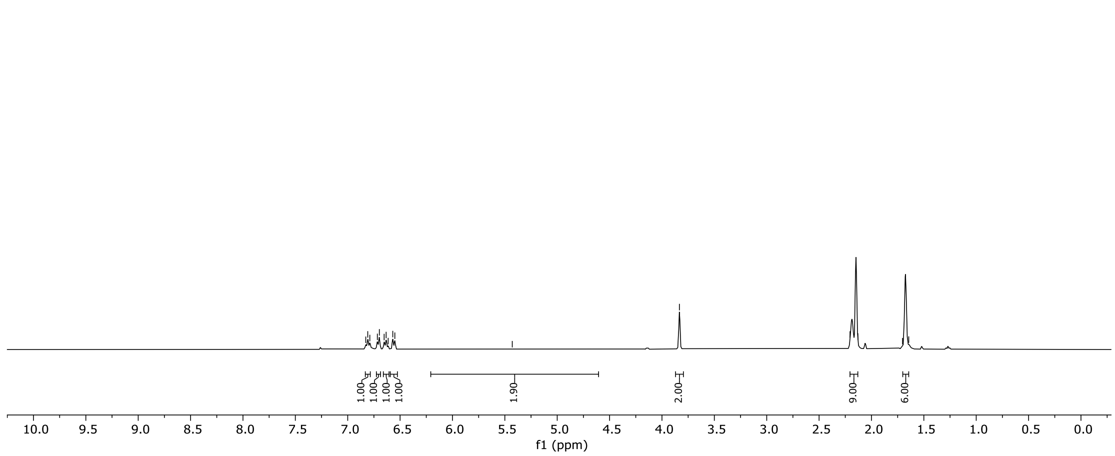

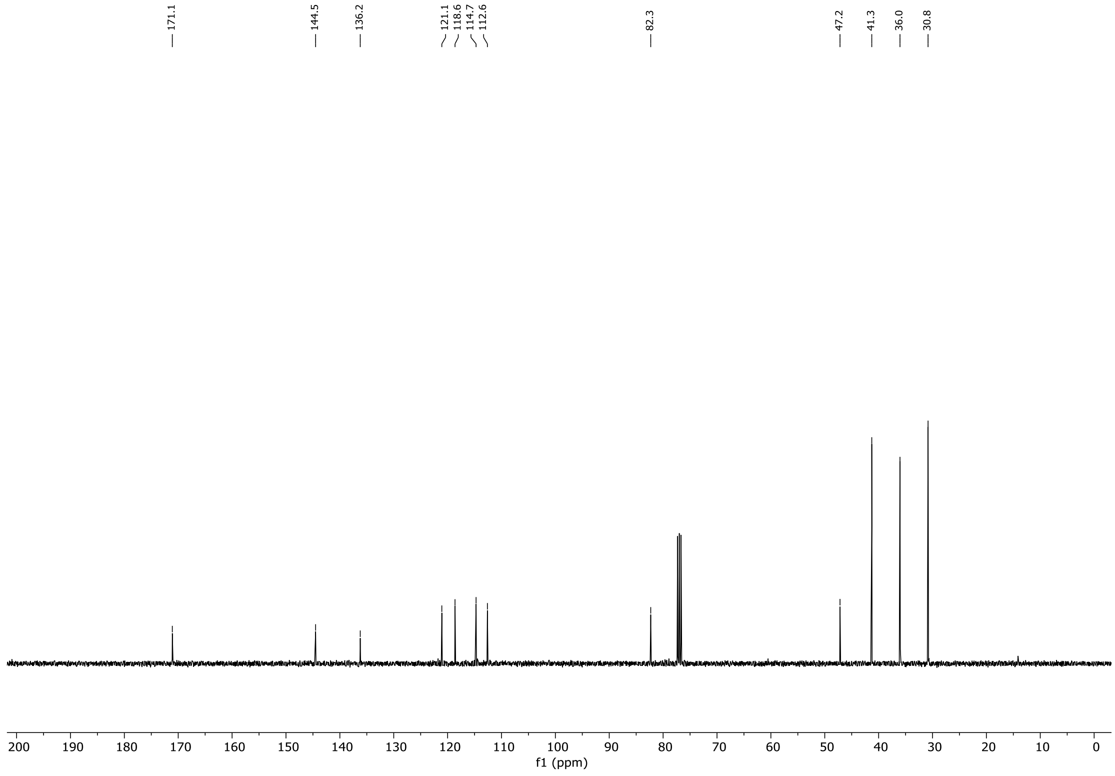

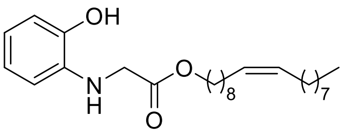
OH
N
H
O
O
8
7

3j

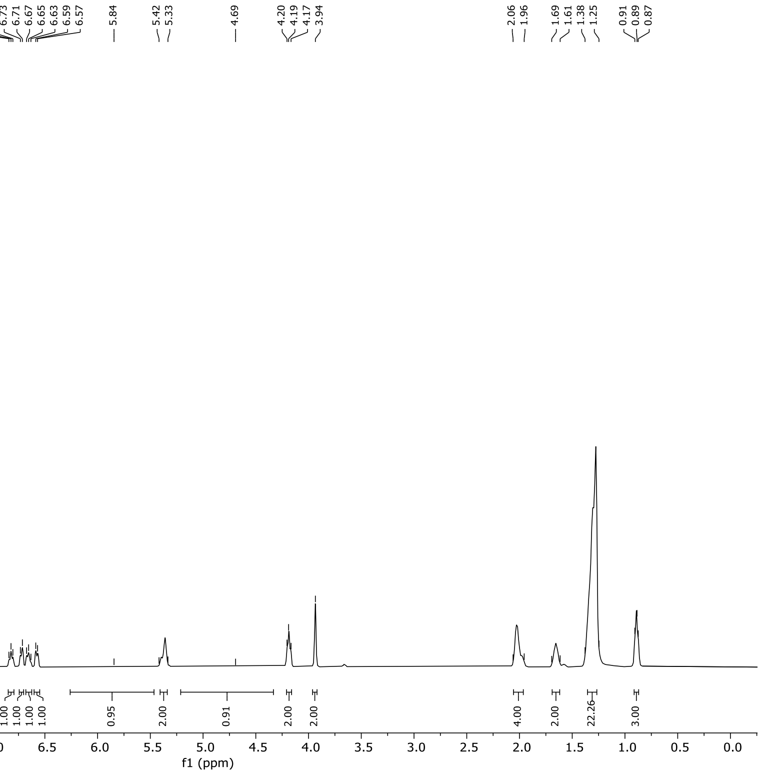
6.84 6.82 6.80 6.73 6.71 6.67 6.65 6.63 6.59 6.57
5.84
5.42 5.33
4.69
4.20 4.19 4.17 3.94
2.06 1.96
1.69 1.61 1.38 1.25
0.91 0.89 0.87
1.00 1.00 1.00 1.00
0.95
2.00
0.91
2.00
2.00
4.00
2.00
22.26
3.00
10.0 9.5 9.0 8.5 8.0 7.5 7.0 6.5 6.0 5.5 5.0 4.5 4.0 3.5 3.0 2.5 2.0 1.5 1.0 0.5 0.0
f1 (ppm)

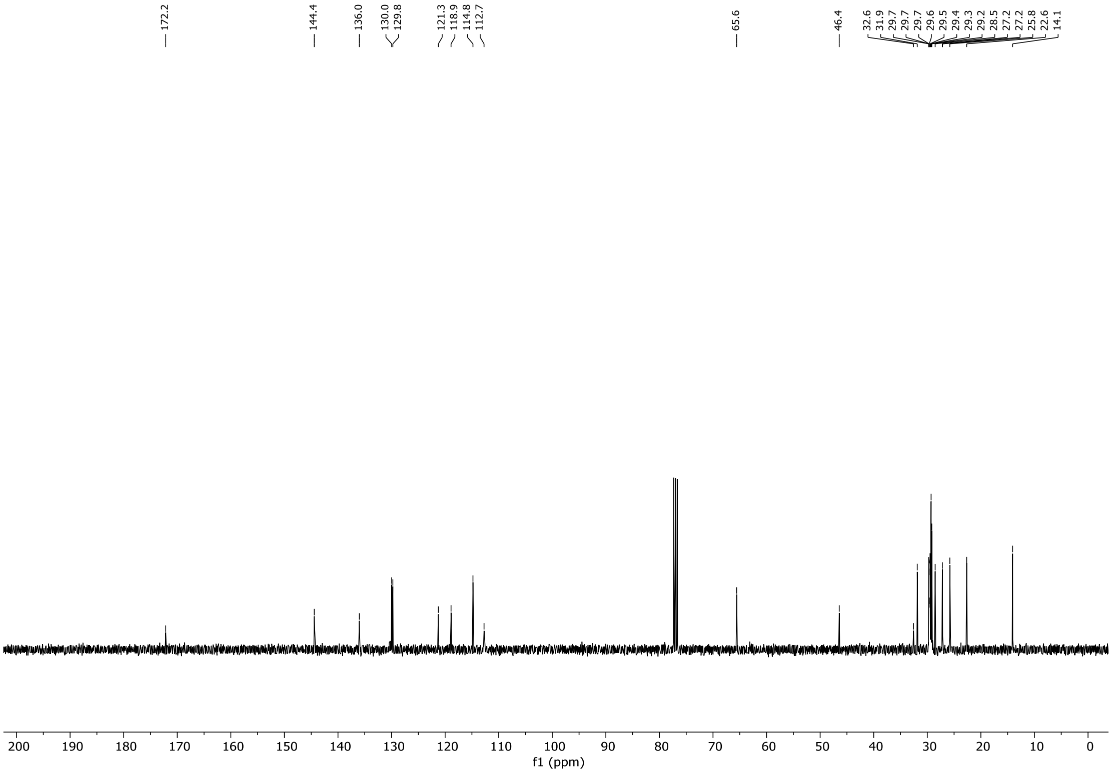
172.2
144.4
136.0
130.0 129.8
121.3 118.9 114.8 112.7
65.6
46.4
32.6 31.9 29.7 29.7 29.6 29.5 29.4 29.3 29.2 28.5 27.2 27.2 25.8 22.6 14.1
200 190 180 170 160 150 140 130 120 110 100 90 80 70 60 50 40 30 20 10 0
f1 (ppm)

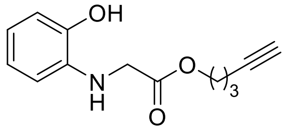
OH
N
H
O
O
3

3k

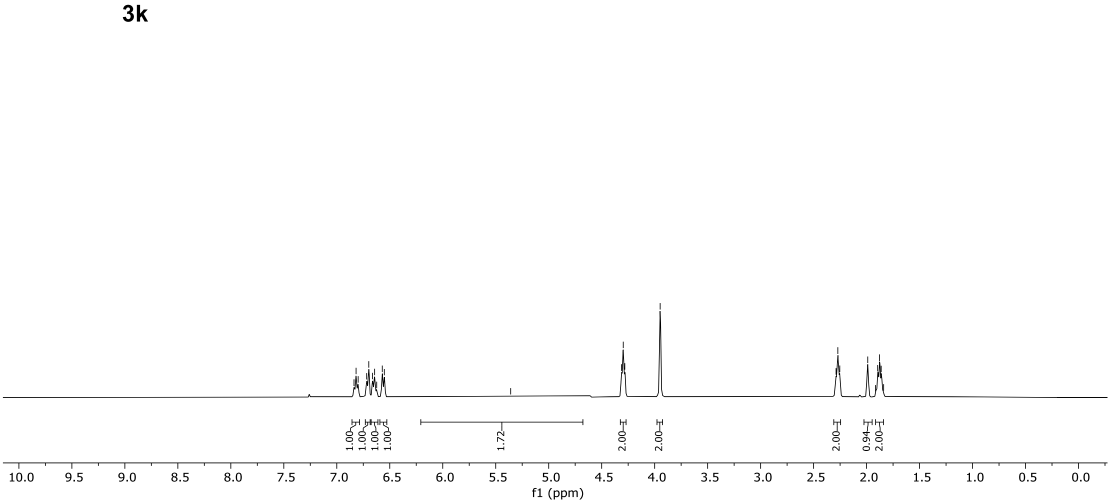
6.84
6.82
6.80
6.72
6.70
6.66
6.64
6.62
6.57
6.55
5.36
4.31
4.30
4.28
3.95
2.29
2.27
2.25
1.99
1.92
1.90
1.88
1.86
1.84
f1 (ppm)

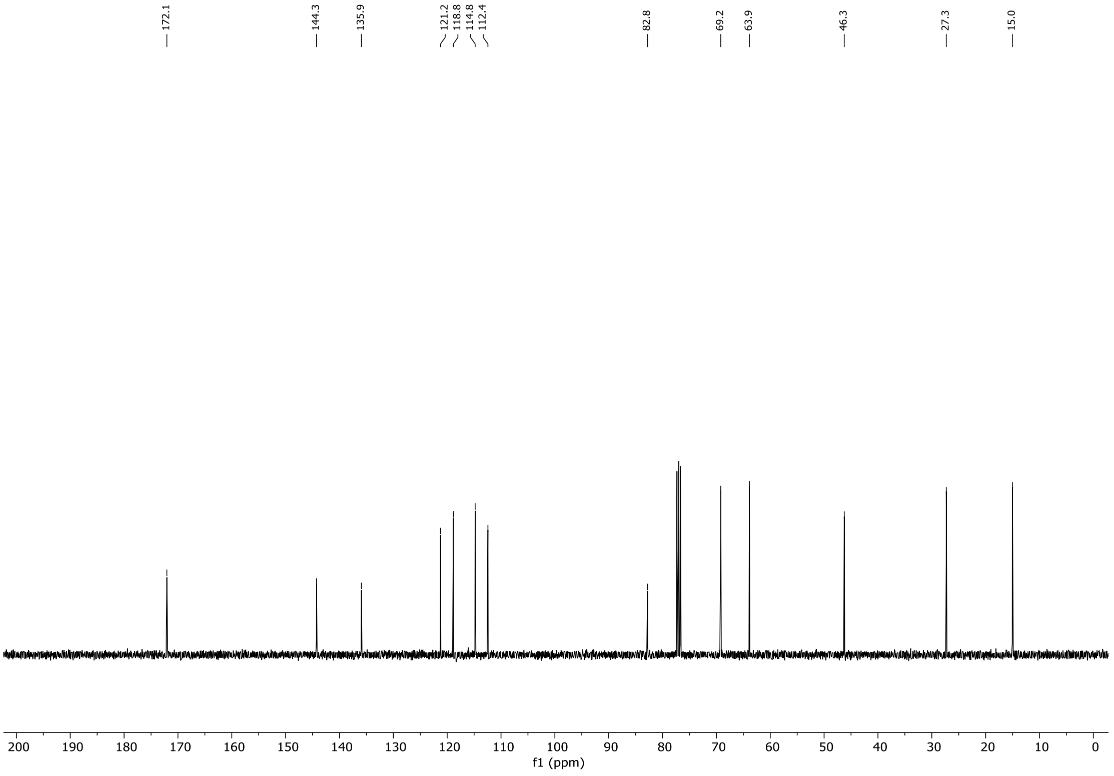
172.1
144.3
135.9
121.2
118.8
114.8
112.4
82.8
69.2
63.9
46.3
27.3
15.0
f1 (ppm)

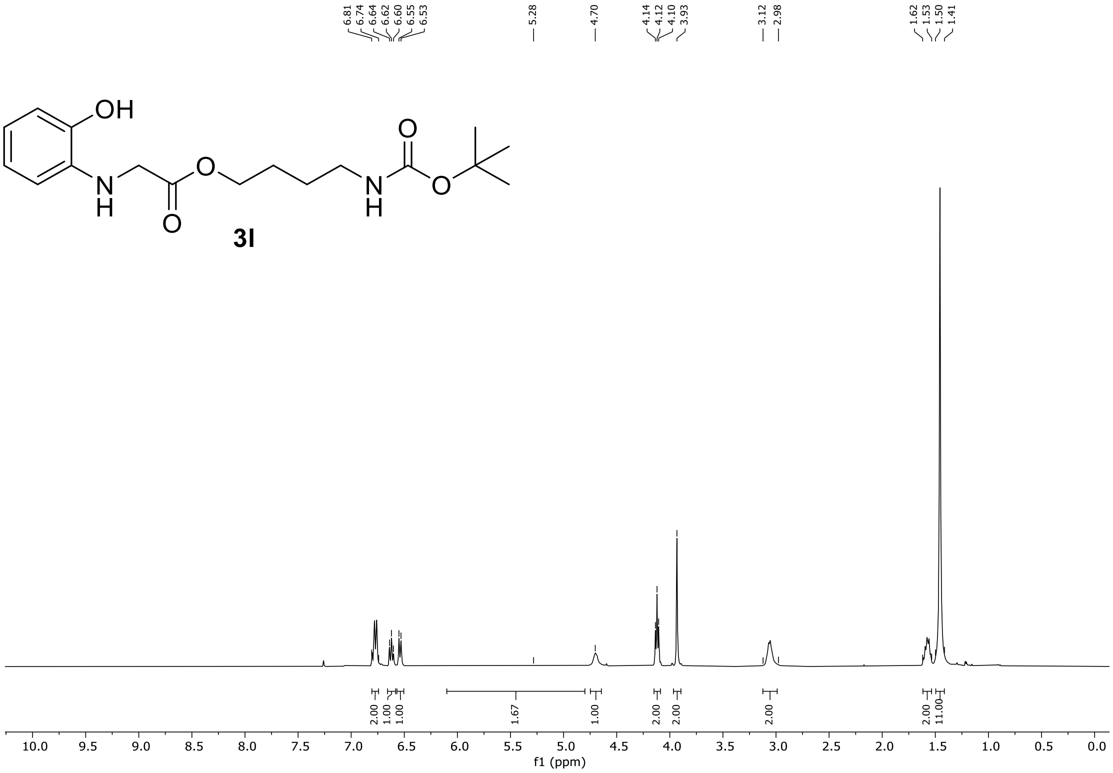
OH
N
H
O
O
O
N
H
O
O
3l
6.81
6.74
6.64
6.62
6.60
6.55
6.53
5.28
4.70
4.14
4.12
4.10
3.93
3.12
2.98
1.62
1.53
1.50
1.41
2.00
1.00
1.00
1.67
1.00
2.00
2.00
2.00
2.00
11.00
10.0 9.5 9.0 8.5 8.0 7.5 7.0 6.5 6.0 5.5 5.0 4.5 4.0 3.5 3.0 2.5 2.0 1.5 1.0 0.5 0.0
f1 (ppm)

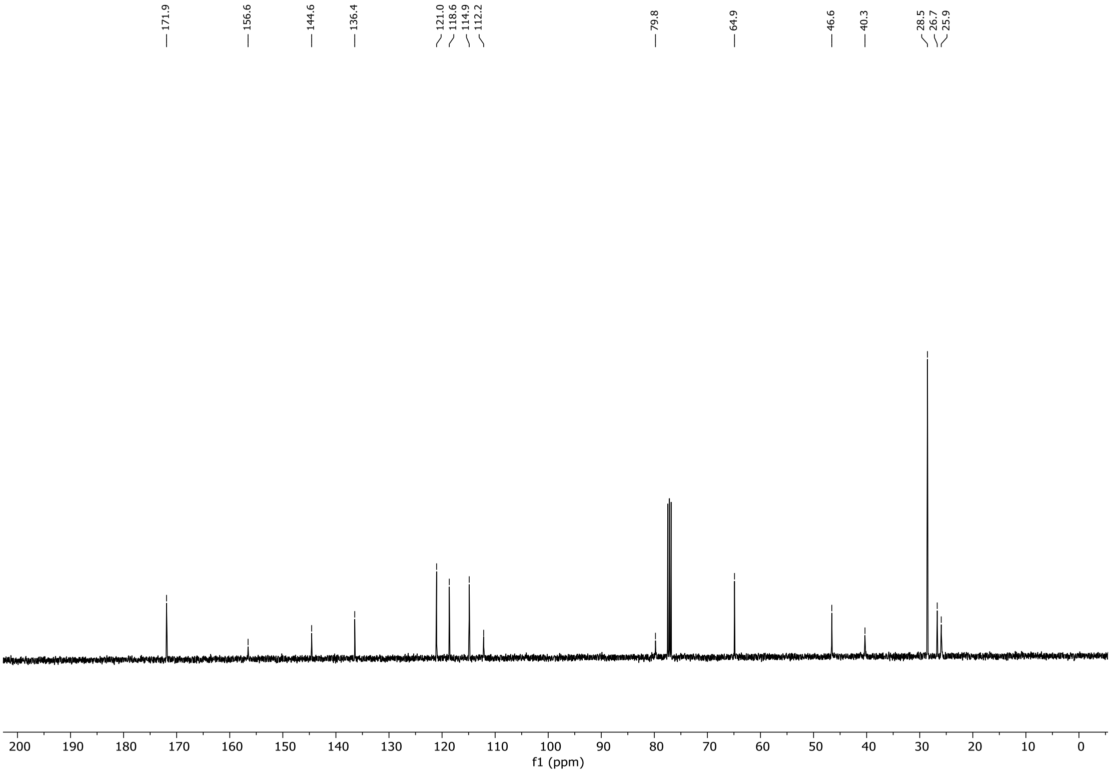
171.9
156.6
144.6
136.4
121.0
118.6
114.9
112.2
79.8
64.9
46.6
40.3
28.5
26.7
25.9
200 190 180 170 160 150 140 130 120 110 100 90 80 70 60 50 40 30 20 10 0
f1 (ppm)

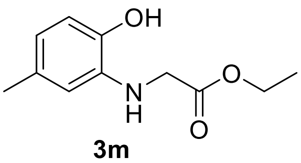
OH
N
H
O
O

3m

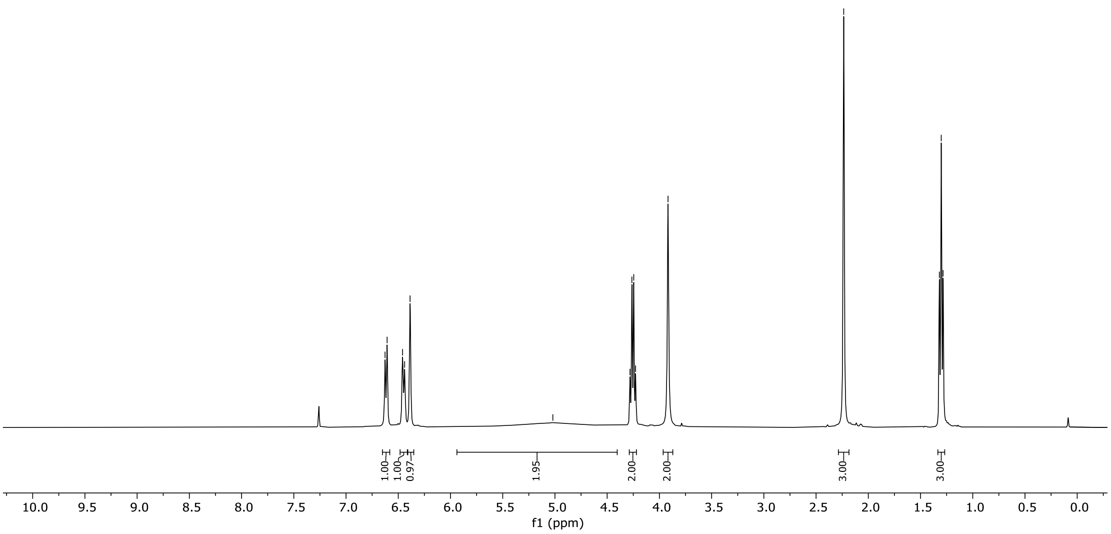
6.63
6.61
6.46
6.44
6.39
5.02
4.28
4.26
4.25
4.23
3.92
2.24
1.32
1.30
1.28
1.00
1.00
0.97
1.95
2.00
2.00
3.00
3.00
f1 (ppm)

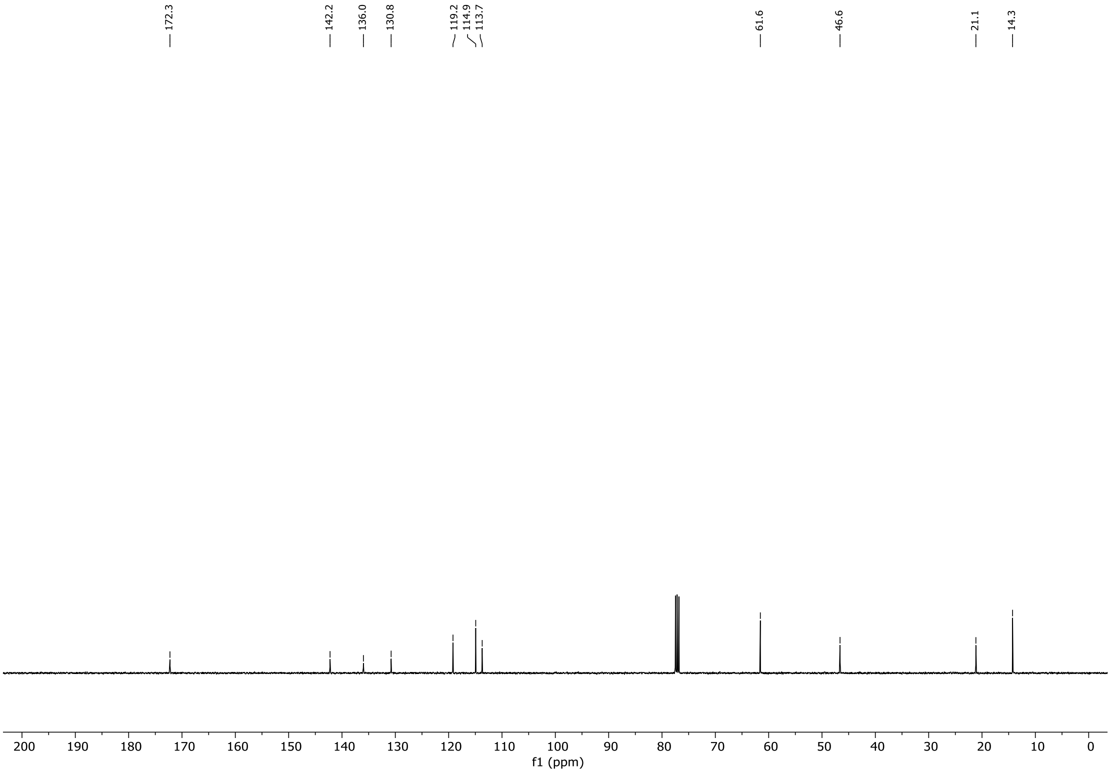
172.3
142.2
136.0
130.8
119.2
114.9
113.7
61.6
46.6
21.1
14.3
f1 (ppm)

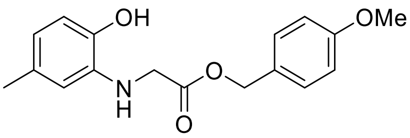
OH
OMe
N
H
O
O

3n

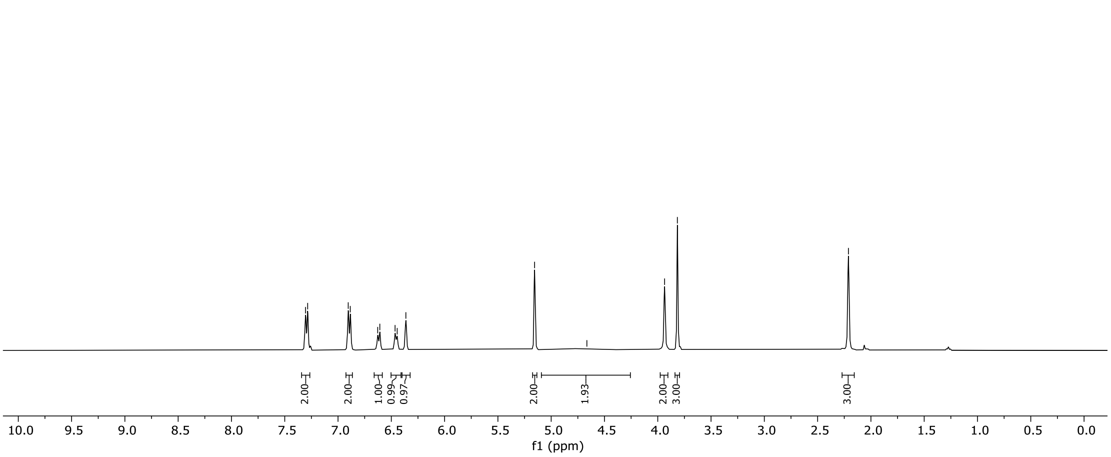
7.31
7.28
6.90
6.89
6.63
6.61
6.46
6.44
6.36
5.16
4.67
3.94
3.82
2.21
2.00
2.00
1.00
0.99
0.97
2.00
1.93
2.00
3.00
3.00
10.0 9.5 9.0 8.5 8.0 7.5 7.0 6.5 6.0 5.5 5.0 4.5 4.0 3.5 3.0 2.5 2.0 1.5 1.0 0.5 0.0
f1 (ppm)

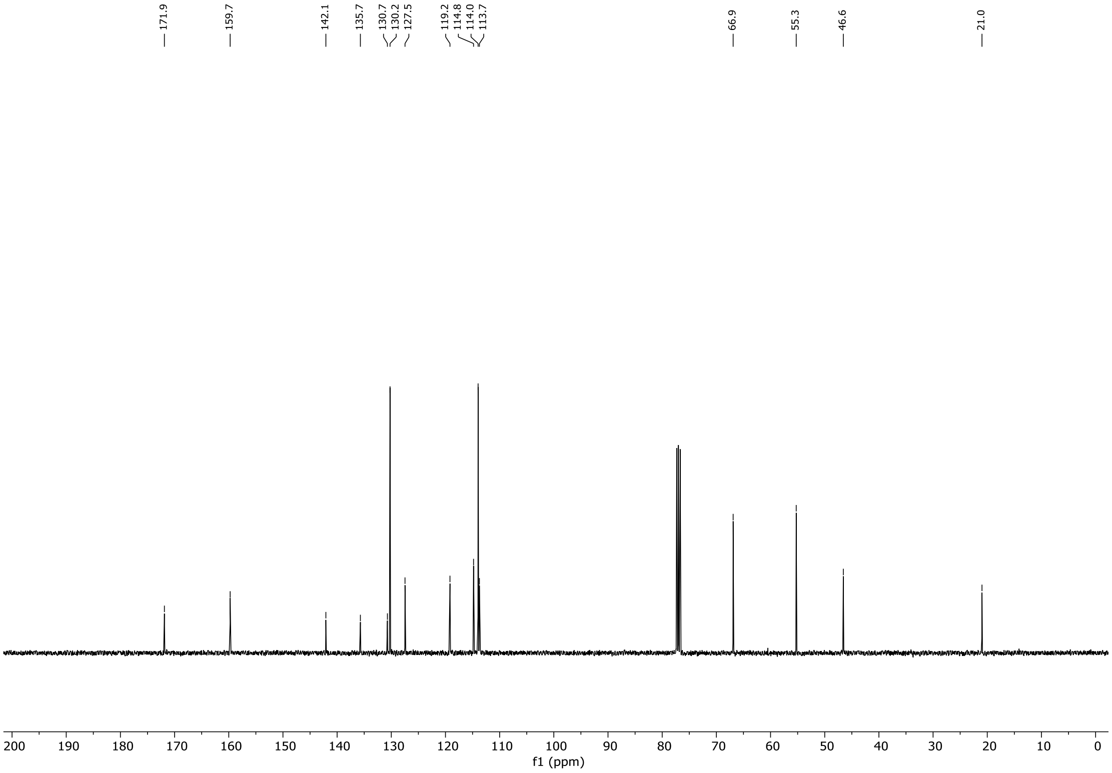
171.9
159.7
142.1
135.7
130.7
130.2
127.5
119.2
114.8
114.0
113.7
66.9
55.3
46.6
21.0
200 190 180 170 160 150 140 130 120 110 100 90 80 70 60 50 40 30 20 10 0
f1 (ppm)

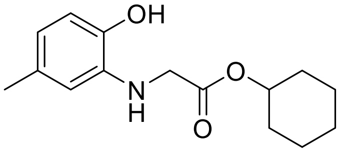
OH
N
H
O
O

3o

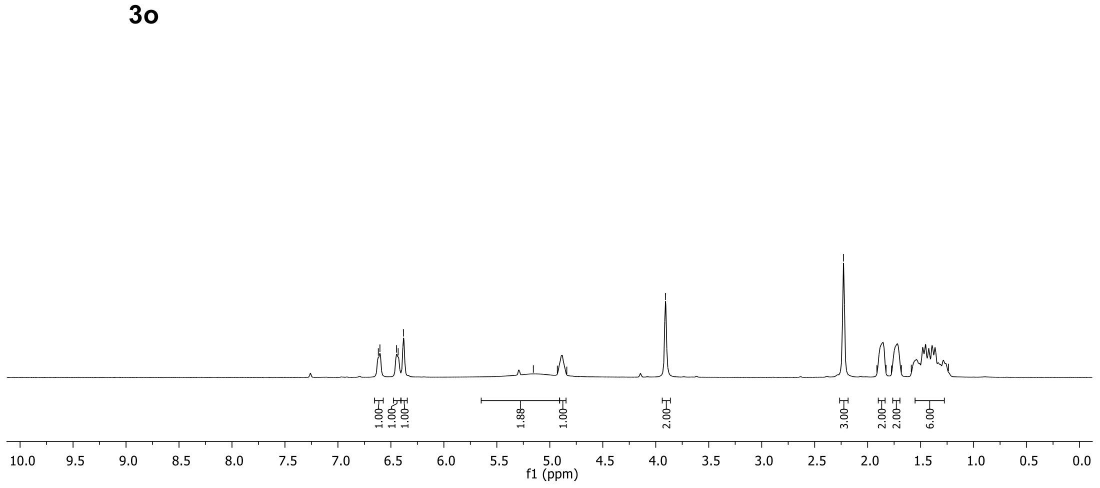
6.62
6.60
6.45
6.43
6.38
5.16
4.93
4.84
3.91
2.23
1.91
1.83
1.78
1.68
1.59
1.24
1.00
1.00
1.00
1.88
1.00
2.00
3.00
2.00
2.00
6.00
10.0 9.5 9.0 8.5 8.0 7.5 7.0 6.5 6.0 5.5 5.0 4.5 4.0 3.5 3.0 2.5 2.0 1.5 1.0 0.5 0.0
f1 (ppm)

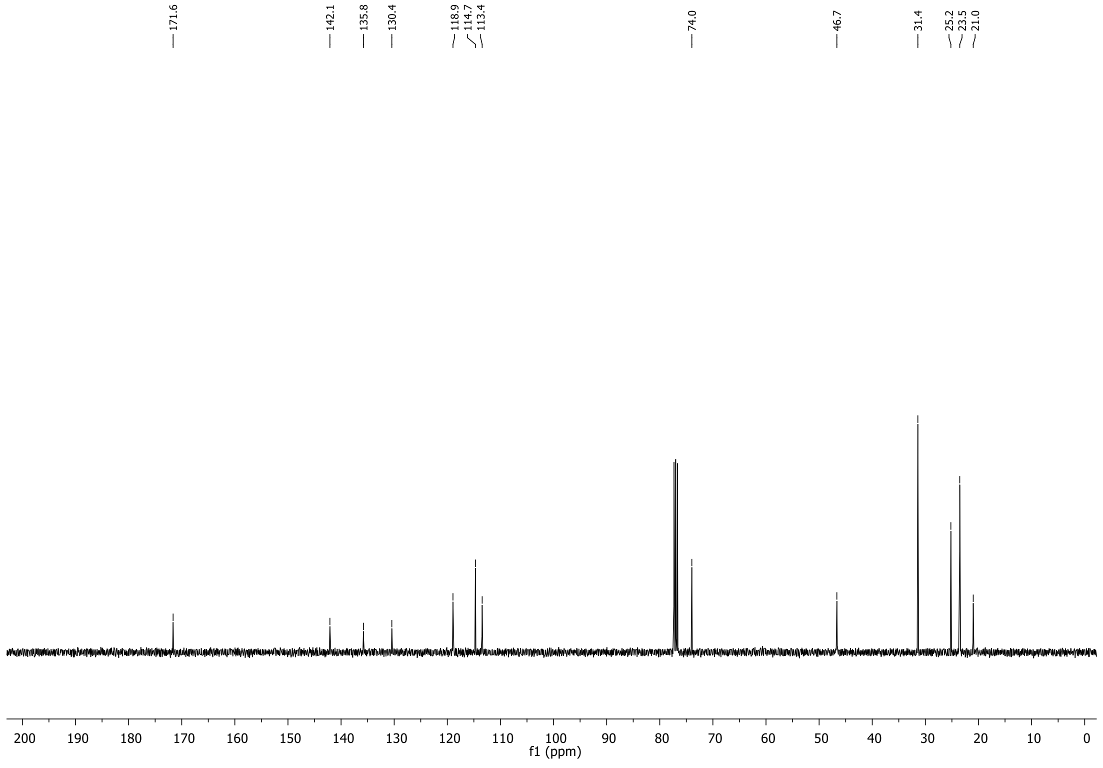
171.6
142.1
135.8
130.4
118.9
114.7
113.4
74.0
46.7
31.4
25.2
23.5
21.0
200 190 180 170 160 150 140 130 120 110 100 90 80 70 60 50 40 30 20 10 0
f1 (ppm)

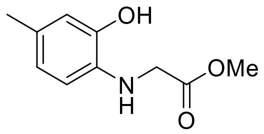
OH
N
H
OMe
O

3p

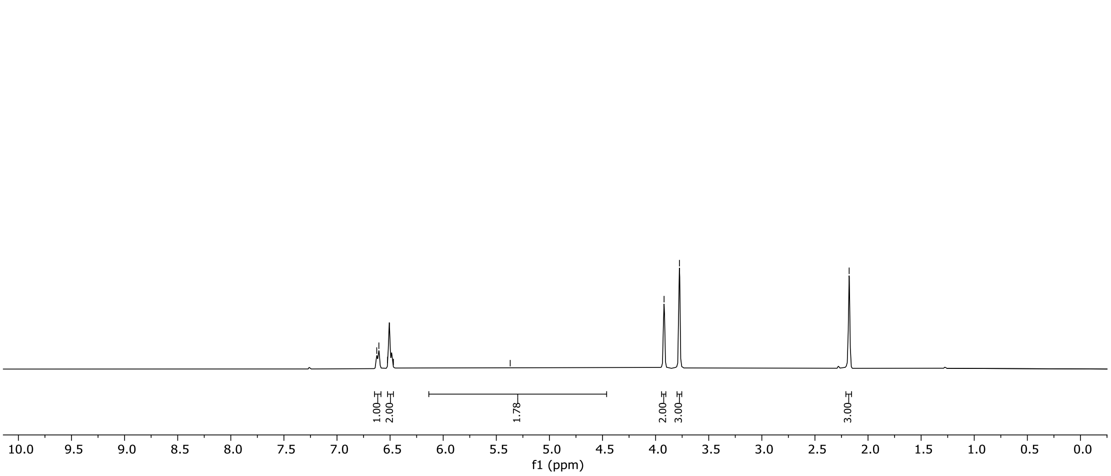
6.63
6.61
6.52
6.47
5.37
3.92
3.78
2.18
1.00
2.00
1.78
2.00
3.00
3.00
10.0 9.5 9.0 8.5 8.0 7.5 7.0 6.5 6.0 5.5 5.0 4.5 4.0 3.5 3.0 2.5 2.0 1.5 1.0 0.5 0.0
f1 (ppm)

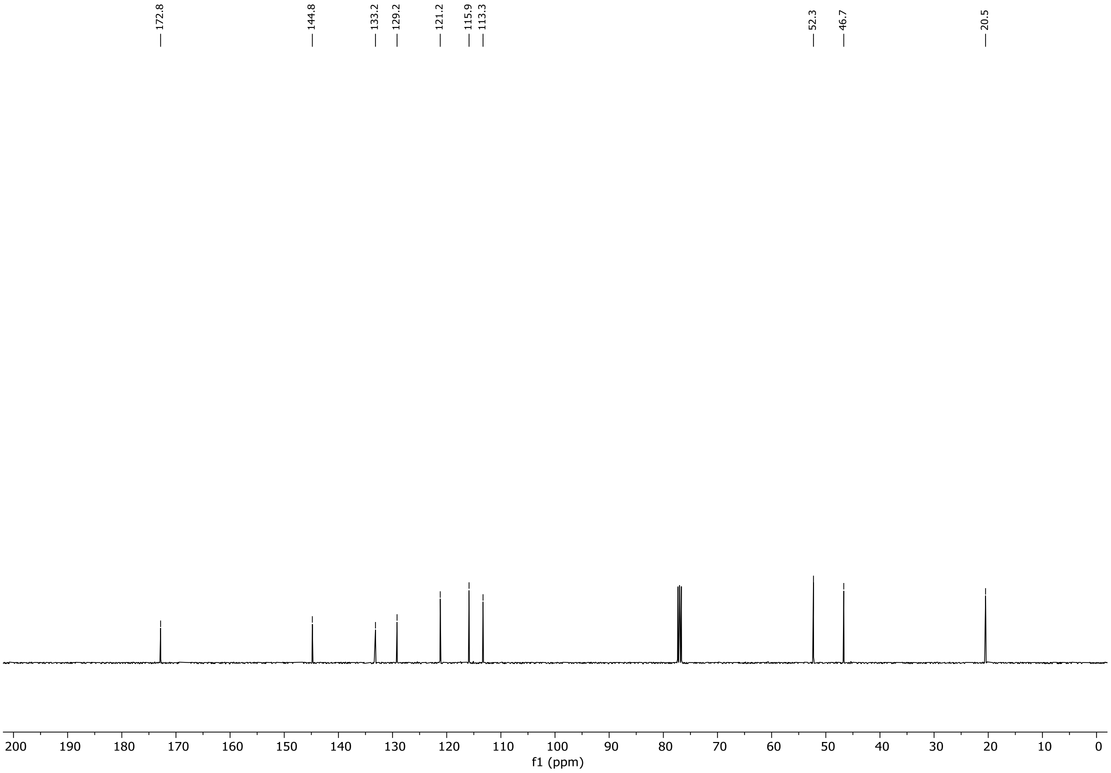
172.8
144.8
133.2
129.2
121.2
115.9
113.3
52.3
46.7
20.5
200 190 180 170 160 150 140 130 120 110 100 90 80 70 60 50 40 30 20 10 0
f1 (ppm)

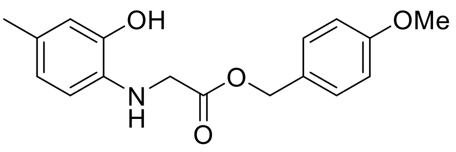
OH
OMe
N
H
O
O

3q

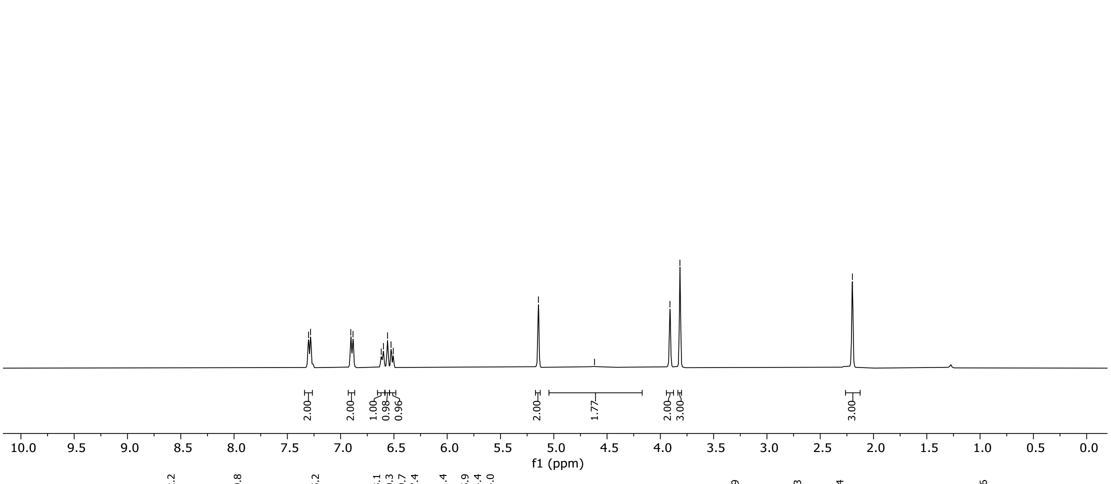
7.30
7.28
6.90
6.88
6.62
6.60
6.56
6.53
6.51
5.14
4.62
3.91
3.82
2.20
2.00
2.00
1.00
0.98
0.96
2.00
1.77
2.00
3.00
3.00
10.0
9.5
9.0
8.5
8.0
7.5
7.0
6.5
6.0
5.5
5.0
4.5
4.0
3.5
3.0
2.5
2.0
1.5
1.0
0.5
0.0
f1 (ppm)

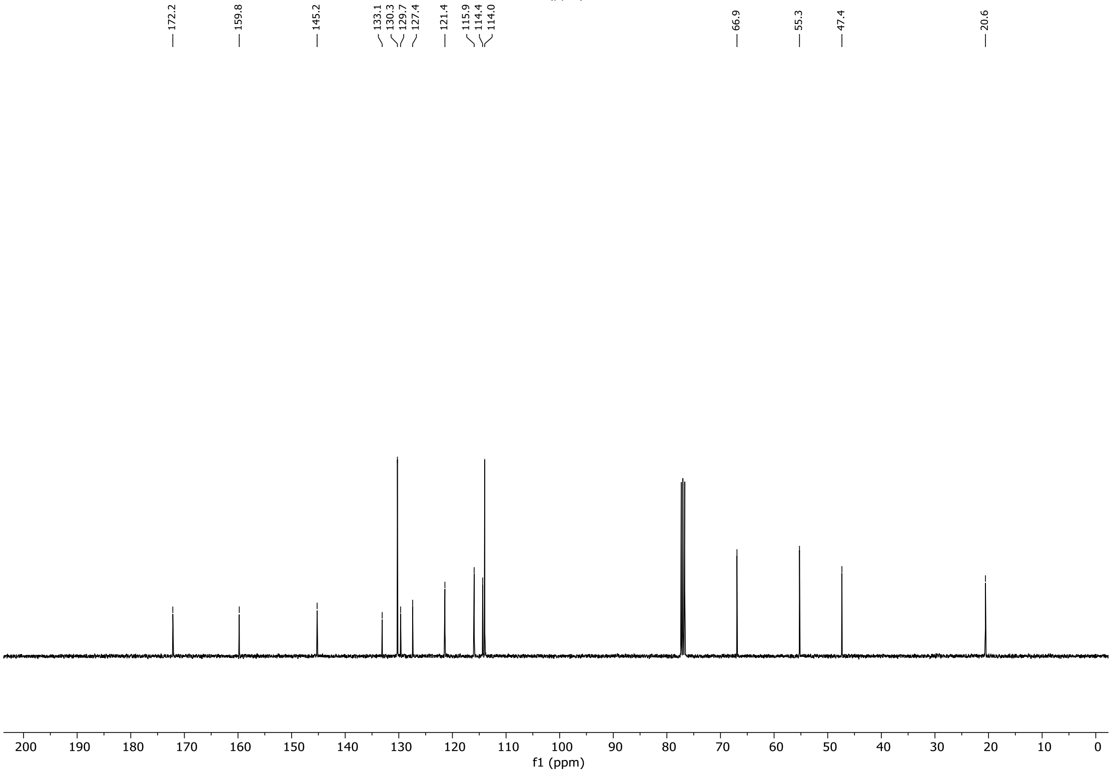
172.2
159.8
145.2
133.1
130.3
129.7
127.4
121.4
115.9
114.4
114.0
66.9
55.3
47.4
20.6
200
190
180
170
160
150
140
130
120
110
100
90
80
70
60
50
40
30
20
10
0
f1 (ppm)

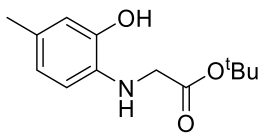
OH
N
H
O^{t}Bu
O

3r

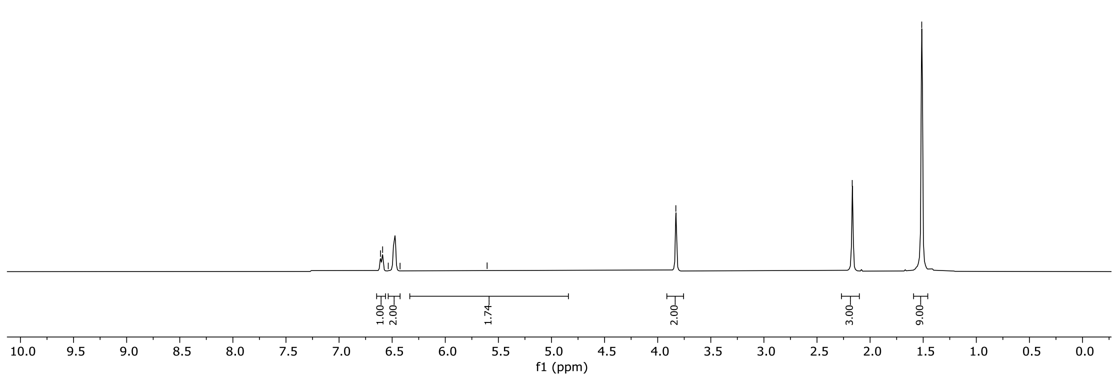
6.61
6.59
6.54
6.43
5.61
3.83
2.17
1.51
1.00
2.00
1.74
2.00
3.00
9.00
10.0 9.5 9.0 8.5 8.0 7.5 7.0 6.5 6.0 5.5 5.0 4.5 4.0 3.5 3.0 2.5 2.0 1.5 1.0 0.5 0.0
f1 (ppm)

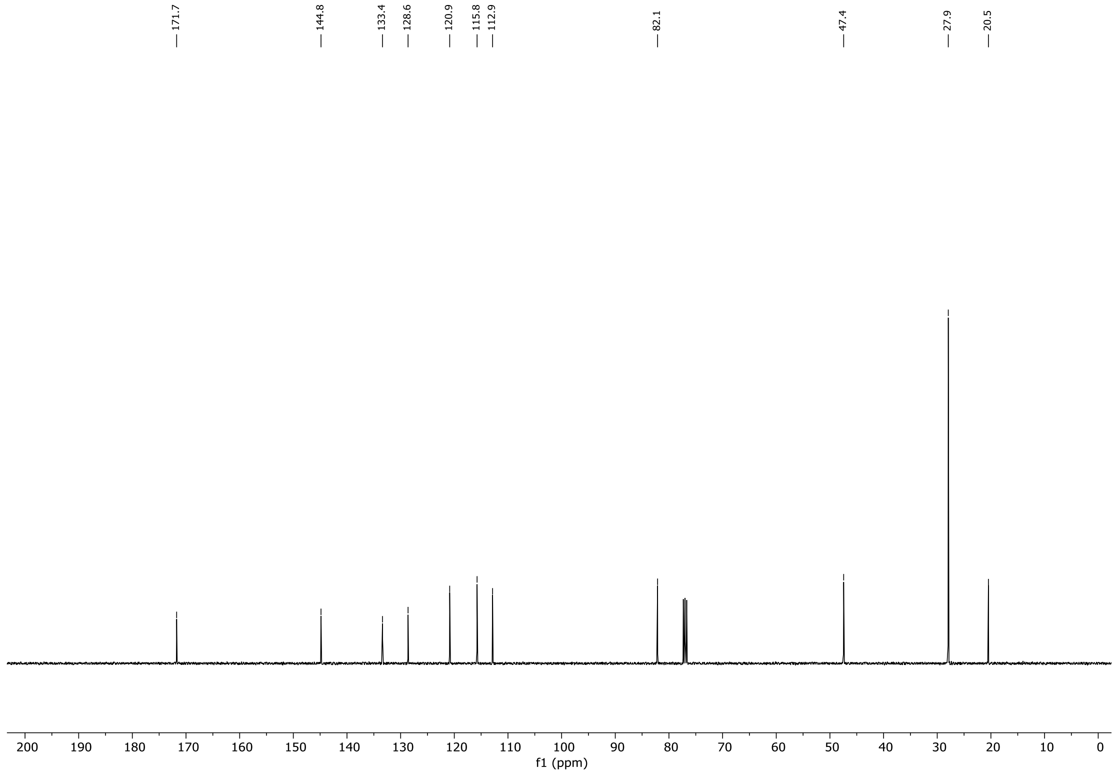
171.7
144.8
133.4
128.6
120.9
115.8
112.9
82.1
47.4
27.9
20.5
200 190 180 170 160 150 140 130 120 110 100 90 80 70 60 50 40 30 20 10 0
f1 (ppm)

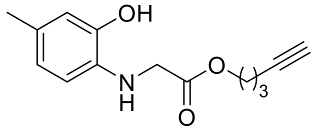
OH
N
H
O
O
3

3s

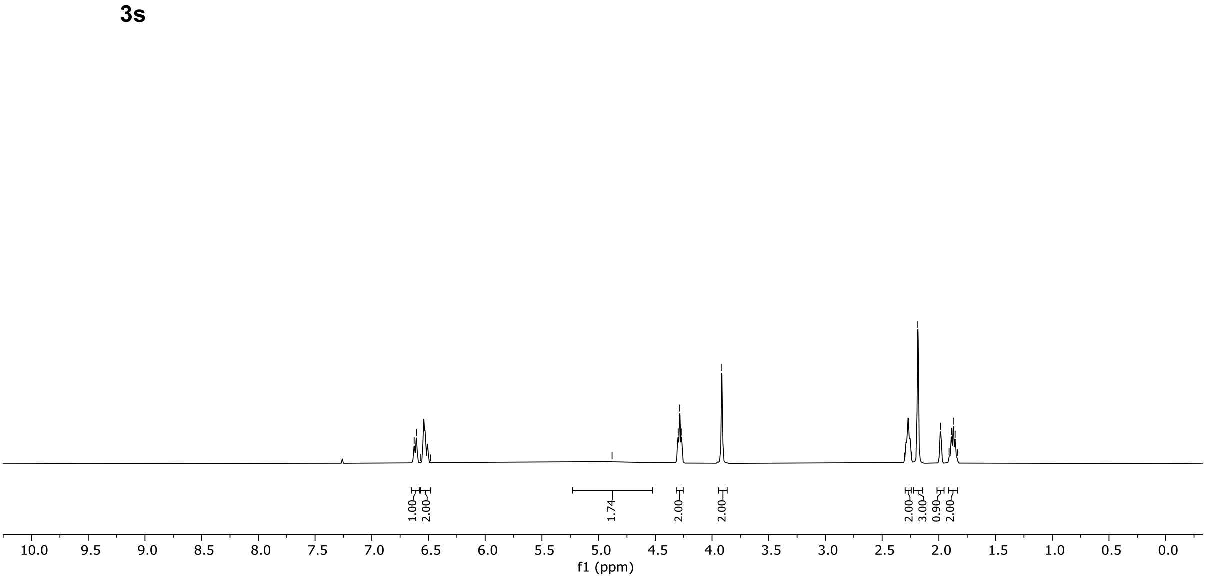
6.63
6.61
6.57
6.48
4.88
4.30
4.28
4.27
3.91
2.30
2.24
2.19
1.98
1.91
1.89
1.87
1.86
1.84
1.00
2.00
1.74
2.00
2.00
2.00
3.00
0.90
2.00
f1 (ppm)

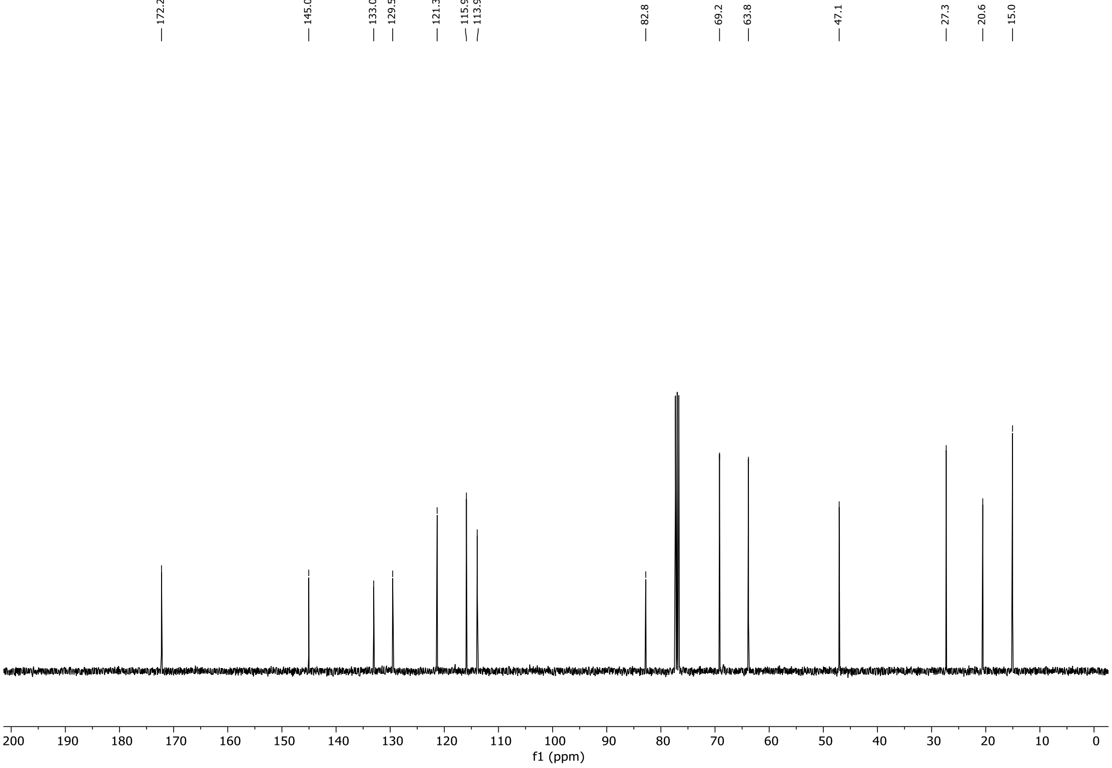
172.2
145.0
133.0
129.5
121.3
115.9
113.9
82.8
69.2
63.8
47.1
27.3
20.6
15.0
f1 (ppm)

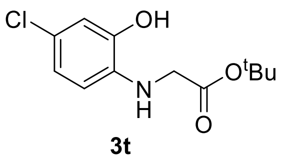
Cl
OH
N
H
O
O^{t}Bu

3t

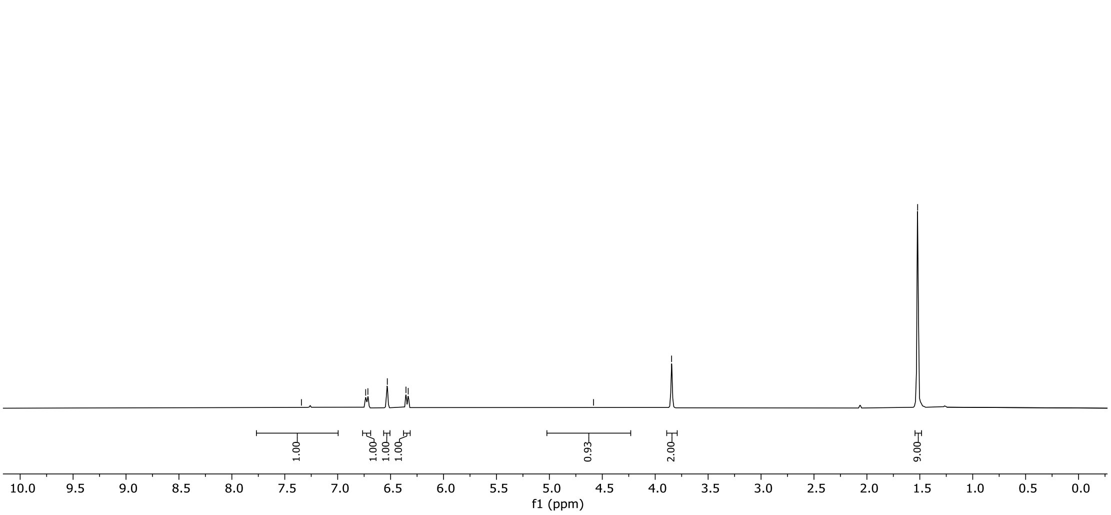
7.34
6.74
6.71
6.53
6.35
6.33
4.58
3.85
1.52
1.00
1.00
1.00
1.00
0.93
2.00
9.00
10.0 9.5 9.0 8.5 8.0 7.5 7.0 6.5 6.0 5.5 5.0 4.5 4.0 3.5 3.0 2.5 2.0 1.5 1.0 0.5 0.0
f1 (ppm)

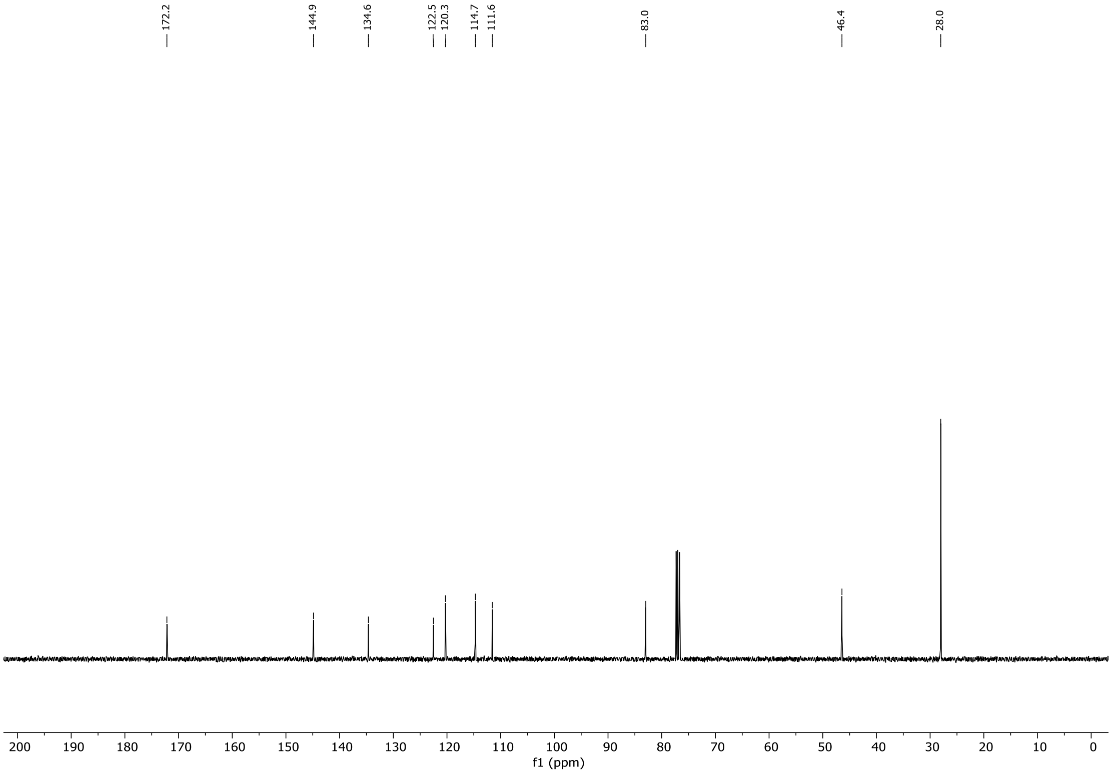
172.2
144.9
134.6
122.5
120.3
114.7
111.6
83.0
46.4
28.0
200 190 180 170 160 150 140 130 120 110 100 90 80 70 60 50 40 30 20 10 0
f1 (ppm)

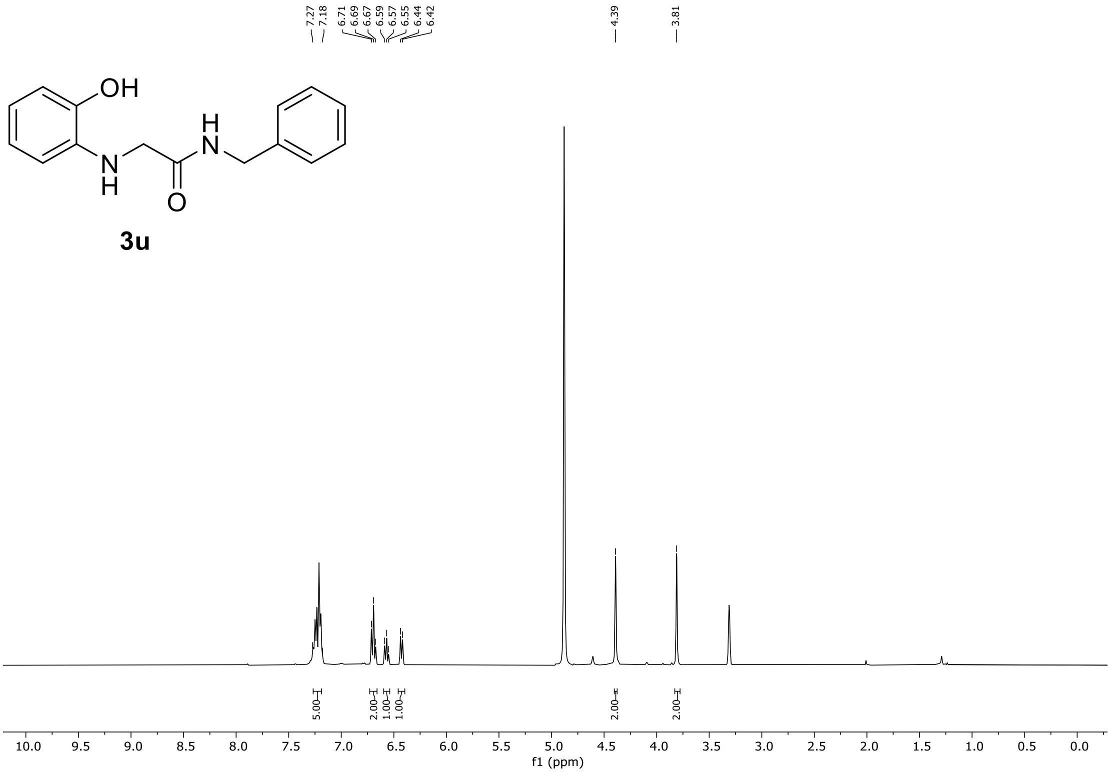

OH
H
N
N
H
O
3u
7.27
7.18
6.71
6.69
6.67
6.59
6.57
6.55
6.44
6.42
4.39
3.81
5.00
2.00
1.00
1.00
2.00
2.00
f1 (ppm)

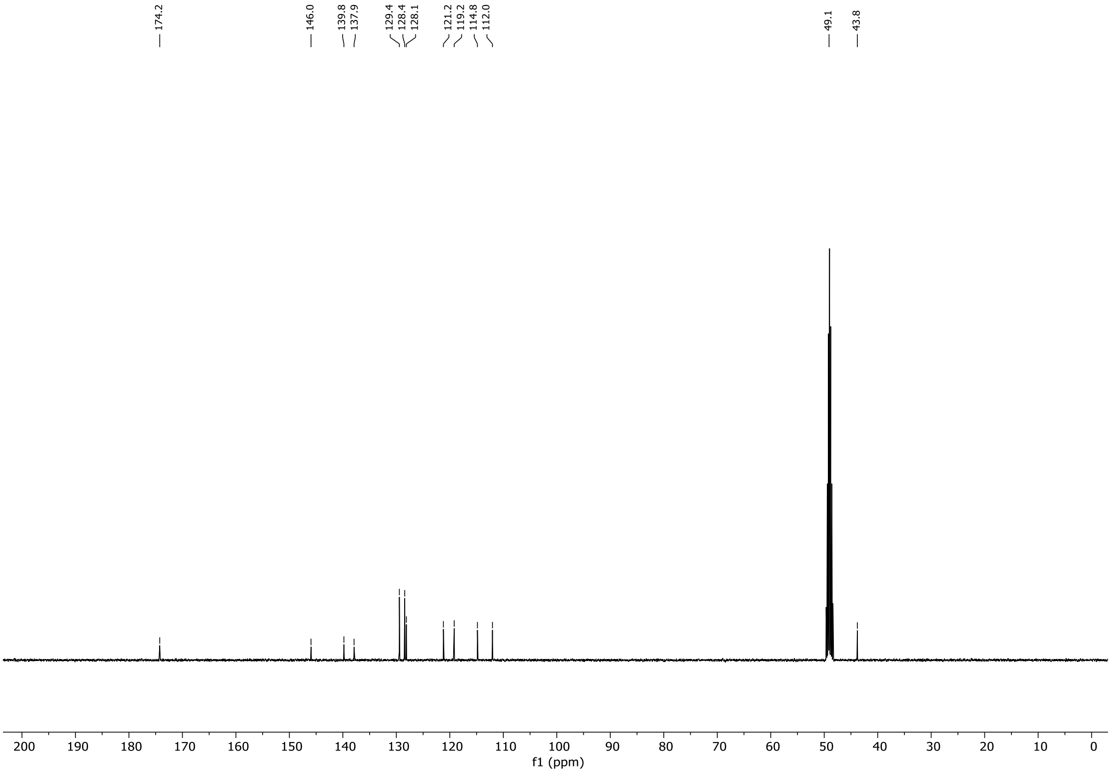

174.2
146.0
139.8
137.9
129.4
128.4
128.1
121.2
119.2
114.8
112.0
49.1
43.8
f1 (ppm)

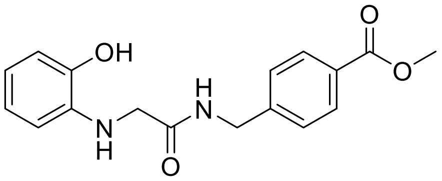
OH
H
N
N
H
O
O
O

3v

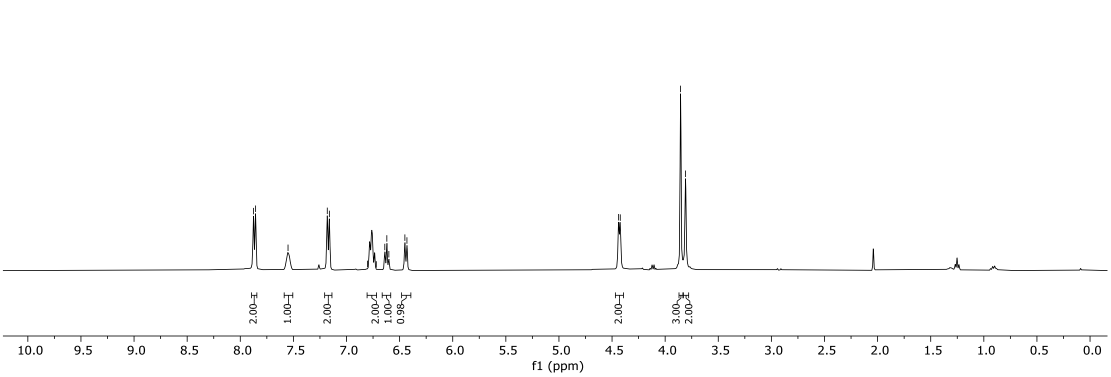
7.88
7.86
7.55
7.18
7.16
6.80
6.72
6.64
6.62
6.62
6.60
6.45
6.43
4.44
4.42
3.86
3.81
2.00
1.00
2.00
2.00
1.00
0.98
2.00
3.00
2.00
f1 (ppm)

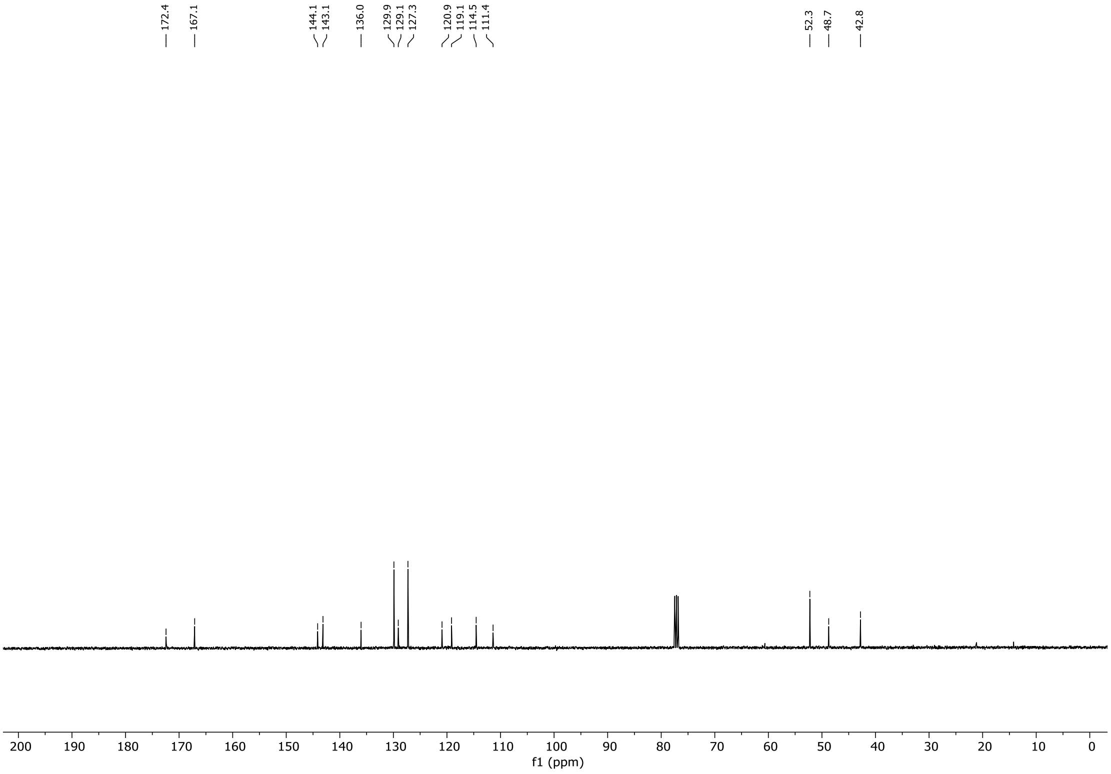
172.4
167.1
144.1
143.1
136.0
129.9
129.1
127.3
120.9
119.1
114.5
111.4
52.3
48.7
42.8
f1 (ppm)

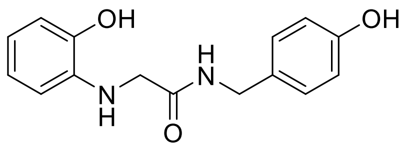
OH
H
N
N
H
O
OH

3u

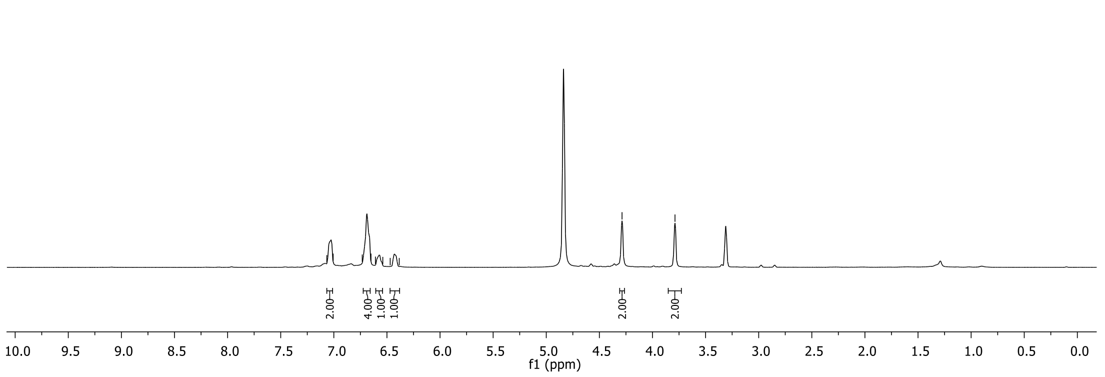
7.07
7.01
6.73
6.65
6.61
6.54
6.47
6.38
4.29
3.79
2.00
4.00
1.00
1.00
2.00
2.00
f1 (ppm)

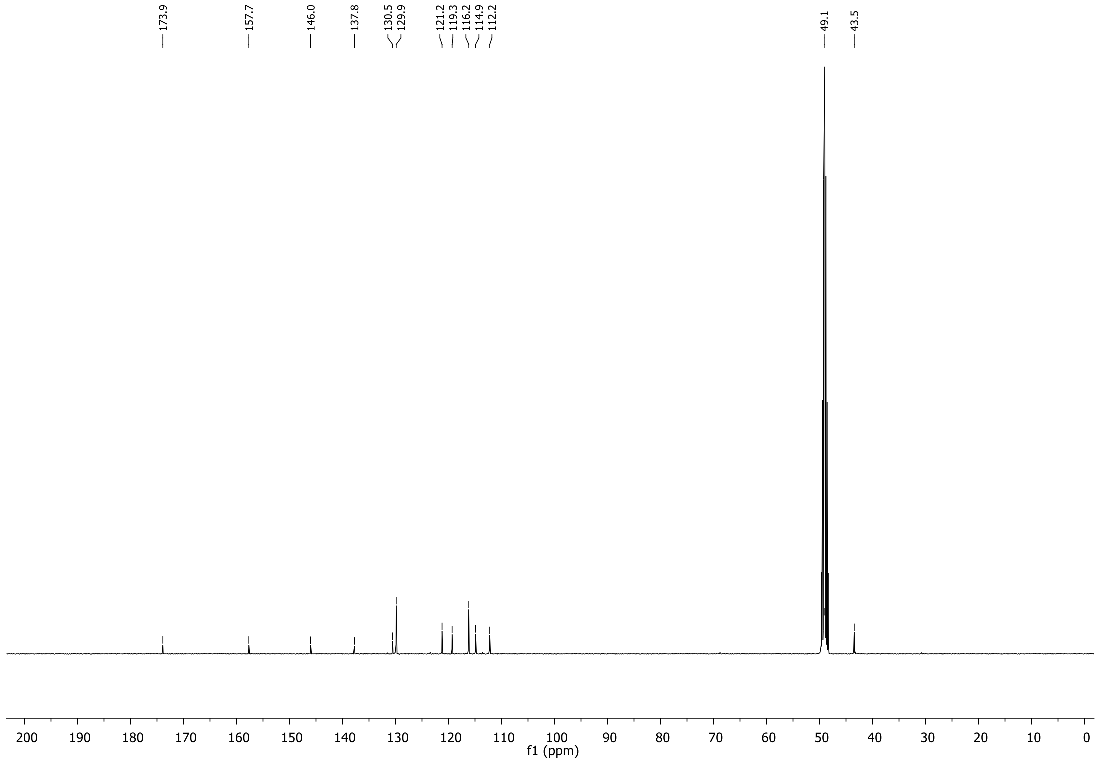
173.9
157.7
146.0
137.8
130.5
129.9
121.2
119.3
116.2
114.9
112.2
49.1
43.5
f1 (ppm)

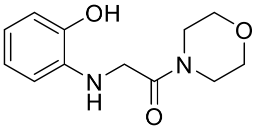
OH
N
H
O
N
O

3y

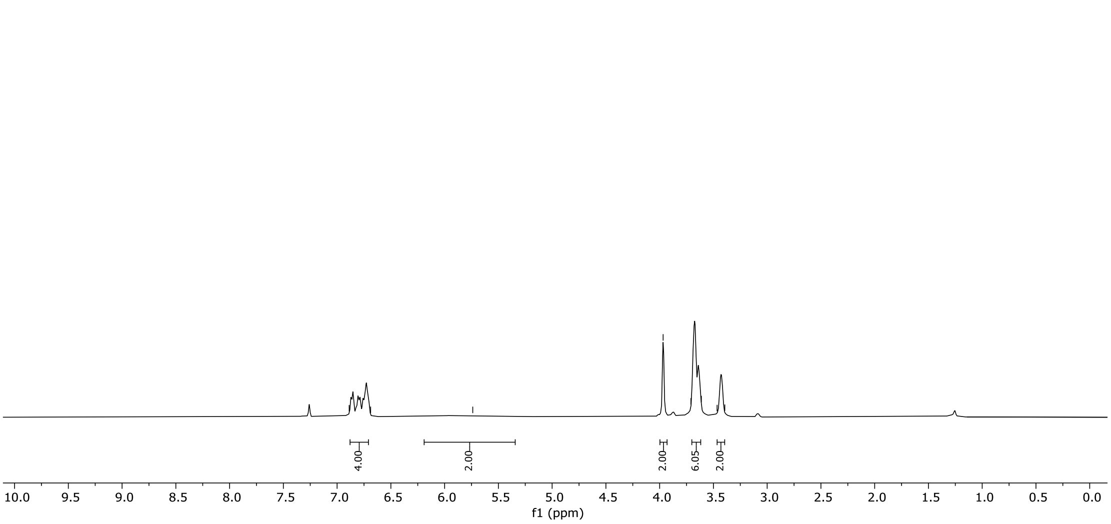
6.89
6.69
5.74
3.97
3.71
3.61
3.47
3.39
4.00
2.00
2.00
6.05
2.00
10.0 9.5 9.0 8.5 8.0 7.5 7.0 6.5 6.0 5.5 5.0 4.5 4.0 3.5 3.0 2.5 2.0 1.5 1.0 0.5 0.0
f1 (ppm)

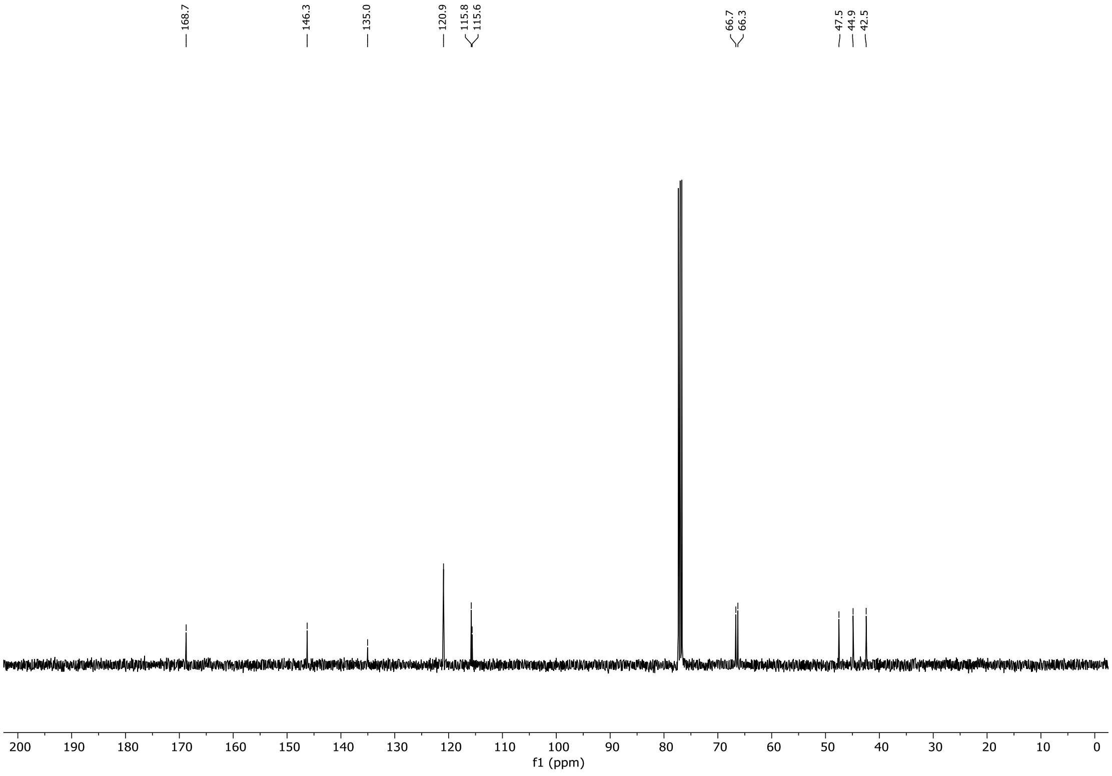
168.7
146.3
135.0
120.9
115.8
115.6
66.7
66.3
47.5
44.9
42.5
200 190 180 170 160 150 140 130 120 110 100 90 80 70 60 50 40 30 20 10 0
f1 (ppm)

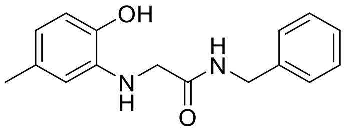
OH
H
N
N
H
O

3z

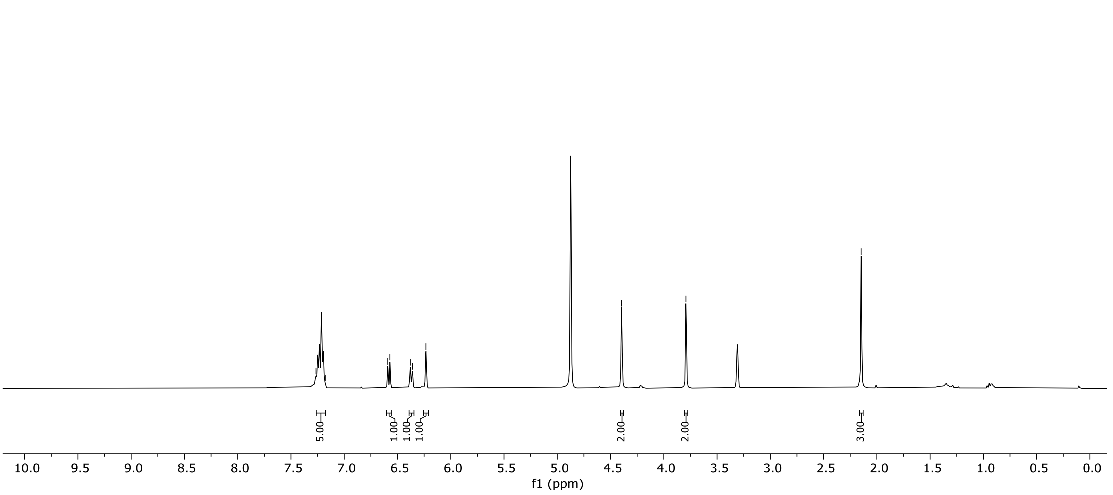
7.26
7.18
6.59
6.57
6.38
6.36
6.23
4.40
3.79
2.15
5.00
1.00
1.00
1.00
2.00
2.00
3.00
10.0 9.5 9.0 8.5 8.0 7.5 7.0 6.5 6.0 5.5 5.0 4.5 4.0 3.5 3.0 2.5 2.0 1.5 1.0 0.5 0.0
f1 (ppm)

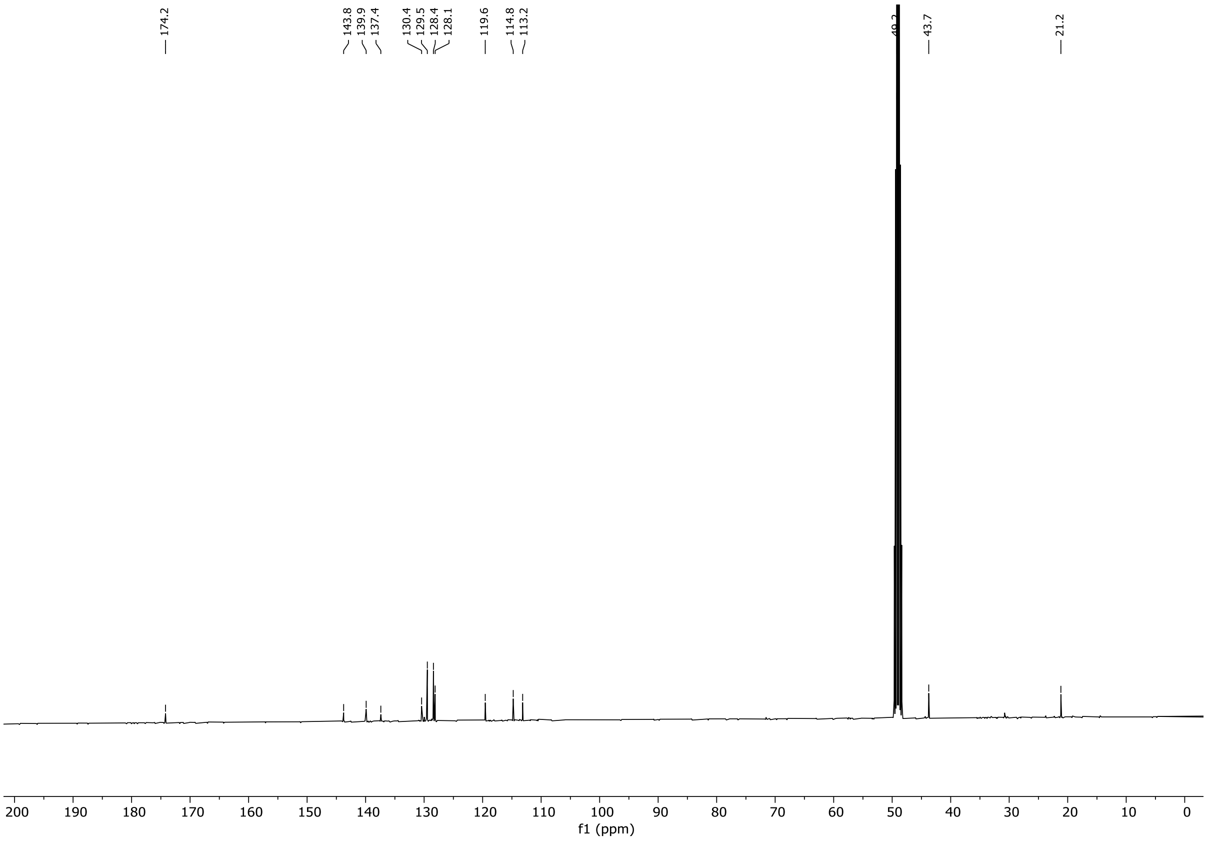
174.2
143.8
139.9
137.4
130.4
129.5
128.4
128.1
119.6
114.8
113.2
43.7
21.2
200 190 180 170 160 150 140 130 120 110 100 90 80 70 60 50 40 30 20 10 0
f1 (ppm)

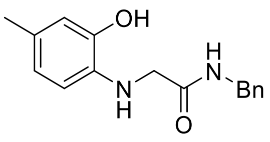
OH
H
N
Bn
N
H
O

3aa

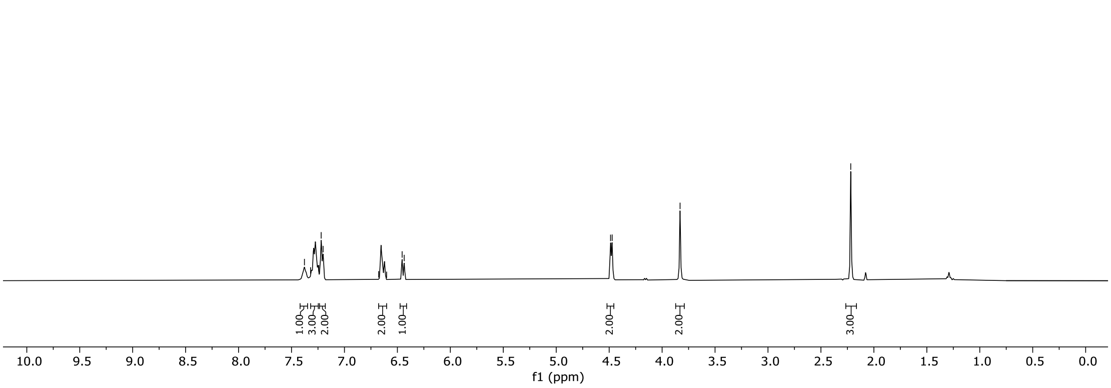
7.38
7.32
7.24
7.22
7.20
6.68
6.60
6.46
6.44
4.49
4.47
3.83
2.22
1.00
3.00
2.00
2.00
1.00
2.00
2.00
3.00
f1 (ppm)

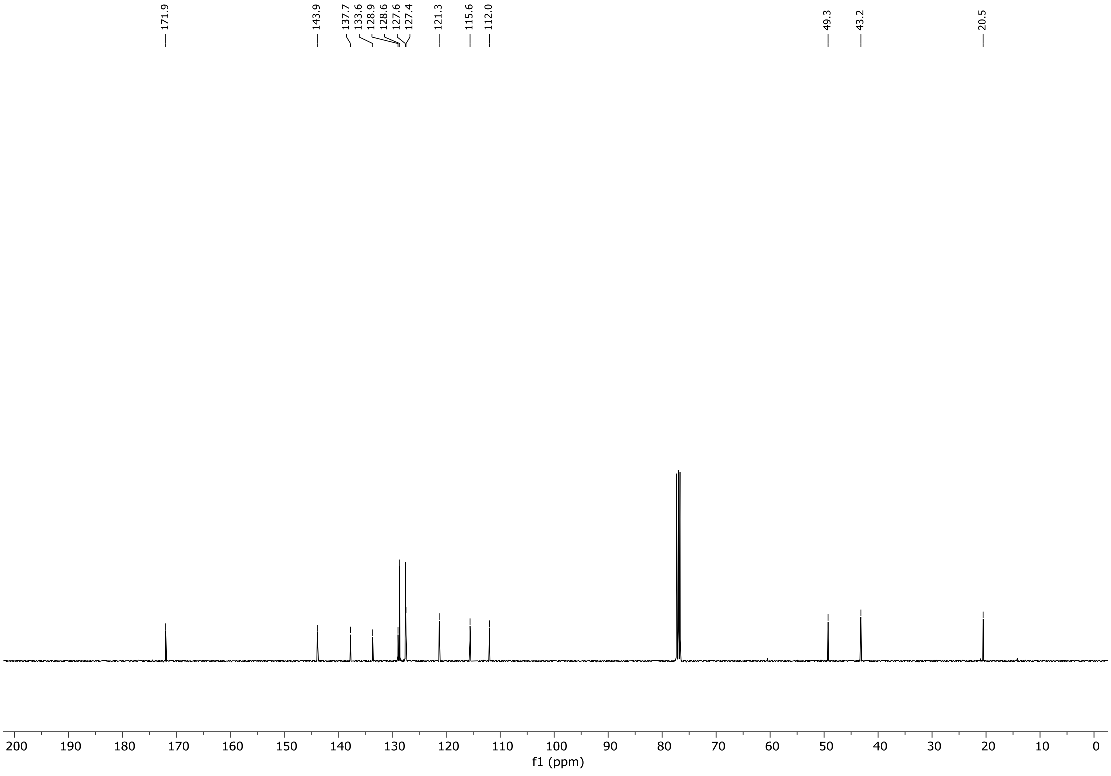
171.9
143.9
137.7
133.6
128.9
128.6
127.6
127.4
121.3
115.6
112.0
49.3
43.2
20.5
f1 (ppm)

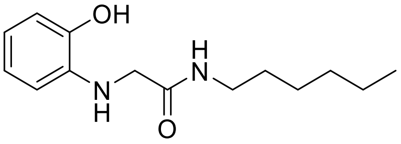
OH
N
H
H
N
O

3ab

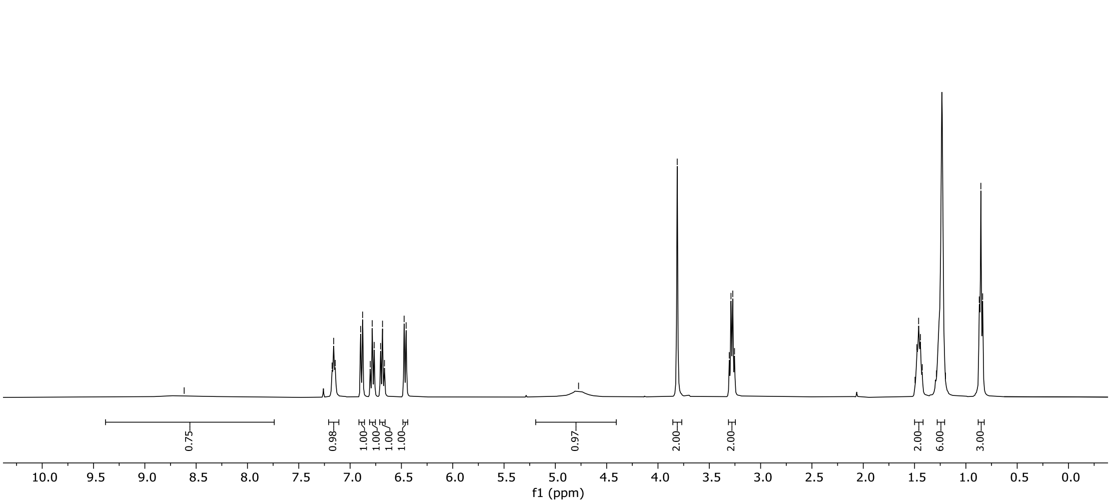
8.62
7.18 7.16 7.14 6.90 6.88 6.80 6.78 6.77 6.70 6.68 6.67 6.47 6.45
4.77
3.81
3.31 3.29 3.27 3.25
1.50 1.48 1.46 1.44 1.43 1.29 1.20 0.87 0.85 0.84
0.75 0.98 1.00 1.00 1.00 1.00 0.97 2.00 2.00 2.00 6.00 3.00
f1 (ppm)

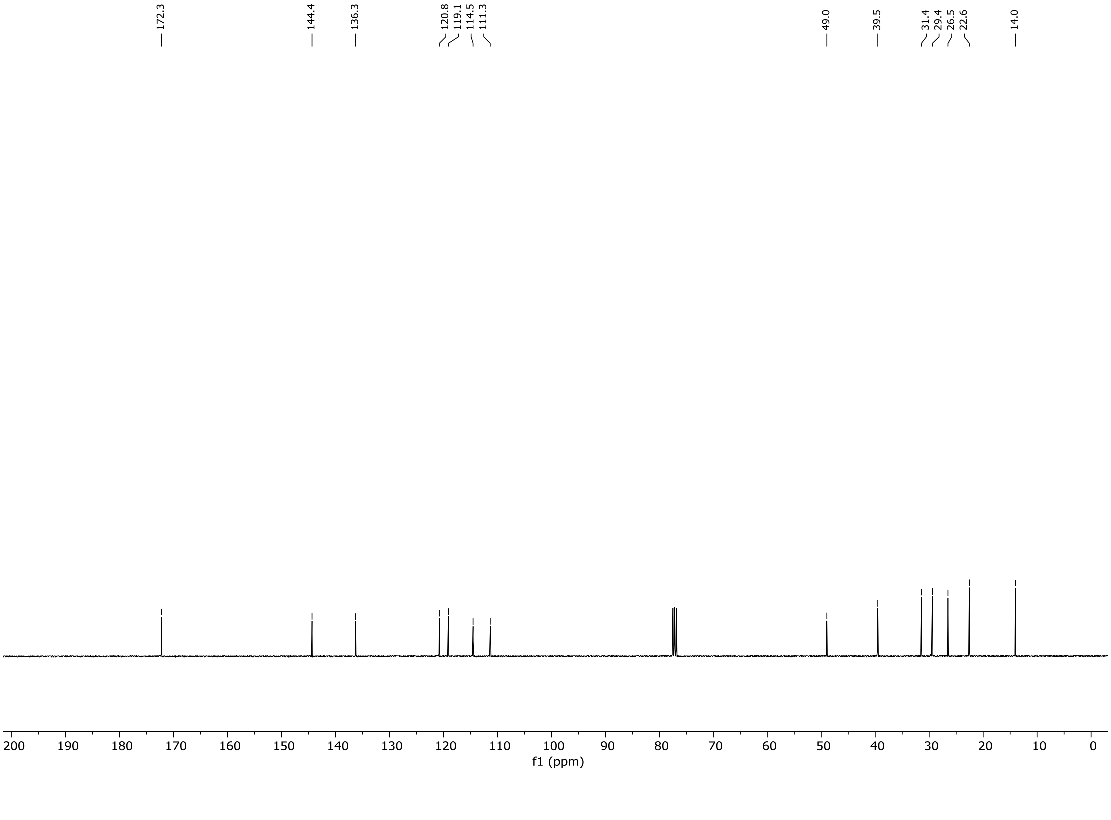
172.3
144.4
136.3
120.8 119.1 114.5 111.3
49.0
39.5
31.4 29.4 26.5 22.6
14.0
f1 (ppm)

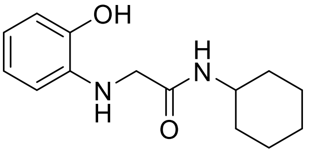
OH
H
N
N
H
O

3ac

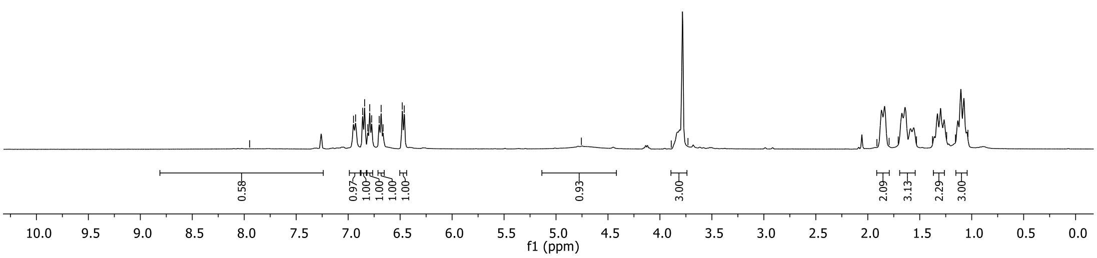
7.95
6.95
6.93
6.86
6.84
6.81
6.79
6.77
6.70
6.68
6.66
6.48
6.46
4.76
3.89
3.73
1.91
1.79
1.71
1.53
1.38
1.24
1.16
1.04
0.58
0.97
1.00
1.00
1.00
1.00
0.93
3.00
2.09
3.13
2.29
3.00
10.0 9.5 9.0 8.5 8.0 7.5 7.0 6.5 6.0 5.5 5.0 4.5 4.0 3.5 3.0 2.5 2.0 1.5 1.0 0.5 0.0
f1 (ppm)

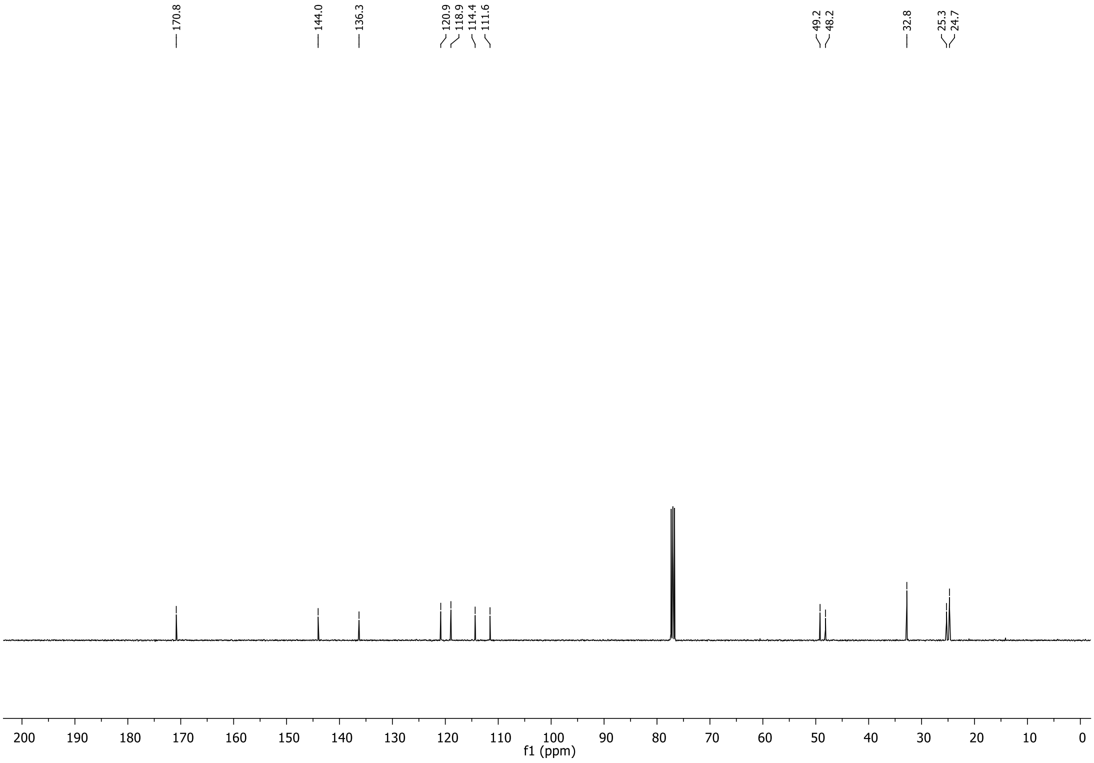
170.8
144.0
136.3
120.9
118.9
114.4
111.6
49.2
48.2
32.8
25.3
24.7
200 190 180 170 160 150 140 130 120 110 100 90 80 70 60 50 40 30 20 10 0
f1 (ppm)

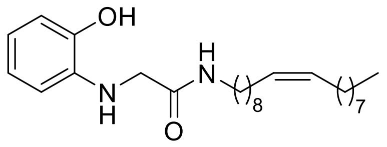
OH
N
H
H
N
O
8
7

3ad

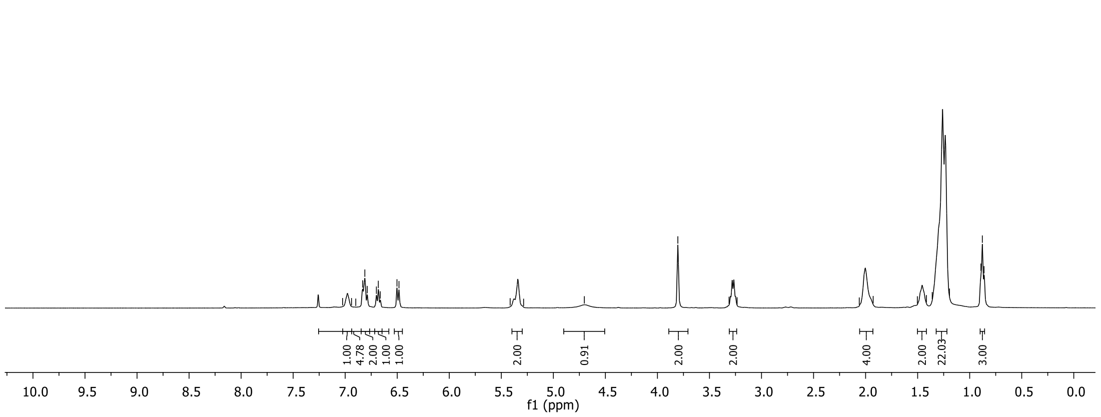
7.02
6.94
6.90
6.83
6.81
6.79
6.70
6.68
6.66
6.50
6.48
5.42
5.29
4.70
3.80
3.31
3.23
2.06
1.93
1.50
1.42
1.36
1.19
0.89
0.88
0.86
1.00
4.78
2.00
1.00
1.00
2.00
0.91
2.00
2.00
4.00
2.00
22.03
3.00
10.0 9.5 9.0 8.5 8.0 7.5 7.0 6.5 6.0 5.5 5.0 4.5 4.0 3.5 3.0 2.5 2.0 1.5 1.0 0.5 0.0
f1 (ppm)

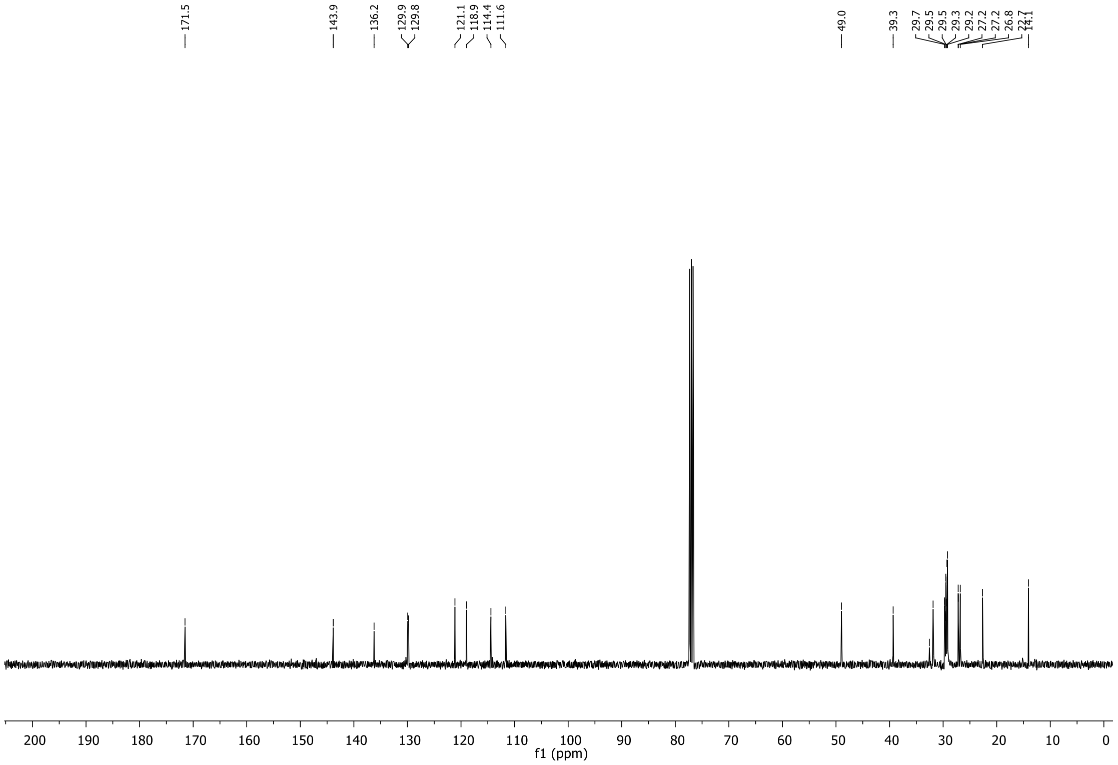
171.5
143.9
136.2
129.9
129.8
121.1
118.9
114.4
111.6
49.0
39.3
29.7
29.5
29.5
29.3
29.2
27.2
27.2
26.8
22.7
14.1
200 190 180 170 160 150 140 130 120 110 100 90 80 70 60 50 40 30 20 10 0
f1 (ppm)

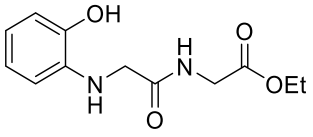
OH
H
N
O
N
H
O
OEt

3ae

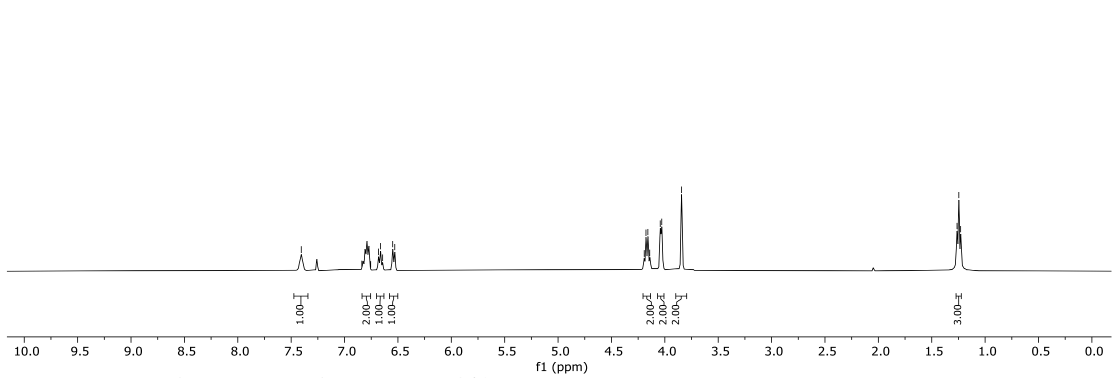
7.41
6.84
6.76
6.68
6.66
6.64
6.55
6.53
4.19
4.18
4.16
4.14
4.04
4.03
3.84
1.26
1.25
1.23
1.00
2.00
1.00
1.00
2.00
2.00
2.00
3.00
10.0 9.5 9.0 8.5 8.0 7.5 7.0 6.5 6.0 5.5 5.0 4.5 4.0 3.5 3.0 2.5 2.0 1.5 1.0 0.5 0.0
f1 (ppm)

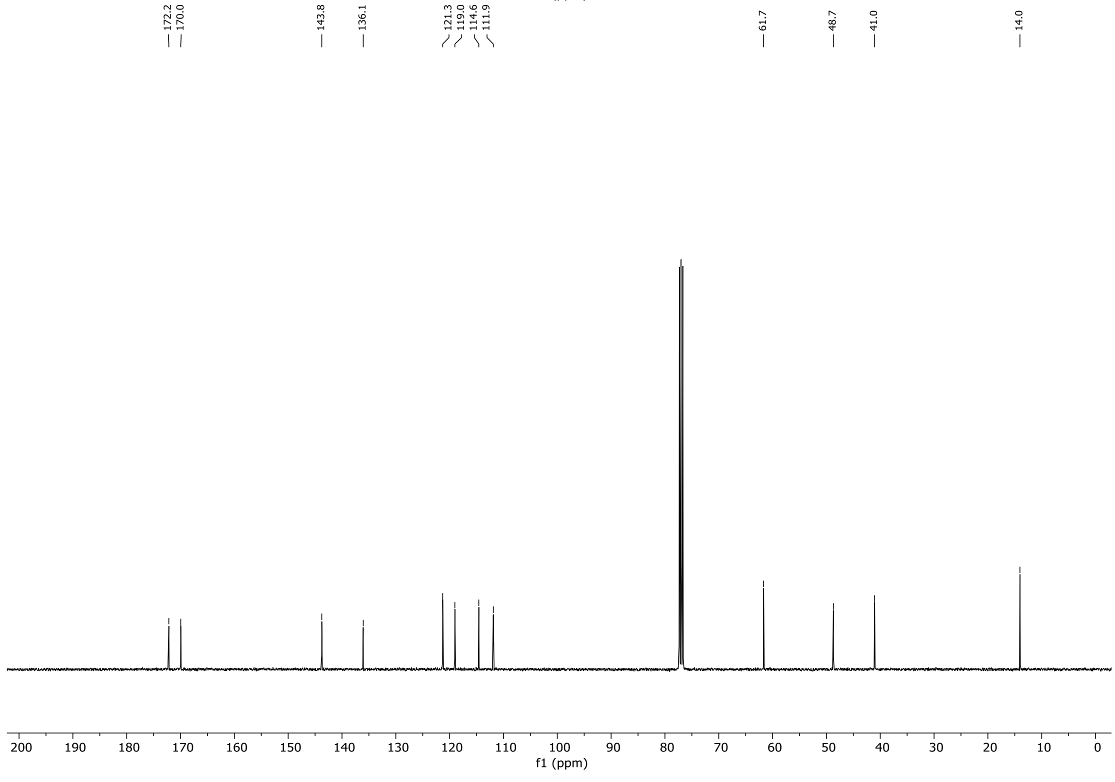
172.2
170.0
143.8
136.1
121.3
119.0
114.6
111.9
61.7
48.7
41.0
14.0
200 190 180 170 160 150 140 130 120 110 100 90 80 70 60 50 40 30 20 10 0
f1 (ppm)

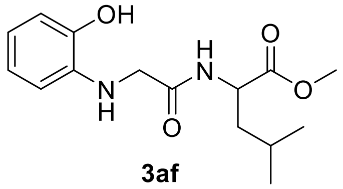
OH
H
N
O
O
N
H
O

3af

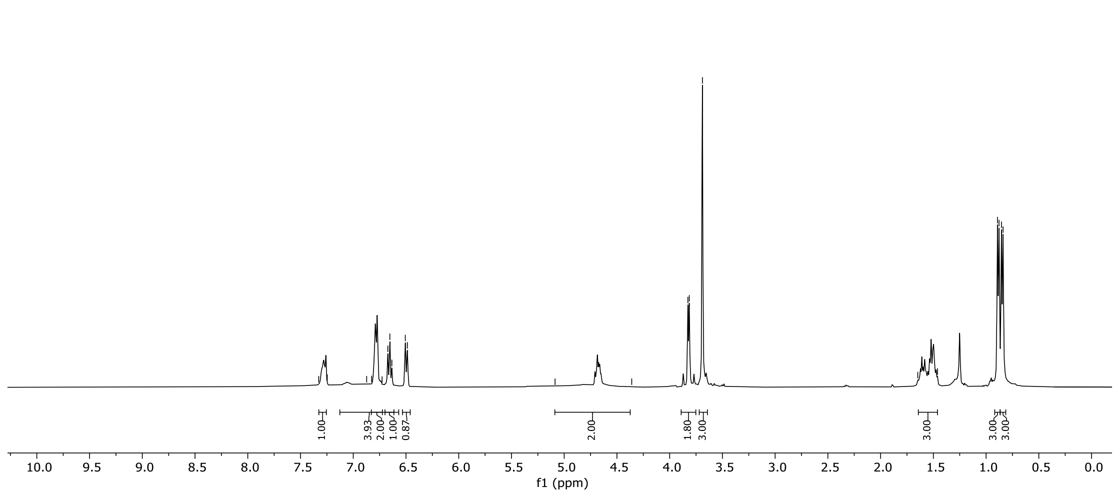
7.33
7.25
6.87
6.83
6.73
6.67
6.65
6.63
6.51
6.49
5.09
4.36
3.83
3.81
3.69
1.65
1.46
0.89
0.88
0.85
0.84
1.00
3.93
2.00
1.00
0.87
2.00
1.80
3.00
3.00
3.00
3.00
10.0 9.5 9.0 8.5 8.0 7.5 7.0 6.5 6.0 5.5 5.0 4.5 4.0 3.5 3.0 2.5 2.0 1.5 1.0 0.5 0.0
f1 (ppm)

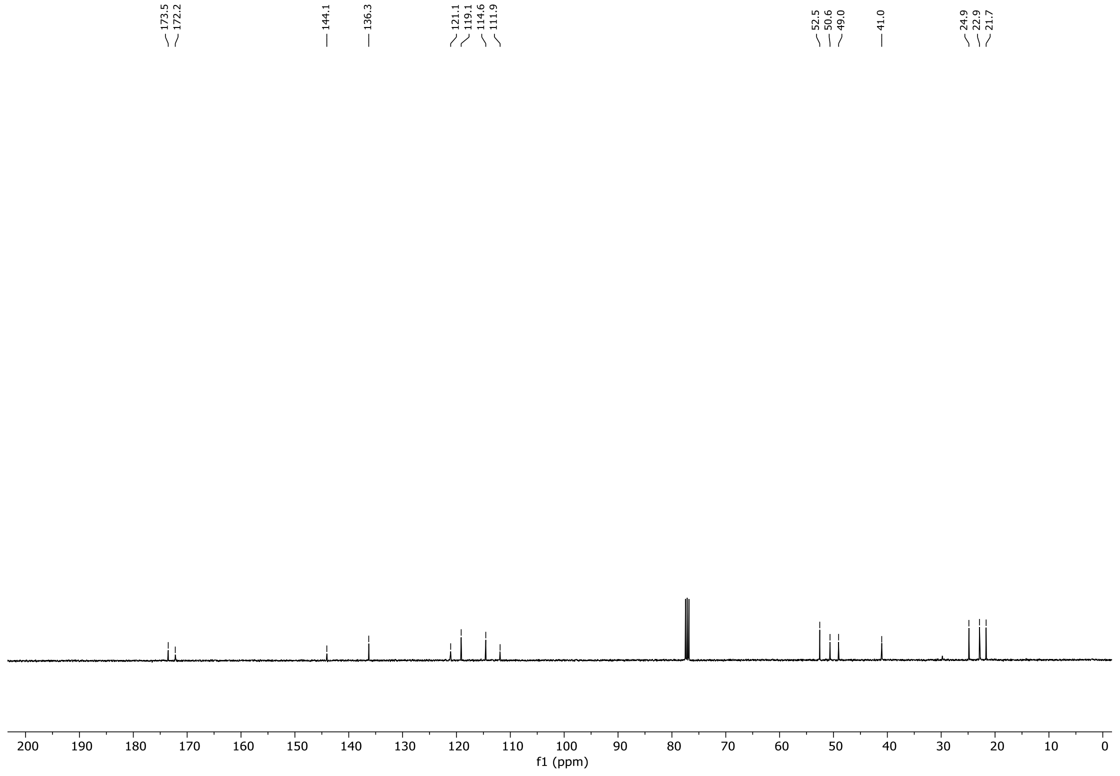
173.5
172.2
144.1
136.3
121.1
119.1
114.6
111.9
52.5
50.6
49.0
41.0
24.9
22.9
21.7
200 190 180 170 160 150 140 130 120 110 100 90 80 70 60 50 40 30 20 10 0
f1 (ppm)

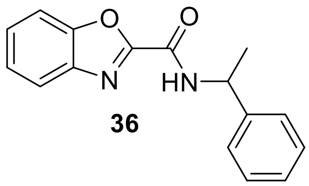

36

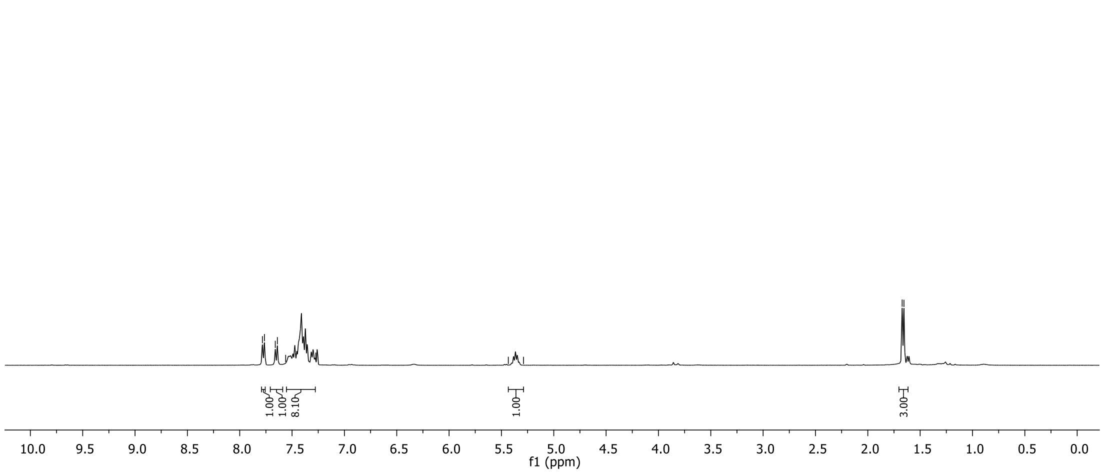
7.78
7.76
7.66
7.64
7.56
7.27
5.43
5.29
1.67
1.65
1.00
1.00
8.10
1.00
3.00
10.0 9.5 9.0 8.5 8.0 7.5 7.0 6.5 6.0 5.5 5.0 4.5 4.0 3.5 3.0 2.5 2.0 1.5 1.0 0.5 0.0
f1 (ppm)

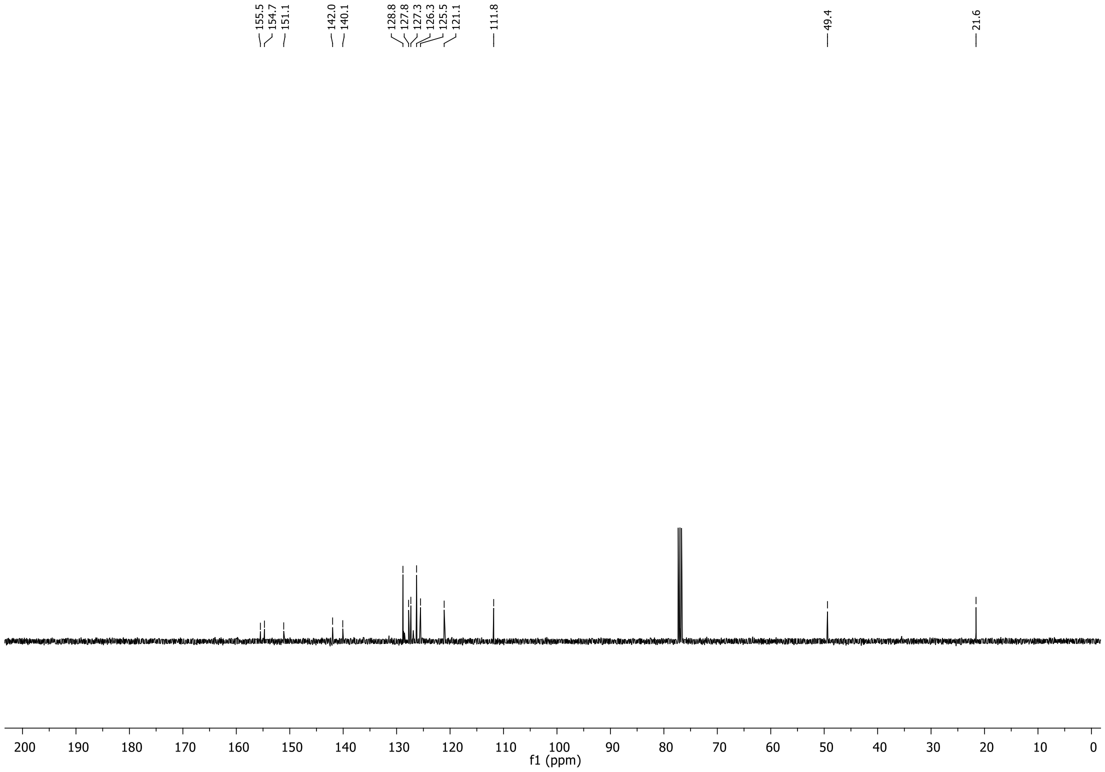
155.5
154.7
151.1
142.0
140.1
128.8
127.8
127.3
126.3
125.5
121.1
111.8
49.4
21.6
200 190 180 170 160 150 140 130 120 110 100 90 80 70 60 50 40 30 20 10 0
f1 (ppm)

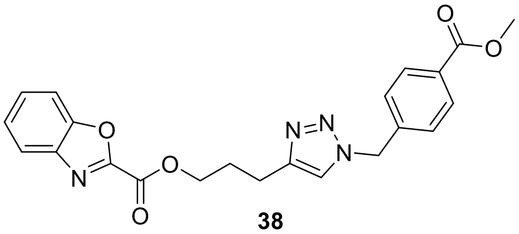
O
O
N
O
O
N=N
N
O

38

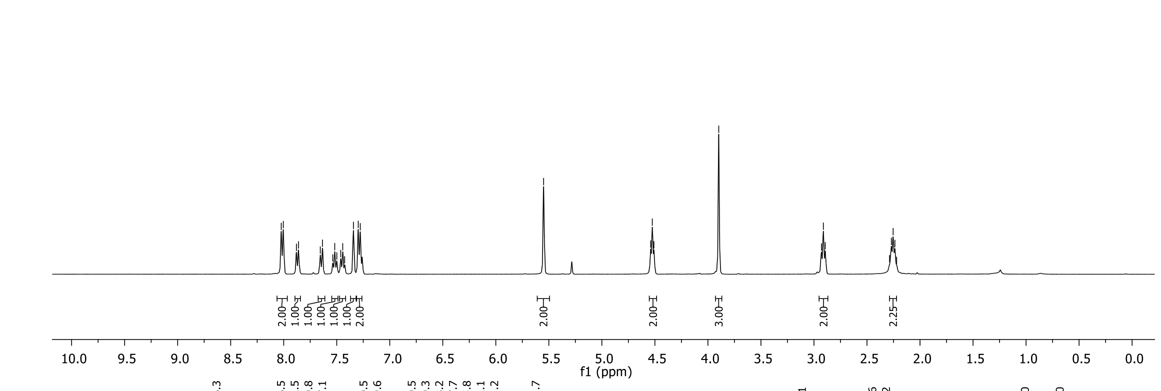
8.02
8.00
7.88
7.86
7.66
7.63
7.54
7.52
7.50
7.46
7.44
7.43
7.34
7.30
7.28
5.55
4.54
4.52
4.51
3.90
2.93
2.91
2.89
2.29
2.27
2.25
2.24
2.22
2.00
1.00
1.00
1.00
1.00
1.00
2.00
2.00
2.00
3.00
2.00
2.25
f1 (ppm)

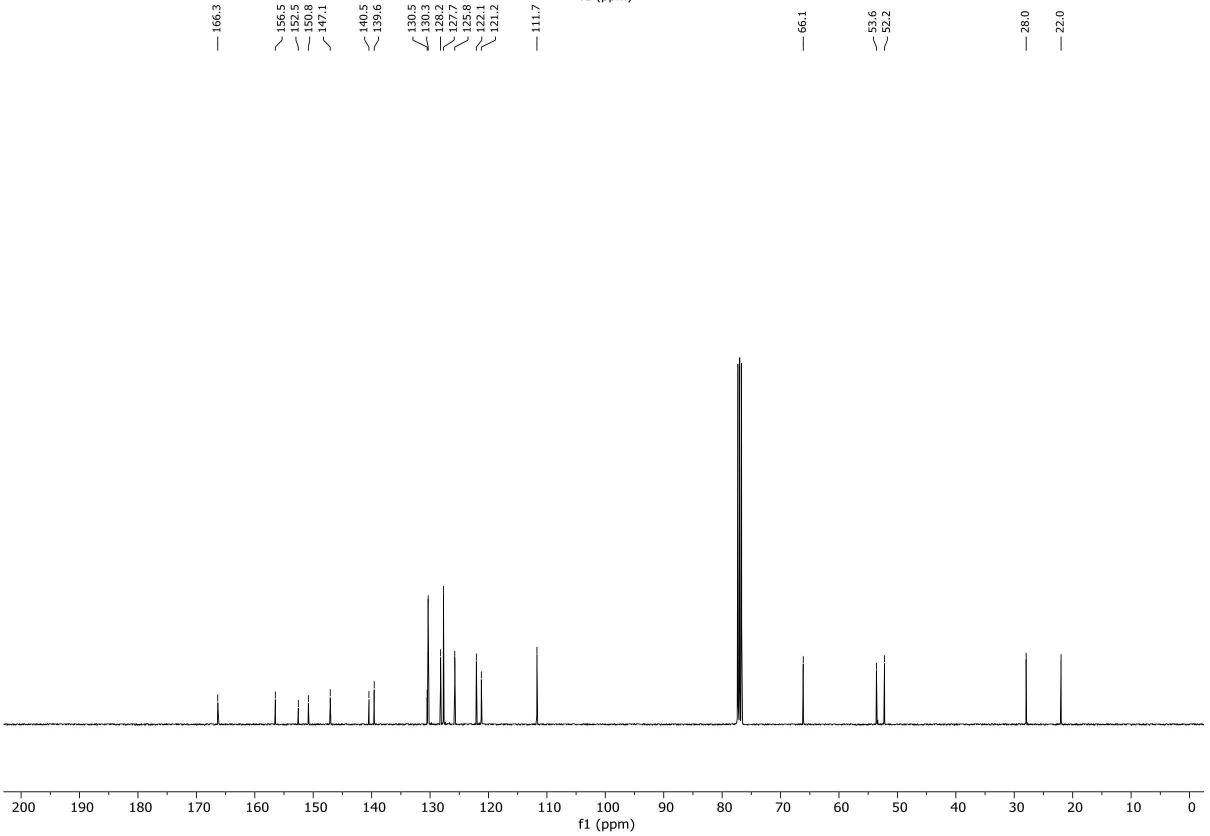
166.3
156.5
152.5
150.8
147.1
140.5
139.6
130.5
130.3
128.2
127.7
125.8
122.1
121.2
111.7
66.1
53.6
52.2
28.0
22.0
f1 (ppm)

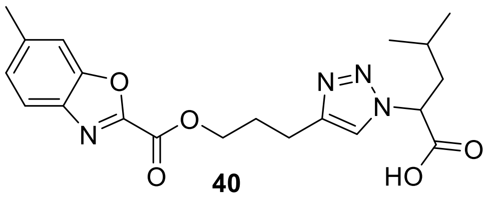

N=N
N
O
N
O
O
HO
O

40

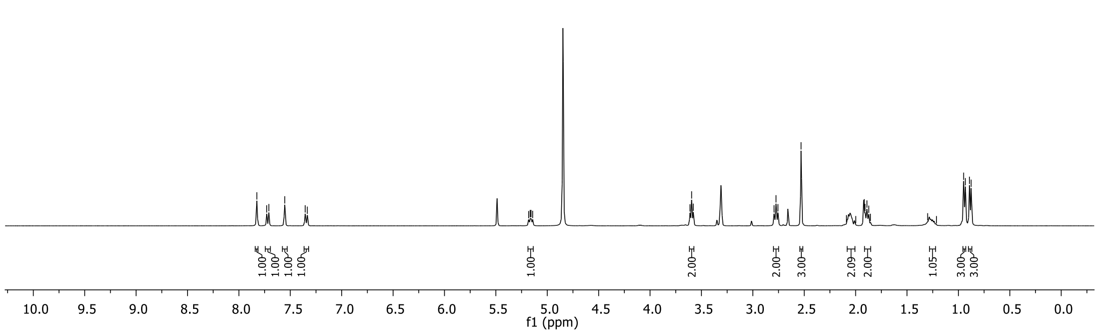

7.83
7.73
7.71
7.55
7.35
7.33
5.18
5.17
5.16
5.14
3.61
3.60
3.58
2.79
2.77
2.76
2.53
2.09
2.00
1.92
1.91
1.89
1.87
1.86
1.30
1.21
0.95
0.93
0.89
0.88
1.00
1.00
1.00
1.00
1.00
2.00
2.00
3.00
2.09
2.00
1.05
3.00
3.00
10.0 9.5 9.0 8.5 8.0 7.5 7.0 6.5 6.0 5.5 5.0 4.5 4.0 3.5 3.0 2.5 2.0 1.5 1.0 0.5 0.0
f1 (ppm)

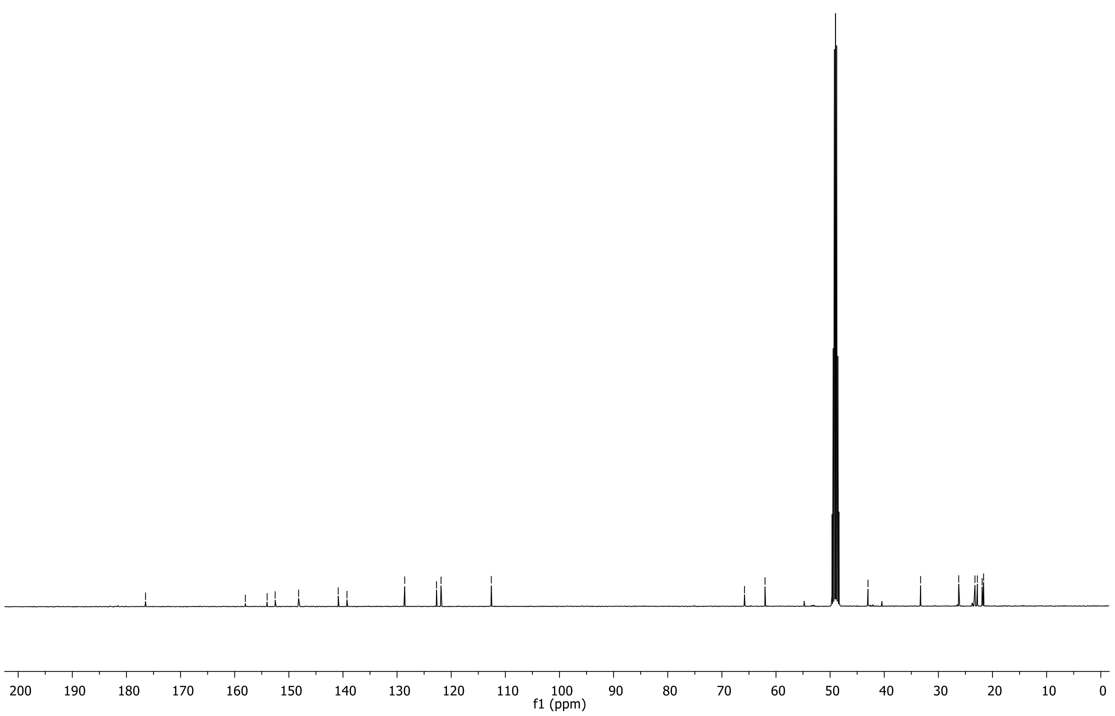

176.5
158.0
154.0
152.5
148.2
140.9
139.3
128.6
122.7
121.9
112.6
65.8
62.0
43.0
33.3
26.2
23.2
22.8
21.9
21.6
200 190 180 170 160 150 140 130 120 110 100 90 80 70 60 50 40 30 20 10 0
f1 (ppm)

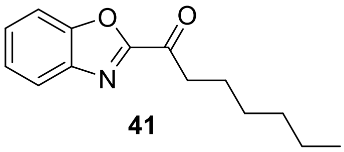
O
N
O

41

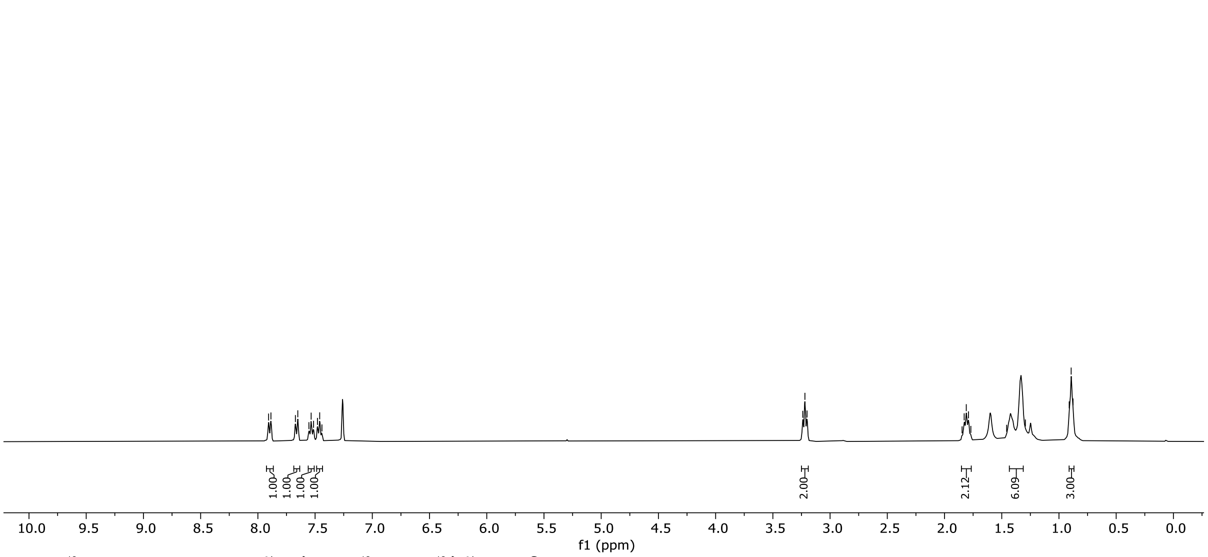
7.91
7.89
7.67
7.65
7.55
7.53
7.51
7.48
7.46
7.44
3.24
3.22
3.20
1.85
1.83
1.81
1.79
1.77
1.46
1.29
0.91
0.89
0.88
1.00
1.00
1.00
1.00
2.00
2.12
6.09
3.00
10.0 9.5 9.0 8.5 8.0 7.5 7.0 6.5 6.0 5.5 5.0 4.5 4.0 3.5 3.0 2.5 2.0 1.5 1.0 0.5 0.0
f1 (ppm)

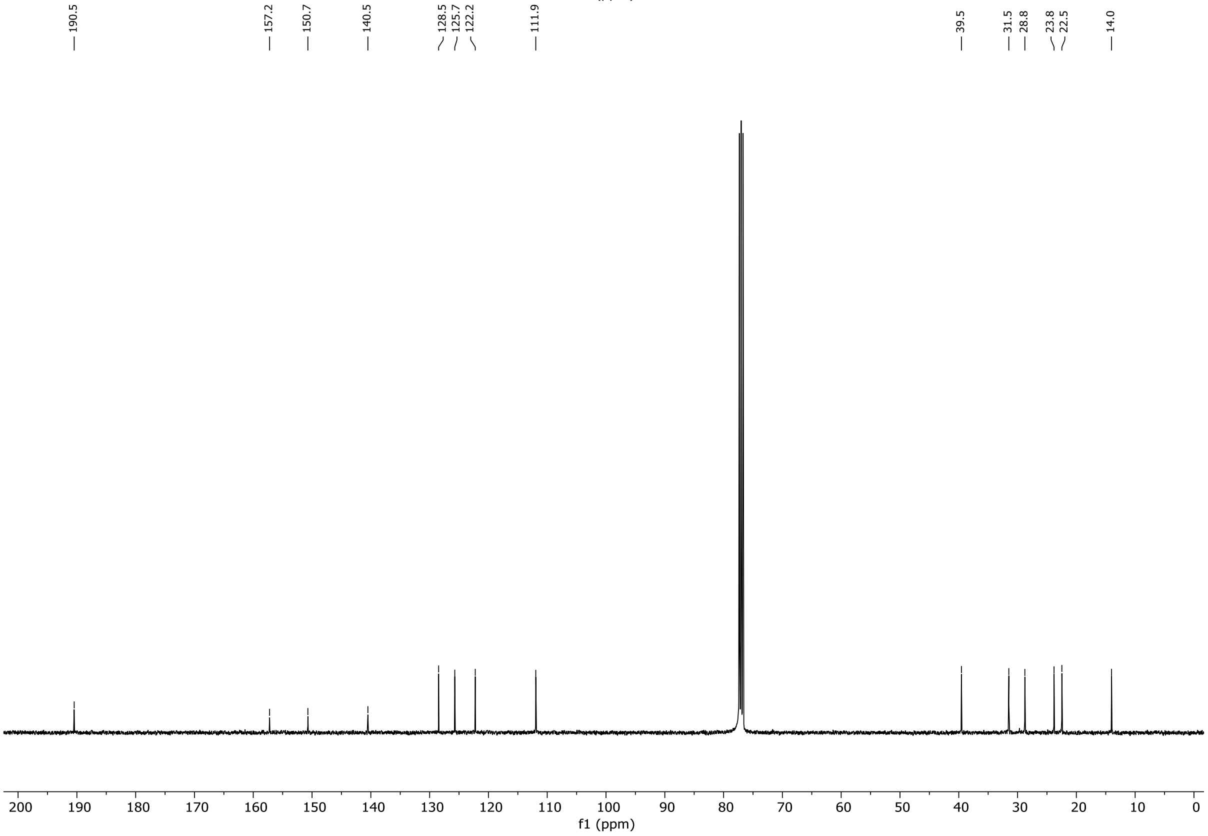
190.5
157.2
150.7
140.5
128.5
125.7
122.2
111.9
39.5
31.5
28.8
23.8
22.5
14.0
200 190 180 170 160 150 140 130 120 110 100 90 80 70 60 50 40 30 20 10 0
f1 (ppm)
